# Self-assembling scaffolds epigenetically reactivate and electroactively guide neuronal regeneration to restore central neural circuits

**DOI:** 10.1038/s41467-026-72397-6

**Published:** 2026-05-04

**Authors:** Shiqiang Tong, Shuai Ye, Fenfen Ma, Xiaoying Xie, Yinzhe Sun, Chuchu Ma, Tiantian Shi, Zheng Cheng, Chang Li, Weili Han, Laozhi Xie, Songlei Zhou, Jianing Gong, Chen Huang, Yukun Huang, Gan Jiang, Xiaolin Liu, Bing Li, Feng Zeng, Jingru Gong, Zhihua Wang, Xiaoling Gao, Qiyong Mei, Wei-Guang Li, Jun Chen

**Affiliations:** 1 Shanghai Pudong Hospital & Department of Pharmaceutics, School of Pharmacy, Key Laboratory of Smart Drug Delivery, Ministry of Education, State Key Laboratory of Advanced Drug Formulations for Overcoming Delivery Barriers, Fudan University, Shanghai, China; 2 NMPA Key Laboratory for Research and Evaluation of Pharmaceutical Preparations and Excipients, State Key Laboratory of Natural Medicines, Department of Pharmaceutics, China Pharmaceutical University, Nanjing, China; 3 Department of Rehabilitation Medicine, Huashan Hospital, Center for Clinical Neuro-AI, Institute for Translational Brain Research, State Key Laboratory of Brain Function and Disorders and Ministry of Education Frontiers Center for Brain Science, Fudan University, Shanghai, China; 4 Research Center for Clinical Medicine, Jinshan Hospital Affiliated to Fudan University, Shanghai, China; 5 Department of Pharmacy, Shanghai Pudong Hospital, Fudan University Pudong Medical Center, Shanghai, China; 6 School of Medicine, Shanghai University, Shanghai, China; 7 Basic Medicine Experimental Teaching Center, Shanghai Jiao Tong University School of Medicine, Shanghai, China; 8 Department of Pharmacology and Chemical Biology, State Key Laboratory of Oncogenes and Related Genes, Shanghai Universities Collaborative Innovation Center for Translational Medicine, Shanghai Jiao Tong University School of Medicine, Shanghai, China; 9 Department of Neurosurgery, Changzheng Hospital, Naval Medical University, Shanghai, China; 10 Department of Emergency, Shanghai Pudong Hospital, Fudan University Pudong Medical Center, Shanghai, China; 11 Ministry of Education-Shanghai Key Laboratory for Children’s Environmental Health, Xinhua Hospital, Shanghai Jiao Tong University School of Medicine, Shanghai, China

**Keywords:** Nanoparticles, Drug delivery

## Abstract

Central nervous system (CNS) injury is a leading cause of death and long-term disability worldwide. Neurological deficits reflect disruption of central neural circuits. A major barrier to circuit repair is the intrinsically low regenerative potential of adult CNS neurons—linked in part to failure of injury-induced nuclear export of class IIa histone deacetylases (notably HDAC5)—together with a hostile post-injury microenvironment. Here we present a multifunctional nanosystem, encapsulating the class IIa HDAC4/5-selective inhibitor LMK-235 and featuring an electroactive polyaniline coating with asymmetrically distributed 5-hydroxytryptamine moieties. Upon reaching the lesion, our nanosystem assembles into large-pore scaffolds that (i) inhibit the activity of nuclear-retained class IIa HDACs in neurons and thereby reactivate intrinsic regenerative programs, (ii) regulate microglial activation to mitigate neuroinflammation, and (iii) provide an electroactive interface promoting activity-dependent synaptic reconnection. This multi-pronged approach illustrates an integrated platform with translational potential for CNS disorders in which circuit disconnection constrains recovery.

## Abstract (web summary)

CNS injury disrupts neural circuits, with poor regeneration due to HDAC retention and hostile environments. Here, the authors show a nanosystem with LMK-235 that forms electroactive scaffolds and promotes regeneration and enhances synaptic reconnection

## Introduction

Central nervous system (CNS) injuries represent a global health crisis, impacting nearly 70 million people annually and causing severe, often irreversible deficits in cognition, motor coordination, and sensory processing^1–3^. Existing clinical interventions remain largely supportive rarely achieve meaningful restoration of neural function^4^. At the core of this challenge is the limited intrinsic regenerative capacity of CNS neurons, compounded by a profoundly hostile injury microenvironment. Achieving substantial recovery therefore requires therapeutic strategies that can overcome these multifaceted barriers and enable functional neural circuit reconstruction.

Neural circuits depend on precisely organized synaptic connections to process and transmit information^5^. Following CNS injury, rapid neuronal death and axonal disruption precipitate acute circuit breakdown. These primary insults trigger secondary pathologies, including chronic inflammation, blood-brain barrier (BBB) compromise, calcium dysregulation, oxidative stress, and progressive neuronal loss^6–11^, which further destabilize local networks. Consequently, most CNS lesions fail to engage effective self-repair, leading to persistent circuit dysfunction, profound neurological impairments, and increased susceptibility to subsequent neurodegenerative processes^12,13^. A successful therapeutic strategy must therefore do more than preserve neurons: it must promote axonal regrowth, provide guidance across the brain, and support durable synaptic reconnection within appropriate networks.

Most efforts to treat CNS injuries have targeted single pathological mechanisms, such as neuroinflammation, oxidative stress, or mitochondrial dysfunction^14–17^. Although such interventions can transiently reduce neuronal loss, they seldom produce sustained improvements in circuit connectivity or function^18–22^. Indeed, neuronal survival alone does not ensure recovery; functional restoration requires that surviving neurons re-establish precise synaptic connections and reintegrate into relevant networks^5,12,13,23,24^. Biomaterial scaffolds implanted at injury sites have therefore emerged as promising platforms to provide structural guidance for regenerating axons and to foster synaptic reconnection^25–29^. However, several limitations remain. First, CNS neurons exhibit intrinsically limited regenerative capacity relative to peripheral neurons, in part because injury fails to robustly induce intrinsic growth-promoting programs^12,13,30–32^. Second, persistent neuroinflammation, oxidative imbalance and gliosis create a microenvironmental barrier that inhibits axonal extension and functional integration^18,33–37^. Third, invasive implantation of conventional scaffolds can exacerbate tissue compression and intracranial pressure, increase infection risk, and induce secondary injury, collectively restricting clinical feasibility^36,38,39^.

Recent studies implicate epigenetic dysregulation—particularly involving class IIa histone deacetylases (HDACs), including HDAC5—as a key molecular bottleneck that constrains intrinsic regenerative responses^40–51^. After CNS injury, many neurons fail to trigger nuclear export of HDAC5, resulting in reduced histone H3 acetylation at regeneration-associated loci and insufficient activation of pro-regenerative transcriptional cascades^30,41,50–54^. Thus, relieving class IIa HDAC-dependent repression represents a compelling route to re-engage intrinsic growth programs. Importantly, class II HDAC dysregulation extends beyond neuronal growth competence: it also shapes microglial activation states, thereby amplifying neuroinflammation and reinforcing a regeneration-inhibitory milieu^19,55–57^. Effective CNS repair is therefore likely to require integrated strategies that simultaneously (i) lift epigenetic constraints on neuronal regeneration, (ii) normalize microglial-driven inflammation, and (iii) provide structural and functional guidance for synaptic reconnection.

**Fig. 1:**
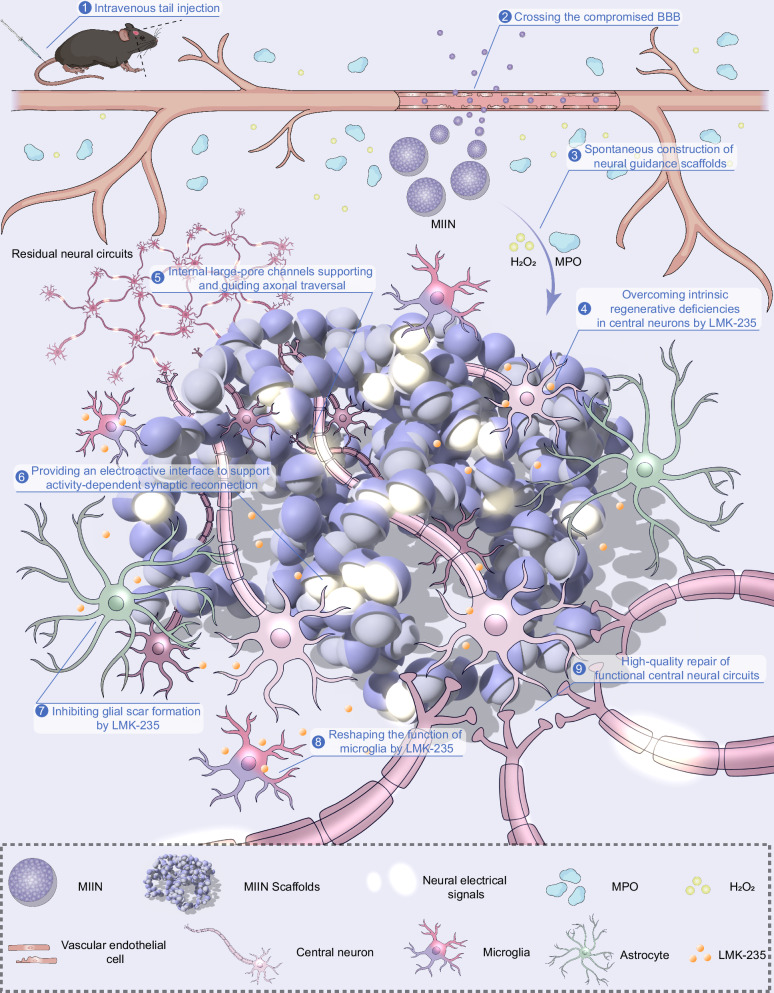
MIIN activate and guide neuronal regeneration to reconstruct central neural circuits. The functions of MIIN are categorized as follows: (1) Prolonged circulation: MIIN undergo prolonged circulation after intravenous injection. (2) BBB penetration: MIIN penetrate the severely compromised BBB and accumulate in the CNS injury region. (3) Responsive scaffold formation: In response to elevated levels of myeloperoxidase at the injury site, MIIN spontaneously assemble into neural guidance scaffolds with internal large-pore channels. (4) Drug reservoir function: The scaffolds serve as drug reservoirs for sustained release of LMK-235, aiming to overcome epigenetic intrinsic deficiencies in central neurons and subsequently activate their regenerative potential. (5) Axonal support and guidance: The large-pore channels within the scaffolds support and guide the traversal of regenerating axons, laying a bridge for neural repair. (6) Electroactive interface: The electroactive PANI interface is compatible with neuronal activity and supports activity-dependent synaptic reconnection. (7) Astrocyte modulation: The LMK-235 released from the scaffolds inhibits the aberrant activation of astrocytes in the injury microenvironment, thereby reducing the formation of glial scars. (8) Microglia regulation: The sustained release of LMK-235 continuously modulates dysregulated microglia in the injured microenvironment, reshaping and maintaining the homeostasis of the regenerative microenvironment. (9) Functional neural circuit repair: Based on these multi-mechanism cooperative actions, MIIN achieve high-quality repair of functional neural circuits.

To address these intertwined challenges, we engineered a multifunctional, intravenously injectable nanosystem MIIN (Fig. 1). Our platform integrates three design elements: (1) sustained delivery of the class IIa HDAC4/5-selective inhibitor LMK-235 to relieve intrinsic epigenetic constraints in CNS neurons; (2) minimally invasive, lesion-localized formation of self-assembling scaffolds in response to elevated local myeloperoxidase (MPO)^58^, creating axon-permissive channels; and (3) an electroactive polyaniline (PANI) interface that is compatible with neuronal activity and supports activity-dependent processes to promote directional growth and synaptic reconnection^59,60^. To ensure physiologically relevant channel architecture upon aggregation, nanoparticles carry asymmetrically distributed 5-hydroxytryptamine (5-HT) moieties through a Janus design^61–67^. By combining intrinsic epigenetic priming, microenvironmental modulation and guided reconnection within a single lesion-assembled scaffold, our approach aims to rebuild damaged CNS circuitry. This strategy may be broadly applicable not only to traumatic CNS injuries but also to neurodegenerative or ischemic disorders in which epigenetic repression, chronic inflammation and circuit-level dysfunction impede recovery.

## Results

### Engineering and physicochemical validation of MIIN

**Fig. 2:**
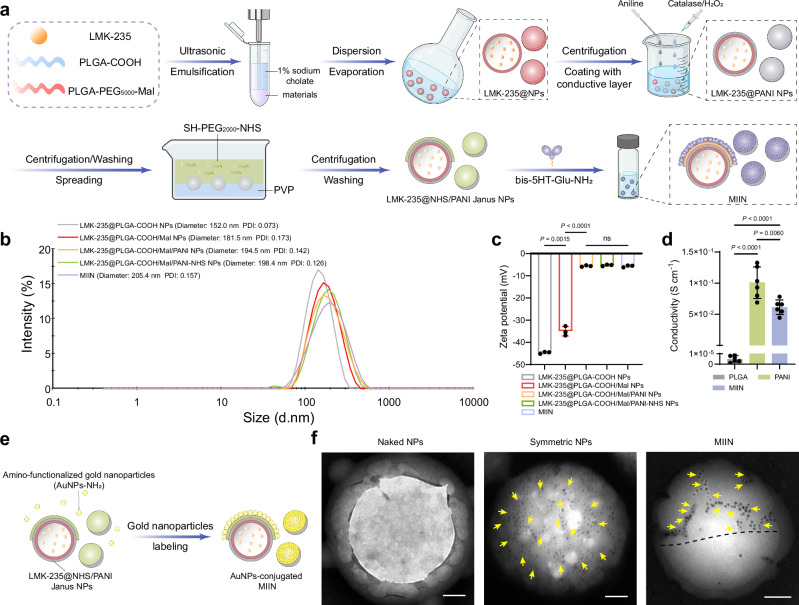
Preparation and characterization of MIIN. **a** Schematic illustration of the preparation process of MIIN. Created in BioRender. Tong, S. (2026) https://BioRender.com/5cuvlhm**b** Size distribution of MIIN detected by DLS. **c** Zeta potential of MIIN (*n* = 3). **d** Electrical conductivity of MIIN (*n* = 6). **e** Scheme for the labeling of MIIN with Au NPs-NH_2_. Created in BioRender. Tong, S. (2026) https://BioRender.com/5cuvlhm**f** TEM images of MIIN labeled with gold nanoparticles. Yellow arrows indicate the gold nanoparticles conjugated to the surface of the formulation. Scale, 50 nm. The experiments were repeated three times independently. The data are presented as the mean ± SD of three independent biological replicates, along with the corresponding P values. Statistical analyses are performed using one-way ANOVA with Tukey’s post hoc test.

To achieve targeted neuronal regeneration, we engineered multifunctional nanoparticles termed MIIN that integrate three design elements: (i) encapsulation of LMK-235 (a class IIa HDAC4/5 selective inhibitor), (ii) a PANI shell to create an electroactive interface, and (iii) asymmetrically distributed 5-HT moieties to control scaffold architecture upon assembly^68–70^. The synthesis comprised staged fabrication and surface functionalization steps (Fig. 2a). We first prepared the ligand bis-5HT-NH_2_ from 5-HT via a two-step reaction^71^, and verified molecular identity and purity by liquid chromatography-mass spectrometry (LC-MS), high performance liquid chromatography (HPLC), and ^1^H nuclear magnetic resonance (^1^H NMR; Supplementary Figs. 1–3). LMK-235-loaded poly(lactic-co-glycolic acid) (PLGA) nanoparticles bearing surface maleimide (Mal) groups were then generated via emulsion solvent evaporation (Fig. 2a). A PANI coating was introduced by in situ polymerization of aniline on the nanoparticle surface, accompanied by a shift in zeta potential from approximately −35 to −5.68 mV (Fig. 2c), consistent with formation of a new surface layer.

To impose hemispherical (“Janus”) surface reactivity, PANI-coated nanoparticles were dispersed on a densely crosslinked polyvinylpyrrolidone (PVP) gel and incubated with thiol-polyethylene glycol_2000_-*N*-hydroxysuccinimide (SH-PEG_2000_-NHS). Steric masking by the PVP matrix selectively limited reactions on one hemisphere, enabling asymmetric presentation of *N*-hydroxysuccinimide (NHS) groups on the exposed side. Subsequently, bis-5HT-glutamate-NH_2_ (bis-5HT-Glu-NH_2_) was conjugated via NHS-amine coupling, yielding MIIN with controlled asymmetric 5-HT distribution.

We generated control formulations lacking one or more key features under identical conditions, including LMK-235@PANI nanoparticles without 5-HT, LMK-235@Bis-5HT/PANI nanoparticles with symmetrically 5-HT distribution, Bis-5HT/PANI Janus nanoparticles without LMK-235, and LMK-235@Bis-5HT Janus nanoparticles without the PANI coating (Supplementary Tabs. 1–4). Dynamic light scattering revealed a uniform hydrodynamic diameter of approximately 205 nm, consistent with transmission electron microscope (TEM) measurements (~200 nm; Fig. 2b, f). LMK-235 encapsulation and loading efficiencies were 70.56 ± 5.62% and 5.88 ± 0.57%, respectively. The PANI layer increased measured conductivity to approximately 6 × 10^−2^ S/cm (Fig. 2d), supporting the presence of an electroactive coating.

To directly validate asymmetric surface functionalization, we substituted bis-5HT-Glu-NH_2_ with amino-functionalized gold nanoparticles (AuNPs-NH_2_). TEM showed gold nanoparticle localized predominantly on one side of MIIN (Fig. 2e, f and Supplementary Fig. 4), consistent with a Janus distribution. Elemental analyses further supported partial versus full surface coverage: gold content was 0.0004% for nanoparticles without gold nanoparticle conjugation, 0.2094% for nanoparticles with symmetric gold nanoparticle conjugation, and 0.1283% for MIIN with asymmetric gold nanoparticle conjugation (Supplementary Fig. 5). Collectively, these data establish robust preparation of MIIN with controlled size, drug loading, electroactive surface coating, and asymmetric surface chemistry suitable for stimulus-responsive scaffold assembly.

### MIIN responsively construct large-pore neural guidance scaffolds

**Fig. 3:**
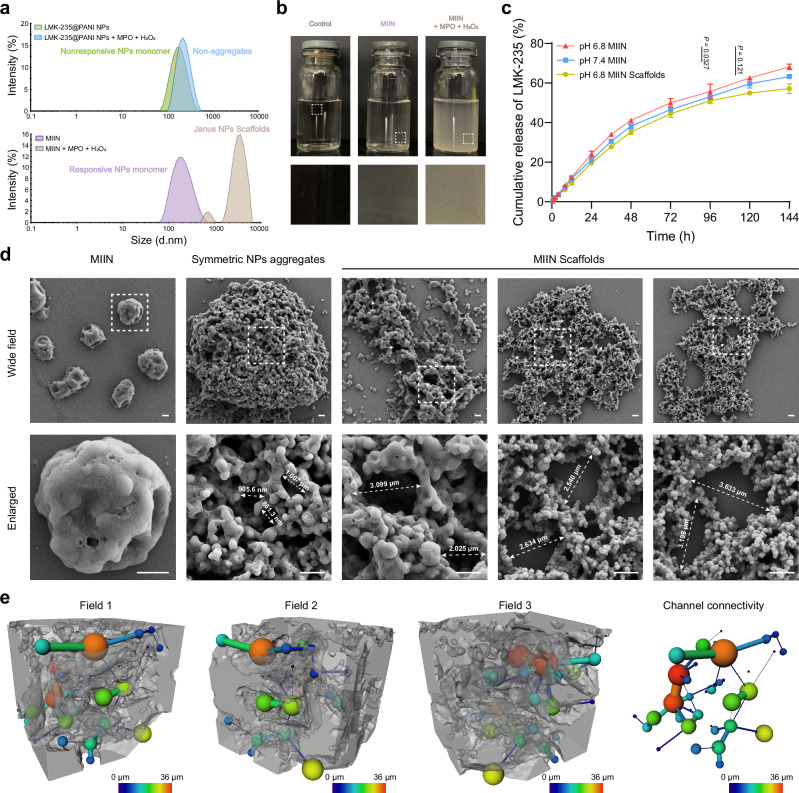
MIIN responsively construct large-pore neural guidance scaffolds. **a** The responsive size variation of MIIN. **b** The responsive morphological changes of MIIN. **c** The release profile of LMK-235 from MIIN before and after scaffolds formation in PBS (pH 6.8 and pH 7.4) at 37 °C (*n* = 3). **d** SEM images depicting the internal channel structure of the MIIN scaffolds. Scale, 50 nm (MIIN); 1 μm (aggregates and scaffolds). The experiments were repeated three times independently. **e** Micro-CT visualization of MIIN scaffolds and an example visualization of the spherical granulometry Micro-CT analysis demonstrating how theoretical spheres of different sizes can access the pore network. The data are presented as the mean ± SD of three independent biological replicates, along with the corresponding P values. Statistical analyses are performed using two-way ANOVA with Tukey’s post hoc test.

To evaluate whether MIIN can assemble into scaffolds under injury-like oxidative conditions, we exposed nanoparticles to a simulated pathological milieu containing MPO and H_2_O_2_. Our system underwent marked size enlargement and formed visible precipitate-like aggregates, whereas LMK-235@PANI nanoparticles lacking bis-5HT remained largely dispersed (Fig. 3a, b and Supplementary Fig. 6), indicating that bis 5HT functionalization is required for robust MPO responsive assembly.

Because neural circuit repair unfolds over weeks, we next quantified LMK-235 release before and after assembly under physiological (pH 7.4) and injury-mimicking (pH 6.8) conditions. MIIN exhibited gradual release with minimal burst leakage, and assembly did not induce premature drug dumping. Approximately 57.14% of LMK-235 was released within initial 144 h, followed by a slower phase, with full release projected over ~45 days (Fig. 3c). Incorporation of 10% fetal bovine serum (FBS) into release media yielded similar kinetics (Supplementary Fig. 7a), consistent with robustness to protein adsorption. Moreover, scaffolds formed after 30 min of in vitro assembly released LMK 235 more slowly than unassembled MIIN, whereas extending assembly time produced minimal additional changes (Supplementary Fig. 7b), suggesting that rapid assembly is sufficient to establish stable release behavior.

We next characterized scaffold pore architecture, a key determinant of axon penetration and guidance^72–74^. SEM revealed that nanoparticles with symmetrically distributed 5-HT densely packed into aggregates with sub-micron pores, whereas our system formed porous scaffolds with internal channels averaging ~3.5 µm, compatible with axonal infiltration and growth (Fig. 3d). Micro-computed tomography (micro-CT) further confirmed an interconnected pore network with a consistent pore size distribution (Fig. 3e and Supplementary Tab. 5). Mechanical assessment showed no detectable alteration in brain tissue elastic modulus upon scaffold integration (Supplementary Fig. 8), supporting mechanical compatibility with CNS tissue.

To evaluate persistence against rapid cellular uptake, we compared internalization of coumarin-6(Cou6)-labeled MIIN versus their assembled scaffolds by activated microglia and injured neurons. Flow cytometry and confocal microscopy showed time-dependent uptake of free nanoparticles, whereas assembled scaffolds exhibited markedly reduced internalization, consistent with size-restricted phagocytic clearance. A minor Cou6 signal remained detectable intracellularly, consistent with partial scaffold degradation and/or fragment uptake (Supplementary Figs. 9, 10). In vitro degradation studies supported slow scaffold breakdown, reaching approximately 98.15% degradation over 120 days (Supplementary Fig. 11). Together, these data indicate that MIIN assemble into mechanically compatible, axon permissive scaffolds that provide sustained LMK-235 release while resisting rapid cellular clearance.

### MIIN efficiently target injured brain tissue and assemble into scaffolds in situ

**Fig. 4:**
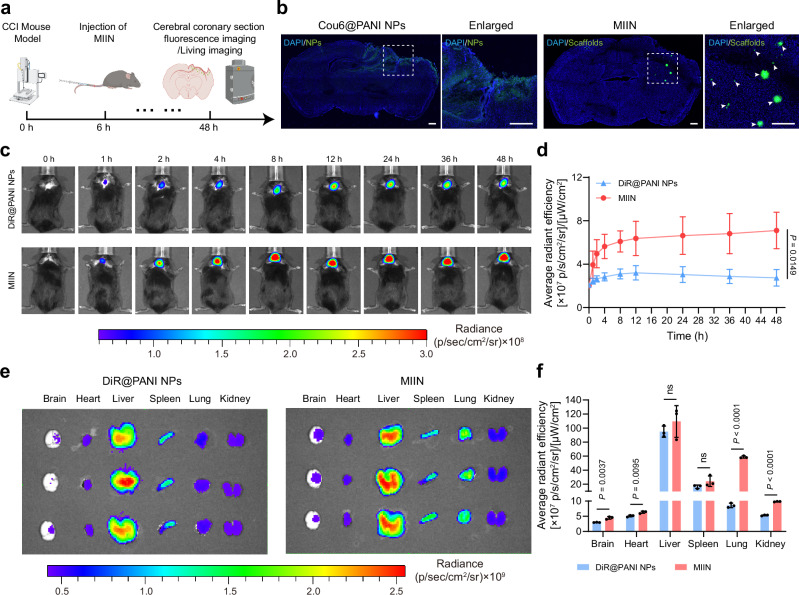
MIIN efficiently target injured brain tissue and assemble into scaffolds in situ. **a** Administration and evaluation regimen. Created in BioRender. Tong, S. (2026) https://BioRender.com/5cuvlhm**b** Brain distribution and aggregation of the Cou6-labeled MIIN. Scale, 500 μm. The experiments were repeated three times independently. **c** Real-time whole-body DiR fluorescence imaging of CCI mice after intravenous injection of MIIN or conductive nanoparticles without bis-5HT modification. **d** Semi-quantitative analysis of the in vivo brain radiant efficiency shown in (**c**) (*n* = 3). **e** Ex vivo DiR fluorescence imaging of main organs obtained from CCI mice at 48 hours post-injection. **f** Semi-quantitative analysis of the ex vivo main organs radiant efficiency shown in (**e**) (*n* = 3). The data are presented as the mean ± SD of three independent biological replicates, along with the corresponding P values. Statistical analyses are performed using two tailed Student’s *t*-test.

Traumatic brain injury (TBI) is characterized by rapid disruption of neural circuits and a transiently compromised BBB, enabling systemically administered nanomaterials to access injured regions^1,6,75^. To evaluate whether our system accumulate in the injured brain and assemble into lesion-resident scaffolds, we established a controlled cortical impact (CCI) mouse model and administered formulations at 6 h post injury (Fig. 4a). This timing was selected because (i) BBB disruption is prominent during the first week after CCI^75^, and (ii) MPO released during the early inflammatory phase—largely within 6–48 h—can catalyze oxidative coupling of 5-HT moieties and thereby promote assembly^76–78^. In our injury paradigm, Evans blue extravasation confirmed substantial BBB leakage, supporting feasibility of brain access by ~200 nm particles under severe injury (Supplementary Fig. 12).

We first tracked DiR-labeled MIIN or DiR-labeled PANI-coated nanoparticles lacking bis-5-HT (control nanoparticles) using longitudinal whole body near infrared (NIR) imaging over 48 h (Fig. 4c). Because NIR whole-body imaging has limited depth resolution, head-associated signals cannot by themselves distinguish parenchymal deposition from superficial tissues (e.g., skin or skull) or intravascular fluorescence. We therefore treated the live imaging primarily as a kinetic readout and complemented it with ex vivo organ imaging and section-level microscopy to establish tissue localization. In vivo, our system exhibited significantly higher head-associated radiant efficiently than control nanoparticles across 1–48 h and showed sustained signal up to 48 h, whereas control nanoparticle signals diminished after ~12 h (Fig. 4c, d), consistent with prolonged circulation (Supplementary Fig. 13) and progressive enrichment in the injured region.

Ex vivo imaging of harvested organs at multiple time points within 48 h showed the expected dominant uptake in clearance organs, particularly the liver, yet our system displayed enhanced brain retention compared with control nanoparticles (Fig. 4e, f, Supplementary Fig. 14a, b). Quantification of organ-level fluorescence further revealed that the brain-to-liver ratio increased over time and reached 15.15 ± 2.40% at 48 h (Supplementary Fig. 14c), indicating improved relative delivery to the brain despite systemic biodistribution typical of nanoparticle therapeutics.

To directly test whether MIIN within injured brain parenchyma and assemble in situ, we administered Cou6-labeled MIIN or Cou6-labeled control nanoparticles and performed confocal imaging on coronal bran sections at 48 h. Our system formed discrete, multicellular scale aggregates concentrated in the lesion core and peri lesional region, whereas control nanoparticles remained largely diffuse and displayed limited accumulation (Fig. 4b). Notably, the MIIN signal appeared as compact deposits embedded within the injured tissue architecture rather than as continuous, vessel like trajectories, supporting extravasation followed by assembly in the injured parenchyma. Together, these results indicate that MIIN achieve enhanced lesion-associated brain accumulation after systemic delivery and undergo MPO responsive assembly into localized scaffold-like structures at the injury site.

### MIIN scaffolds activate regenerative transcription and guide axonal infiltration at the lesion site

**Fig. 5:**
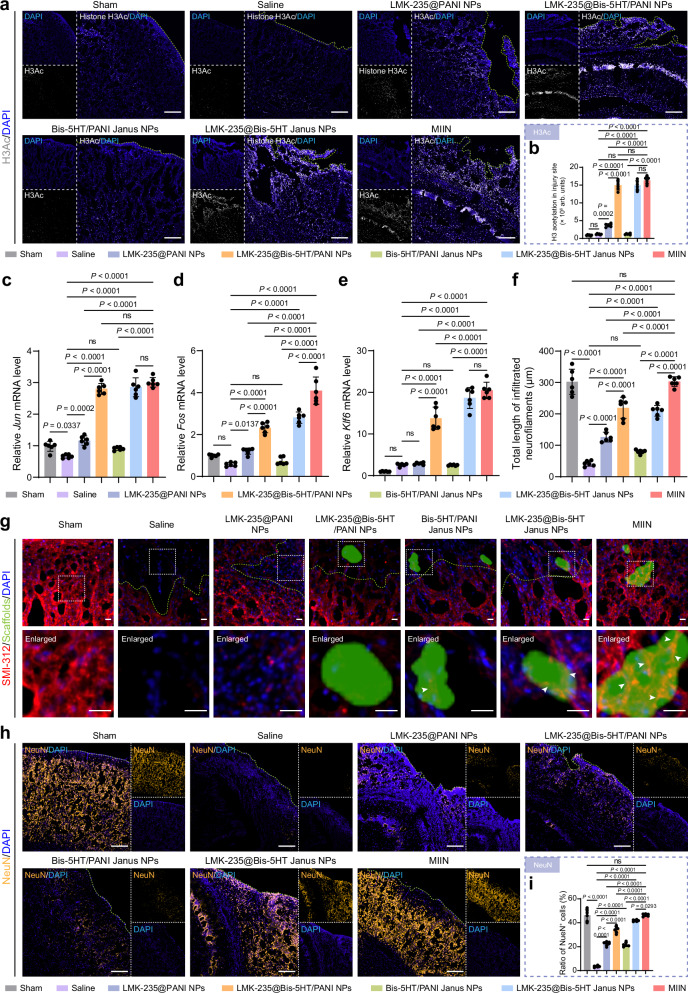
MIIN facilitate and guide axonal regeneration at the injury site. **a** Representative images of histone H3 acetylation levels in injured sites of various groups at 7 days post-injury. Scale, 200 μm. **b** Quantitative analysis of the fluorescence intensity of histone H3 acetylation levels corresponding to (**a**) (*n* = 6). **c–e** qPCR analysis of mRNA levels of (**c**) *Jun*, (**d**) *Fos*, and (**e**) *Klf6* in injured sites (*n* = 6). **f** Quantitative analysis of the total length of neurofilaments infiltrating into the TBI site (*n* = 6). **g** Representative images illustrating the guidance and promotion effects of scaffolds on neuronal axons in the brain injury zone after different treatments for 4 weeks. Arrows indicate the growth of neuronal axons within the scaffolds. Scale, 25 μm. The experiments were repeated six times independently. **h** Representative images of neurons (NeuN) in injured sites of various groups at 28 days after injury. Scale, 200 μm. **i** Quantitative analysis showing the number of NeuN^+^ cells corresponding to (**h**) (*n* = 6). The data are presented as the mean ± SD of six independent biological replicates, along with the corresponding P values. Statistical analyses are performed using one-way ANOVA with Tukey’s post hoc test.

We next tested whether lesion-resident MIIN scaffolds translate into neuronal epigenetic reactivation, axonal regrowth, and tissue-level signs of circuit repair. In the CCI model, mice received MIIN or control formulations (all dosed at 1.5 mg/kg LMK-235 equivalent) at 6 h post-injury. Because injury induced nuclear retention of class IIa HDACs is linked to suppressed histone acetylation and impaired regenerative transcription, we quantified histone H3 acetylation in the lesion region at 7 days. Our system increased histone H3 acetylation at the injury site to ~13.75 fold above untreated controls (Fig. 5a, b), accompanied by elevated mRNA levels of regeneration associated transcription factors (Jun, Fos, and Klf6; Fig. 5c–e)^40–47^.

We then examined whether this epigenetic activation was accompanied by structural indices of repair. At 28 days post injury, Cou6-labeled MIIN scaffolds remained detectable within the lesion, and SMI-312 immunostaining revealed extensive axonal infiltration throughout these scaffold structures (Fig. 5f, g, Supplementary Fig. 15 and Supplementary Movie 1). In contrast, nanoparticles lacking asymmetrically distributed bis 5HT did not form lesion resident scaffolds and exhibited limited local retention (Fig. 5f, g), consistent with the requirement for MPO responsive assembly (Figs. 3, 4). In parallel, our system preserved neuronal populations within the injured cortex: NeuN^+^ mature neuron abundance recovered significantly compared with untreated CCI mice at 28 days (Fig. 5h, i). Component omission controls further indicated that maximizing structural repair required the integrated design (LMK 235 loading, large pore scaffold architecture, PANI coating, and asymmetric bis 5HT functionalization), as removal of individual elements reduced the extent of axonal infiltration and/or neuronal preservation (Fig. 5f–i). Furthermore, in a 3D in vitro neuronal culture model, MIIN scaffolds also effectively activated intrinsic neuronal regenerative programs, markedly promote axonal outgrowth, and facilitate synaptic connectivity (Supplementary Figs. 16, 17).

Given the recognized contribution of glial dysregulation to regeneration failure^18,33–37^, we also assessed whether MIIN modulated the lesion microenvironment. Our system reduced astrocyte reactivity (GFAP) and microglia/macrophage activation (Iba-1) at both early and later time points, and shifted inflammatory mediator profiles toward a more pro-regenerative state (Supplementary Figs. 18–22). In vitro, MIIN scaffolds also countered chronic microglial activation in a repeated-LPS paradigm, including improved mitochondrial membrane potential and reduced oxidative/inflammatory markers (Supplementary Figs. 23, 24)^19,55,79–85^. Finally, unbiased proteomic profiling supported tissue level remodeling consistent with repair: TBI induced broad downregulation of proteins linked to axon regeneration, synaptic transmission and epigenetic regulation, whereas MIIN treatment upregulated proteins associated with cell–matrix adhesion, tissue remodeling and neuronal survival (Supplementary Figs. 25, 26). Together, these convergent readouts support that MIIN scaffolds promote lesion-level regenerative transcription, axonal infiltration into a permissive scaffold architecture, preservation of mature neurons, and coordinated remodeling of the injury milieu.

### MIIN improve long-term neurological function and exhibit favorable biocompatibility

**Fig. 6:**
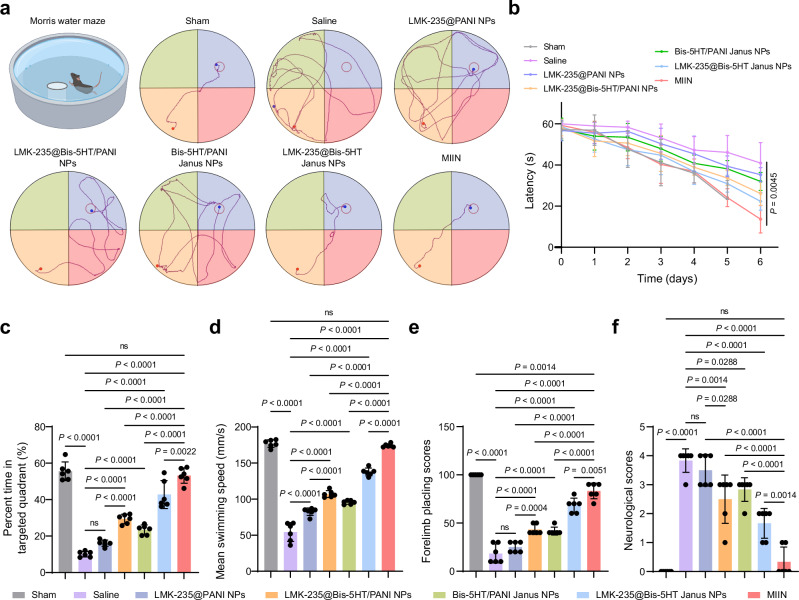
MIIN facilitate enduing recovery of neurological function after TBI. **a** Representative swimming track of the Morris water maze assays on TBI mice at 28 days post-injury. The experiments were repeated six times independently. Created in BioRender. Tong, S. (2026) https://BioRender.com/5cuvlhm**b–d** Quantitative analysis from the Morris water maze assays at 28 days post-injury, including (**b**) escape latency over trial days, (**c**) percentage of time spent in the target quadrant, and (**d**) swimming speed (*n* = 6). **e** Forelimb placing scores assessed at 28 days post-injury (*n* = 6). **f** Neurological scores assessed at 28 days post-injury (*n* = 6). The data are presented as the mean ± SD of six independent biological replicates, along with the corresponding P values. Statistical analyses are performed using two-way ANOVA for escape latency (**b**) and one-way ANOVA (for **c**–**f**), followed by Tukey’s post hoc test.

To determine whether structural and molecular repair translated into functional recovery, we evaluated neurological outcomes at 28 days post CCI using a behavioral battery. In the Morris water maze, MIIN treated-mice showed more direct swim trajectories to the hidden platform than other groups (Fig. 6a), with reduced escape latency, increased time in the target quadrant, and increased mean swimming speed (Fig. 6b–d). In sensorimotor assays, our system improved forelimb placing performance (Fig. 6e) and reduced neurological deficit scores toward levels seen in uninjured controls (Fig. 6f). These improvements were consistent across tasks that probe coordinated movement and sensorimotor integration (Supplementary Fig. 27).

We also assessed systemic safety in healthy C57BL/6 mice receiving the same dosing regimen. MIIN did not significantly alter peripheral blood cell counts (WBCs, granulocytes, lymphocytes and monocytes) (Supplementary Fig. 28a–d) or serum markers of hepatic and renal function (ALT, AST, BUN and creatinine) (Supplementary Fig. 28e–h). Histological analysis revealed no discernible structural abnormalities in major organs (heart, liver, spleen, lung and kidney) (Supplementary Fig. 29). Together, these data indicate that our system promote sustained functional recovery after TBI while showing a favorable short-term biocompatibility profile under the tested conditions.

### Mechanistic contributions of sustained class IIa HDAC inhibition and activity dependent guidance

**Fig. 7:**
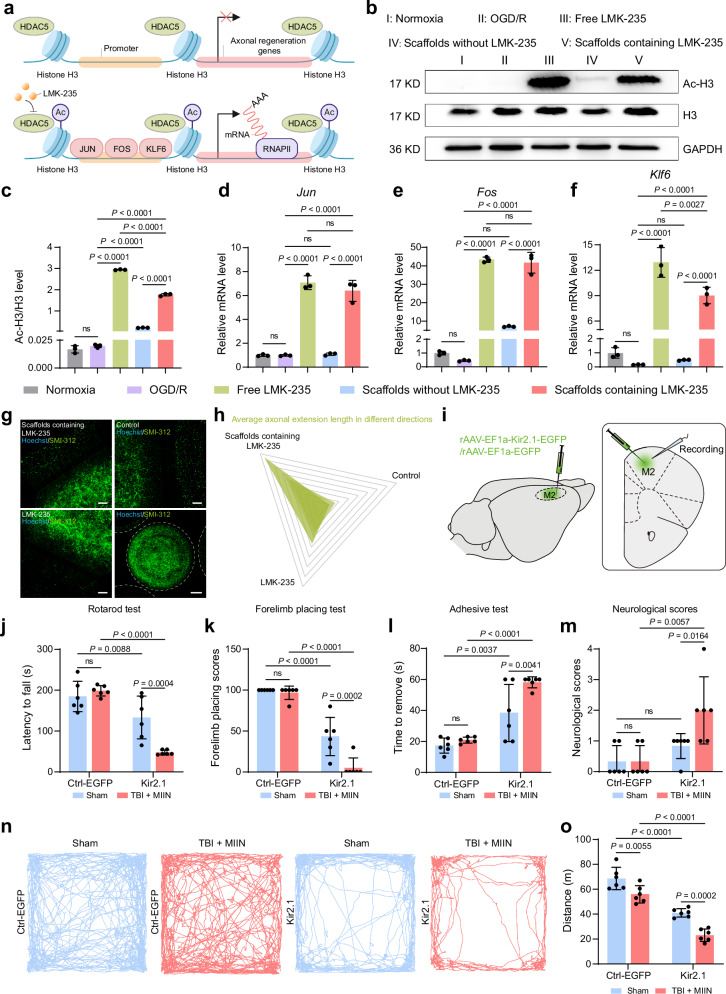
Mechanisms underlying synergistic activation and guidance of synaptic reconnection by MIIN components. **a** Schematic representation of the mechanism of action of LMK-235. Created in BioRender. Tong, S. (2026) https://BioRender.com/5cuvlhm**b** The Western blot analysis of histone H3 acetylation levels. The experiments were repeated three times independently. **c** Semi-quantitative analysis of histone H3 acetylation levels corresponding to (**b**) (*n* = 3). **d–f** qPCR analysis of mRNA levels of (**d**) *Jun*, (**e**) *Fos*, and (**f**) *Klf6* (*n* = 3). **g** Representative images depicting axonal growth of primary neurons in a microfluidic culture platform. Scale, 1 mm (scan); 200 μm (enlarged image). The experiments were repeated three times independently. **h** A radar chart illustrating the directional growth of axons in primary neurons corresponding to (**g**) (*n* = 3). **i** Schematic representation of the rAAV-EF1α-Kir2.1-EGFP or rAAV-EF1α-EGFP injection. **j-m** Assessments of (**j**) latency on the rotarod, (**k**) forelimb placing scores, (**l**) time to remove adhesive stickers, (**m**) neurological scores of M2^Kir2.1^ mice and control mice after MIIN treatment (*n* = 6). **n** Representative movement track from the open field test. **o** Quantitative analysis of the total distance in open field test (*n* = 6). The data are presented as the mean ± SD of three or six independent biological replicates, along with the corresponding P values. Statistical analyses are performed using one-way ANOVA (for **c**–**f**) and two-way ANOVA (for **j–m**,**o**) with Tukey’s post hoc test.

To dissect how MIIN coordinate neuronal reprogramming and reconnection, we examined two mechanistic axes: sustained class IIa HDAC inhibition by LMK 235 and an activity dependent contribution associated with the PANI coated scaffold interface. Because central neurons fail to efficiently export class IIa HDACs after injury, HDAC-dependent repression persists in the nucleus, limiting histone acetylation and suppressing regeneration-associated transcriptional programs (Fig. 7a). Consistent with this framework, injured neurons co-cultured with LMK-235-loaded scaffolds exhibited robustly increased histone H3 acetylation relative to untreated controls (Fig. 7b, c), accompanied by marked induction of regeneration-linked transcription factors Jun, Fos and Klf6 (Fig. 7d–f)^40–47^.

Because LMK-235 inhibits HDAC4 and HDAC5 with comparable potency, we tested whether HDAC4 inhibition alone was sufficient to reproduce the pro-regenerative phenotype. Under the same neuronal injury paradigm, the HDAC4-selective inhibitor tasquinimod did not significantly increase the axonal marker SMI-312, whereas LMK-235 increased SMI-312 by 18.37% (Supplementary Fig. 30), indicating that HDAC4 inhibition alone is not sufficient under these conditions.

We next assessed whether MIIN provide a directional cue that recruits axons toward the scaffold. In a microfluidic device^86,87^, axons preferentially extended toward chambers containing LMK-235–loaded scaffolds compared with chambers containing free LMK-235, while minimal outgrowth was observed toward blank medium (Fig. 7g, h), consistent with scaffold-localized, sustained release enhancing axonal recruitment.

To determine whether neuronal electrical activity is required for the functional benefit of MIIN in vivo, we suppressed excitability by expressing Kir2.1 potassium channels in neurons within the secondary motor cortex (M2) (Fig. 7i). Viral transduction and electrophysiological recordings confirmed reduced neuronal firing at the injection site (Supplementary Fig. 31). Under these conditions, MIIN-treated M2^Kir2.1^ mice exhibited substantially attenuated recovery across behavioral readouts, including rotarod latency, forelimb placing, adhesive removal, neurological deficit scores and open-field locomotion (Fig. 7j–o).

In parallel, whole-cell patch-clamp recordings showed that MIIN enhanced spontaneous synaptic transmission in vitro and restored spontaneous synaptic activity in acute cortical slices ex vivo (Supplementary Fig. 32), consistent with improved functional connectivity when neuronal networks remain electrically competent. Notably, because the PANI coating altered both conductivity and zeta potential (Fig. 2c, d), these experiments do not resolve whether the observed activity requirement dependence reflects electrical permissiveness, surface‑mediated cell–material interactions, or a combination of both.

### MIIN restore functional motor circuitry via reconstruction of M2 → CPu projections

Finally, we asked whether MIIN-enabled reconnection is causally linked to restoration of a defined motor circuit disrupted by CCI. The secondary motor cortex (M2), which is encompassed by the injury region in our model, projects to the dorsal striatum (caudate-putamen, CPu), a pathway essential for coordinated movement^88^. We therefore tested whether M2→CPu projection activity mediates MIIN-associated motor recovery using projection-specific chemogenetic modulation.

**Fig. 8:**
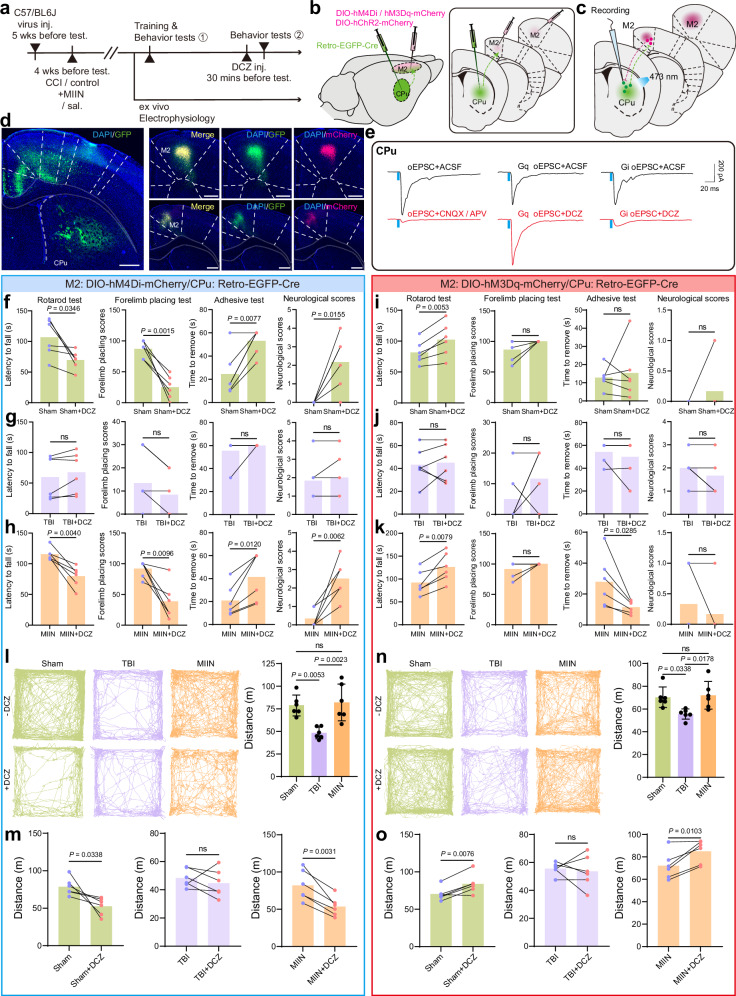
MIIN restore functional motor circuitry via reconstruction of secondary motor cortex-dorsal striatum projections. **a** Drug administration and testing regimen. **b** Schematic representation of viral injections: rAAV-DIO-hM4Di-mCherry or rAAV-DIO-hM3Dq-mCherry into the M2, rAAV-DIO-hChR2-mCherry into M2, and Retro-AAV-EGFP-Cre into the CPu. **c** Schematic of optogenetic activation and electrophysiological recording. **d** Representative images showing AAV-transduced neurons in the M2 and CPu regions. Scale, 500 μm. The experiments were repeated six times independently. **e** Amplitude of optogenetically evoked excitatory postsynaptic currents (oEPSCs) in CPu neurons after incubation of with artificial cerebrospinal fluid (ACSF) or DCZ. **f** Behavioral assessments of hM4Di (Gi) sham mice after PBS or DCZ injection (*n* = 6). **g** Behavioral assessments of hM4Di (Gi) TBI mice after PBS or DCZ injection (*n* = 6). **h** Behavioral assessments of hM4Di (Gi) TBI mice treated with MIIN after PBS or DCZ injection (*n* = 6). **i** Behavioral assessments of hM3Dq (Gq) sham mice after PBS or DCZ injection (*n* = 6). **j** Behavioral assessments of hM3Dq (Gq) TBI mice after PBS or DCZ injection (*n* = 6). **k** Behavioral assessments of hM3Dq (Gq) TBI mice treated with MIIN after PBS or DCZ injection (*n* = 6). **l** Representative movement track and quantitative analysis of total distance traveled in the open field test for hM4D(Gi) mice (*n* = 6). **m** Quantitative analysis of total distance traveled in the open field test for hM4D (Gi) mice after PBS or DCZ injection (*n* = 6). **n** Representative movement track and quantitative analysis of total distance traveled in the open field test for hM3D (Gq) mice (*n* = 6). **o** Quantitative analysis of the total distance traveled in the open field test for hM3D (Gq) mice after PBS or DCZ injection (*n* = 6). The data are presented as the mean ± SD of six independent biological replicates, along with the corresponding P values. Statistical analyses are performed using paired two tailed Student’s *t*-test for(**f–k**,**m**,**o**), and one-way ANOVA for panels **l** and **n**, followed by Tukey’s post hoc test. Some data points are not shown due to overlap of sample points.

We expressed Cre-dependent excitatory (hM3Dq) or inhibitory (hM4Di) DREADDs in M2 neurons projecting to CPu by combining Cre-dependent AAVs in M2 with retrograde Cre delivery from CPu (Fig. 8a, b, d). To validate synaptic connectivity and manipulation efficacy, we co-expressed ChR2 in M2 and recorded optogenetically evoked EPSCs (oEPSCs) in CPu neurons (Fig. 8c). oEPSCs were abolished by CNQX (20 μM) and APV (50 μM), confirming glutamatergic M2 inputs. Incubation with the DREADD agonist deschloroclozapine (DCZ, 50 μM) bidirectionally modulated oEPSC amplitudes in hM3Dq- and hM4Di-expressing projections (Fig. 8e), establishing effective control of M2→CPu transmission.

We then assessed behavior under projection-specific inhibition or activation. In the absence of DCZ, our system improved motor outcomes in both DREADD cohorts (Supplementary Fig. 33). In the hM4Di (Gi) cohort, DCZ reduced motor performance in both sham and MIIN-treated mice, including decreased rotarod latency, reduced forelimb placing scores, prolonged adhesive removal, and increased neurological deficit scores (Fig. 8f–h), together with reduced locomotion in the open field (Fig. 8l, m). Conversely, in the hM3Dq (Gq) cohort, DCZ increased locomotion in sham mice without improving coordination, whereas MIIN-treated mice exhibited improvements in both locomotion and coordinated performance (Fig. 8i–k, n, o). Importantly, TBI mice without MIIN remained severely impaired regardless of DCZ administration (Fig. 8g, j), indicating that chemogenetic modulation alone is insufficient to restore function when the projection remains structurally and functionally compromised. Collectively, these data support that MIIN promote functional recovery by enabling restoration of a defined cortico-striatal pathway, and that the recovered motor performance depends on M2→CPu projection activity.

## Discussion

Restoring functional after CNS injury ultimately requires circuit-level repair, not only neuroprotection. Although many approaches can mitigate secondary pathologies—such as neuroinflammation, oxidative stress, and mitochondrial dysfunction^14–17^—durable recovery depends on whether surviving neurons regenerate axons, reconnect appropriately, and re-establish functional synaptic transmission^5^.

This requirement remains difficult to meet in TBI, where intrinsic growth programs in CNS neurons are weak^12,13,30–32^ and the lesion milieu (reactive glia, inflammatory mediators, oxidative stress, and glial scarring) actively suppresses regrowth^18,33–37^. Implantable polymer scaffolds can provide physical guidance in peripheral nerve repair^25–29^, but implantation in the brain raises translational concerns, including invasiveness, secondary tissue damage, and risks related to intracranial pressure and infection^36,38,39,89^. These constraints motivate strategies that can deliver guidance structures to the lesion minimally invasively, while simultaneously reactivating neuronal regenerative competence and remodeling the inhibitory microenvironment.

Here we report a modular platform—MIIN—that is intravenously administered and forms lesion-resident, large pore scaffolds in situ. The central design principle is to decouple the clinical practicality of systemic dosing from the biological need for local, sustained, multi-modal intervention. Rather than implanting a preformed scaffold, MIIN exploit injury-associated biochemical cues to assemble into porous guidance matrices at the lesion site (Figs. 1–4). This assembly provides two advantages: (i) structural guidance through a macroporous architecture permissive to axonal entry and growth; and (ii) a local drug reservoir enabling sustained epigenetic modulation during the extended timescale of circuit repair.

A central obstacle for CNS regeneration is the failure to initiate a coordinated transcriptional regeneration program after axonal injury^12,13,30–32^. Accumulating evidence places epigenetic repression—particularly class IIa HDAC signaling—upstream of this transcriptional bottleneck^40–51^. In this context, the lesion-resident, sustained delivery enabled by MIIN is well suited to engage chromatin-based constraints that unfold over prolonged repair timescales. Consistent with this framework, we observed increased histone H3 acetylation together with induction of canonical regeneration-associated transcriptional programs in vivo, supporting epigenetic reactivation of intrinsic growth competence.

At the same time, the pharmacology of LMK-235 motivates careful interpretation. Because LMK-235 targets class IIa HDACs with comparable potency toward HDAC4 and HDAC5, our results are best framed as a mechanism grounded in class IIa HDAC4/5 inhibition rather than strict isoform exclusivity. Notably, however, a pharmacological comparison using an HDAC4-selective inhibitor (tasquinimod) did not recapitulate LMK-235’s pro-regenerative effect on an axonal readout, suggesting that HDAC4 inhibition alone is insufficient in this injury paradigm. Although these results cannot fully exclude cooperative or context-dependent contributions from multiple class IIa HDACs, they are compatible with a dominant contribution from HDAC5-linked pathways, consistent with prior work implicating HDAC5 as a regulator of injury-responsive growth programs^41^. Future studies combining isoform-resolved pharmacology with cell type–specific genetic perturbations will be important for defining causal attribution and delineating downstream transcriptional modules with higher precision.

At the materials level, achieving guidance that is permissive to neurite infiltration is nontrivial because nanoparticles typically form dense aggregates with limited internal accessibility. By imposing an asymmetric (“Janus”) distribution^66,67^ of 5-HT moieties, MIIN assemble into scaffolds with internal channels averaging ~3.5 μm, a size range compatible with axonal entry and traversal^72–74^. Under injury-like oxidative conditions (MPO/H_2_O_2_)^58,61^, MIIN rapidly assemble, whereas controls lacking bis-5HT show minimal aggregation, indicating that 5-HT chemistry is essential for responsive scaffold formation. Importantly, assembled scaffolds resist rapid cellular internalization relative to free nanoparticles, consistent with size-limited phagocytic clearance and enabling longer lesion residence—an especially relevant consideration given that regeneration and synaptic stabilization evolve over weeks.

Sustained intervention is also required at the level of molecular signaling. MIIN exhibited gradual drug release with minimal premature leakage and a projected ~45-day release window, supporting prolonged engagement of epigenetic targets and microenvironmental remodeling. This temporal profile aligns with the biological logic of circuit repair: stable reconnection depends on extended periods of axonal growth, synaptic maturation, and network refinement, all of which are unlikely to be supported by short-lived bursts of bioactivity.

A practical challenge for systemically delivered nanomaterials is that clearance organs (particularly the liver) often dominate biodistribution, raising the question of whether brain delivery is sufficient to drive therapeutic effects. In this study, live near-infrared imaging was treated primarily as a kinetic readout, rather than definitive evidence of parenchymal localization, because head-associated signal can arise from superficial tissues or vasculature. We therefore relied on ex vivo organ imaging across early time points and, critically, section-level evidence of discrete aggregates embedded within injured cortex, supporting parenchymal extravasation followed by in situ assembly rather than purely intravascular signal. Quantification indicated increased injury-associated brain enrichment relative to the systemic sink (brain-to-liver ratio reaching ~15% at 48 h). This does not imply brain-specific targeting, but supports meaningful lesion-associated accumulation and retention.

Nanoparticle size is another determinant of brain access: MIIN are ~200 nm, whereas some reports highlight enhanced BBB transfer at smaller sizes under certain injury conditions^90^. In our severe controlled cortical impact paradigm, Evans blue extravasation confirmed pronounced BBB disruption, providing an empirical basis for brain access at this size. We interpret these data to mean that our system can reach and assemble within injured brain parenchyma under sufficiently compromised BBB conditions, although systematic size-optimization remains an important direction to increase brain exposure and reduce peripheral burden.

Beyond neuron-intrinsic limitations, sustained glial dysregulation is a major barrier to CNS regeneration^18,33–37^. MIIN reduced reactive astrocytosis and microglial/macrophage activation (GFAP and Iba-1) and shifted inflammatory mediator profiles toward a more permissive state over both early and later time points. In vitro, MIIN scaffolds attenuated features of chronic microglial activation, including mitochondrial depolarization and oxidative/inflammatory markers^19,55,79–85^. Together, these findings are consistent with a coordinated model in which sustained class IIa HDAC inhibition can influence not only neuronal transcriptional competence but also microglial inflammatory programs, thereby addressing neuron-intrinsic and microenvironmental barriers in parallel. Unbiased proteomic profiling provides an orthogonal view aligned with this concept: injury broadly suppressed proteins linked to axon regeneration, synaptic transmission, and epigenetic regulation, whereas MIIN increased proteins involved in cell–matrix adhesion, tissue remodeling, and anti-apoptotic processes—changes consistent with a shift from an acutely degenerative state toward structural integration and repair.

A central question in CNS repair is whether structural regrowth yields functional synaptic transmission and circuit recovery. Whole-cell patch-clamp recordings showed that our system increased spontaneous excitatory and inhibitory synaptic activity in vitro and restored both sEPSCs and sIPSCs in acute cortical slices after TBI, supporting recovery of functional synaptic transmission rather than solely structural regrowth. Importantly, projection-specific DREADD modulation further linked recovery to a defined motor pathway by demonstrating that M2→CPu activity is required for coordinated motor output and is functionally reinstated after MIIN treatment. Together, these convergent readouts strengthen the interpretation that our system promote meaningful network repair rather than only local tissue preservation.

Our design includes a PANI coating intended to enable an electrical guidance component; however, PANI necessarily alters both electrical properties and surface physicochemistry, including zeta potential and likely protein adsorption. Accordingly, the current dataset does not fully disentangle contributions from electrical permissiveness versus charge- or adhesion-mediated cell–material interactions. Two observations nevertheless argue that activity-dependent processes are integral to the therapeutic effect: MIIN promoted recovery of synaptic transmission in electrophysiological assays, and suppressing neuronal excitability via Kir2.1 expression in M2 markedly attenuated functional recovery after MIIN treatment. These findings support interpreting the PANI layer as an electroactive interface that can support activity-dependent reconnection during repair, while acknowledging that they do not prove bulk conductivity alone as the sole causal feature. Dissecting the relative contributions of electronic/ionic transport, interfacial capacitance, charge-mediated adhesion, and protein-conditioned surface interactions will require orthogonal material controls—such as conductivity-matched/dedoped PANI and charge-matched nonconductive coatings—and will inform the next generation of lesion-assembled electroactive scaffolds.

In summary, our system implement a minimally invasive strategy for CNS repair by combining injury-responsive scaffold assembly, sustained class IIa HDAC inhibition to epigenetically reactivate intrinsic growth capacity, microenvironmental remodeling, and activity-dependent guidance of reconnection. By linking molecular reactivation to synaptic recovery and circuit-level causality, this work supports a generalizable framework for rebuilding disrupted CNS networks following TBI and potentially other conditions marked by vascular disruption, chronic inflammation, and persistent circuit dysfunction.

## Methods

### Ethical regulations

All the animal experiments were performed in accordance with the guidelines evaluated and approved by Institutional Animal Care and Use Committee (IACUC), Fudan University School of Pharmacy (Ethical approval number: 2023-03-YJ-CJ-36).

### Materials

Amino-functionalized gold nanoparticles (5 nm), 5-Hydroxy-L-tryptophan, N-hydroxysuccinimide, 1-Ethyl-3-(3-dimethylaminopropyl)carbodiimide (EDC), aniline, Hoechst 33258, 3-[4,5-dimethylthiazol-2-yl]-2,5-diphenyl tetrazolium bromide (MTT), coumarin-6 (Cou6), MPO, and hydrogen peroxide enzyme were purchased from Sigma-Aldrich (St. Louis, MO, USA). LMK-235, Fmoc-Glu-OH and deschloroclozapine (DCZ) were purchased from MedChemExpress (Monmouth County, NJ, USA). PLGA-COOH (75:25, MW:100k) was kindly provided by East China University of Science and Technology. PLGA-PEG5000-Mal (50:50) and HS-PEG2000-NHS were obtained from Xi’an Ruixi biotech (Xi’an, China). Peptide hydrogel was purchased from Corning (Corning, NY, USA). 1,1’-dioctadecyl-3,3,3’,3’-tetramethylindotricarbocyanine iodide (DiR) was purchased from MeilunBio (Dalian, China). Nuclear and cytoplasmic protein extraction kit, nitric oxide synthase assay kit, ROS assay kit, LPS and enhanced mitochondrial membrane potential detection kits (JC-1) were purchased from Beyotime (Shanghai, China). HDAC Activity Assay Kit was obtained from AAT Bioquest (Pleasanton, CA, USA). ELISA kits were provided by Multi Science (Hangzhou, China). Anti-GFAP primary antibody (ab278054), Anti-NeuN primary antibody (ab177487), Anti-HDAC5 primary antibody (ab55403), Anti-Histone H3 acetylation primary antibody (ab300641), Anti-Histone H3 primary antibody (ab176842), Anti-GAPDH primary antibody (ab181602), goat anti-rabbit IgG Alexa Fluor 488 secondary antibody (ab150077), goat anti-rabbit IgG Alexa Fluor 594 secondary antibody (ab150080), goat anti-rabbit IgG Alexa Fluor 647 secondary antibody (ab150079), goat anti-guinea pig IgG Alexa Fluor 594 secondary antibody (ab150188), goat anti-mouse IgG Alexa Fluor 647 secondary antibody (ab150115) and Rhodamine phalloidin (ab235138) were purchased from Abcam (Cambridge, UK). Anti-Iba1 primary antibody (Cat No: 17198 T) and Anti-PSD95 primary antibody (Cat No: 3450S) were purchased from Cell Signaling Technology (Danvers, MA, USA). Anti-SMI-312 primary antibody (Cat No: 837904) was purchased from Biolegend (San Diego, CA, USA). Anti-VGluT1 primary antibody (Cat No: 135304), Anti-VGAT primary antibody (Cat No: 131004) and Anti-Gephyrin primary antibody (Cat No: 147011) were purchased from Synaptic Systems (Gottingen, NI, Germany). Anti-mouse CD80-FITC antibody (Cat No: 11-0801-82) and Anti-mouse CD206-PE (Cat No: 12-2061-82) were purchased from eBioscience (San Diego, CA, USA). Mouse *Jun* qPCR primer pair (Cat No: 102595), mouse *Fos* qPCR primer pair (Cat No: 38359), mouse *Klf6* qPCR primer pair (Cat No: 103985) and mouse *Actb* qPCR primer pair (Cat No: 10343) were purchased from Sangon Biotech Co., Ltd (Shanghai, China). All the other chemical reagents and solvents were purchased from Sinopharm Chemical Reagent Co., Ltd (Shanghai, China) unless specified.

### Primary neurons, cell lines, and animals

HT22 cells were kindly provided by Prof. Gao Xiaoling of Shanghai Jiaotong University. BV2 cells were purchased from Chinese Academy of Science Cell Bank (Shanghai, China). Primary mouse cortical neurons were extracted from 15-day-old C57BL/6 fetal mice and cultured in Dulbecco’s modified Eagle’s medium (DMEM, high glucose, Gibco) supplemented with 10% FBS, 1% GlutaMAX and 1% antibiotics. The following day, the medium was replaced with Neurobasal (Gibco) containing 10% B27, 1% GlutaMAX and 1% penicillin-streptomycin solution, and a half-medium change was performed every week thereafter. HT22 and BV2 cells were cultured in DMEM (high glucose, Hyclone) containing 10% fetal bovine serum and 1% penicillin-streptomycin solution. Male C57BL/6 mice (6–10 weeks old, 18–22 g) were purchased from SLAC Animal Ltd. (Shanghai, China) and raised in a pathogen-free facility with a 12 h light and dark cycle at 18–23 °C and 40–60% humidity and had free access to food and water. In terms of animal experiment studies, male mice were chosen. Male mice are less likely to die during the establishment of TBI models based on our historical experience. This can reduce accidental death and help to ensure the objectivity of the studies.

### Synthesis and characterization of bis-5HT-Glu-NH_2_

To a mixture of compound 5-HT (2 g, 9.43 mmol, 4.0 eq) and Glu (870 mg, 2.36 mmol, 1.0 eq) in DMF (20 mL) were added HATU (3.58 g, 9.43 mmol, 4.0 eq) and DIEA (2.05 mL, 11.79 mmol, 5.0 eq). The reaction mixture was stirred at 25 °C for 4 h. The mixture was purified by Flash (0.1% FA in water/ACN) to obtain compound bis-5HT-Glu-Fmoc (1.2 g, 74% yield) as a brown solid. A mixture of compound bis-5HT-Glu-Fmoc (1.2 g, 1.75 mmol) in DEA (10 mL) and DCM (30 mL) was stirred at 25 °C for 4 h. The mixture was purified by Flash (0.1% FA in water/ACN) and prep-HPLC (0.1% FA in water/ACN) to obtain compound bis-5HT-NH_2_ (106.3 mg, 13% yield) as a gray solid.

### Preparation and characterization of MIIN

10 mg of PLGA, 1.7 mg of Mal-PEG5000-PLGA, and 1 mg of LMK-235 were dissolved in 2 mL of dichloromethane. The solution was added to 4 mL of 1% sodium cholate solution, emulsified by ultrasound, and dispersed in 20 mL of 0.5% sodium cholate solution. The organic solvent was removed by vacuum rotary evaporation, and the precipitate of LMK-235@Mal-PLGA nanoparticles was obtained by centrifugation at 12,000 g for 40 min. Additionally, coumarin-6, PLGA, and LMK-235 were together dissolved in dichloromethane and prepared coumarin-6-loaded Mal-PLGA nanoparticles via an emulsion solvent evaporation method^68^.

LMK-235@Mal-PLGA nanoparticles were dispersed in 20 mL of deionized water. The pH of the system was adjusted to around 6 using dilute hydrochloric acid. A solution of ethanol containing 0.2 mmol of aniline was slowly added, stirred at room temperature for 30 min. Under the presence of H₂O₂, the heme cofactor (Fe^3+^) of catalase is first oxidized by H_2_O_2_ to generate a highly reactive intermediate. This intermediate can abstract an electron from an aniline molecule, forming an aniline radical cation, which in turn efficiently promotes the in situ polymerization of aniline to yield PANI^60^. Therefore, 500 U of hydrogen peroxide enzyme was added, followed by continued stirring for 10 min. A 20 μmol solution of H_2_O_2_ was slowly added within 30 min, and the mixture was stirred at room temperature for 6 h. The reaction was terminated by adding a sufficient amount of NaHSO_3_. The product was centrifuged at 12,000 g for 40 min, dialyzed using a dialysis bag (MWCO = 3500 Da) for 72 h, and centrifuged to obtain LMK-235@PANI/Mal-PLGA conductive nanoparticles.

Subsequently, a 30% PVP (M.W. 24,000) gel was evenly spread in a glass dish and allowed to stand at room temperature for 1 h. The above conductive nanoparticle solution was slowly added to the gel surface, gently shaking the glass dish to evenly spread the droplets. After standing at room temperature for 2 h, a solution containing 1 mg of SH-PEG2000-NHS was slowly added to completely submerge the spreading surface. After standing at room temperature for 2 h, the spreading surface was repeatedly washed with a pipette, and the wash solution was collected. The product was centrifuged at 12,000 g for 40 min, dialyzed using a dialysis bag (MWCO = 3500 Da) for 24 h, and LMK-235@PANI/NHS Janus NPs were obtained^69,70^.

Finally, the prepared LMK-235@PANI/NHS Janus NPs were dispersed in the aqueous phase, and 0.2 mg of bis-5HT-Glu-NH_2_ was added. The mixture was stirred at room temperature for 2 h, centrifuged at 12,000 g for 40 min, and dialyzed using a dialysis bag (MWCO = 3500 Da) for 24 h to obtain MIIN.

Under identical conditions, control nanoparticles lacking critical features were prepared: (i) LMK-235@PANI nanoparticles by omitting the bis-5HT-Glu-NH_2_ modification; (ii) LMK-235@Bis-5HT/PANI nanoparticles via symmetric modification with bis-5HT-Glu-NH_2_; (iii) Bis-5HT/PANI nanoparticles by excluding LMK-235; and (iv) LMK-235@Bis-5HT Janus nanoparticles by omitting the in-situ polymerization of polyaniline.

MIIN labeled with coumarin-6 were prepared by adding 100 μg of coumarin-6 in the initial steps using the same procedure. The MIIN labeled with gold nanoparticles were prepared by replacing bis-5HT-Glu-NH_2_ with amino-functionalized gold nanoparticles and following the same procedure. The LMK-235@PANI/NHS Janus NPs loaded with coumarin-6 were also prepared using a similar method.

The particle size and zeta potential of MIIN were measured using a dynamic light scattering nanoparticle size and zeta potential analyzer (Zetasizer Nano ZS90, Malvern, UK). TEM images of MIIN were obtained using a TEM (TEM-1400 Plus Electron Microscope, Leica, Germany). SEM images of MIIN were obtained using a SEM (GeminiSEM 300, ZEISS, Germany). The LE and EE of LMK-235 were determined by high-performance liquid chromatography (HPLC, Shimadzu, Japan) at a detector wavelength of 214 nm.

### Electrical conductivity measurement of MIIN

To measure electrical conductivity of MIIN and control nanoparticles, the cylindrical nanoparticles were prepared (cross-sectional area: 3.14 × 1.0 cm^2^; length: 1.0 cm). Then, two-terminal electrical resistance (Ω) was monitored using a digital multimeter (Dawson), and the electrical conductivity was calculated as follows: electrical conductivity (S cm^−1^) = L/(R × A), where R is (electrical resistance, Ω), A is (Atra of cross-sectional (cm^2^) = 3.14 × radius^2^), and L is (length, cm).

### Transmission electron microscopy (TEM)

TEM imaging was carried out using a transmission electron microscope operated at 60 kV. MIIN labeled with gold nanoparticles and other control nanoparticles were deposited onto a carbon-coated electron microscope copper grid at room temperature until the surface liquid was completely evaporated. Then, a 1% (w/v) solution of phosphotungstic acid (pH 7.2) was added and allowed to react for 3 min. The excess liquid was removed, and images were subsequently captured.

### In vitro responsive assembly of MIIN

MIIN were dispersed in 20 mL of PBS containing 125 U/mL MPO and 1 μM H_2_O_2_, followed by slow stirring at 37 °C for 2 hours^91–93^. The appearance changes of the system were observed, and the particle size variation was monitored during the process.

### In vitro release behavior investigation

The in vitro release behavior of MIIN and relevant control formulations was investigated using the equilibrium dialysis method. The release media were buffered saline solutions at pH 7.4 and pH 6.8, with or without 10% FBS. Each drug-loaded formulation was introduced into a pre-swelled dialysis bag (MWCO = 14,000 Da), sealed, and placed in a container containing an appropriate volume of release medium, ensuring sink conditions. The release experiments were conducted at 37 °C under stirring conditions at 120 rpm. Samples were collected at predetermined time points, with an equivalent volume of buffer added after each collection. HPLC was employed to determine the concentration of LMK-235 in the release samples, and cumulative release rates were calculated.

### Characterization of the internal channels of MIIN neural guidance scaffolds

The respective formulations were diluted to an appropriate concentration and dropped onto the surface of silicon slices. After thorough drying under infrared light, the surfaces of samples underwent gold sputter coating using a metal coating technique. Subsequently, SEM observations were conducted using the German ZEISS GeminiSEM 300, and a quantitative assessment of the pore diameter and spatial distribution of neural scaffolds in different regions was performed.

### In vitro cell uptake study

Activated BV2 cells (LPS-pretreated) and injured HT22 cells (OGD-pretreated) were seeded at a density of 5000 cells per well in 96-well plates and cultured for 24 h for attachment. Subsequently, the culture medium was replaced with DMEM containing MIIN labeled with cou6 or the scaffolds formed by them (at a cou6 concentration of 50 ng/mL), and further incubated for 12 h at 37 °C. Then, BV2 cells were washed with PBS, fixed with 4% paraformaldehyde, and stained with 2 μg/mL Hoechst 33258. The cells were analyzed using flow cytometry (CytoFLEX, Beckman, USA) and a spinning disk laser confocal microscopy (SpinSR10, Olympus, Japan).

### Cytotoxicity assay

The cytotoxicity of LMK-235 in HT22 cells was evaluated using the MTT assay. Briefly, HT22 cells were seeded at a density of 5000 cells per well in 96-well plates and cultured for 24 h for attachment. The culture medium was then replaced with DMEM containing varying concentrations of free LMK-235, with LMK-235-free DMEM serving as the control group. After incubation for 24 h, MTT solution (5 mg/mL) was added and incubated with cells for an additional 4 h at 37 °C. Formazan crystals produced by viable cells were dissolved in 200 μL of DMSO, and the absorbance was measured at 490 nm using a microplate reader (Multiskan MK3, Thermo fisher, USA).

### In vitro microglia polarization assay

To investigate the in vitro polarization of microglia, BV2 cells were seeded into 6-well plates and stimulated with 100 ng/mL LPS for 24 h, followed by treatment with PBS (control group), free LMK-235, MIIN, LMK-235@Bis-5HT/PANI NPs scaffolds, Bis-5HT/PANI Janus NPs scaffolds, LMK-235@Bis-5HT Janus NPs scaffolds and MIIN scaffolds (equivalent to a concentration of 11.77 μg/mL LMK-235) for 24 h. Subsequently, cells were collected and washed with PBS. Anti-mouse CD80 and CD206 antibodies were added separately and incubated for 45 min at 4 °C. The cells were then washed with PBS and analyzed using flow cytometry (CytoFLEX, Beckman, USA).

### Detection of iNOS in BV2

To assess the in vitro iNOS levels, BV2 cells were planted in 96-well plates and stimulated with 100 ng/mL LPS for 24 h, followed by treatment with PBS (control group), free LMK-235, MIIN, LMK-235@Bis-5HT/PANI NPs scaffolds, Bis-5HT/PANI Janus NPs scaffolds, LMK-235@Bis-5HT Janus NPs scaffolds and MIIN scaffolds (equivalent to a concentration of 11.77 μg/mL LMK-235) for an additional 24 h. Subsequently, the culture medium was removed, and 100 μL of iNOS detection buffer was added to each well, followed by the addition of 100 μL of detection reaction solution with gentle mixing. The plate was then incubated at 37°C for 20–60 min in a cell culture incubator. The fluorescence intensity was measured using a microplate reader (Multiskan MK3, Thermo fisher, USA) at excitation and emission wavelengths of 495 nm and 515 nm, respectively. Wells without cells served as blank controls.

### Detection of ROS in BV2

To assess intracellular ROS levels in BV2 cells, cells were seeded in a 96-well plate and stimulated with 100 ng/mL LPS for 24 h. Subsequently, BV2 cells were treated with PBS (control group), free LMK-235, MIIN, LMK-235@Bis-5HT/PANI NPs scaffolds, Bis-5HT/PANI Janus NPs scaffolds, LMK-235@Bis-5HT Janus NPs scaffolds and MIIN scaffolds (equivalent to a concentration of 11.77 μg/mL LMK-235) for an additional 24 h. Cells were washed with PBS, and 10 μM DCFH-DA was added for 1 h at 37 °C. After the incubation, the culture medium was removed, and cells were washed with PBS three times. The fluorescence intensity was measured directly using a fluorescence microplate reader with excitation and emission wavelengths set to 488 nm and 525 nm, respectively. Wells without cells served as blank controls.

### Mitochondrial membrane potential assay

To evaluate the mitochondrial membrane potential, the JC-1 staining method was employed. Briefly, BV2 cells were pre-stimulated with 100 ng/mL LPS or left unstimulated and cultured in confocal culture dishes until reaching 60–70% confluence. After aspirating the culture medium, cells were washed once with PBS. Subsequently, 1 mL of cell culture medium and 1 mL of JC-1 staining working solution were added, thoroughly mixed, and incubated at 37 °C for 20 min. Following incubation, the supernatant was removed, and cells were washed twice with JC-1 staining buffer. Then, 2 mL of cell culture medium was added, and cells were observed using a confocal laser microscope. ImageJ was utilized to assess the fluorescence intensity ratio in each image for determining the mitochondrial membrane potential level.

### 3D culture of neurons

Prior to use, Corning® PuraMatrix™ peptide hydrogel was sonicated in a water bath for 30 min. HT22 cells or mouse primary cortical neurons were then collected by low-speed centrifugation, washed with sterile 10% sucrose solution, and resuspended in 10% sucrose solution to the desired concentration. The resuspended cells were rapidly mixed with an equal volume of 1% Corning® PuraMatrix™ peptide hydrogel. The hydrogel/cell mixture was then quickly transferred to confocal dishes using a pipette within 2 min. Finally, cell culture medium was gently added to the hydrogel/cell mixture. After 5–10 min, the medium was replaced, and two additional medium changes were performed within the following 30 min.

### In vitro OGD/R model

BV2 cells and HT22 cells were planted in a 96-well plate at a density of 2 × 10^4^ cells per well and cultured in a 37 °C, 5% CO_2_ incubator for 24 h. Subsequently, the regular DMEM medium was replaced with glucose-free and serum-free DMEM medium. The cells were then transferred to a three-gas incubator with 92% N_2_, 3% O_2_, and 5% CO_2_ for 10 h, followed by reoxygenation under normal culture conditions at 37 °C, 5% CO_2_ for 2 h^94^.

### Western blotting analysis

Cell samples were lysed in RIPA buffer supplemented with a protease and phosphatase inhibitor cocktail. Protein quantification was performed using the standard BCA assay. For Western blot analysis, protein samples were mixed with loading buffer (5x) containing a reducing agent, heated at 95 °C for 5 min, and then separated using 12% SDS-PAGE electrophoresis (Invitrogen). After electrophoresis, proteins were transferred to nitrocellulose filter (NC) membranes and blocked with a 5% albumin TBST solution at room temperature for 1 h. The blots were incubated with primary antibodies overnight at 4 °C, followed by incubation with horseradish peroxidase (HRP)-conjugated anti-rabbit IgG for 1 h at room temperature. Detection was performed using Pierce ECL Western Blotting Substrate (Thermo Fisher Scientific, IL).

### Quantitative reverse transcriptase PCR (qPCR) analysis

Trizol reagent (Invitrogen) was used to extract RNA from brain tissues or cells according to the manufacturer’s instructions. To detect the target gene, RNA reverse transcription kit (Bio-Rad) was used to reverse the RNA chain into cDNA. cDNA (800 ng) was amplified using Universal SYBR Green Fast qPCR Mix with CFX Manager software. The expression of each gene was normalized to the expression of β-actin. All the primers for the qPCR reactions are listed in Supplementary Table 6.

### ELISA assay

To explore alterations in the microenvironment during the acute and chronic phases following TBI, an enzyme-linked immunosorbent assay (ELISA) was conducted to quantify pro-inflammatory cytokines IL-1β, IL-6, TNF-α, and iNOS, as well as anti-inflammatory cytokines IL-4, IL-10, TGF-β, and Arg-1 in the injured brain tissue at 7 and 28 days post-injury. All specimens were processed and analyzed following the manufacturer’s protocol.

### Controlled cortical impact (CCI) mouse model

The CCI model was established according to previous protocols with some modifications^15,17^. In brief, animals were intraperitoneally anesthetized with sodium pentobarbital (50 mg/kg, IP). The bregma and the right parietal bone of the mice were exposed, and a 4 mm-diameter burr hole was drilled 1 mm posterior to the bregma along the right side of the midline. The bone flap was removed to fully expose the dura mater. The injury model was induced by impacting the dura with a 3 mm-diameter impactor tip. The impact velocity was set at 3.5 m/s, with an dwell time of 400 ms and a injury depths of 1.0 mm (severe injury). Following the impact, blood was removed using a cotton swab, taking care not to touch the injured area. Mice were placed on a warming pad to maintain body temperature. Once hemostasis was achieved, the wound was sutured and disinfected. Subsequently, the animals were returned to a clean cage to recover overnight on the warming pad. Mice were closely monitored for postoperative recovery, signs and symptoms of pain and distress or other adverse effects after surgery. Buprenorphine (0.05 mg/kg, IP) was administered within 24 h post-surgery. Sham-operated mice underwent the same procedures without the impact procedure^95,96^.

### The brain targeting and lesion responsiveness of MIIN study

Healthy C57BL/6 mice aged 6–8 weeks were used to establish the TBI animal evaluation model using the CCI model. MIIN loaded with the fluorescent dye DiR, along with their control formulations, were intravenously injected into the tail vein of the mice. The DiR injection dose was 1 mg/kg. At 0, 1, 2, 4, 8, 12, 24, 36, and 48 h post-injection, mice were anesthetized with isoflurane and placed in an IVIS imaging system (excitation wavelength: 748 nm, emission wavelength: 780 nm) to evaluate the brain targeting and lesion-responsive accumulation capabilities of MIIN.

### Tissue processing

MIIN containing LMK-235, along with control formulations, were intravenously injected into the tail vein of the mice 6 hours post-injury. The LMK-235 injection dose was 1.5 mg/kg. After perfusion with physiological saline and 4% paraformaldehyde through the heart for 15 min at 7 and 28 days post-treatment, the brain was collected and fixed in 4% paraformaldehyde for 48 h at 4 °C. Subsequently, the tissues were dehydrated gradually using 15% and 30% sucrose solutions. For immunofluorescence staining, brains were embedded in OCT (Sakura, Torrance, CA, USA), and 10 µm thick sections were sliced using a cryostat (Leica, CM3050S).

### Immunohistochemistry (IHC) analysis

IHC staining was performed on paraformaldehyde-fixed cells or frozen brain tissue sections. The paraformaldehyde-fixed cells or frozen sections incubated in Immunol Staining Blocking Buffer (Beyotime Biotechnology Co., Ltd, Nantong, China) for 1 h at room temperature for permeabilization and blocking. Subsequently, the cells or sections were incubated with primary antibodies or fluorescently labeled phalloidin, followed by incubation with secondary antibodies, such as Alexa Fluor 488, Alexa Fluor 594, or Alexa Fluor 647-labeled goat anti-rabbit IgG antibodies. Finally, the cells or sections were stained with Hoechst 33258 and observed under a spinning disk laser confocal microscopy (SpinSR10, Olympus, Japan). The analysis of positive signals in images was performed by Image J software.

### Morris water maze assay

MIIN and other control formulations (LMK-235 at a dose of 1.5 mg/kg) were administered via tail vein injection 6 h post-injury. The Morris water maze was assessed 28 days post-treatment. The water maze was filled with white dyed water, and a platform was fixed 3 cm beneath the water surface. Mice underwent training to reach the platform from random starting positions along the maze border, with each quadrant tested randomly. Learning trials were conducted four times a day, with 30-min intervals between each trial, for seven consecutive days before the assay. During training, if the experimental mouse successfully found and reached the platform within 60 s, it was allowed to remain on the platform for 20 s. If it failed to find the platform, the tester would gently guide it to the platform, where it stayed for 20 s. To assess neurological function, each trained mouse was allowed to swim freely for 60 s from a starting location far away from the platform 28 days after CCI model establishment. The paths, latency, and swimming distance were recorded using a computerized video tracking system.

### Behavior tests

MIIN and other control formulations (LMK-235 at a dose of 1.5 mg/kg) were administered via tail vein injection 6 h post-injury. The behavior tests were assessed 28 days post-treatment. In behavior tests, mice underwent a 5-day training period prior to tests. The adhesive test was conducted three times daily, while the forelimb placing test was conducted once daily. All tests were carried out on day 28 post-CCI.

For the rotarod test, the Rotor-Rod motor function system (San Diego Instruments, San Diego, CA, USA) was utilized. Mice were placed on rotating cylinders, which linearly accelerated from 0 to 50 rpm over a 5-min period. The latency to fall was recorded as the average of five separate runs for each animal^17^.

For the adhesive test, a 10×10 mm^2^ sticker was gently affixed to the paralyzed forepaw using forceps. Subsequently, the mouse was returned to its home cage. The time duration from the initiation to the point when the mouse first made contact with the sticker was recorded as the time to touch. The duration from the initiation to the point when the mouse successfully removed the sticker was recorded as the time to remove.

For the forelimb placing test, the mouse was restrained by holding its back to suspend the limbs. The beard was gently touched to the edge of the table corner to test the activity of the forelimb on the ipsilateral side. This maneuver reflects varying degrees of impairment following brain injury. Mice underwent this test 10 times, and the score was calculated as the percentage of instances where the forelimbs made contact with the corner edge of the table.

### Measurement of neurological scores

MIIN and other control formulations (LMK-235 at a dose of 1.5 mg/kg) were administered via tail vein injection 6 h post-injury. Neurological scores were evaluated 28 days post-administration using a 6-point scale: 0, representing a normal and active condition; 1, indicating incomplete extension of the right forepaw; 2, reflecting circling to the right side; 3, denoting an inability to stand up and falling to the right side; 4, indicating no spontaneous movement; and 5, representing no response to stimulation or mortality.

### Open field test

Mice, housed in their homecages, were transferred to the experimental room and acclimated for a minimum of 0.5 h prior to the experiments. Subsequently, each mouse wsa placed in the center of a square Plexiglas open field apparatus (40 × 40 × 35 cm). Movements were recorded (UGO BASILE srl, Italy) for 20 min. Between each test, the open-field arena was thoroughly cleaned with 70% ethanol. The ANY-maze software (UGO BASILE srl, Italy) was utilized to quantify the total distance traveled in the zone by the mice.

### Proteomic analysis

Brain tissue samples were collected from different experimental groups of mice in the brain injury region, and proteins were extracted from these samples for mass spectrometry analysis. A two-tailed Student’s *t*-test was used to conduct differential analysis of protein expression between the two groups being compared. Upregulation or downregulation of proteins was determined based on a log2 fold change (FC) threshold of 1.5 and a significance level of *P* < 0.05, meaning that only proteins with expression changes exceeding 1.5-fold and showing significant differences would be designated as differentially expressed proteins (DEPs). For appropriate differential results, we utilized volcano plots and clustering heat maps for visualization of the differential proteins. GO enrichment analysis of the DEPs was performed using the GOATOOLS, applying a significance threshold of *P* < 0.05 for the selection of significantly enriched terms.

### Stereotaxic surgery

After anesthesia with 1% (w/v) sodium pentobarbital (10 ml per kg body weight of mice), a craniotomy was carried out for stereotaxic injection. Mice were securely positioned in a state-of-the-art stereotactic frame with non-rupture ear bars (RWD Life Science, Shenzhen, China) to minimize discomfort. AAV-containing solutions were loaded into the tips of pipettes and injected at the following coordinates (anteroposterior to bregma, AP; lateral to the midline, ML; below the bregma, DV; in mm): M2: Site 1. AP, +2.2, ML, -1.0, DV, -1.8; M2: Site 2. AP, +0.8, ML, -0.9, DV, -1.6; CPu: AP, +0.3, ML, -2.5, DV -3.35. Virus-containing solutions were injected unilaterally into the M2: Site 1. (0.25 μl), M2: Site 2. (0.2 μl), CPu (0.4 μl) at a rate of 0.1 μl/min. Waited for 10 min after the infusion, then withdrew the needle slowly to avoid spilling over.

### Viral vectors

The following viral vectors were procured from BrainVTA Co., Ltd (Wuhan, China) for the current study: rAAV-EF1α-DIO-hM4D(Gi)-mCherry-WPREs (Serotype 2/9), rAAV-EF1α-DIO-hM3D(Gq)-mCherry-WPREs (Serotype 2/9), rAAV-EF1α-DIO-ChR2-E123T/T159C-mCherry (Serotype 2/9), rAAV-EF1α-DIO-Kir2.1-P2A-EGFP (Serotype 2/9) and Retro-AAV-hSyn-EGFP-Cre (Serotype 2/retro). All viral vectors were stored in aliquots at −80 °C until further use. Unless otherwise specified, the viral titers of AAVs for injection exceeded 10^12^ viral particles per mL.

### DCZ administration

For the DREADDs, groups of mice were administered with DCZ (0.1 mg/kg i.p.) 30 min before each behavior test 2.

### Patch clamp electrophysiology in primary cortical neuron

The whole-cell configuration of the patch clamp technique was used to record membrane currents (voltage clamp). Cells were bathed in extracellular fluid containing (in mM): 150 NaCl, 5 KCl, 2 CaCl_2_, 1 MgCl_2_, 10 glucose, and 10 HEPES, pH 7.4 adjusted with Tris-base. The pipette solution (internal) contained (in mM): 120 KCl, 30 NaCl, 1 MgCl_2_, 0.5 CaCl_2_, 5 EGTA, 2 MgATP, 0.3 Na_2_GTP, and 10 HEPES, pH 7.4 adjusted with Tris-base. Cells were held at –60 mV in whole-cell modenand currents were digitized at 10 kHz and filtered at 2 kHz. All data were acquired in the voltage-clamp mode using Axon Digidata 1550B and MultiClamp 700B amplifier^97^.

### Patch clamp electrophysiology in brain slices

MIIN containing LMK-235, along with control formulations, were intravenously injected into the tail vein of the mice 6 hours post-injury. The LMK-235 injection dose was 1.5 mg/kg. Whole-cell recording using patch-clamp was employed to record neuronal electrical signals at 28 days post-treatment. In brief, mice were intraperitoneally anesthetized with sodium pentobarbital. The brain was rapidly extracted after perfusion through the heart with cold slicing solution and immersed in the same solution. Coronal slices of the hippocampus (300 μm) were obtained, with four slices per mouse. The brain slices were initially incubated in a chamber with oxygenated artificial cerebrospinal fluid (ACSF) at 34 °C for 45 min, followed by an additional 30-min incubation at room temperature. After the incubation, brain slices were placed on the stage of a microscope, and oxygenated fresh ACSF was continuously perfused at a rate of 2-3 mL/min. The field of view was then shifted to the motor cortex adjacent to the injury site, and target motor neurons were selected. Glass microelectrodes were controlled by micromanipulators to achieve a whole-cell recording by forming a seal with the neuron membrane and applying negative pressure to rupture the membrane. Once the signals stabilized, spontaneous excitatory postsynaptic current (sEPSC) and spontaneous inhibitory postsynaptic current (sIPSC) of neurons were recorded using Clampex 11.2 software. The recorded neuronal electrical signals were processed and analyzed using MiniAnalysis 6.0.3 software^98^.

### Optically evoked EPSCs

Optically evoked EPSCs (oEPSCs) were induced to elicit synaptic responses in the CPu by optogenetic photostimulation of M2 axons or those in M2-CPu projectors. This was accomplished by illuminating each brain slice every 20 s with 5-ms blue-light pulses. To ensure that only monosynaptic activities were recorded in the EPSC experiments, photostimulation intensities were meticulously adjusted to achieve 30–50% of the maximum synaptic response. Optical stimulation of ChR2-expressing axons utilized a 473 nm peak wavelength blue collimated LED (Lumen Dynamics Group Inc, USA), connected to an Axon 200B amplifier. The brain slice in the recording chamber was illuminated through a 40× water-immersion objective lens (Olympus LUMPLFLN 40XW, Japan), with the intensity and duration of photostimulation regulated by the stimulator and the Digidata 1550 and pClamp 10.5 software, respectively.

For recording oEPSCs, recording pipettes (3–5 MΩ) were filled with a solution comprising 132.5 mM Cs-gluconate, 17.5 mM CsCl, 2 mM MgCl_2_, 0.5 mM EGTA, 10 mM HEPES, 2 mM Na_3_-ATP, and 5 mM QX-314. The solution’s pH was adjusted to 7.3 using CsOH, with an osmolarity between 280 and 290 mOsm. To assess the impact of DCZ on oEPSCs, 0.05 µM of DCZ was added to the ACSF following the recording of oEPSCs from the same cell in standard ACSF. During these recordings, the patched CPu neurons were voltage-clamped at –70 mV for oEPSCs.

### Statistics and reproducibility

All the studies were replicated three times and the results were consistent across independent experimental runs. Aside from the evaluation of animal neurological function, researchers were not blinded during the allocation and assessment of the remaining experiments and results. In the evaluation of fluorescence intensity in tissues or cells, microscopes were used to automatically capture images, and random fields of view were selected according to strict predetermined selection protocols. Criteria for skipping views or discarding data were established and validated before data collection. Subsequently, bulk data analysis was performed using image analysis software based on predefined pixel intensity thresholds. Other experiments were not randomized. The sample size for the nanoparticle studies was selected based on prior published research^99–101^. The sample size for the IVIS studies was determined following published works^15,16,101,102^. Similarly, the sample sizes for other cellular and animal studies were also based on previously published literature^103–106^. All the statistical analyses were performed using GraphPad Prism 9 software. Data are generally presented as mean ± SD, with figure legends specifying the statistical tests used: two-tailed Student’s *t*-test for comparisons between two groups, one-way ANOVA using Tukey post hoc test for comparison among multiple groups, and two-way ANOVA followed by Tukey post hoc test for multiple groups involving two variables. Individual data points and distributions were included in the plots and data distribution was assumed to be normal, but this was not formally tested.

### Reporting summary

Further information on research design is available in the Nature Portfolio Reporting Summary linked to this article.

## Supplementary information

Supplementary Information

Description of Additional Supplementary Files

Supplementary Movie 1

Reporting Summary

Transparent Peer Review file

## Source data

Source data

## Data Availability

The proteomics raw data generated in this study have been deposited in the ProteomeXchange Consortium via the iProX partner repository under accession code PXD051033 [https://www.iprox.cn/page/home.html]. Other data generated in this study are provided in the Supplementary Information/Source Data file. Any additional requests for information can be directed to, and will be fulfilled by, the corresponding authors. Source data are provided with this paper.
