# Supplementary material for: Self-assembling scaffolds epigenetically reactivate and electroactively guide neuronal regeneration to restore central neural circuits
Source: Nat Commun. 2026 May 4;17:5987. doi: 10.1038/s41467-026-72397-6 (PMC13347008; doi:10.1038/s41467-026-72397-6)
Supplement: Supplementary file 1 — Supplementary Information [file 41467_2026_72397_MOESM1_ESM.pdf]

Self-assembling scaffolds epigenetically reactivate and electroactively guide neuronal regeneration to restore central neural circuits

Shiqiang Tong[1,2†], Shuai Ye[3,4†], Fenfen Ma[1,5], Xiaoying Xie[1], Yinzhe Sun[1], Chuchu Ma[1], Tiantian Shi[3], Zheng Cheng[1], Chang Li[1], Weili Han[6], Laozhi Xie[1], Songlei Zhou[1], Jianing Gong[1], Chen Huang[7], Yukun Huang[8], Gan Jiang[8], Xiaolin Liu[5], Bing Li[4], Feng Zeng[9], Jingru Gong[5], Zhihua Wang[10*], Xiaoling Gao[8*], Qiyong Mei[9*], Wei-Guang Li[3,4,11*], Jun Chen[1*]

[1]Shanghai Pudong Hospital & Department of Pharmaceutics, School of Pharmacy, Key Laboratory of Smart Drug Delivery, Ministry of Education, State Key Laboratory of Advanced Drug Formulations for Overcoming Delivery Barriers, Fudan University; Shanghai, 201203, China.
[2]NMPA Key Laboratory for Research and Evaluation of Pharmaceutical Preparations and Excipients, State Key Laboratory of Natural Medicines, Department of Pharmaceutics, China Pharmaceutical University; Nanjing, 210009, China.
[3]Department of Rehabilitation Medicine, Huashan Hospital, Center for Clinical Neuro-AI, Institute for Translational Brain Research, State Key Laboratory of Medical Neurobiology and Ministry of Education Frontiers Center for Brain Science, Fudan University; Shanghai, 200032, China.
[4]Research Center for Clinical Medicine, Jinshan Hospital Affiliated to Fudan University; Shanghai, 201508, China.
[5]Department of Pharmacy, Shanghai Pudong Hospital, Fudan University Pudong Medical Center; Shanghai, 201399, China.
[6]School of Medicine, Shanghai University; Shanghai, 200444, China.
[7]Basic Medicine Experimental Teaching Center, Shanghai Jiao Tong University School of Medicine; Shanghai, 200025, China.
[8]Department of Pharmacology and Chemical Biology, State Key Laboratory of Oncogenes and Related Genes, Shanghai Universities Collaborative Innovation Center for Translational Medicine, Shanghai Jiao Tong University School of Medicine; Shanghai, 200025, China.
[9]Department of Neurosurgery, Changzheng Hospital, Naval Medical University; Shanghai, 200003, China.
[10]Department of Emergency, Shanghai Pudong Hospital, Fudan University Pudong Medical Center; Shanghai, 201399, China.
[11]Ministry of Education-Shanghai Key Laboratory for Children's Environmental Health, Xinhua Hospital, Shanghai Jiao Tong University School of Medicine; Shanghai 200092, China.

*Corresponding author. Email: chenjun@fudan.edu.cn (J.C.); liwg@fudan.edu.cn (W.-G.L.); meiqiyong@smmu.edu.cn (Q.M.); shellygao1@sjtu.edu.cn (X.G.); wzhys@163.com (Z.W.)

†These authors contributed equally: Shiqiang Tong, Shuai Ye.

Supplementary Information

Supplementary Tab. 1 Meaning of the abbreviations for formulation components

Abbreviation	Meaning
**LMK-235**	The small-molecule drug LMK-235
**Bis-5HT**	A ligand containing two 5-HT ends
**PANI**	Polyaniline conductive layer
**Janus**	Bis-5HT groups are distributed on the semi-surface of the nanoparticles
**NPs**	Nanoparticles

Supplementary Tab. 2 The composition of the different control nanoparticles

Nanoparticles	Does it contain LMK-235?	Is it modified by Bis-5HT?	Is the Bis-5HT predominantly distributed on the semi-surface?	Is it coated with a PANI conductive layer?
**LMK-235@PANI NPs**	Yes	No	No	Yes
**LMK-235@Bis-5HT/PANI NPs**	Yes	Yes	No	Yes
**Bis-5HT/PANI Janus NPs**	No	Yes	Yes	Yes
**LMK-235@Bis-5HT Janus NPs**	Yes	Yes	Yes	No
**MIIN**	Yes	Yes	Yes	Yes

Supplementary Tab. 3 The detailed information on the intermediates and the final nanocarrier.

Nanoparticles	Size (nm)	PDI	*Zeta* potential (mV)	EE (%)	LE (%)	Yield (%)
**LMK-235@PLGA-COOH NPs**	152.0	0.073	-44.7	78.84	8.26	81.58
**LMK-235@PLGA-COOH/Mal NPs**	181.5	0.173	-35	77.46	8.17	81.03
**LMK-235@PLGA-COOH/Mal/PANI NPs**	194.5	0.142	-5.6	73.75	7.05	34.49
**LMK-235@PLGA-COOH/Mal/PANI-NHS NPs**	198.4	0.126	-5.3	71.39	5.93	38.43
**MIIN**	205.4	0.157	-5.7	70.56	5.88	38.06

Supplementary Tab. 4 The detailed information on the control and the final nanocarrier.

Nanoparticles	Size (nm)	PDI	*Zeta* potential (mV)	EE (%)	LE (%)
**LMK-235@PANI NPs**	160.9	0.115	-5.5	74.12	7.11
**LMK-235@Bis-5HT/PANI NPs**	208.7	0.126	-6.2	71.18	5.72
**Bis-5HT/PANI Janus NPs**	201.3	0.155	-5.9	0	0
**LMK-235@Bis-5HT Janus NPs**	186.6	0.146	-36.9	75.71	7.09
**MIIN**	205.4	0.157	-5.7	70.56	5.88

Supplementary Tab. 5 the pore size distribution of MIIN scaffolds

ID	Pore area (μm^2)	Pore diameter (μm)	Channel length (μm)
0	1286.642304	20.23736523	65.73560937
1	144.215696	6.77534375	77.543125
2	487.042112	12.45111719	55.45821484
3	107.55224	5.851063477	26.36438867
4	3.306366	1.025889404	35.35620703
5	23.70451	2.746885498	25.35435352
6	4.0067585	1.129332031	65.80741406
7	21.841742	2.636748535	75.34374219
8	335.868	10.33973438	74.08741406
9	1367.996928	20.86736523	38.34100781
10	70.725944	4.744762207	79.6368125
11	23.095926	2.711394775	57.95059375
12	232.304672	8.599120117	66.73061719
13	3848.701184	35.00113672	50.10132813
14	0.276372844	0.296601105	47.83521484
15	64.178792	4.519816895	86.82354688
16	6.9800355	1.490575195	36.19092188
17	903.11232	16.95492773	29.80460156
18	364.091904	10.76541016	61.56683203
19	10.03697	1.787419067	42.42642188
20	21.806316	2.634609375	59.62224219
21	10.926251	1.864921875	30.08030664
22	952.691072	17.41410352	33.19417969
23	2.1109635	0.819719788	57.31508984
24	27.938596	2.982135254	37.02789453
25	504.664864	12.67437598	40.68934766
26	140.090432	6.677736816	49.47885156
27	13.049136	2.038055176	51.35495313
28	1168.063872	19.2822793	31.83699414
29	123.438384	6.268305664	38.37060547
30	247.467648	8.875325195	39.49689062
31	884.479296	16.77910938	62.24355078
32	110.839072	5.93979541	63.47600781
33	591.823872	13.7252832	22.86721094
34	0.69619825	0.470751312	24.80974414
35	0.184735766	0.242493759	54.41383203
36	35.198796	3.347256348	35.13827734
37	69.237336	4.694563965	57.09550781

Supplementary Tab. 6 Primer sequences for real-time quantitative PCR

Gene	Forward	Reverse
Jun	5'-GAACTCGGACCTTCTCACG-3'	5'-TGGGGCACAAGAACTGG-3'
Fos	5'-CTCCTCCGACTCCCTGA-3'	5'-TTGCCTTCTCTGACTGCTC-3'
Klf6	5'-ACTTTCACCCACGACCAA-3'	5'-CACAGCCCCATAGTTGAGA-3'
Actb	5'-TCTTTGCAGCTCCTTCGT-3'	5'-GACCCATTCCCACCATC-3'

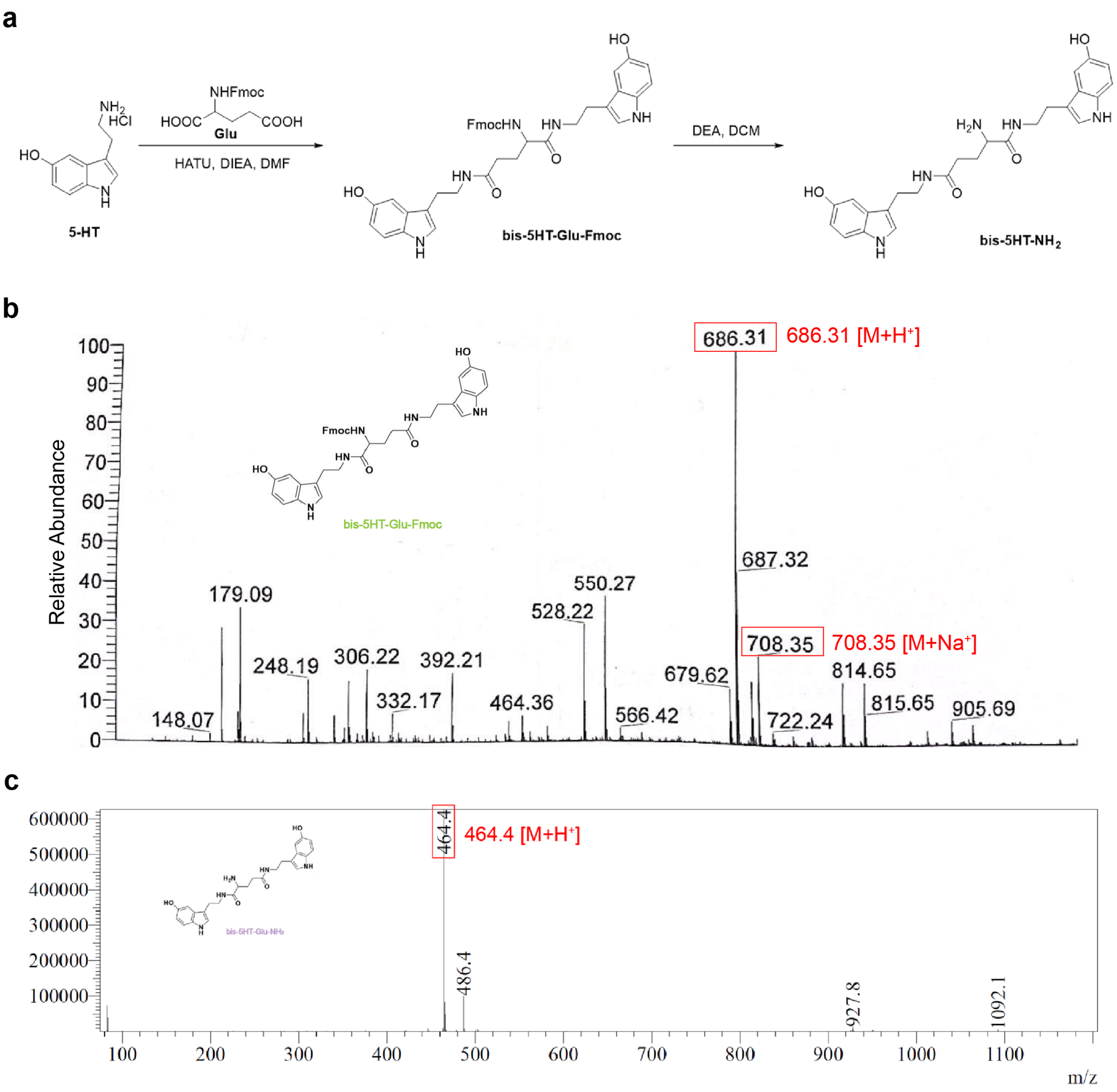

Supplementary Fig. 1 Synthesis and characterization of bis-5HT-Glu-NH₂. a Synthetic route of bis-5HT-NH₂. **b** LC-MS spectrum of intermediates bis-5HT-Glu-Fmoc. **c** LC-MS spectrum of final compounds bis-5HT-Glu-NH₂. The experiments were repeated three times independently.

<Chromatogram>

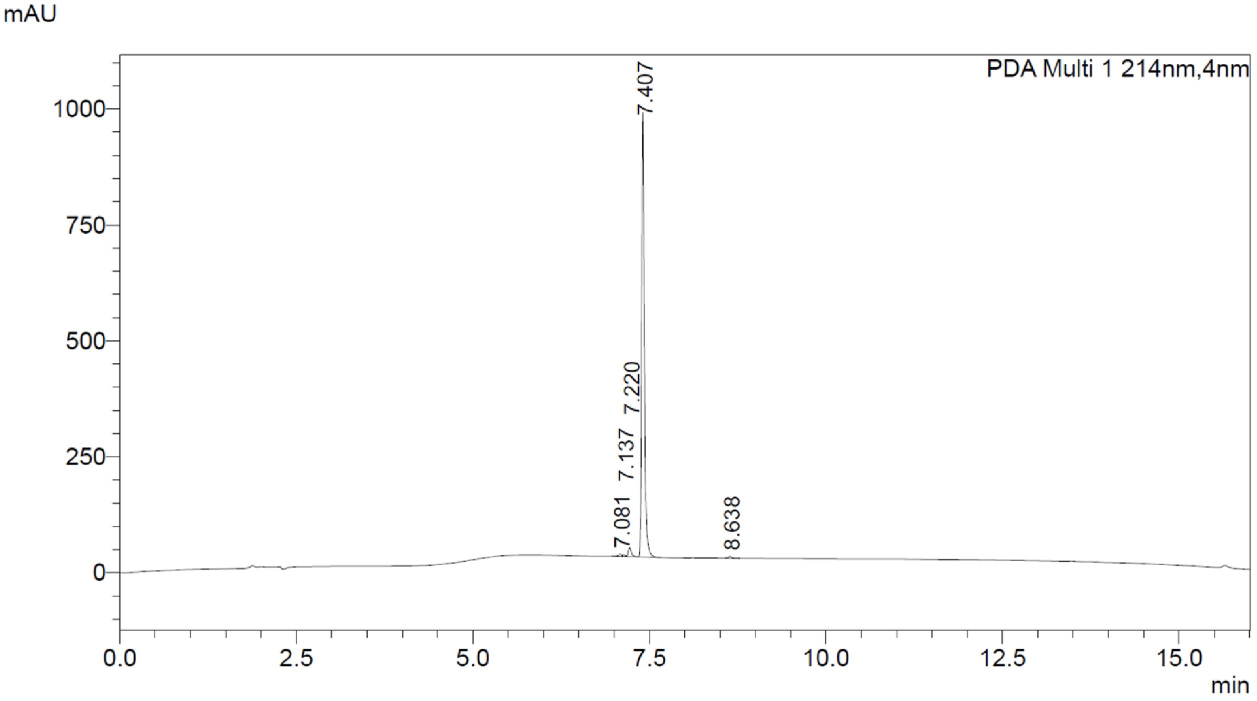

<Peak Table>

PDA Ch1 214nm

Peak#	Ret. Time	USP Width	Height	Area	S/N	Area%
1	7.081	0.128	4791	17428	5.46	0.658
2	7.137	0.192	3520	10224	4.01	0.386
3	7.220	0.087	19699	58662	22.46	2.213
4	7.407	0.076	958499	2551130	1092.73	96.257
5	8.638	0.091	4103	12876	4.68	0.486
Total				2650320		100.000

Supplementary Fig. 2 HPLC purity analysis of bis-5HT-Glu-NH_2. The experiments were repeated three times independently.

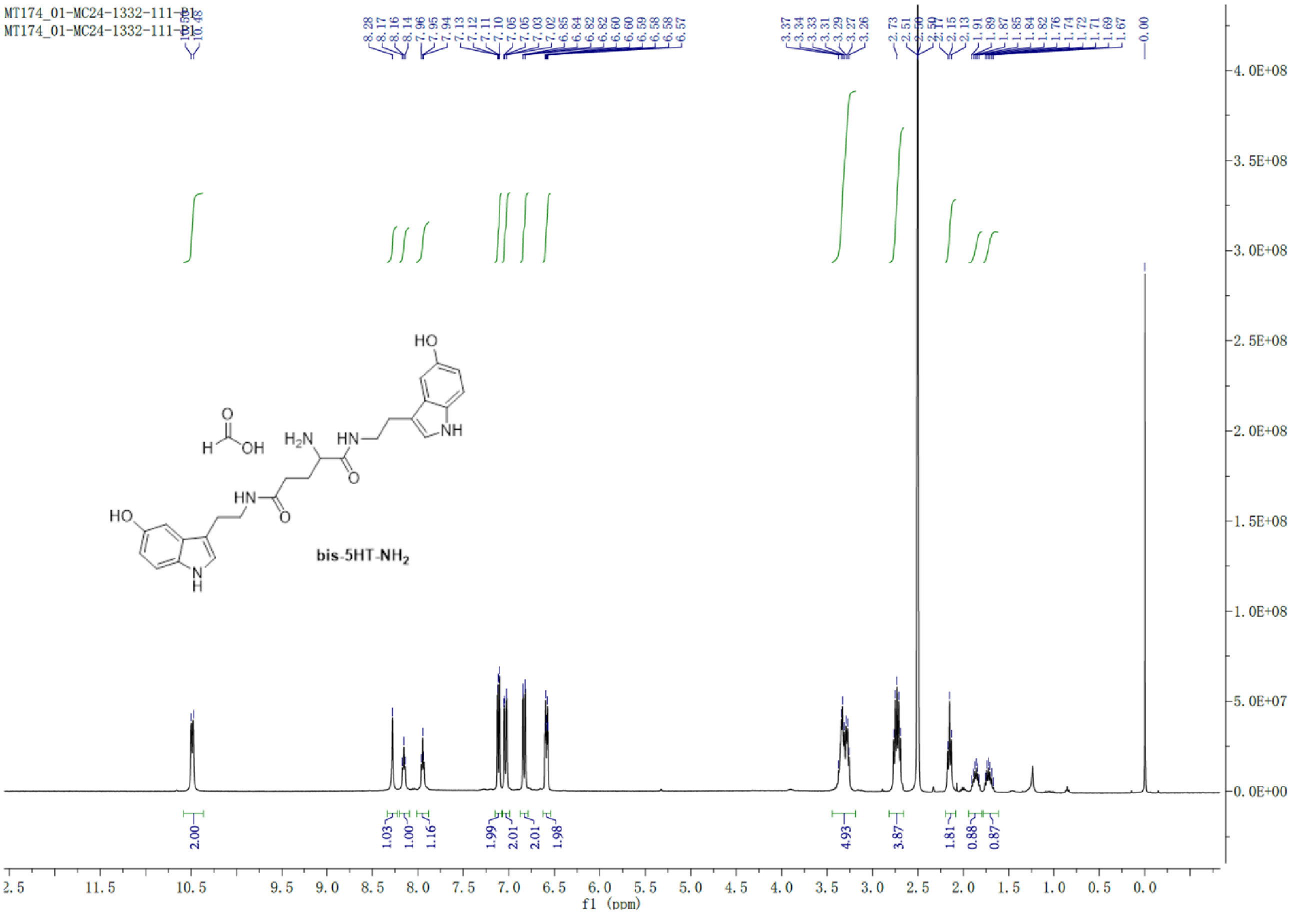

Supplementary Fig. 3 ^{1}H NMR purity analysis of bis-5HT-Glu-NH_2. The experiments were repeated three times independently.

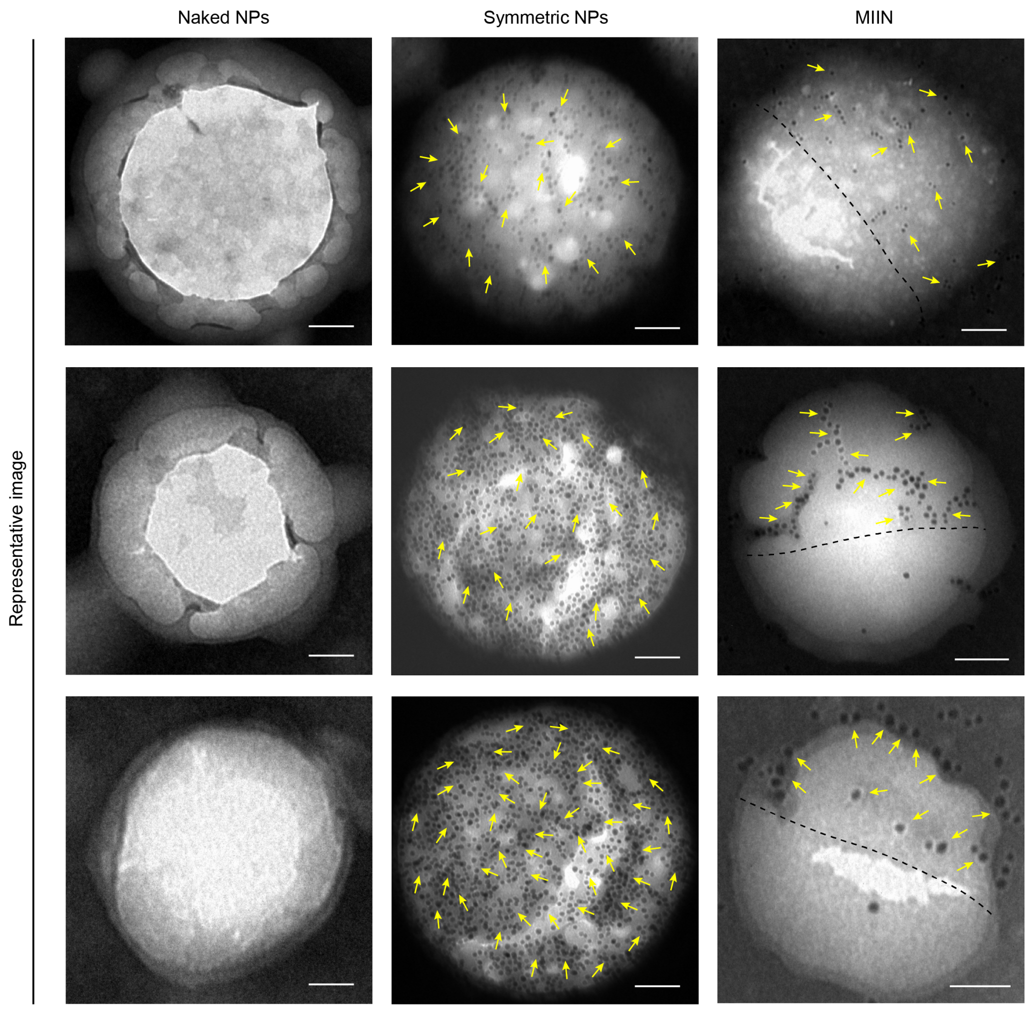

Supplementary Fig. 4 TEM of MIIN labeled with gold nanoparticles from multiple angles of several nanoparticles. The yellow arrows indicate the gold nanoparticles conjugated to the surface of the formulation. Scale, 50 nm. The experiments were repeated three times independently.

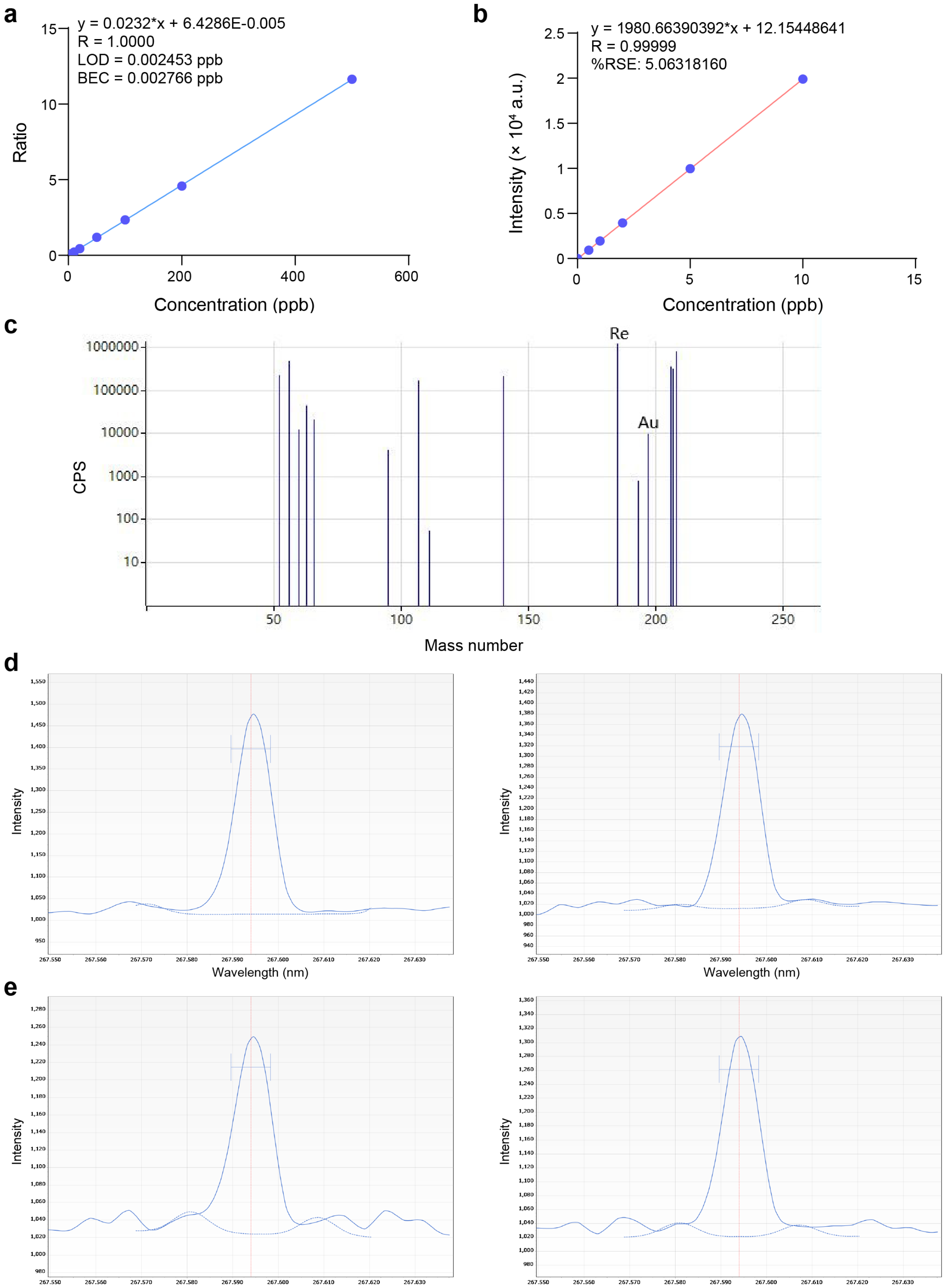

Supplementary Fig. 5 Quantitative determination of the gold content using ICP-OES/MS elemental analysis. a, b Standard calibration curve for gold element determination by (**a**) ICP-MS and (**b**) ICP-OES. **c** Mass spectrum of gold element in control nanoparticles determined by ICP-MS. The experiments were repeated three times independently. **d, e** Peak profile of gold element in (**d**) symmetrical nanoparticles and (**e**) MIIN determined by ICP-OES. The experiments were repeated three times independently.

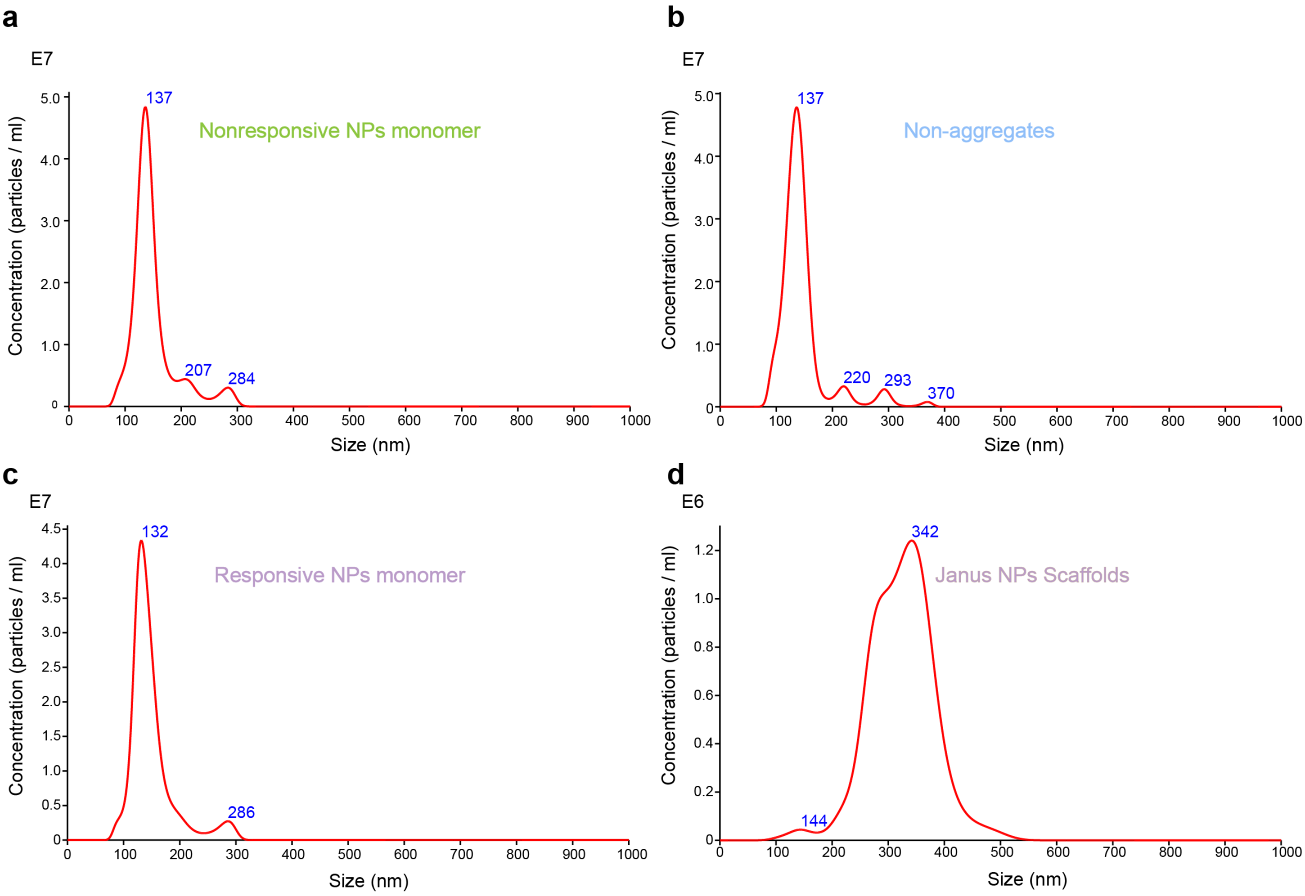

Supplementary Fig. 6 Quantitative determination of the gold content using ICP-OES/MS elemental analysis. a, b NTA analysis of non-responsive nanoparticles in (**a**) PBS or in (**b**) medium containing MPO and H_2O_2. The experiments were repeated three times independently. **c, d** NTA analysis of responsive nanoparticles in (**c**) PBS or in (**d**) medium containing MPO and H_2O_2. The experiments were repeated three times independently.

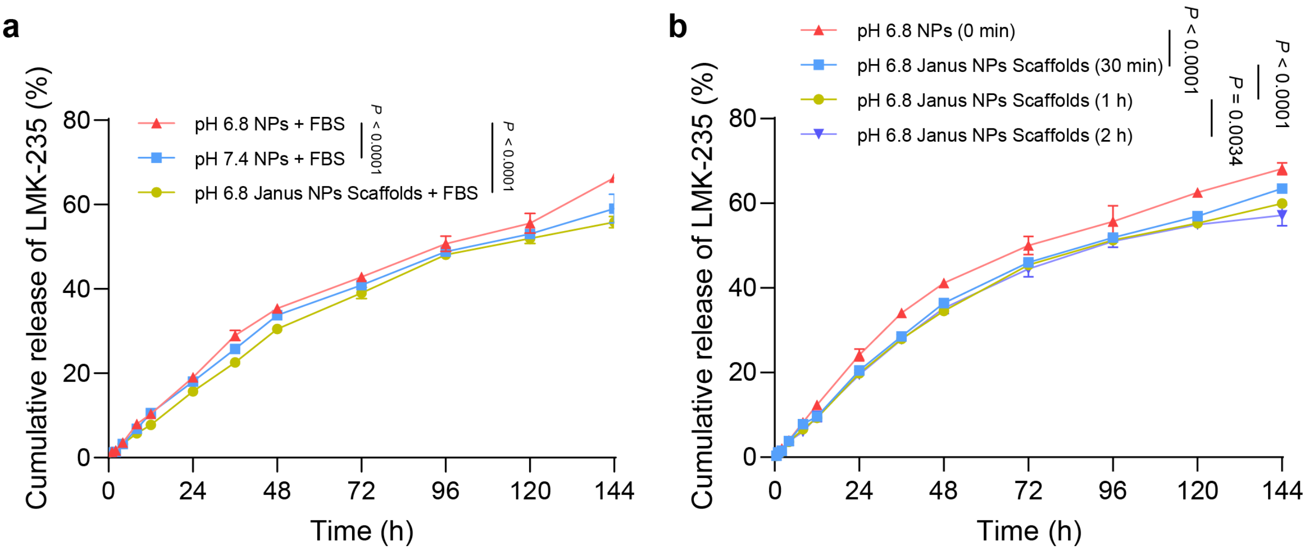

Supplementary Fig. 7 The release profile of LMK-235 from MIIN or scaffolds. a The release profile of LMK-235 from MIIN before and after scaffolds formation in PBS (pH 6.8 and pH 7.4) at

37 °C with 10% FBS (n = 3). **b** The release profile of LMK-235 from scaffolds with different degrees of assembly in PBS (pH 6.8 and pH 7.4) at 37 °C (n = 3). The data are presented as the mean ± SD of three independent biological replicates, along with the corresponding P values. Statistical analyses are performed using two-way ANOVA with Tukey's post hoc test.

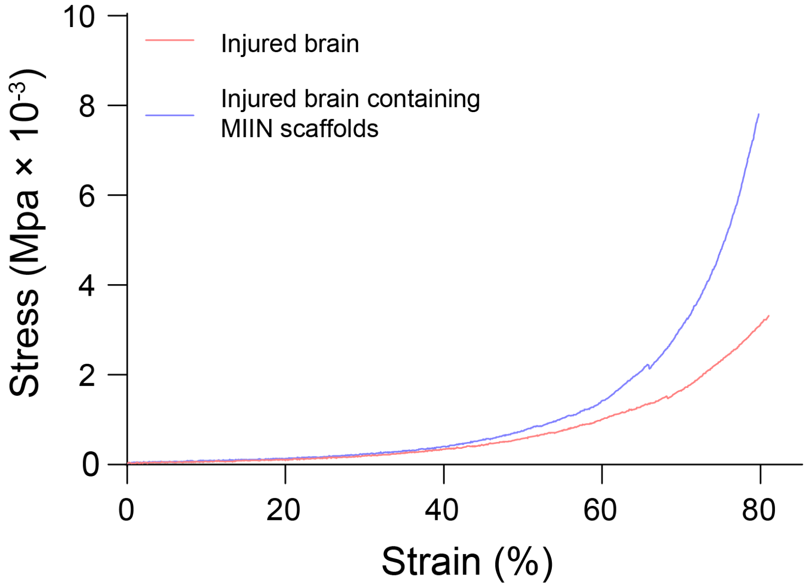

Supplementary Fig. 8 The elastic modulus of brain tissue with and without the internal scaffolds. The experiments were repeated three times independently.

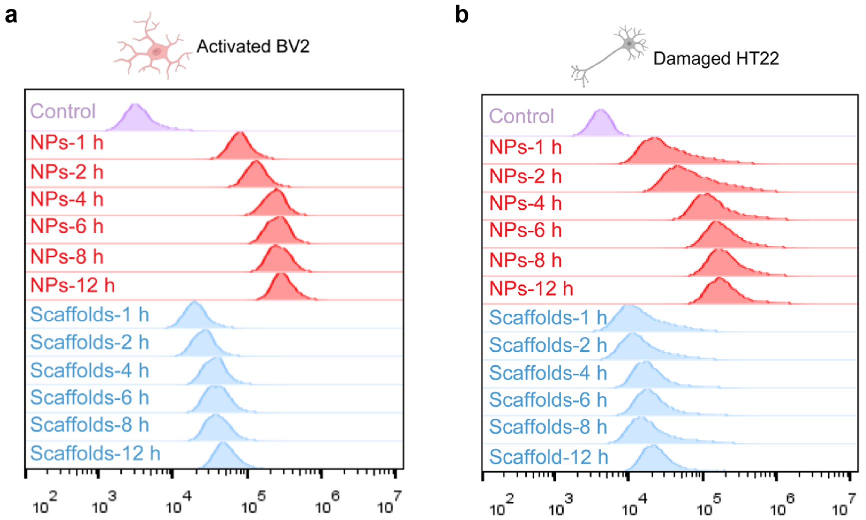

Supplementary Fig. 9 A representative histogram depicting the uptake behavior of MIIN or MIIN scaffolds by activated BV2 cells analyzed by flow cytometry. The experiments were repeated three times independently. Schematic diagram of cell was created in BioRender. Tong, S. (2026) https://BioRender.com/5cuvlhm

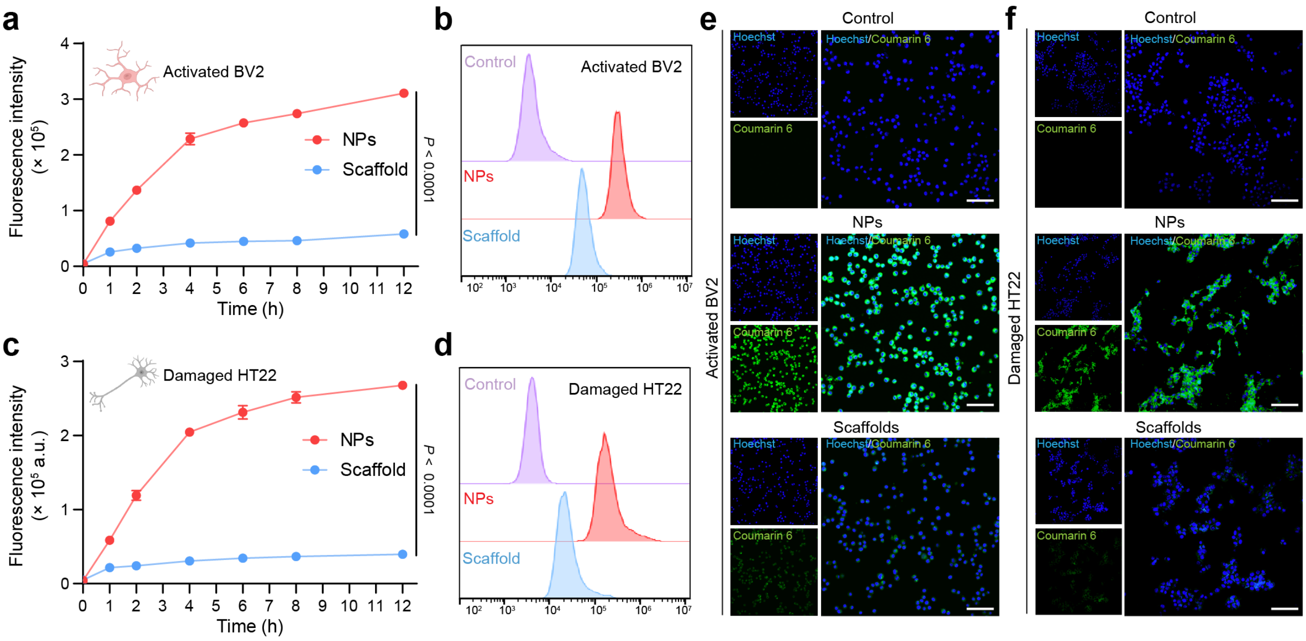

Supplementary Fig. 10 The ability of scaffolds to resist rapid cellular uptake and clearance. a, b The uptake behavior (**a**) of MIIN or MIIN scaffolds by activated BV2 cells and a representative histogram (**b**) at 24 hours analyzed by flow cytometry ($n = 3$). Schematic diagram of cell was created in BioRender. Tong, S. (2026) https://BioRender.com/5cuvlhm **c, d** The uptake behavior (**c**) of MIIN and MIIN scaffolds by damaged HT22 cells, and a representative histogram (**d**) at 24 hours analyzed by flow cytometry ($n = 3$). Schematic diagram of cell was created in BioRender. Tong, S. (2026) https://BioRender.com/5cuvlhm **e, f** Representative fluorescence images illustrating the uptake of MIIN or MIIN scaffolds by activated BV2 cells (**e**) and damaged HT22 cells (**f**). Scale, 100 μm. The experiments were repeated three times independently. The data are presented as the mean ± SD of three independent biological replicates, along with the corresponding P values. Statistical analyses are performed using two-way ANOVA with Tukey's post hoc test.

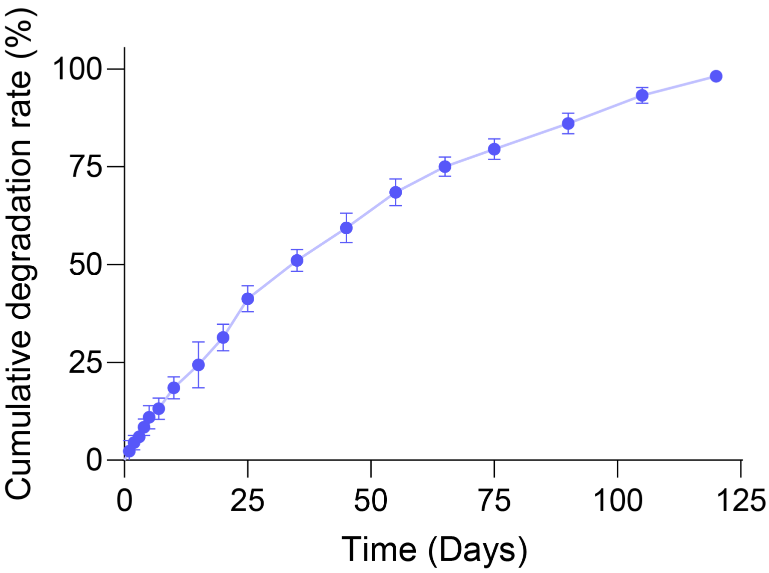

Supplementary Fig. 11 The degradation curve of MIIN scaffolds ($n = 3$). Data represent mean ± SD of three independent replicates.

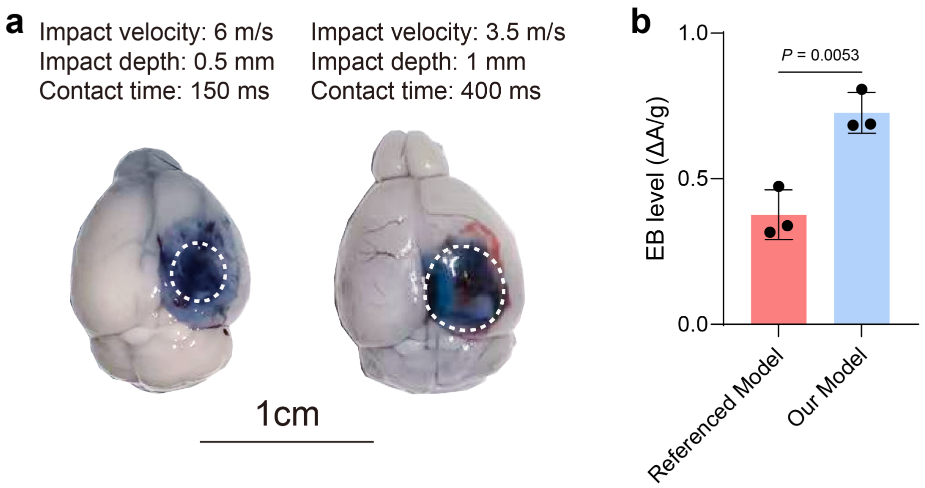

Supplementary Fig. 12 Analysis of Evans blue extravasation in the brain. a, b Representative images (**a**) and quantitative analysis (**b**) of Evans blue leakage in the brain under different CCI parameters ($n = 3$). The data are presented as the mean ± SD of three independent biological replicates, along with the corresponding P values. Statistical analyses are performed using one-way ANOVA with Tukey's post hoc test.

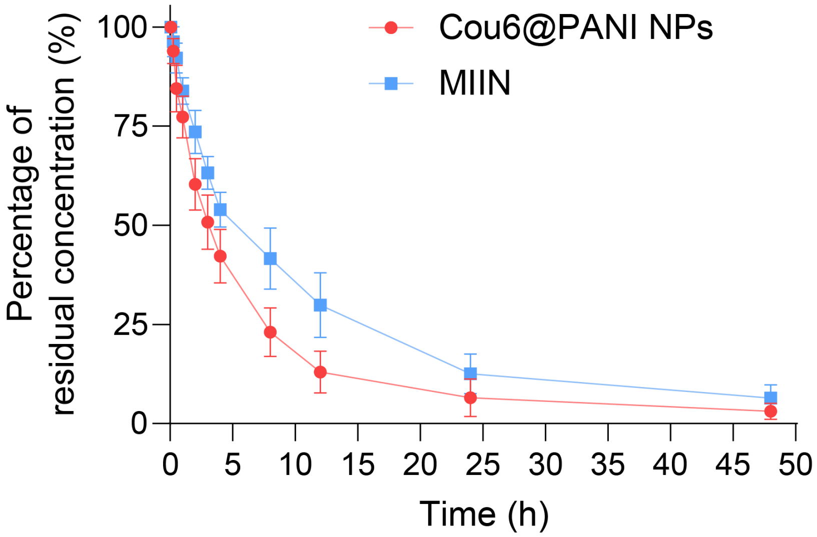

Supplementary Fig. 13 The pharmacokinetic properties of MIIN scaffolds (n = 3). Data represent mean ± SD of three independent biological replicates.

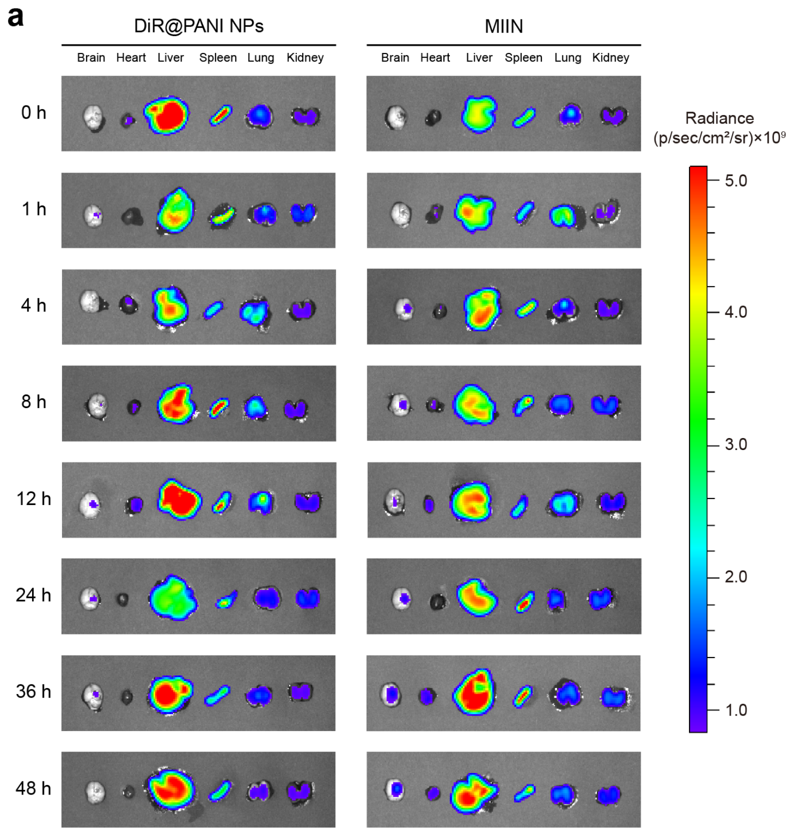

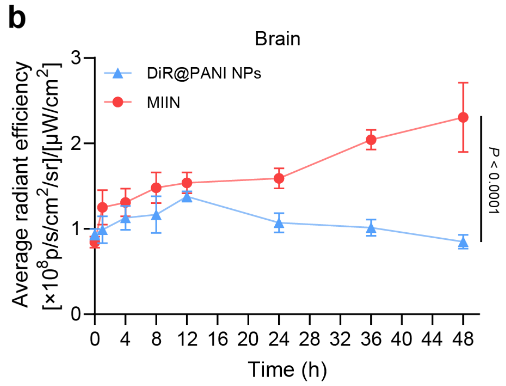

c

Brain-to-Liver Ratio (%)

	DiR@PANI NPs	MIIN
0 h	4.3761±0.3539	8.9253±0.8959
1 h	6.9874±1.0212	8.2756±1.0832
4 h	7.4126±1.6382	8.2641±0.9945
8 h	6.4807±0.6995	9.2133±1.2845
12 h	6.0592±0.8673	9.9242±1.9607
24 h	8.1954±0.8493	12.8151±1.8600
36 h	4.8078±1.2157	9.8384±0.6862
48 h	3.9349±0.4935	15.1515±2.4021

Supplementary Fig. 14 *Ex vivo* fluorescence analysis at multiple time points. a *Ex vivo* DiR fluorescence imaging of main organs obtained from CCI mice at multiple time points after injection. The experiments were repeated three times independently. **b** Semi-quantitative analysis of the *ex vivo* brain radiant efficiency shown in (**a**) (n = 3). **c** Brain-to-liver fluorescence signal ratio shown in (**a**). The data are presented as the mean ± SD of three independent biological replicates, along with the corresponding P values. Statistical analyses are performed using two-way ANOVA with Tukey's post hoc test.

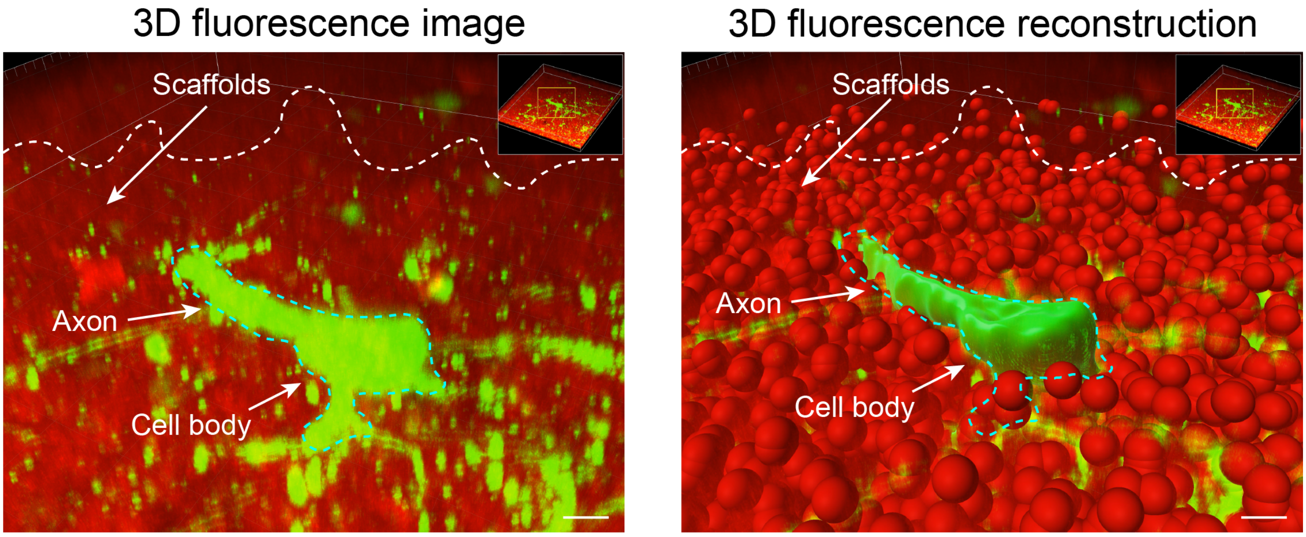

Supplementary Fig. 15 3D fluorescence image and 3D reconstructed image showing the infiltration of axons (Biocytin-labeled) into the MIIN scaffold (Rhodamine B-labeled) at the brain injury site. The 3D reconstructed image was rendered using Imaris software. The fluorescence signals of the scaffolds were reconstructed based on a spherical model, which does not represent its actual shape or size. Scale,

10 μm. The experiments were repeated six times independently.

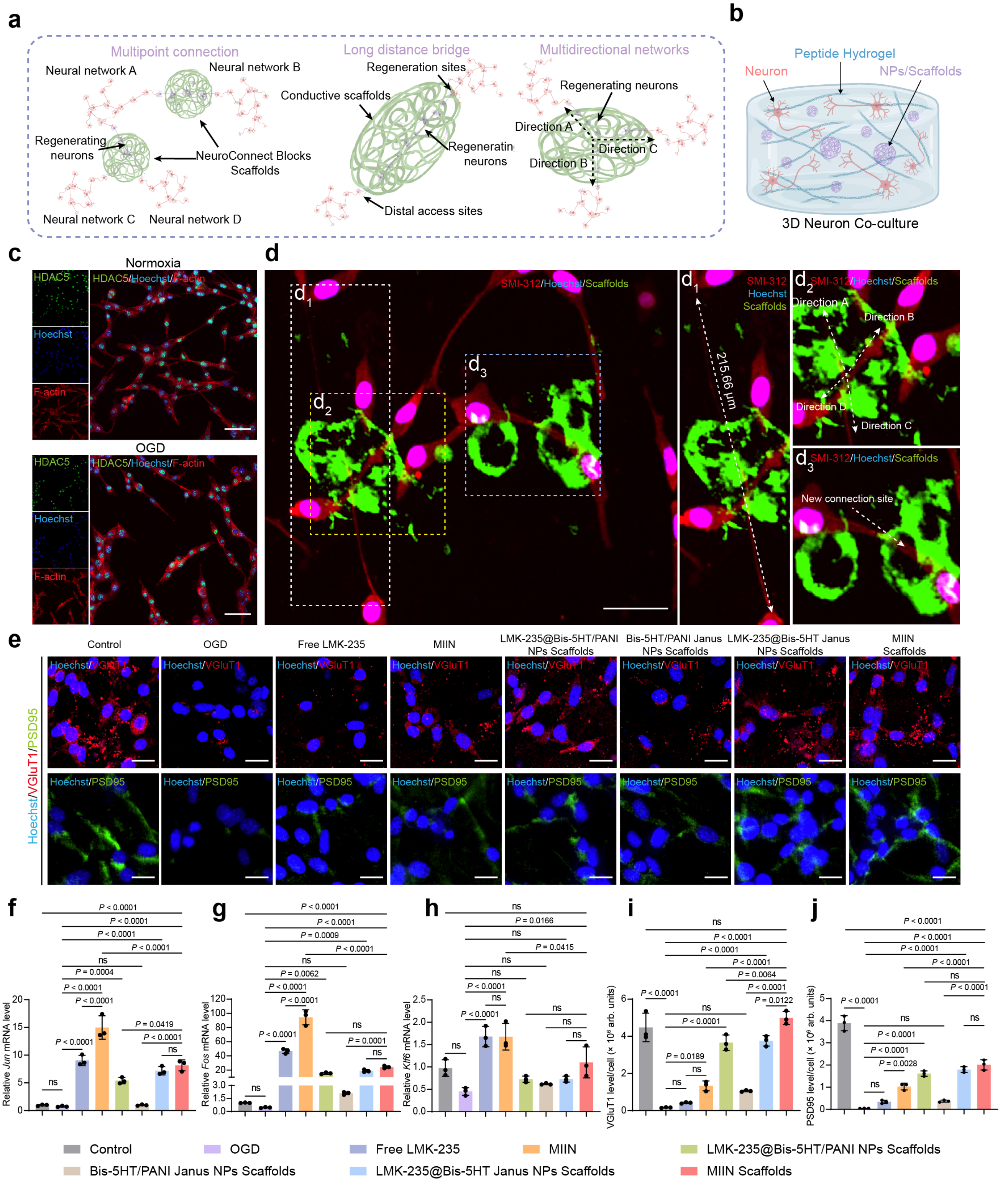

Supplementary Fig. 16 MIIN scaffolds activate intrinsic regenerative programs and guide axonal growth *in vitro*. a A schematic representation illustrating the multi-site connections, long-distance bridging, and multi-directional guidance capabilities of the MIIN scaffolds. Created in BioRender. Tong, S. (2026) https://BioRender.com/5cuvlhm **b** Schematic diagram for the 3D co-culture of neurons. Created in BioRender. Tong, S. (2026) https://BioRender.com/5cuvlhm **c** Representative images

showing the subcellular localization of HDAC5 in HT22 cells before and after injury. Scale, 50 μm. The experiments were repeated three times independently. **d** Representative images illustrating the regenerative guidance effects of MIIN scaffolds, including (**d1**) long-distance bridging, (**d2**) multi-directional guidance and (**d3**) multi-site connections. Scale, 500 μm. The experiments were repeated three times independently. **e** Representative images demonstrating the promotive effects of the scaffolds on the formation of synaptic connections (VGluT1 and PSD95) in injured HT22 cells. Scale, 25 μm. The experiments were repeated three times independently. **f-h** qPCR analysis of mRNA levels of (**f**) *Jun*, (**g**) *Fos*, and (**h**) *Klf6* in injured HT22 cells (n = 3). **i, j** Semi-quantitative analysis of (**i**) VGluT1 and (**j**) PSD95 in injured HT22 cells as shown in (**e**) (n = 3). The data are presented as the mean ± SD of three independent biological replicates, along with the corresponding P values. Statistical analyses are performed using one-way ANOVA with Tukey's post hoc test.

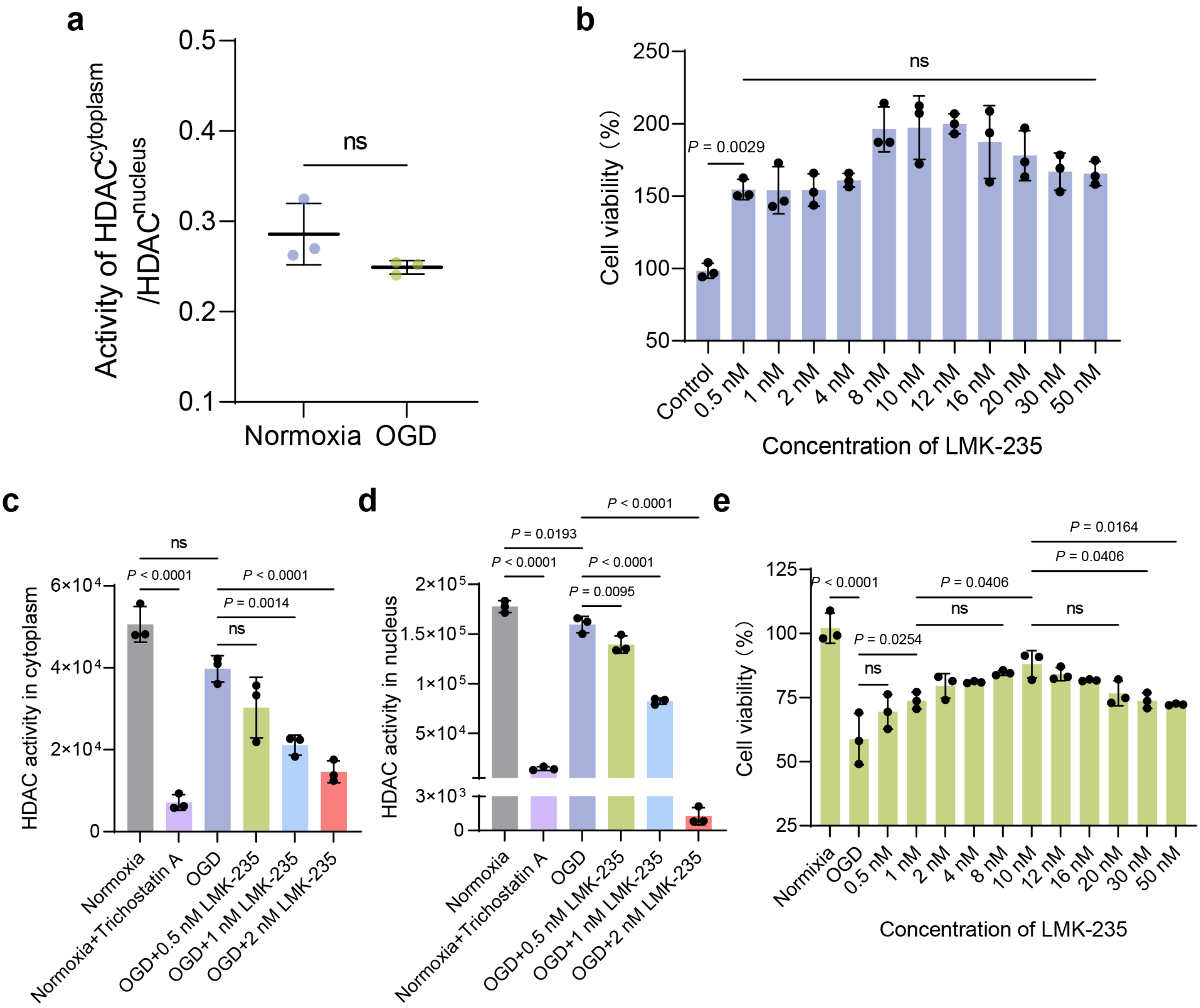

Supplementary Fig. 17 HT22 cell-related assays. **a** The ratio of HDAC activity between the cytoplasm and nucleus in HT22 cells (n = 3). **b** Cell viability assessment at different concentrations of LMK-235 on HT22 cells (n = 3). **c** The cytoplasmic HDAC activity in injured HT22 cells with the classic HDAC inhibitor Trichostatin A or different concentrations of LMK-235 (n = 3). **d** The nuclear HDAC activity in injured HT22 cells with the classic HDAC inhibitor Trichostatin A or different concentrations of LMK-235 (n = 3). **e** Cell viability assessment at different concentrations of LMK-

235 on injured HT22 cells (n = 3). The data are presented as the mean ± SD of three independent biological replicates, along with the corresponding P values. Statistical analyses are performed using two tailed Student's t-test for panels **a**, and one-way ANOVA for panels **b-e**, followed by Tukey's post hoc test.

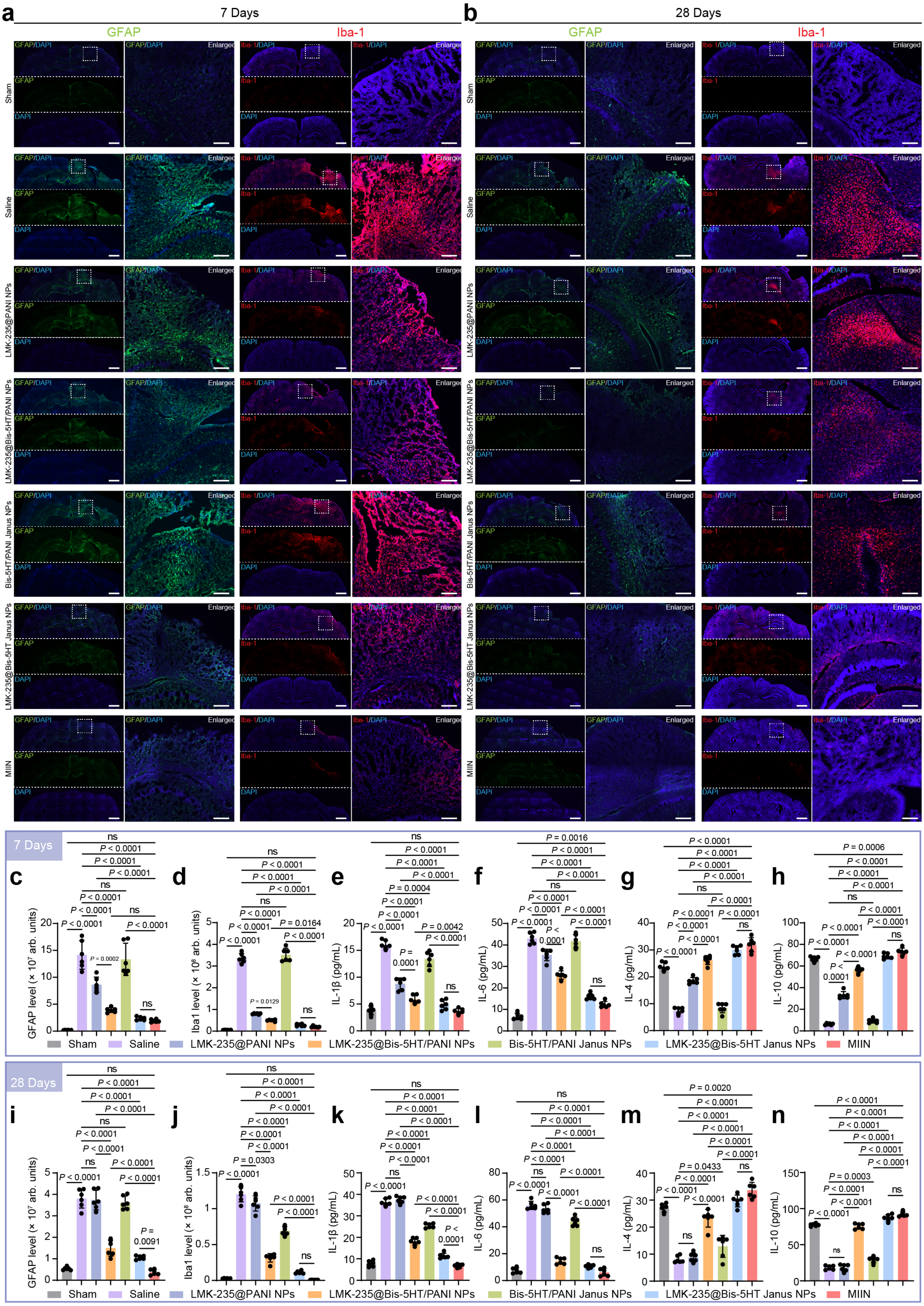
a
7 Days
GFAP
Iba-1
b
28 Days
GFAP/DAPI
Iba-1/DAPI
Enlarged
Sham
Saline
LMK-235@PANI NPs
LMK-235@Bis-5HT/PANI NPs
Bis-5HT/PANI Janus NPs
LMK-235@Bis-5HT Janus NPs
MIIN
7 Days
c
d
e
f
g
h
GFAP level (× 10⁷ arb. units)
Iba1 level (× 10⁸ arb. units)
IL-1β (pg/mL)
IL-6 (pg/mL)
IL-4 (pg/mL)
IL-10 (pg/mL)
28 Days
i
j
k
l
m
n
Sham
Saline
LMK-235@PANI NPs
LMK-235@Bis-5HT/PANI NPs
Bis-5HT/PANI Janus NPs
LMK-235@Bis-5HT Janus NPs
MIIN

Supplementary Fig. 18 MIIN persistently reshape the injured microenvironment, enabling sustained neural circuit reconstruction. a, b Representative images of glial scars (GFAP) and microglia (Iba-1) in injured sites of various groups at 7 (**a**) and 28 days (**b**) post-injury. Scale, 1 mm (scan); 200 μm (enlarged image). The experiments were repeated six times independently. **c, d** Quantitative analysis showing the fluorescence intensity of (**c**) GFAP and (**d**) Iba-1 corresponding to (**a**) (n = 6). **e-h** Detection of pro-inflammatory cytokines in the homogenates of injured brains from various groups at 7 days post-injury, including (**e**) IL-1β, (**f**) IL-6, (**g**) IL-4, and (**h**) IL-10 (n = 6). **i, j** Quantitative analysis showing the fluorescence intensity of (**i**) GFAP and (**j**) Iba-1 corresponding to (**b**) (n = 6). **k-n** Detection of pro-inflammatory cytokines in the homogenates of the injured brain from various groups at 28 days post-injury, including (**k**) IL-1β, (**l**) IL-6, (**m**) IL-4, and (**n**) IL-10 (n = 6). The data are presented as the mean ± SD of six independent biological replicates, along with the corresponding P values. Statistical analyses are performed using one-way ANOVA with Tukey's post hoc test.

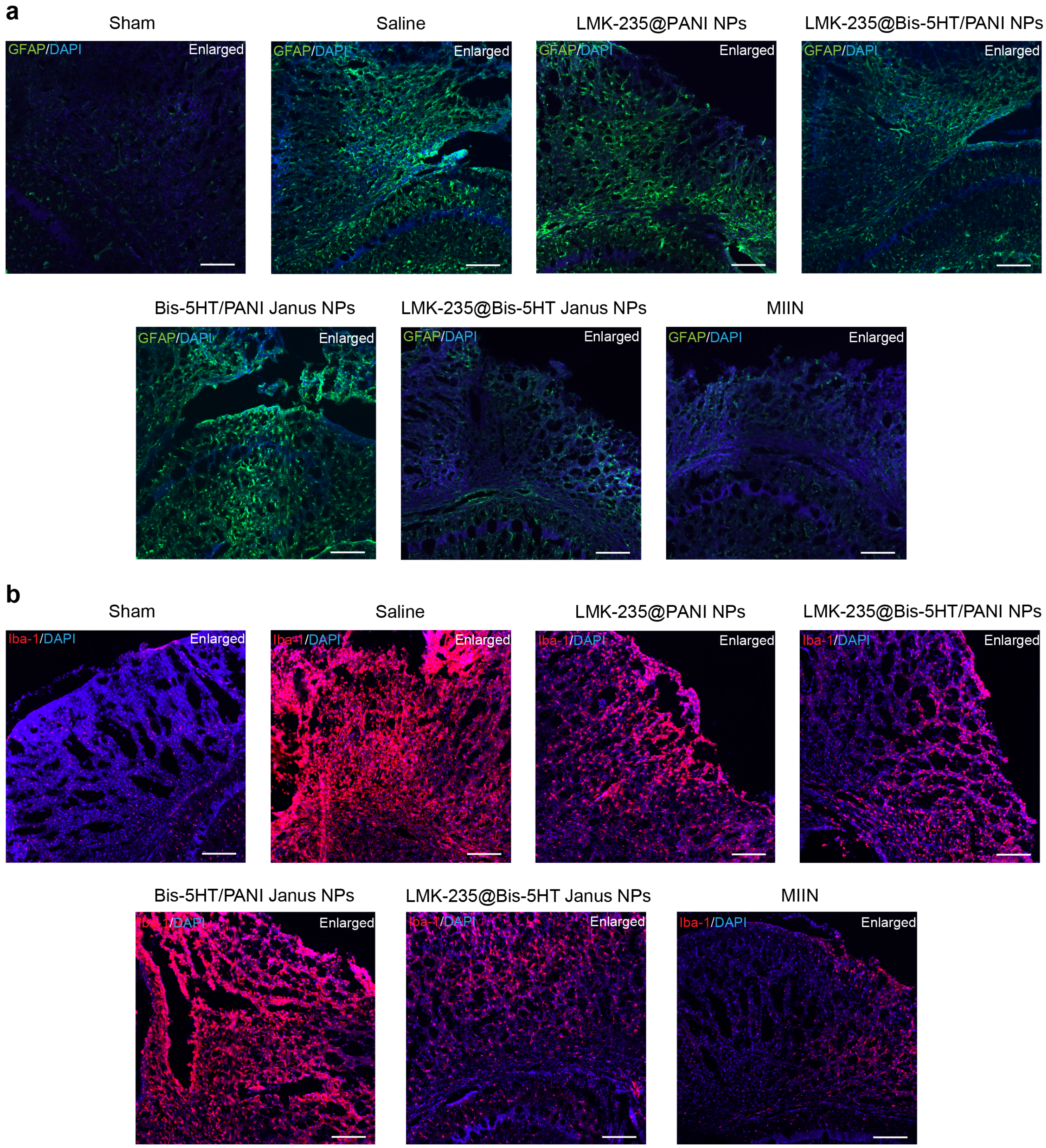

Supplementary Fig. 19 a, b Representative images of (**a**) glial scars (GFAP) and (**b**) microglia (Iba-1) in injured sites of various groups at 7 days post-injury. Scale, 200 μm. The experiments were repeated six times independently.

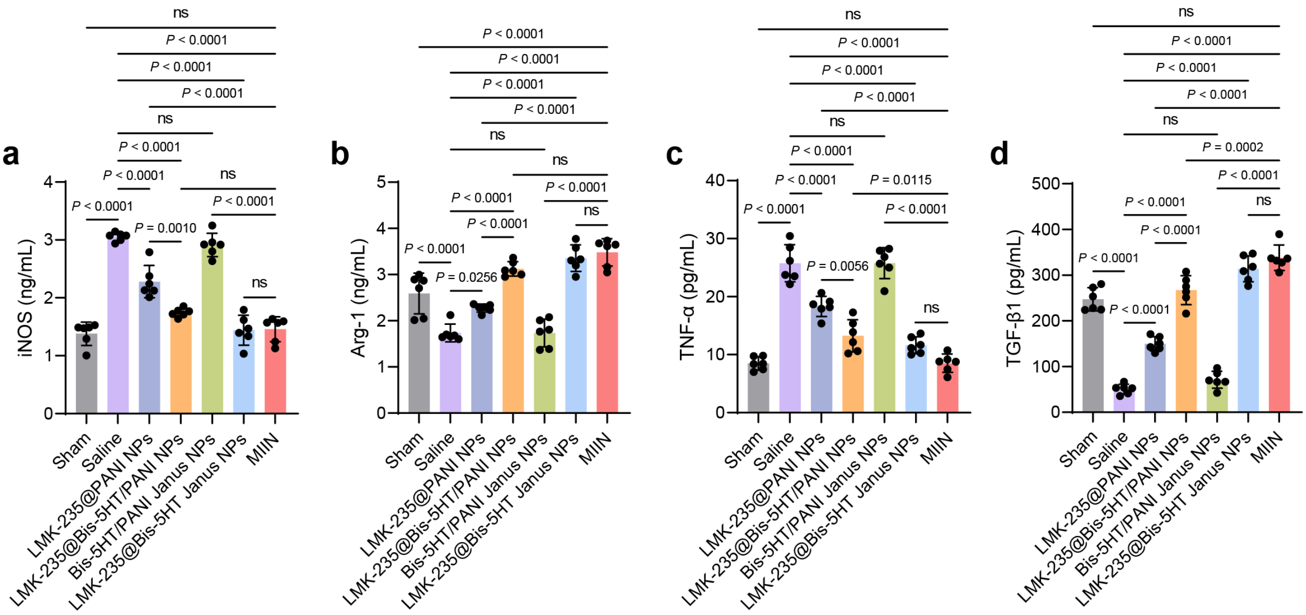

Supplementary Fig. 20 a-d Quantification of the expression level of (**a**) iNOS, (**b**) Arg-1, (**c**) TNF-α, and (**d**) TGF-β1 in the injury brain 7 days after injury (*n* = 6). The data are presented as the mean ± SD of six independent biological replicates, along with the corresponding P values. Statistical analyses are performed using one-way ANOVA with Tukey's post hoc test.

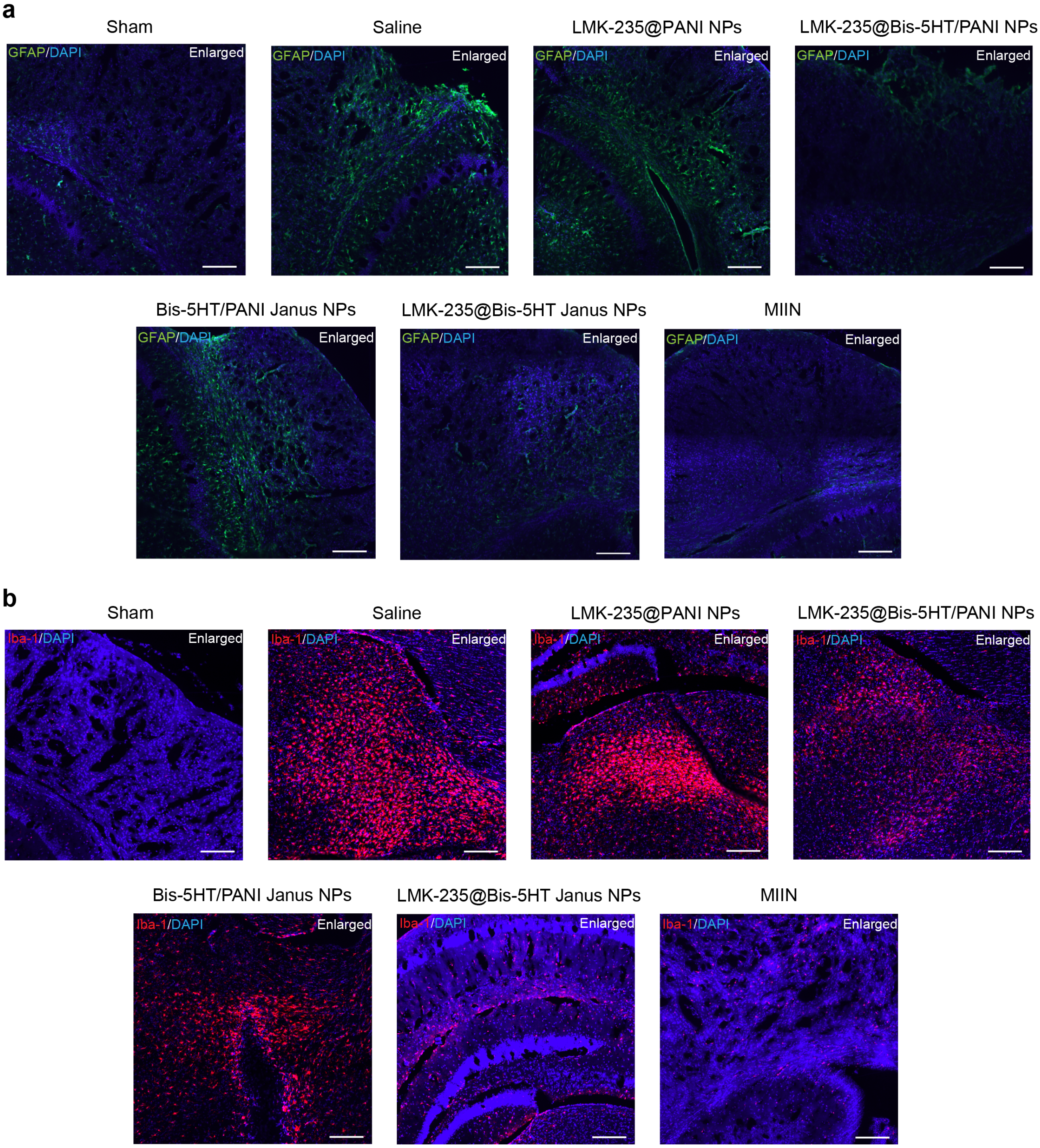

Supplementary Fig. 21 a, b Representative images of (**a**) glial scars (GFAP) and (**b**) microglia (Iba-1) in injured sites of various groups at 28 days post-injury. Scale, 200 μm. The experiments were repeated six times independently.

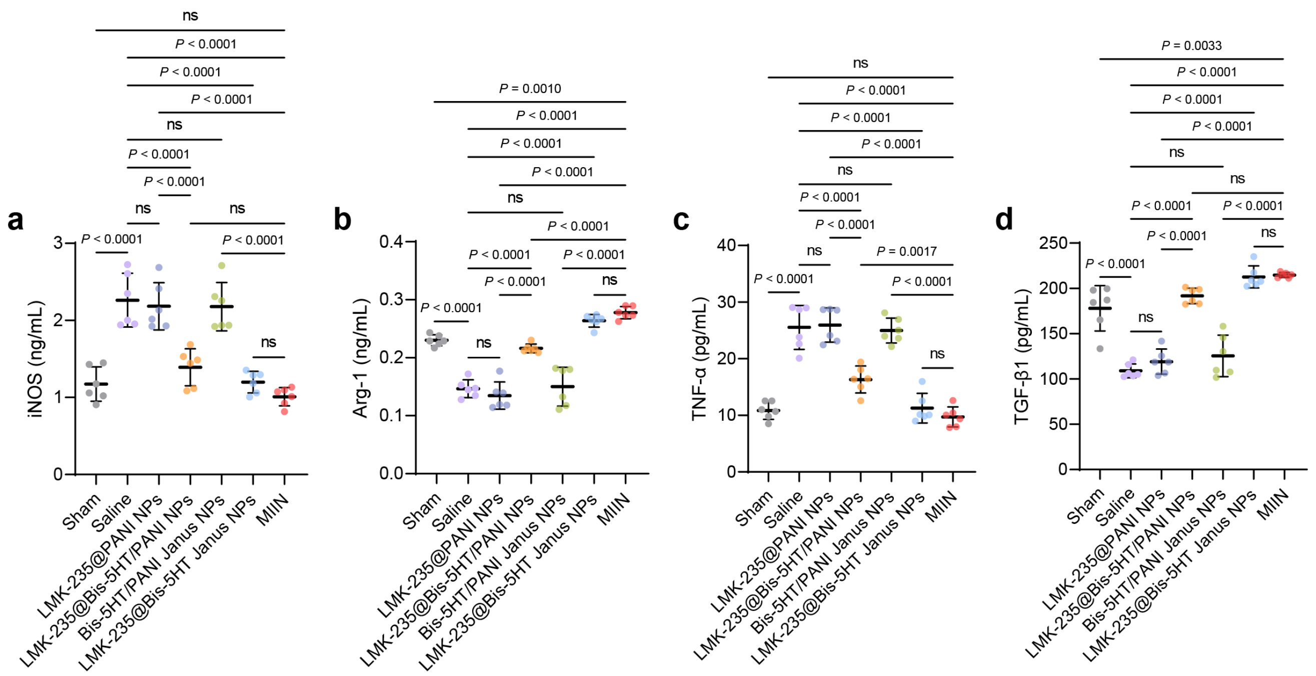

Supplementary Fig. 22 a-d Quantification of the expression level of (**a**) iNOS, (**b**) Arg-1, (**c**) TNF-α, and (**d**) TGF-β1 in the injury brain 28 days after injury (*n* = 6). The data are presented as the mean ± SD of six independent biological replicates, along with the corresponding P values. Statistical analyses are performed using one-way ANOVA with Tukey's post hoc test.

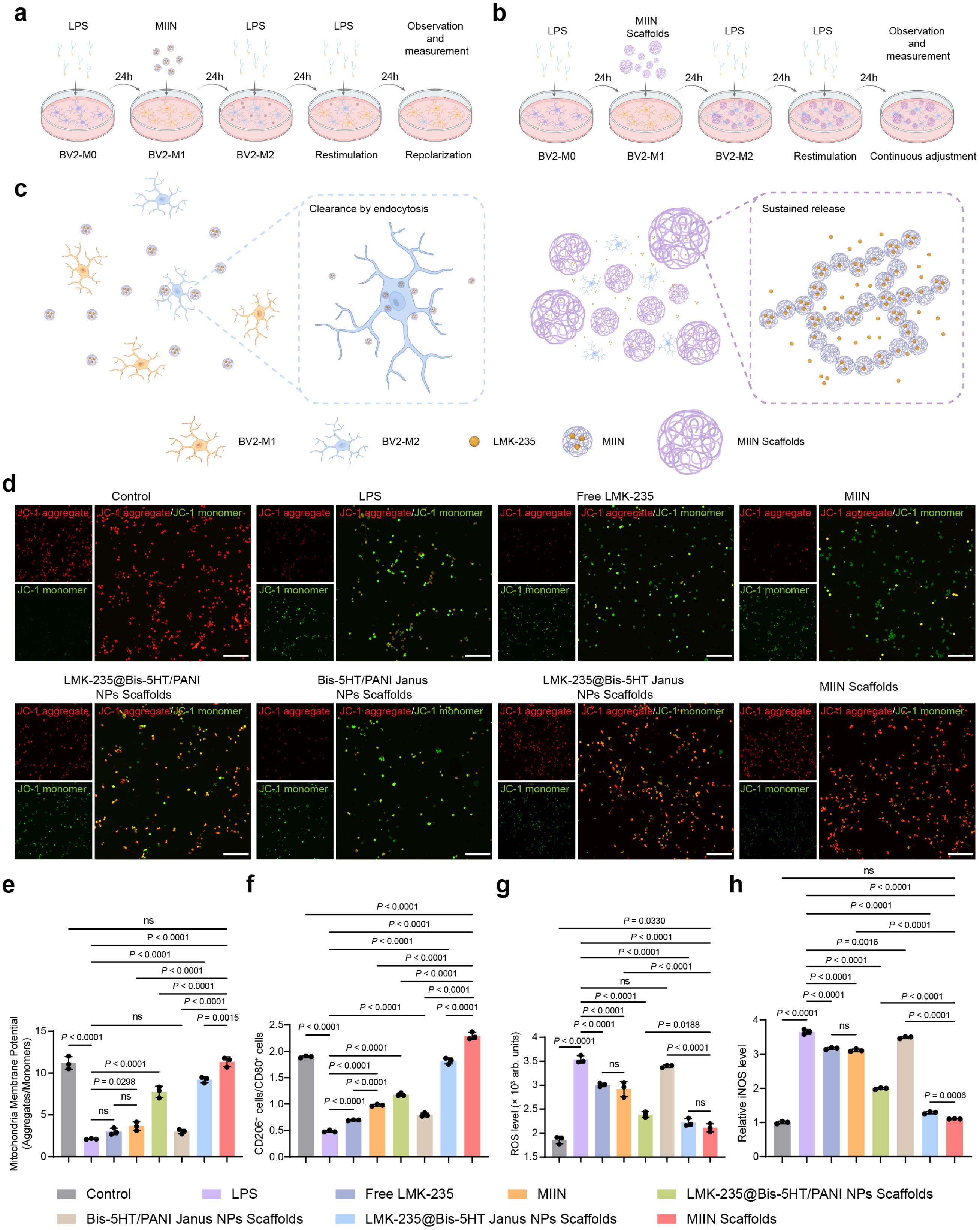

Supplementary Fig. 23 MIIN scaffolds persistently reshape dysregulated microglia and restore mitochondrial homeostasis *in vitro*. a Schematic diagram for the co-incubation of MIIN monomers with BV2 cells. Created in BioRender. Tong, S. (2026) https://BioRender.com/5cuvlhm **b** Schematic diagram for the co-incubation of MIIN scaffolds with BV2 cells. Created in BioRender. Tong, S. (2026) https://BioRender.com/5cuvlhm **c** Schematic representation of the mechanisms by which MIIN

monomers and MIIN scaffolds regulate BV2 cells. Created in BioRender. Tong, S. (2026) https://BioRender.com/5cuvlhm **d, e** Representative images (**d**) and semi-quantitative results (**e**) of JC-1 staining in activated BV2 cells subjected to different treatments (*n* = 3). Scale, 50 µm. **f** Flow cytometry analysis of $CD80^+$ and $CD206^+$ cells in activated BV2 cells after different treatments (*n* = 3). **g, h** Quantification of the level of ROS (**g**) and iNOS (**h**) in activated BV2 cells after different treatments (*n* = 3). The data are presented as the mean ± SD of three independent biological replicates, along with the corresponding P values. Statistical analyses are performed using one-way ANOVA with Tukey's post hoc test.

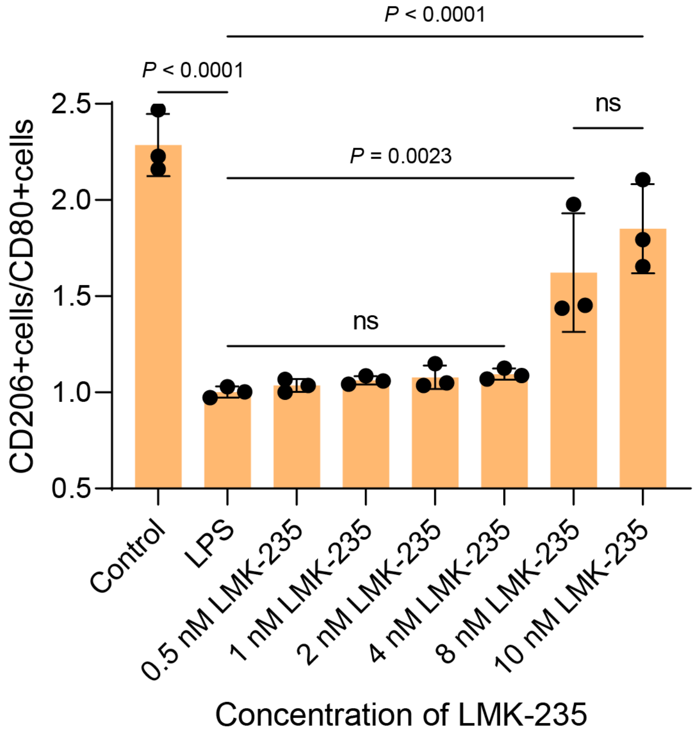

Supplementary Fig. 24 Flow cytometry analysis of the ratio of $CD206^+$ BV2 cells to $CD80^+$ BV2 cells with different concentrations of LMK-235 (*n* = 3). The data are presented as the mean ± SD of three independent biological replicates, along with the corresponding P values. Statistical analyses are performed using one-way ANOVA with Tukey's post hoc test.

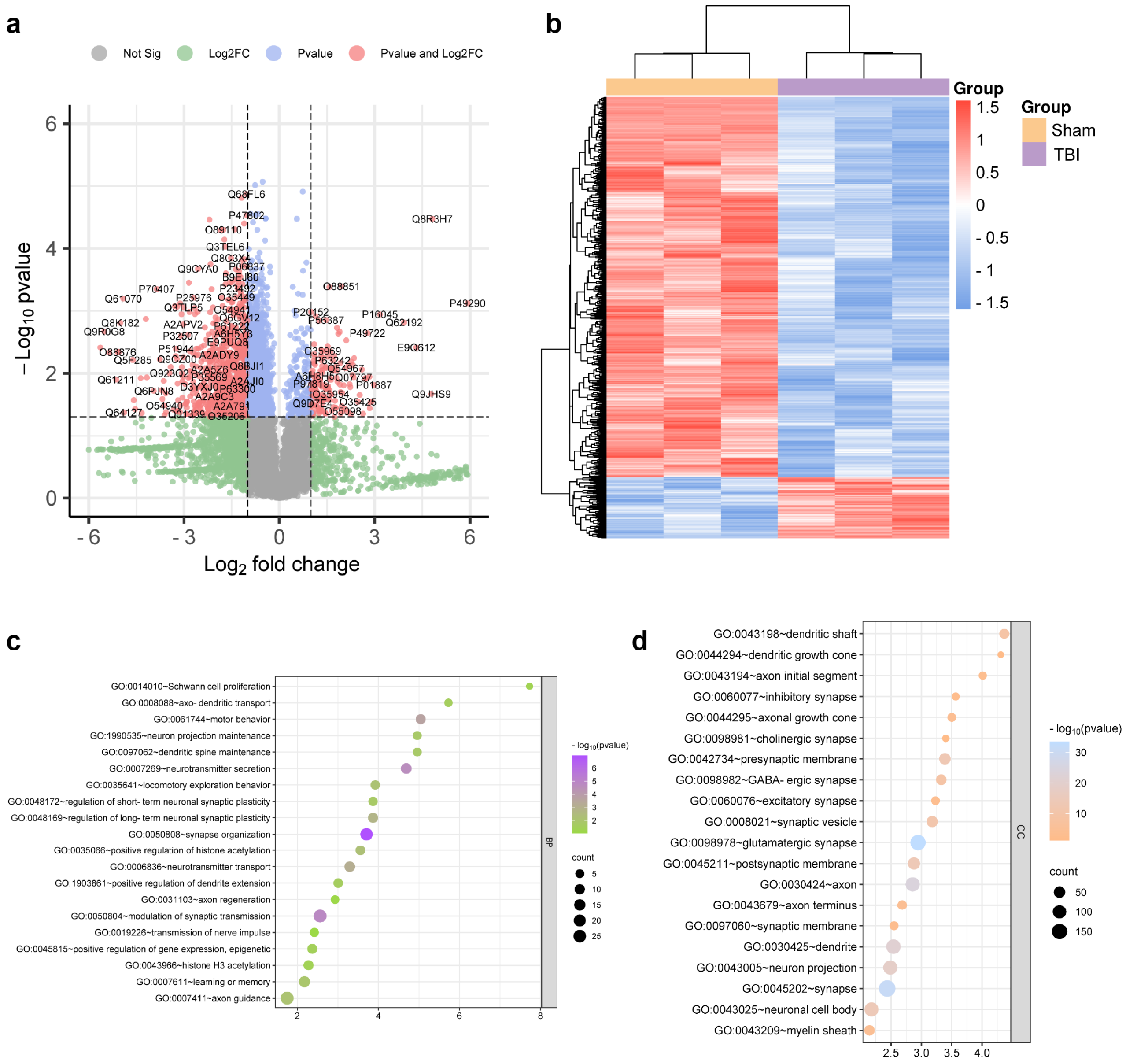

Supplementary Fig. 25 Proteomic analysis of the TBI and sham groups. a Volcano plots showing differentially expressed proteins in brain tissues extracted from the injury site of TBI mice in comparison to those in sham-operated mice ($P < 0.05$ and fold change ≥ 1.5). **b** Heat map depicting significantly up-regulated and down-regulated proteins in brain tissues extracted from the injury site of TBI mice in comparison to those in sham-operated mice. **c** GO enrichment analysis of significantly down-regulated proteins categorized into "biological process" in brain tissues extracted from the injury site of TBI mice in comparison to those in sham-operated mice. **d** GO enrichment analysis of significantly down-regulated proteins categorized into "cellular component" in brain tissues extracted from the injury site of TBI mice in comparison to those in sham-operated mice.

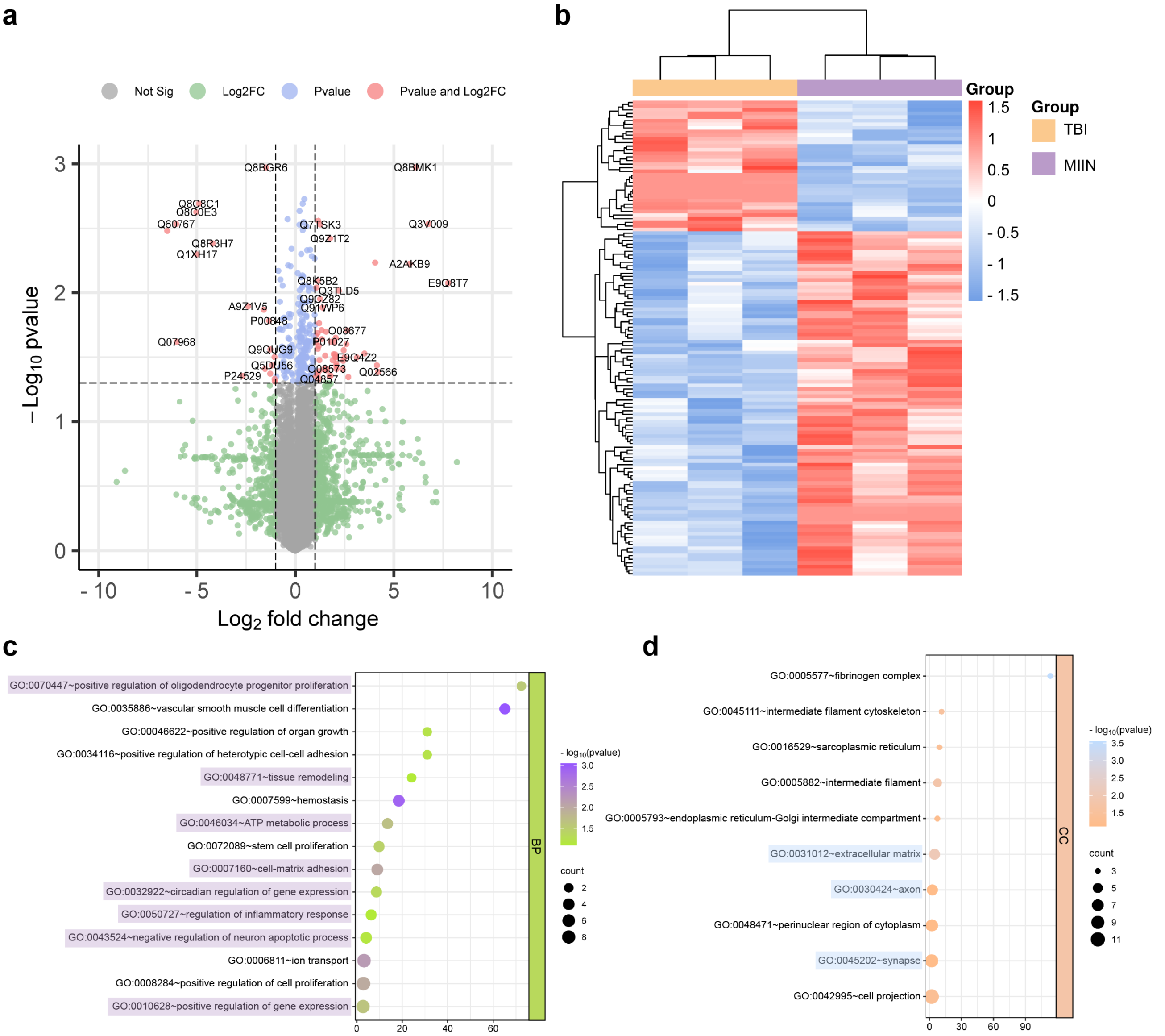

Supplementary Fig. 26 Proteomic analysis of the MIIN and TBI groups. a Volcano plots showing differentially expressed proteins in brain tissues extracted from the injury site of TBI mice treated with MIIN in comparison to those in untreated mice ($P < 0.05$ and fold change ≥ 1.5). **b** Heat map depicting significantly up-regulated and down-regulated proteins in brain tissues extracted from the injury site of TBI mice treated with MIIN in comparison to those in untreated mice. **c** GO enrichment analysis of significantly up-regulated proteins categorized into "biological process" in brain tissues extracted from the injury site of TBI mice treated with MIIN in comparison to those in untreated mice. **d** GO enrichment analysis of significantly up-regulated proteins categorized into "cellular component" in brain tissues extracted from the injury site of TBI mice treated with MIIN in comparison to those in untreated mice.

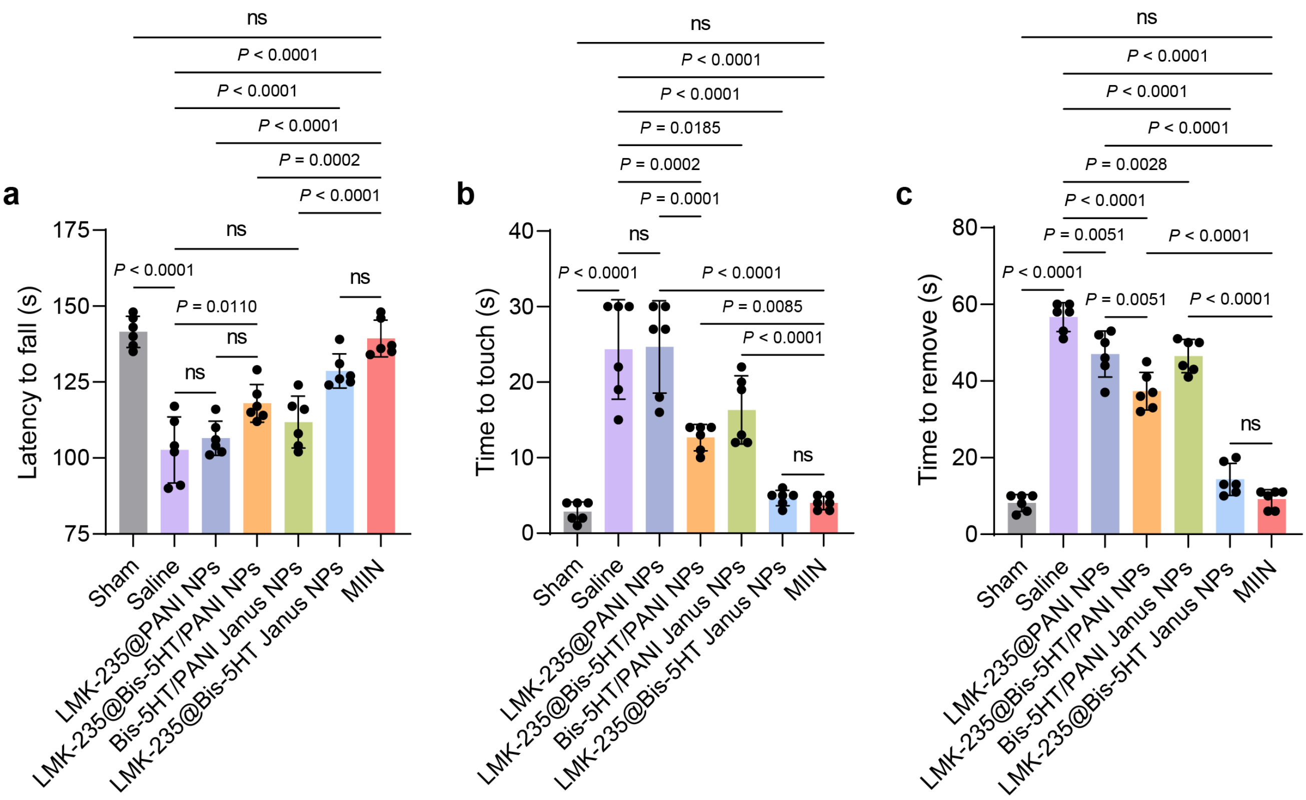

Supplementary Fig. 27 Effects of MIIN on motor and sensory functional recovery in TBI mice. a-c Quantification of (**a**) the latency on the rotarods in rotarod test, (**b**) the time to touch adhesive stickers and (**c**) the time to remove adhesive stickers in adhesive test 28 days after injury (n = 6). The data are presented as the mean ± SD of six independent biological replicates, along with the corresponding P values. Statistical analyses are performed using one-way ANOVA with Tukey's post hoc test.

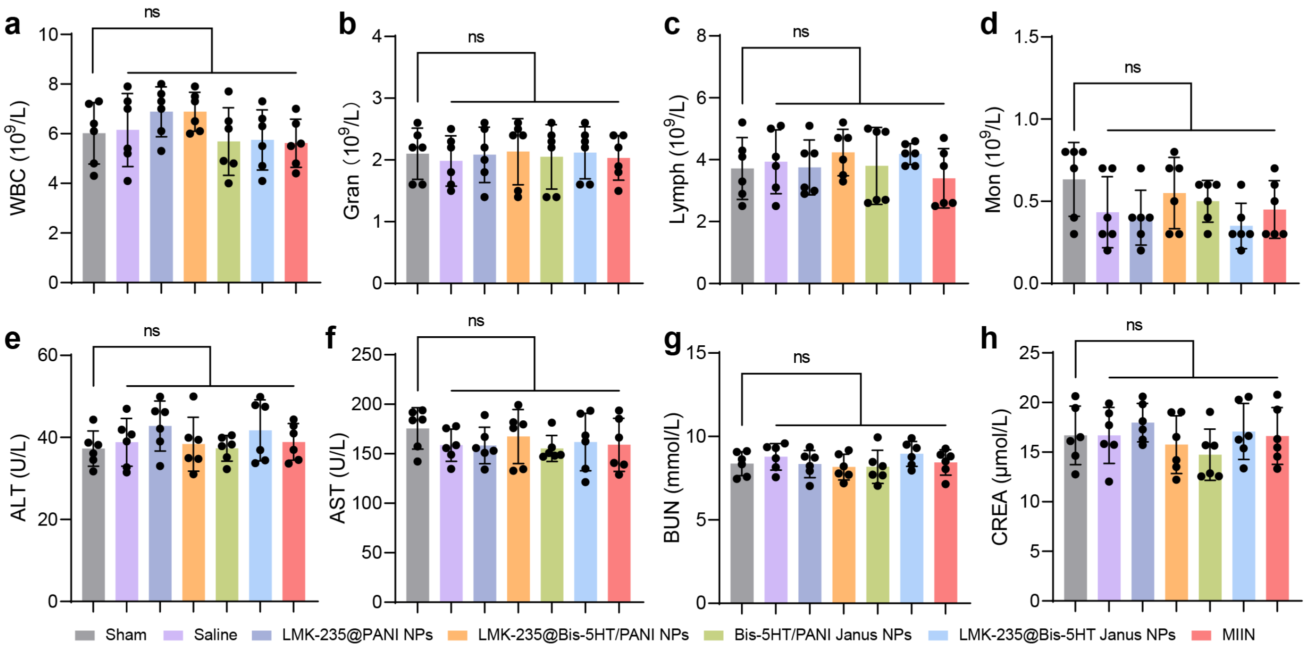

Supplementary Fig. 28 Hematological and biochemical analyses. a-d Evaluations of immune cell levels, including (**a**) WBC counts, (**b**) granulocyte counts, (**c**) lymphocytes counts and (**d**) monocytes

counts. **e-h** Assessments of liver and kidney function, including the serum levels of (**e**) ALT, (**f**) AST, (**g**) BUN, and (**h**) CREA. The data are presented as the mean ± SD of six independent biological replicates, along with the corresponding P values. Statistical analyses are performed using one-way ANOVA with Tukey's post hoc test.

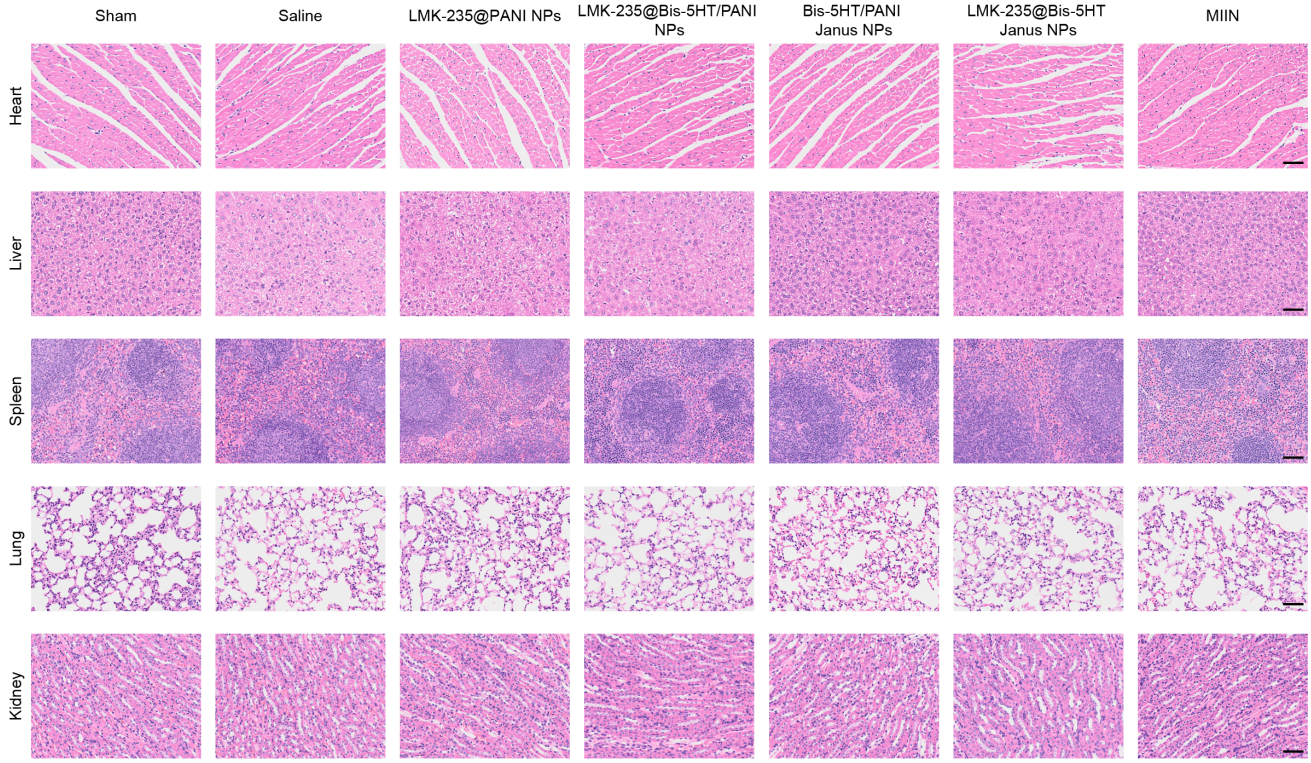

Supplementary Fig. 29 H&E staining to assess the toxicity of various formulations on major organs. Scale, 100 μm. The experiments were repeated six times independently.

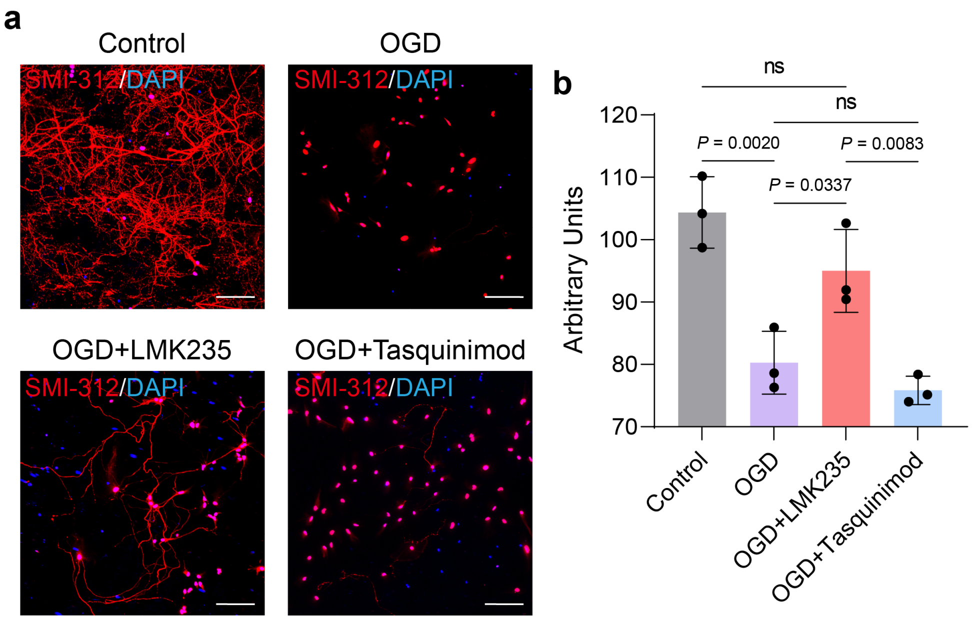

Supplementary Fig. 30 Comparative analysis of pro-axonal regeneration effects of LMK-235 versus tasquinimod. a Representative images of axonal regeneration in injured primary cortical neurons after treatment with 10 nM LMK-235 or tasquinimod. Scale, 100 μm. The experiments were

repeated three times independently. **b** Quantitative analysis of axonal outgrowth in primary cortical neurons shown in (**a**) ($n = 3$). The data are presented as the mean ± SD of three independent biological replicates, along with the corresponding P values. Statistical analyses are performed using one-way ANOVA with Tukey's post hoc test.

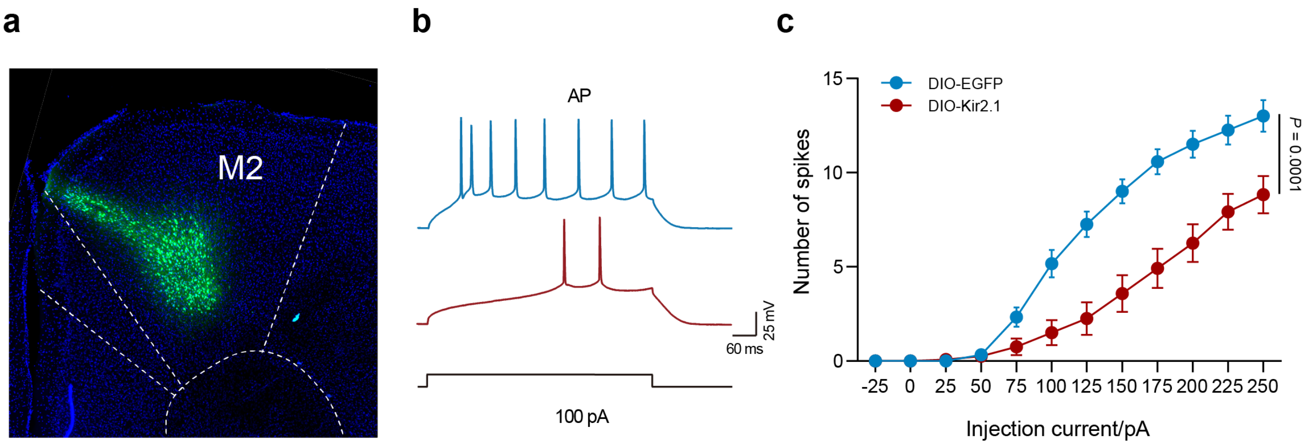

Supplementary Fig. 31 a Representative images of the rAVV carrying the Kir2.1 gene transduced the neurons in the M2 region of the motor cortex. Scale, 500 μm. The experiments were repeated six times independently. **b, c** The rAAV-EF1a-Kir2.1-EGFP injection site exhibited differences in (**b**) neuronal action potentials and (**c**) spike counts compared to the control ($n = 12$). The data are presented as the mean ± SD of twelve independent biological replicates, along with the corresponding P values. Statistical analyses are performed using two-way ANOVA with Tukey's post hoc test.

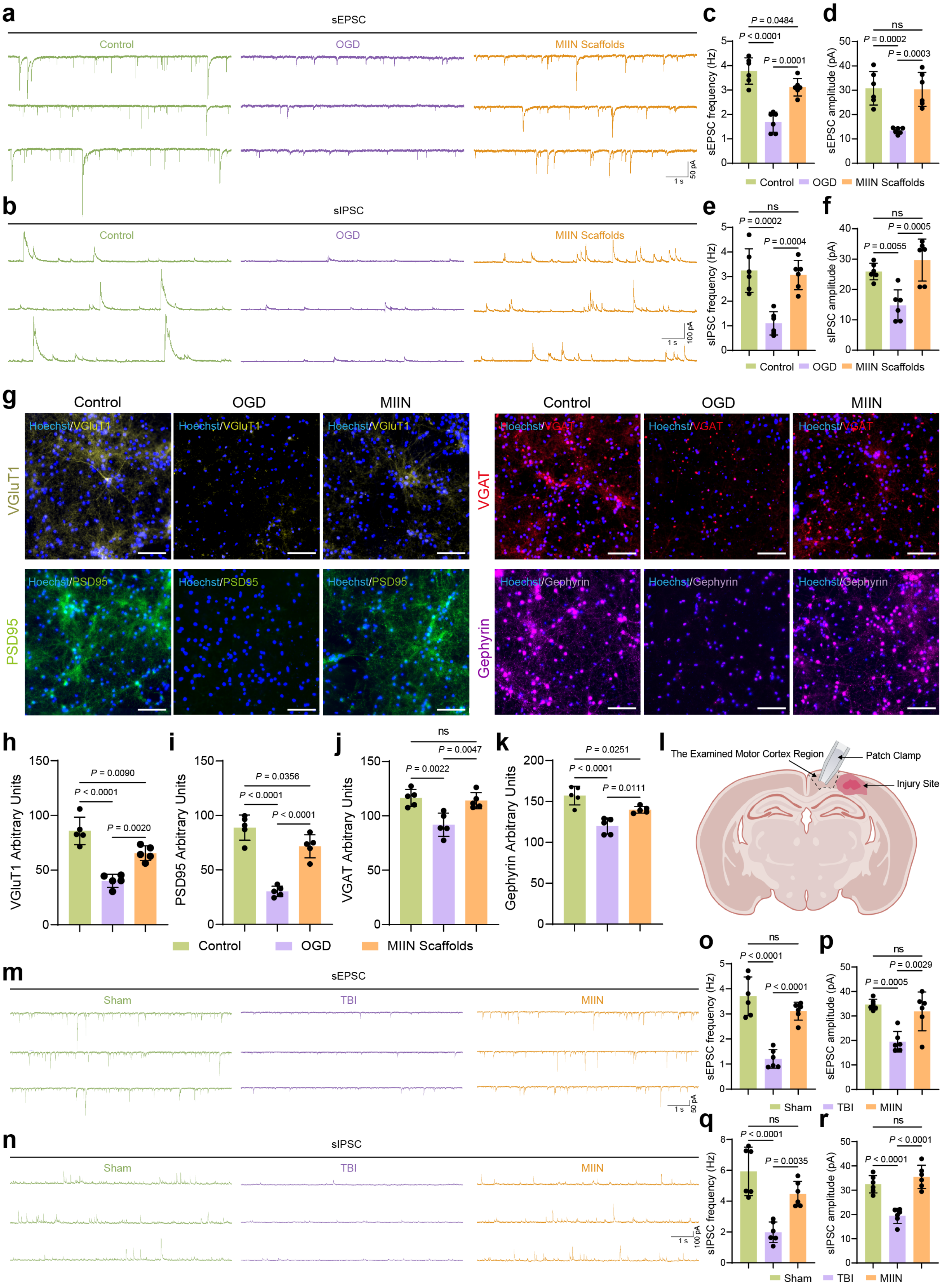

Supplementary Fig. 32 MIIN restore functional synaptic connectivity *in vitro* and *in vivo*. a, b Representative traces of (**a**) sEPSCs and (**b**) sIPSCs recorded from primary cortical neurons. The

experiments were repeated six times independently. **c, d** Quantitative analysis of the (**c**) frequency and (**d**) amplitude of sEPSCs in primary cortical neurons (n = 6). **e, f** Quantitative analysis of the (**e**) frequency and (**f**) amplitude of sIPSCs in primary cortical neurons (n = 6). **g** Representative images showing the levels of the presynaptic and postsynaptic markers—VGluT1, PSD95, VGAT, and Gephyrin—in primary cortical neurons. Scale, 100 μm. The experiments were repeated six times independently. **h-k** Quantitative analysis of (**h**) VGluT1, (**i**) PSD95, (**j**) VGAT, and (**k**) Gephyrin levels in primary cortical neurons as shown in (**g**) (n = 5). **l** Schematic representation of whole-cell patch-clamp recordings used to assess the electrical activity of neurons in the motor cortex affected by TBI. Created in BioRender. Tong, S. (2026) https://BioRender.com/5cuvlhm **m, n** Representative traces of (**m**) sEPSCs and (**n**) sIPSCs in the neurons from motor cortex slices affected by TBI. The experiments were repeated six times independently. **o, p** Quantitative analysis of the (**o**) frequency and (**p**) amplitude of sEPSCs (n = 6). **q, r** Quantitative analysis of the (**q**) frequency and (**r**) amplitude of sIPSCs (n = 6). The data are presented as the mean ± SD of five or six independent biological replicates, along with the corresponding P values. Statistical analyses are performed using one-way ANOVA with Tukey's post hoc test.

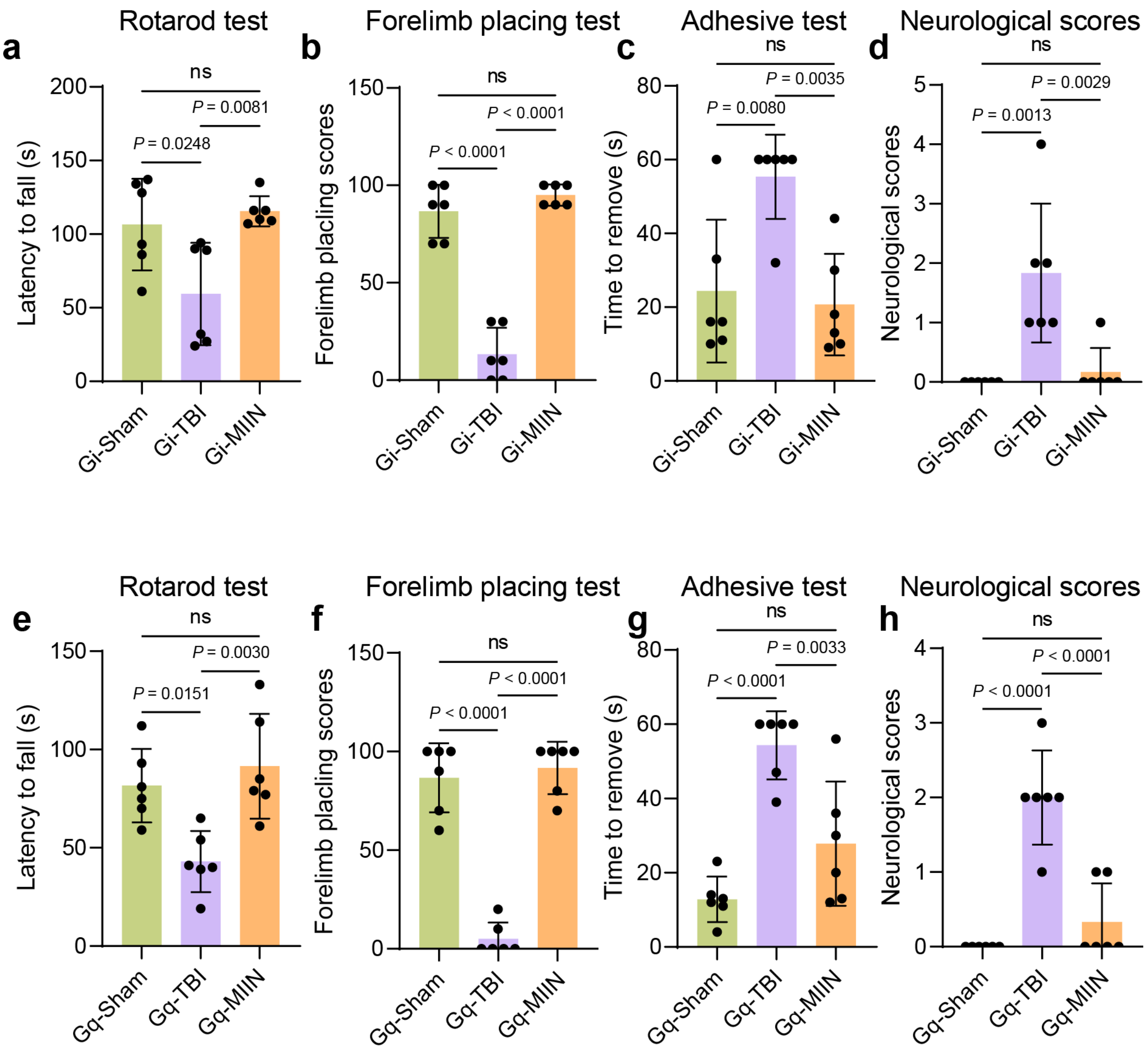

Supplementary Fig. 33 Therapeutic effects of MIIN on neurological functional recovery in hM4D(Gi) and hM3D(Gq) injured mice. a-d The latency on the rotarod (**a**), forelimb placing scores

(**b**), the time to remove adhesive stickers (**c**) and the neurological scores (**d**) of hM4D(Gi) mice with different treatments (n = 6). **e-h** The latency on the rotarod (**e**), forelimb placing scores (**f**), the time to remove adhesive stickers (**g**) and the neurological scores (**h**) of hM3D(Gq) mice with different treatments (n = 6). The data are presented as the mean ± SD of six independent biological replicates, along with the corresponding P values. Statistical analyses are performed using one-way ANOVA with Tukey's post hoc test.
