# Supplementary material for: Self-assembling scaffolds epigenetically reactivate and electroactively guide neuronal regeneration to restore central neural circuits
Source: Nat Commun. 2026 May 4;17:5987. doi: 10.1038/s41467-026-72397-6 (PMC13347008; doi:10.1038/s41467-026-72397-6)
Supplement: Supplementary file 2 — Description of Additional Supplementary Files [file 41467_2026_72397_MOESM2_ESM.pdf]

Description of Additional Supplementary Files

Supplementary Movie 1 - 3D reconstruction images of axons infiltrating the scaffolds. 3D fluorescence image and 3D reconstructed image showing the infiltration of axons (Biocytin-labeled) into the MIIN scaffolds (Rhodamine B-labeled) at the brain injury site. The 3D reconstructed image was rendered using Imaris software. The fluorescence signals of the scaffolds were reconstructed based on a spherical model, which does not represent its actual shape or size. The experiments were repeated six times independently.
