# Supplementary material for: Self-assembling scaffolds epigenetically reactivate and electroactively guide neuronal regeneration to restore central neural circuits
Source: Nat Commun. 2026 May 4;17:5987. doi: 10.1038/s41467-026-72397-6 (PMC13347008; doi:10.1038/s41467-026-72397-6)
Supplement: Supplementary file 4 — Reporting Summary [file 41467_2026_72397_MOESM4_ESM.pdf]

nature portfolio

Corresponding author(s): Jun Chen

Last updated by author(s): Mar 2, 2026

Reporting Summary

Nature Portfolio wishes to improve the reproducibility of the work that we publish. This form provides structure for consistency and transparency in reporting. For further information on Nature Portfolio policies, see our Editorial Policies and the Editorial Policy Checklist.

Statistics

For all statistical analyses, confirm that the following items are present in the figure legend, table legend, main text, or Methods section.

n/a	Confirmed	
☐	☒	The exact sample size (*n*) for each experimental group/condition, given as a discrete number and unit of measurement
☐	☒	A statement on whether measurements were taken from distinct samples or whether the same sample was measured repeatedly
☐	☒	The statistical test(s) used AND whether they are one- or two-sided<br>*Only common tests should be described solely by name; describe more complex techniques in the Methods section.*
☒	☐	A description of all covariates tested
☒	☐	A description of any assumptions or corrections, such as tests of normality and adjustment for multiple comparisons
☐	☒	A full description of the statistical parameters including central tendency (e.g. means) or other basic estimates (e.g. regression coefficient) AND variation (e.g. standard deviation) or associated estimates of uncertainty (e.g. confidence intervals)
☐	☒	For null hypothesis testing, the test statistic (e.g. *F*, *t*, *r*) with confidence intervals, effect sizes, degrees of freedom and *P* value noted<br>*Give P values as exact values whenever suitable.*
☒	☐	For Bayesian analysis, information on the choice of priors and Markov chain Monte Carlo settings
☒	☐	For hierarchical and complex designs, identification of the appropriate level for tests and full reporting of outcomes
☒	☐	Estimates of effect sizes (e.g. Cohen's *d*, Pearson's *r*), indicating how they were calculated

Our web collection on statistics for biologists contains articles on many of the points above.

Software and code

Policy information about availability of computer code

Data collection	1. Nanoparticles: Dynamic light scattering and Zeta potential analysis (Zetasizer Nano Zs90, UK), Transmission electron microscopy (TEM-1400 Plus Electron Microscope, Leica, Germany), Scanning electron microscope (GeminiSEM 300, ZEISS, Germany), Nanoparticle tracking analvsis technique (Nanosight LM 10, Malvern, UK).<br>2. lmaging system: Spinning disk laser confocal microscopy (SpinSR10, Olympus, Japan).<br>3. Quantitative system: High-performance liquid chromatography (Shimadzu, Japan), Microplate reader (Multiskan MK3, Thermo fisher, USA), Flow cytometry analysis (CytoFLEX, Beckman, USA).
Data analysis	Prism 9.0 software (GraphPad), FlowJo (10.4), ImageJ software (version 1.8.0).

For manuscripts utilizing custom algorithms or software that are central to the research but not yet described in published literature, software must be made available to editors and reviewers. We strongly encourage code deposition in a community repository (e.g. GitHub). See the Nature Portfolio guidelines for submitting code & software for further information.

Data

Policy information about availability of data

All manuscripts must include a data availability statement. This statement should provide the following information, where applicable:

- Accession codes, unique identifiers, or web links for publicly available datasets
- A description of any restrictions on data availability
- For clinical datasets or third party data, please ensure that the statement adheres to our policy

1. The proteomics raw data generated in this study have been deposited in the ProteomeXchange Consortium via the iProX partner repository under accession code PXD051033 [https://www.iprox.cn/page/home.html].
2. Other data generated in this study are provided in the Supplementary Information/Source Data file.

Research involving human participants, their data, or biological material

Policy information about studies with human participants or human data. See also policy information about sex, gender (identity/presentation), and sexual orientation and race, ethnicity and racism.

Reporting on sex and gender	N.A.
Reporting on race, ethnicity, or other socially relevant groupings	N.A.
Population characteristics	N.A.
Recruitment	N.A.
Ethics oversight	N.A.

Note that full information on the approval of the study protocol must also be provided in the manuscript.

Field-specific reporting

Please select the one below that is the best fit for your research. If you are not sure, read the appropriate sections before making your selection.

☒ Life sciences ☐ Behavioural & social sciences ☐ Ecological, evolutionary & environmental sciences

For a reference copy of the document with all sections, see nature.com/documents/nr-reporting-summary-flat.pdf

Life sciences study design

All studies must disclose on these points even when the disclosure is negative.

Sample size	A biological repeats (n ≥ 3) is applied to evaluate significance and to calculate statistical descriptions (mean ± s.e.m.). In vivo studies, the details regarding sample size are provided in the methods section and figure legends. The sample sizes were determined with reference to established guidelines and recommendations summarized in authoritative literature.
Data exclusions	No samples were excluded for all analysis.
Replication	All the studies were replicated three times and the results were consistent across independent experimental runs.s.
Randomization	Animals were randomized into groups. In the evaluation of fluorescence intensity in tissues or cells, microscopes were used to automatically capture images, and random fields of view were selected according to strict predetermined selection protocols. Criteria for skipping views or discarding data were established and validated before data collection. Subsequently, bulk data analysis was performed using image analysis software based on predefined pixel intensity thresholds. Other experiments were not randomized.
Blinding	Blinding was used in the evaluation of animal neurological function. During the allocation and assessment of the remaining experiments, the researchers were not blinded, as most of the experiments involved multiple steps, and the data collection followed standardized criteria.

Reporting for specific materials, systems and methods

We require information from authors about some types of materials, experimental systems and methods used in many studies. Here, indicate whether each material, system or method listed is relevant to your study. If you are not sure if a list item applies to your research, read the appropriate section before selecting a response.

Materials & experimental systems

n/a	Involved in the study
☐	☒ Antibodies
☐	☒ Eukaryotic cell lines
☒	☐ Palaeontology and archaeology
☐	☒ Animals and other organisms
☒	☐ Clinical data
☒	☐ Dual use research of concern
☒	☐ Plants

Methods

n/a	Involved in the study
☒	☐ ChIP-seq
☐	☒ Flow cytometry
☒	☐ MRI-based neuroimaging

Antibodies

Antibodies used	Anti-GFAP primary antibody (Abcam, ab278054), Anti-NeuN primary antibody (Abcam, ab177487), Anti-HDAC5 primary antibody (Abcam, ab55403), Anti-Histone H3 acetylation primary antibody (Abcam, ab300641), Anti-Histone H3 primary antibody (Abcam, ab176842), Anti-GAPDH primary antibody (Abcam, ab181602), goat anti-rabbit IgG Alexa Fluor 488 secondary antibody (Abcam, ab150077), goat anti-rabbit IgG Alexa Fluor 594 secondary antibody (Abcam, ab150080), goat anti-rabbit IgG Alexa Fluor 647 secondary antibody (Abcam, ab150079), goat anti-guinea pig IgG Alexa Fluor 594 secondary antibody (Abcam, ab150188), goat anti-mouse IgG Alexa Fluor 647 secondary antibody (Abcam, ab150115), Rhodamine phalloidin (Abcam, ab235138), Anti-Iba1 primary antibody (Cell Signaling Technology, 17198T), Anti-PSD95 primary antibody (Cell Signaling Technology, 3450S), Anti-SMI-312 primary antibody (Biolegend, 837904), Anti-VGluT1 primary antibody (Synaptic Systems, 135304), Anti-VGAT primary antibody (Synaptic Systems, 131004), Anti-Gephyrin primary antibody (Synaptic Systems, 147011), Anti-mouse CD80-FITC antibody (eBioscience, 11-0801-82) and Anti-mouse CD206-PE (eBioscience, 12-2061-82).
Validation	These antibodies have been verified by the supplier and used by western blot or immunofuorescent staining. All the antibodies used are from commercial sources and validation data are available on the manufacturer's website based on their catalogue numbers. The manufacturers include, Abcam, Cell Signaling Technology, Biolegend, Synaptic Systems and eBioscience.

Eukaryotic cell lines

Policy information about cell lines and Sex and Gender in Research

Cell line source(s)	HT22 cells were kindly provided by Prof. Gao Xiaoling of Shanghai Jiaotong University. BV2 cells were purchased from Chinese Academy of Science Cell Bank (Shanghai, China). These cell lines were cultured in a HERAcell 150i CO2-incubator (Thermo Fisher Scientific) with the Dulbecco's modified Eagle's medium (high glucose, Hyclone) containing 10% fetal bovine serum and 1% penicillin-streptomycin solution.
Authentication	Cell lines were not independently authenticated.
Mycoplasma contamination	Cell lines do not have mycoplasma contamination.
Commonly misidentified lines (See ICLAC register)	No commonly misidentified cell lines were used.

Animals and other research organisms

Policy information about studies involving animals; ARRIVE guidelines recommended for reporting animal research, and Sex and Gender in Research

Laboratory animals	C57BL/6 mice (6–10 weeks old,18–22 g) were purchased from SLAC Animal Ltd. (Shanghai, China) and raised in a pathogen-free facility with a 12 h light and dark cycle at 18-23 °C and 40-60% humidity and had free access to food and water. Primary mouse cortical neurons were extracted from 15-day-old C57BL/6 fetal mice. In terms of animal experiment studies, male mice were chosen. Male mice are less likely to die during the establishment of TBI models based on our historical experience. This can reduce accidental death and help to ensure the objectivity of the studies.
Wild animals	The study did not involve wild animals.
Reporting on sex	Both male and female animals were used as indicated in this study
Field-collected samples	This study did not involve samples collected from the field.
Ethics oversight	All the animal experiments were performed in accordance with the guidelines evaluated and approved by Institutional Animal Care and Use Committee (IACUC), Fudan University School of Pharmacy (Ethical approval number: 2023-03-YJ-CJ-36).

Note that full information on the approval of the study protocol must also be provided in the manuscript.

Plants

Seed stocks	N.A.
Novel plant genotypes	N.A.
Authentication	N.A.

Flow Cytometry

Plots

Confirm that:

☒ The axis labels state the marker and fluorochrome used (e.g. CD4-FITC).

☒ The axis scales are clearly visible. Include numbers along axes only for bottom left plot of group (a 'group' is an analysis of identical markers).

☒ All plots are contour plots with outliers or pseudocolor plots.

☒ A numerical value for number of cells or percentage (with statistics) is provided.

Methodology

Sample preparation	1. Activated BV2 cells (LPS-pretreated) and injured HT22 cells (OGD-pretreated) were seeded at a density of 5000 cells per well in 96-well plates and cultured for 24 hours for attachment. Subsequently, the culture medium was replaced with DMEM containing NeuroConnect Blocks labeled with cou6 or the scaffolds formed by them (at a cou6 concentration of 50 ng/mL), and further incubated for 12 hours at 37°C. Then, BV2 cells were washed with PBS, fixed with 4% paraformaldehyde, and stained with 2 µg/mL Hoechst 33258. The cells were analyzed using flow cytometry (CytoFLEX, Beckman, USA).<br>2. To investigate the in vitro polarization of microglia, BV2 cells were seeded into 6-well plates and stimulated with 100 ng/mL LPS for 24 hours, followed by treatment with PBS (control group), free LMK-235, NeuroConnect Blocks, LMK-235@Bis-5HT/PANI NPs Scaffolds, Bis-5HT/PANI Janus NPs Scaffolds, LMK-235@Bis-5HT Janus NPs Scaffolds and NeuroConnect Blocks Scaffolds (equivalent to a concentration of 11.77 µg/mL LMK-235) for 24 hours. Subsequently, cells were collected and washed with PBS. Anti-mouse CD80 and CD206 antibodies were added separately and incubated for 45 minutes at 4 °C. The cells were then washed with PBS and analyzed using flow cytometry (CytoFLEX, Beckman, USA).
Instrument	flow cytometry (CytoFLEX, Beckman, USA).
Software	FlowJo (Version 10.4)
Cell population abundance	Cells were not sorted.
Gating strategy	Cell debris and dead cells was excluded in a forward/side scatter dot plot (FSC vs. SSC) and the gate was applied to all samples. The median fluorescence intensity (MFI) was determined via a histogram.

☒ Tick this box to confirm that a figure exemplifying the gating strategy is provided in the Supplementary Information.
