# Supplementary figures and images for: Self-assembling scaffolds epigenetically reactivate and electroactively guide neuronal regeneration to restore central neural circuits
Source: Nat Commun. 2026 May 4;17:5987. doi: 10.1038/s41467-026-72397-6 (PMC13347008; doi:10.1038/s41467-026-72397-6)

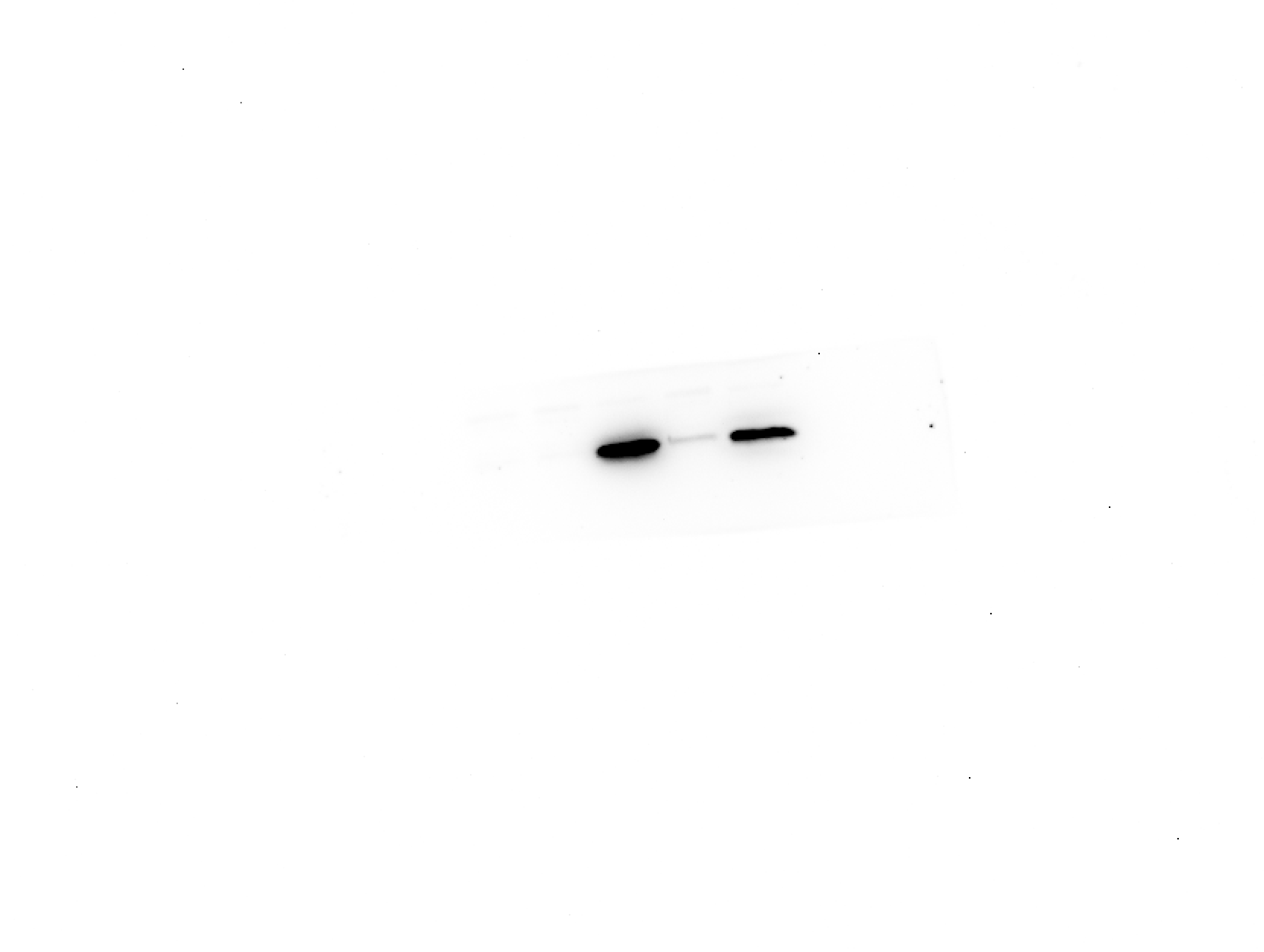

Supplement: Supplementary file 6 — Source data [file 41467_2026_72397_MOESM6_ESM.zip › Source Data_NCOMMS-25-44797C/WB/WB_AC-H3.tif]

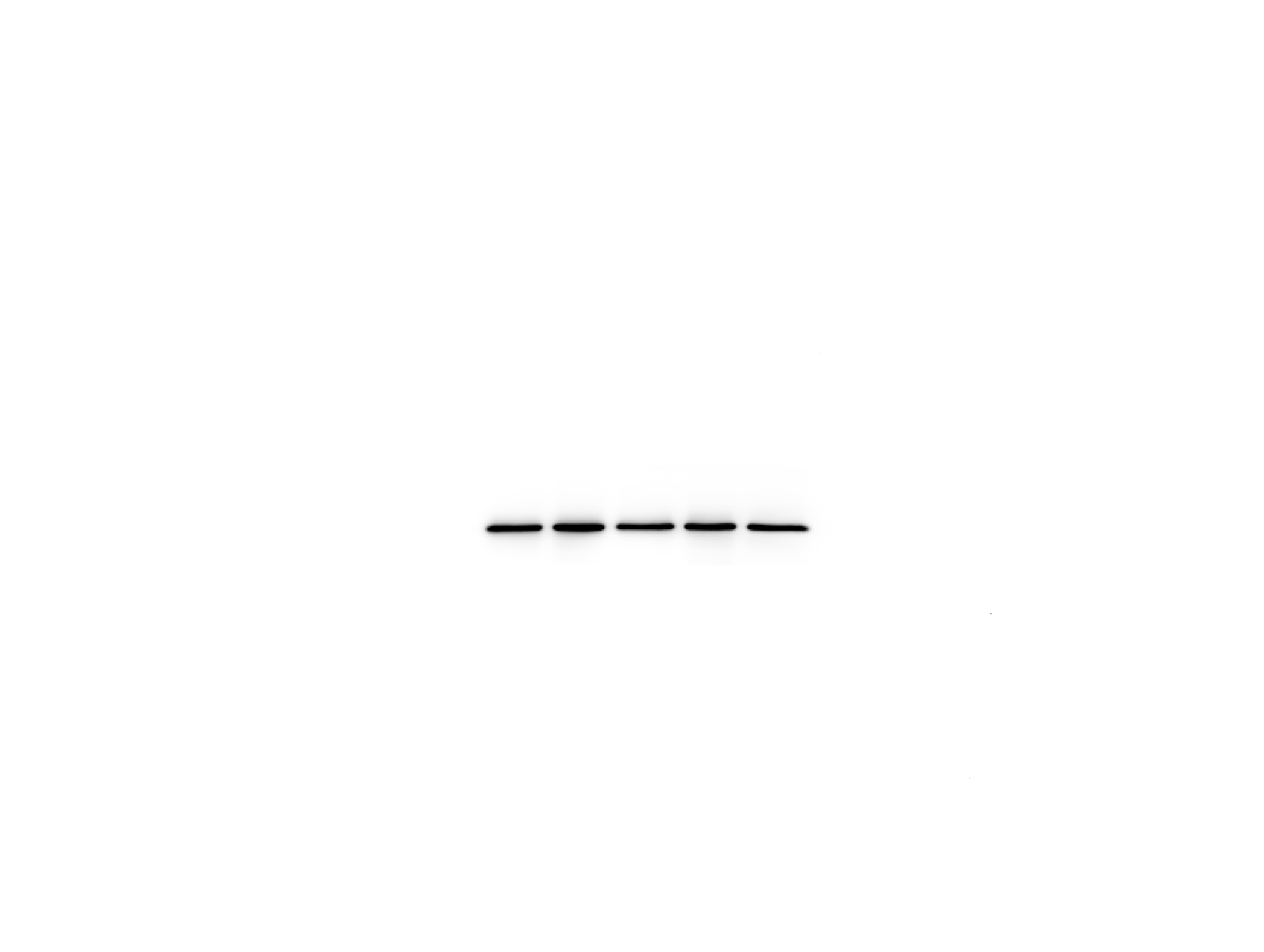

Supplement: Supplementary file 6 — Source data [file 41467_2026_72397_MOESM6_ESM.zip › Source Data_NCOMMS-25-44797C/WB/WB_GAPDH.tif]

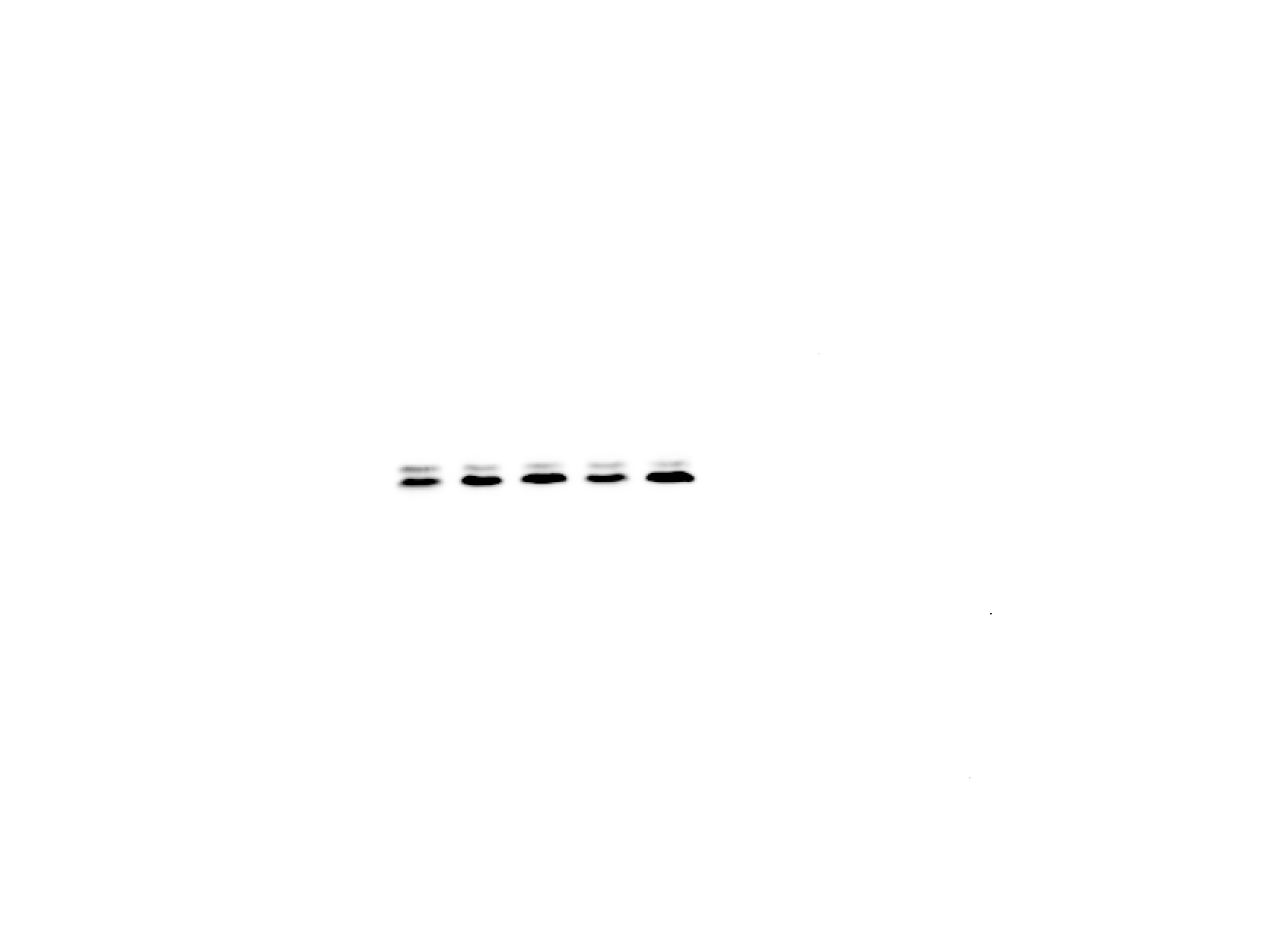

Supplement: Supplementary file 6 — Source data [file 41467_2026_72397_MOESM6_ESM.zip › Source Data_NCOMMS-25-44797C/WB/WB_H3.tif]
