# Supplementary material for: Self-assembling scaffolds epigenetically reactivate and electroactively guide neuronal regeneration to restore central neural circuits
Source: Nat Commun. 2026 May 4;17:5987. doi: 10.1038/s41467-026-72397-6 (PMC13347008; doi:10.1038/s41467-026-72397-6)
Supplement: Supplementary file 5 — Transparent Peer Review file [file 41467_2026_72397_MOESM5_ESM.pdf]

nature portfolio

Peer Review File

Self-assembling scaffolds epigenetically reactivate and electroactively guide neuronal regeneration to restore central neural circuits

Corresponding Author: Professor Jun Chen

This file contains all reviewer reports in order by version, followed by all author rebuttals in order by version.

Version 0:

Reviewer comments:

Reviewer #1

(Remarks to the Author)
Review Nat comm: In this manuscript, the authors develop particles that are uniquely synthesized named (NeuroConnect blocks) with the aim to develop into scaffolds specifically into the Traumatic brain injury region. They load the particles with LMK-235 an HDAC5 inhibitor. They synthesize layer by layer micelles with functional polymers. Every layer serves as a functionalization substrate for the next layer. They add polyaniline as one layer then they add another with a process that coats half of the particles. Finally, they add Au nanoparticles as labelling molecules. Their results show that the particles create porous scaffolds in condition found specifically on the brain injury and in few other organs. They show that the particles become scaffolds based on peroxidase activity in the presence of H2O2. While asymmetric functionalization of half of the particles with Bis… governs the percolation and the porosity of the scaffolds. They show that the particles can delivered to the brain through an impaired BBB layer at TBI mouse models, as well as in other organs, through in vivo fluorescent microscopy. They also show in cell cultures that cells proliferate faster with the NeuroConnec This manuscript has a lot of different techniques and measurements. The number of experiments and data is impressive, and I would even say overwhelming. Based on these results and claims this work can be a significant step towards TBI treatmen Some of their assumptions and claims are not fully clear or convincing see comment 3. Below there are few specific comments

Comments:
1. Are these particles and scaffolds bioresorbable?
2. Can these particles aggregate with other enzymes including cytochrome C? It is possible
3. Page33: Lines730-745, I fail to understand how this experiment shows that PANI facilitates propagation of neuronal signaling. The fact that the authors stop neurons from firing is not directly linked with PANI transmitting the signal. Moreover, genetic modification can alter their plasticity due to non-firing. The NeuroConnect blocks are not directly coupled with an external electronic source to mediate signaling, and PANI acts as a normal non-conducting biopolymer in the ion flow. To my opinion this claim is not properly supported and the authors should address this claim or give a justification why they used PANI or modify their clai A control experiment should show that addition LMK-235@Bis-5HT Janus NPs does not improve the axonal growth and plasticity.

Reviewer #2

(Remarks to the Author)
This original research highlights an interesting multifaceted platform namely “NeuroConnect Blocks” as a therapy approach for CNS regeneration. This platform is designed to be conductive and be delivered intravenously and encapsulate a selective HDAC5 inhibitor (LMK-235). This NeuroConnect Blocks can pass the BBB and form scaffold with large pores that serve multiple functions.
While this study presents a promising strategy, critical data appear to be missing to fully support the authors' claims. The following comments outline key areas that should be addressed.

Major comments:

Methods

1. It would help strengthen the study if some rationale is given on why the injection was performed 6h post injury? It is well known that in the span of 1 week-10 days post injury the inflammatory cells are in their most reactive state. How relevant is this in vivo model for studying the regeneration capacity of the proposed material in the higher intensity of inflammation?

Results

1. From figure 4, the significant higher accumulation of NeuroConnect Blocks in brain as well as main organs is observed. What is the justification for that? What is the off-target side effects? Perhaps to evaluate any potential damage of inflammation within the resected tissues, organ morphology and histopathology assessment is required.
2. Is the release rate of LMK-235 in vivo comparable to in vitro conditions? The authors should consider the microenvironmental differences between in vitro and in vivo conditions.
3. Does the extent of NeuroConnect Block assembly to scaffold affect the LMK-235 release rate?
4. Figure 7 g. The authors stated, “Confocal imaging revealed that NeuroConnect Blocks scaffolds maintained their structural integrity within the injury site and displayed extensive infiltration by regenerating neuronal axons (Fig. 7f-i).” To show the infiltration of cells and confirm the statement the 3D confocal image is required.
5. Figure 8: A) It seems different location of brain is being analysed across different groups. Where in the brain is this stained section from in relation to the site of injection? B) The intensity of the images taken looks differen We expect to see different intensity in Iba-1 and GFAP between different groups however, the DAPI should be the same in images with same intensity and microscopic adjustmen So, please make sure all the images are taken with the same adjustment as the results and quantifications are based on intensity. (for example, in NeuroConnect Block group in 28 days). C) Higher magnification images are required to better visualise the GFAP and Iba-1 cells.
6. What is the degradation profile of the NeuroConnect Blocks scaffold and by what mechanism does it degrade in the body (i.e. enzymatic)?
7. The image quality and resolution in Figures are suboptimal specially for in vivo, making it difficult to observe marker staining.

Reviewer #3

(Remarks to the Author)
I co-reviewed this manuscript with one of the reviewers who provided the listed reports. This is part of the Nature Communications initiative to facilitate training in peer review and to provide appropriate recognition for Early Career Researchers who co-review manuscripts.

Reviewer #4

(Remarks to the Author)
Tong et al present in their manuscript a new approach using targeting nanoparticles for drug delivery to restore neural functions after traumatic brain injury (TBI). The authors support the studies of this new drug delivery system with substantial experiments both in vitro and in vivo. The main contribution to the field of this manuscript is the elegant use of elevated Myeloperoxidase levels at injury sites to self-assemble the nanoparticles providing enhanced local retention and delivery of the encapsulated drug. This could potentially be of interest to a general public, but many studies have shown the accumulation of nanoscale drug carriers in the CNS after TBI or the central effects of their cargoes. For example, Li et al Sci Adv 2021 BBB pathophysiology–independent delivery of siRNA in traumatic brain injury or the recent review by McLoughlin et al 2024 and ref in there Overcoming the Blood–Brain Barrier: Multifunctional Nanomaterial-Based Strategies for Targeted Drug Delivery in Neurological Disorders - McLoughlin - 2024 - Small Science - Wiley Online Library .
The manuscript is very extensive and the authors should be more selective and focus on the most relevant results.
In addition, the synthesis described of the nanocarriers are complex and included multiple intermediates and there is a lack of details and inadequate characterization of intermediates and the final nanocarrier (more details below) which makes it difficult to validate the in vitro and in vivo results.
The authors claim that the encapsulated drug LMK-235 is a selective HDAC 5 inhibitor and conclusions at several places in the manuscript are drawn based on this fac However, LMK-235 is equipotent on HDAC4. Other isoforms of HDACs although 10-100 fold lower potency might also contribute considering the local application and nanomolar potency. For inhibitor profile of LMK-235 see https://www.medchemexpress.com/LMK-235.html?srsltid=AfmBOopA1cuwzkN6saYlQ4eJhO4ydSzxB7IkxyzlcOhw6r2cisRm2wmI
Either the authors need to demonstrate that it is the HDAC5 inhibitory effect that contribute to the regeneration or rephrase throughout the manuscrip
The nanocarrier is reported to be approximately 200 n Previous studies have found that nanoparticles around 30 nm are optimal for TBI application and 80 nm remains in the blood vessel. Could the authors comment on this. See Size-Selective Transfer of Lipid Nanoparticle-Based Drug Carriers Across the Blood Brain Barrier Via Vascular Occlusions Following Traumatic Brain Injury
Specific comments
The method section for the synthesis and characterizations of the intermediates and nanocarrier is not complete and lacks important information that enables reproducibility.
The details for synthesis of control nanoparticles lacking critical features are inadequate
No/limited references to methods used in the synthesis of the nanocarrier
There is no reported yield for the first, PANI step and the final nanocarrier despite isolating the particles.

What is hydrogen peroxide enzyme in the PANI step?
The authors report conductivity of the nanoparticles in figure 2D but does not describe the procedure neither do they show that the conductivity of the PANI layer is crucial for the system considering that the title include “electrically guide..”.
And the authors claim that the nanocarrier contain a conductive polyaniline layer proven by a change in zeta potential and the analysis of conductivity described in figure 2D. The reported conductivity (50-60 mS/cm) is only slightly above CSF (15-20) and blood (5-10) therefore it is hard to understand how this limited conductivity contribute.
But one important aspect is that the negative zeta potential is reduced approaching 0 and it is well known that this will increase the likelihood of aggregation and most likely increase tissue adherence. Both could potentially be important for retention at the site of injury and neuron adherence.
Bis-5HT-Glu-NH2 is not properly characterized only with MS (supp info). LC/1H/13C NMR should be added to verify purity.
The authors covalently adds gold nanoparticles using NHS chemistry. TEM images shows asymmetric distribution on selected particles, but a composition analysis based on elemental analysis with ICP or surface plasmon analysis to quantify % gold is necessary to confirm partial distribution for all particles.
In addition to TEM (selected particles) and DLS (size), it would be beneficial with composition analysis of the final nanocarrier and the control nanocarrier described in table 2 supp.
The authors claim substantial aggregation in vitro using MPO/hydrogen peroxide in figure 3a/b DLS and ocular analysis. However, the DLS technique does not scale with size thus it is not possible to compare peak sizes. Scattered light intensity scales with the sixth power of particle diameter, meaning larger particles dominate the signal, even if they’re rare. This leads to overrepresentation of large particles in the size distribution. Thus, further analysis to quantify the aggregation is necessary.
In figure 4 c there is an increase of the signal in the head of the mouse but nowhere else. In 4e most of the material is in other organs foremost the liver? Could the authors comment on this. It would be relevant to see the distribution in main organs at earlier time points (0-48 h).
Since the nanocarriers mainly end up in the liver, would it not be relevant to perform toxicity test on liver cells.

Version 1:

Reviewer comments:

Reviewer #1

(Remarks to the Author)
I would like to appreciate that the authors replied and modified the manuscript according to the comments. I have seen their responses and modifications. However, I still recommend that the authors modify their statements in which they mention that "conductive" PANI is crucial for neuronal conduction in this case, because in that case a reader will assume that the property that PANI is mixed conductor is necessary for the neuronal signal to propagate. If the authors claim this, to my understanding this is wrong, or not proved in this manuscrip In the response they mention that in Fig 5 the Janus NPs show decreased neuronal growth compared to Neuroconnect blocks, but the difference is non significant (ns) as they mention. Moreover, figure 9 shows the behavioral tests in which there is some significance but still both control with Janus and Neuroconnects are showing good resutls, and it can't be extrapolated from behavioral studies facilitation of neuronal activity by a conducting PANI layer. Conduction may be facilitated but the claim "facilitated by a conductive PANI layer" can be misleading. A simple fix to the claim is to remove the "conductive" term from their claim and mention that Neuroconnect blocks seem to not prevent neuronal signaling, possibly due to incorporation of PANI.

Reviewer #2

(Remarks to the Author)
All changes have been appropriately addressed and significantly approved the manuscrip

Reviewer #3

(Remarks to the Author)
I co-reviewed this manuscript with one of the reviewers who provided the listed reports. This is part of the Nature Communications initiative to facilitate training in peer review and to provide appropriate recognition for Early Career Researchers who co-review manuscripts.

Reviewer #4

(Remarks to the Author)
Response to the revised manuscript Self-assembling scaffolds epigenetically reactivate and electrically guide neuronal regeneration to restore central neural circuits NCOMMS-25-44797A

1. The manuscript is very extensive and the authors should be more selective and focus on the most relevant results.

Responses: We sincerely appreciate your kind advice. We have reorganized the data according to your suggestions, making the manuscript more selective and focused on the relevant results while preserving its overall integrity. Specifically, we have included the in vitro results demonstrating the ability of NeuroConnect Blocks scaffolds to resist rapid cellular uptake and clearance in the Supplementary Information, in order to enhance the focus of the experimental findings

presented in the main manuscript (Supplementary Fig. 14).

Comment: The authors have not reduced the manuscript to a comprehensible forma In fact, only limited changes to the original manuscrip The manuscript is still 49 pages and contain 12 figures. They have not adhered to the commen This needs to be substantially reduced and figures moved to SI section.

2. Either the authors need to demonstrate that it is the HDAC5 inhibitory effect that contribute to the regeneration or rephrase throughout the manuscrip

Responses: We sincerely appreciate your highly professional and important comments. You correctly pointed out that LMK-235 exhibits comparable inhibitory activity against HDAC4 and HDAC5, and that other HDAC isoforms may carry potential off-target risks at locally elevated concentrations. We fully agree with your view. In response to your comment, we have taken the following steps: 1. Comprehensive revision of the manuscript: We have carefully revised the relevant statements throughout the manuscript, avoiding absolute terms such as "selective HDAC5 inhibitor". Instead, we now employ more precise and rigorous descriptions (Location of Changes: Line 41 in page 2; Line 105-106 in page 4; Line 359 in page 15; Line 725 in page 34; Line 990 in page 46). In addition, we have added a dedicated paragraph in the Discussion to specifically address the issue of LMK-235 selectivity, transparently analyzing the correlation between its inhibitory activity on HDAC5 and the observed outcomes in neural regeneration (Location of Changes: Line 1022-1027 in page 47; Line 1028-1030 in page 48). 2. Inference regarding the role of HDAC5: Although LMK-235 demonstrates similar inhibitory activity against HDAC4 and HDAC5, we infer that HDAC5 inhibition plays a more critical role in our model based on the following considerations: A) Evidence from the literature: Multiple independent studies have directly linked the loss or inhibition of HDAC5 with enhanced axonal regeneration and neuronal survival, supporting HDAC5 as a more specific regulatory target for neural regeneration28-30. B) Specificity within our model: Our experimental data show that LMK-235 treatment induces distinct phenotypes—such as enhanced axonal regeneration and upregulation of HDAC5-associated transcription factors including Jun, Fos, and Klf6. These phenotypes closely align with those reported for HDAC5 loss-of-function, rather than HDAC428. C) Concentration considerations: The dosing regimen we employed was carefully designed to achieve effective drug concentrations in the target tissue within the low nanomolar range. At this concentration, the inhibitory effects of LMK-235 on other isoforms are expected to be negligible. Therefore, the main off-target concern lies in the inability to clearly distinguish between HDAC4 and HDAC5. On this basis, we have incorporated additional discussion in the manuscript to strengthen the rigor of the study (Location of Changes: Line 1022-1027 in page 47; Line 1028-1030 in page 48). Once again, we are deeply grateful for this constructive feedback, which has greatly helped us improve the scientific robustness of our work.

Comment: The authors' claim that HDAC5 inhibition specifically drives the observed neural regeneration phenotypes is not adequately supported. While the cited literature (Cho & Cavalli, 2013) demonstrates HDAC5 role in peripheral nerve regeneration, these studies did not test whether HDAC4 inhibition would produce similar effects. Given that LMK-235 inhibits HDAC4 and HDAC5 with comparable potency, HDAC4 and HDAC5 both repress MEF2-dependent transcription including immediate early genes, and recent work demonstrates functional redundancy between HDAC4 and HDAC5 in neuronal gene regulation (Zhu et al., 2019), the claim that the observed effects are HDAC5-specific requires direct experimental validation or the use of HDAC4-selective inhibitors such as Tasquinimod.
References for support
Fitzsimons HL, Schwartz S, Given FM, Scott MJ. The histone deacetylase HDAC4 regulates long-term memory in Drosophila. PLoS One. 2013 Dec 9;8(12):e83903. doi: 10.1371/journal.pone.0083903. PMID: 24349558; PMCID: PMC3857321.
Zhu, Y., Huang, , Bushong, E. et al. Class IIa HDACs regulate learning and memory through dynamic experience-dependent repression of transcription. Nat Commun 10, 3469 (2019). https://doi.org/10.1038/s41467-019-11409-0
Zhu et al demonstrate that HDAC4 and HDAC5 redundantly act in the same pathway that is essential for spatiotemporal control of ERG programs.
Sando R 3rd, Gounko N, Pieraut S, Liao L, Yates J 3rd, Maximov A. HDAC4 governs a transcriptional program essential for synaptic plasticity and memory. Cell. 2012 Nov 9;151(4):821-834. doi: 10.1016/j.cell.2012.09.037. PMID: 23141539; PMCID: PMC3496186.

And the authors revision from selective HDAC5 inhibitor to HDAC5 inhibitor does not change the interpretation by the reader. HDAC5 is mentioned 36 times and HDAC4 two times in the manuscript,
Since the authors argue that the HDAC5 inhibition is the most important pharmacological effect of LMK-235, I suggest experimental validation to confirm this view.

3. The nanocarrier is reported to be approximately 200 n Previous studies have found that nanoparticles around 30 nm are optimal for TBI application and 80 nm remains in the blood vessel. Could the authors comment on this. See Size-Selective Transfer of Lipid Nanoparticle-Based Drug Carriers Across the Blood Brain Barrier Via Vascular Occlusions Following Traumatic Brain Injury

Responses: Thank you for your valuable comments. The findings reported in the reference you provided were obtained using a moderate TBI model (impact velocity: 6 m/s, impact depth: 0.5 mm, contact time: 150 ms), whereas our study employed a severe TBI model (impact velocity: 3.5 m/s, impact depth: 1 mm, contact time: 400 ms). The degree of BBB permeability following TBI is largely determined by the extent of both primary and secondary injury31. Therefore, given the more severe injury model used in our study, it is entirely plausible that NeuroConnect Blocks particles of approximately 200 nm in size were able to penetrate the BBB and accumulate within the brain. Moreover, dedicated studies have demonstrated that PLGA nanoparticles ranging from 100 nm to 800 nm are capable of accumulating in the brain after TBI32.

Comment: The authors claim that their model produces a more severe injury, yet the reported impact velocity is lower. It would be helpful to clarify how a reduced velocity leads to increased severity, given that velocity is typically a major contributor of impact energy. Are there other parameters such as impact depth, dwell time, or tip geometry that could account for the heightened injury? It may also strengthen the argument to confirm the severity using established biomarkers of tissue damage or neuroinflammation.

4. What is hydrogen peroxide enzyme in the PANI step?

Responses: We appreciate your feedback. In our study, hydrogen peroxide enzyme was employed to catalyze the in situ polymerization of aniline. Under the presence of H_2O_2, the heme cofactor ($Fe3+$) of catalase is first oxidized by H2O2 to generate a highly reactive intermediate. This intermediate can abstract an electron from an aniline molecule, forming an aniline radical cation, which in turn efficiently promotes the in situ polymerization of aniline to yield PANI33. Accordingly, we have incorporated the relevant explanation and references into the manuscript (Location of Changes: Line 1180-1185 in page 53).

Comment: There are several enzymes that use or degrade H2O2 thus the expression hydrogen peroxide enzyme is unspecific. State the enzyme and include the quality+vendor in the materials.
For the generation of PANI the most common is HRP and multiple references cover this process. The author claim that they use catalase which is degrading hydrogen peroxide to water which is unusual and with limited suppor The mechanism describe in the response by the authors that abstracts electrons from a substrate is more characteristic of peroxidases like HRP, not catalase.

5. The authors report conductivity of the nanoparticles in figure 2D but does not describe the procedure neither do they show that the conductivity of the PANI layer is crucial for the system considering that the title include “electrically guide..”.

Responses: Thank you very much for the kind suggestion. We have incorporated the procedural details of the conductivity testing (Location of Changes: Line 1223-1229 in page 54). Furthermore, in our design, the conductivity of the PANI layer is essential to the syste Mechanistically, the scaffolds formed by NeuroConnect Blocks promote neural circuit repair primarily through two steps: (1) attracting central neurons to grow directionally along the scaffold via the concentration gradient of LMK-235, and (2) facilitating synaptic reconnection of the recruited central neurons within the scaffold channels through neural signal transmission mediated by the conductive PANI layer. Experimentally, we established control groups in both in vitro or in vivo neuronal synaptic reconnection assays and behavioral tests to demonstrate the critical role of the PANI conductive layer in the system (Fig. 5, 7, 9, 11; Supplementary Figs. 22, 23, 27- 29, 43-45). In addition, by overexpressing Kir2.1 in neurons to suppress their electrical activity, we directly confirmed that the scaffold’s synaptic reconstruction effect is partly dependent on electrical signal conduction mediated by the PANI conductive layer (Fig. 10i-o; Supplementary Figs. 57, 58).

Comments: The authors has still not convincingly demonstrated that it is the conductivity and not the surface properties of PANI that are importan The authors nicely provide analysis of zeta potential which clearly show that the PANI layer provide a neutral/positive surface. The positive surface have been reported to promote neuronal adhesion, neurite outgrowth, and cell spreading.

6. And the authors claim that the nanocarrier contain a conductive polyaniline layer proven by a change in zeta potential and the analysis of conductivity described in figure 2D. The reported conductivity (50-60 mS/cm) is only slightly above CSF (15-20) and blood (5-10) therefore it is hard to understand how this limited conductivity contribute.

Responses: We greatly appreciate your professional comments. The point you raised is indeed critical. Admittedly, in terms of absolute values, the nanocarriers increase conductivity only from 5–20 mS/cm (in body fluids) to 50–60 mS/cm, which may appear to be a modest improvemen However, the core function of the NeuroConnect Blocks design in this study is not merely to elevate bulk conductivity, but rather to direct neural regeneration through the construction of an anisotropic, macroscale conductive architecture. To enhance the reader’s understanding, we have incorporated the relevant elaboration into the manuscript (Location of Changes: Line 178-180 in page 7)…more

Comment: The authors change from conductivity matters to anisotropic architecture matters but these are different claims. If the architecture is what is doing the work, then why emphasize conductivity in the title and throughout the paper? They are essentially conceding the conductivity might not be functionally significant while trying to make it sound like they planned it that way.
The numbers problem remains PANI layer 50–60 mS/cm vs CSF 15–20 mS/cm and Blood 5–10 mS/c Is it conductivity or surface charge that promote neuronal adhesion, neurite outgrowth, and cell spreading?

7.But one important aspect is that the negative zeta potential is reduced approaching 0 and it is well known that this will increase the likelihood of aggregation and most likely increase tissue adherence. Both could potentially be important for retention at the site of injury and neuron adherence.

Responses: Thank you very much for the professional comments and kind advice. We fully agree that, under typical

circumstances, a near-neutral Zeta potential markedly reduces colloidal electrostatic stability, thereby leading to aggregation. However, in the nanoparticle design of this study, we circumvented this issue through a steric stabilization mechanism: As described in the manuscript, the nanoparticle surfaces were densely functionalized with long, hydrophilic PEG2000 and PEG5000 chains. These PEG chains extend fully in aqueous environments to form a hydrated layer, which generates substantial steric repulsion between nanoparticles and thereby effectively prevents aggregation. This strategy is widely employed in nanomedicine to ensure colloidal stability under physiological conditions34-39. Moreover, the uniform particle size distribution shown in Fig. 2b directly demonstrates that the nanoparticles remained well-dispersed after preparation, without evidence of aggregation (Fig. 2b).

Comments: If their PEGylation is so effective at preventing all aggregation and tissue interaction, then how do the nanoparticles stay at the injury site? How do neurons adhere to the scaffold? What is the actual mechanism of interaction with neural tissue?

8. In figure 4 c there is an increase of the signal in the head of the mouse but nowhere else. In 4e most of the material is in other organs foremost the liver? Could the authors comment on this. It would be relevant to see the distribution in main organs at earlier time points (0-48 h).

Responses: We sincerely appreciate your insightful questions and constructive feedback. The exclusive enhancement of fluorescence in the mouse head is attributable to the greater ability of near-infrared signals to penetrate and reach brain tissue compared with other organs, thereby generating detectable fluorescence. The observed increased accumulation of NeuroConnect Blocks across all organs is likely due to their enhanced circulation time in the bloodstream, as demonstrated by our pharmacokinetic evaluation (Supplementary Fig. 16). A detailed explanation is provided as follow: 1. The reason for the exclusive enhancement of fluorescence in the mouse head: The enhanced fluorescence observed in the mouse head, but not in other regions, can be attributed to two factors: first, the monitoring was performed with the mouse in a prone position; second, the fur on the head had been shaved for surgical procedures. Under these conditions, near-infrared fluorescence penetrates more readily and reaches the brain compared with other organs. Consequently, at the applied fluorescence threshold, only signals from the head are detectable, primarily reflecting the accumulation of nanoparticles in the brain. 2. The reason for the increased accumulation of NeuroConnect Blocks across all organs: In comparison to the control formulation, NeuroConnect Blocks were modified with a PEG2000 segment (it was introduced by SH-PEG2000-NHS), which likely enhanced their circulation, contributing to increased accumulation in various organs. To demonstrate the long-circulation capability of NeuroConnect Blocks, we have conducted a pharmacokinetic evaluation. The results indicated that the plasma half-life of NeuroConnect Blocks was significantly prolonged compared to the control nanoparticles (Supplementary Fig. 16). Supplementary Fig. 16 The pharmacokinetic properties of NeuroConnect Blocks scaffolds (n = 3). Data represent mean ± SD of three independent biological replicates.

Comment: If NIR penetration was the issue, you would still expect some signal from other superficial tissues. More importantly, if you can not detect signal from other organs in the live imaging, how can you claim brain-specific targeting? The head signal could be skin, skull, or vasculature, not necessarily brain parenchyma
And early time point data for organ distribution has not been provided. So what fraction actually reaches the brain? What is the brain-to-liver ratio? Is the brain accumulation therapeutically meaningful or just background? If most material goes to liver and other organs, what evidence supports that enough reaches the injured spinal cord/brain to have the claimed therapeutic effects?

9- Other comments concerning method section and characterization have been addressed.

Version 2:

Reviewer comments:

Reviewer #4

(Remarks to the Author)
The authors have satisfactorily addressed my critical points, toned down their claims, and performed additional experiments that strengthen the manuscript and lend reasonable support to their conclusions. I am now positive toward the current version, and no further revision is needed.
That said, the manuscript remains substantial, eight figures and over twenty pages, and I maintain my reservations about whether such extensive articles will find a broad readership.

Open Access This Peer Review File is licensed under a Creative Commons Attribution 4.0 International License, which permits use, sharing, adaptation, distribution and reproduction in any medium or format, as long as you give appropriate credit to the original author(s) and the source, provide a link to the Creative Commons license, and indicate if changes were made.
In cases where reviewers are anonymous, credit should be given to 'Anonymous Referee' and the source.
The images or other third party material in this Peer Review File are included in the article’s Creative Commons license, unless indicated otherwise in a credit line to the material. If material is not included in the article’s Creative Commons license and your intended use is not permitted by statutory regulation or exceeds the permitted use, you will need to obtain permission directly from the copyright holder.
To view a copy of this license, visit https://creativecommons.org/licenses/by/4.0/

Point-by-Point Response to Reviewer Comments

Manuscript Number: NCOMMS-25-44797

Title: Self-assembling scaffolds epigenetically reactivate and electrically guide neuronal regeneration to restore central neural circuits

Reviewer #1 (Remarks to the Author):

In this manuscript, the authors develop particles that are uniquely synthesized named (NeuroConnect blocks) with the aim to develop into scaffolds specifically into the Traumatic brain injury region. They load the particles with LMK-235 an HDAC5 inhibitor. They synthesize layer by layer micelles with functional polymers. Every layer serves as a functionalization substrate for the next layer. They add polyaniline as one layer then they add another with a process that coats half of the particles. Finally, they add Au nanoparticles as labelling molecules. Their results show that the particles create porous scaffolds in condition found specifically on the brain injury and in few other organs. They show that the particles become scaffolds based on peroxidase activity in the presence of H_2O_2. While asymmetric functionalization of half of the particles with Bis… governs the percolation and the porosity of the scaffolds. They show that the particles can delivered to the brain through an impaired BBB layer at TBI mouse models, as well as in other organs, through *in vivo* fluorescent microscopy. They also show in cell cultures that cells proliferate faster with the NeuroConnect. This manuscript has a lot of different techniques and measurements. The number of experiments and data is impressive, and I would even say overwhelming. Based on these results and claims this work can be a significant step towards TBI treatment. Some of their assumptions and claims are not fully clear or convincing see comment 3. Below there are few specific comments.

Comments:

1. Are these particles and scaffolds bioresorbable?

Responses: We greatly appreciate the reviewer for the professional comments and questions. We have assessed the degradation of the NeuroConnect Blocks scaffolds, and the results indicate that they degrade slowly in a simulated physiological environment *in vitro*, with approximately 98.15% degradation observed over 120 days

(Supplementary Fig. 15).

The nanoparticles and scaffolds are primarily composed of PLGA, polyaniline, and bis-5HT. The degradation products of PLGA are lactic acid and glycolic acid, which are also intermediates of normal human metabolic pathways. Consequently, when applied in pharmaceuticals and biomaterials, PLGA does not elicit toxic side effects and is therefore regarded as a bioresorbable material[1-3]. Polyaniline (PANI) itself exhibits chemical stability under physiological conditions, owing to its conjugated backbone and the absence of hydrolyzable bonds, rendering it poorly degradable and absorbable *in vivo*. However, when PANI is incorporated with biodegradable materials—for instance, in this study, through the in situ polymerization of aniline monomers on the nanoparticle surface to form a core–shell biodegradable polymer—PANI can be eliminated from the body via "bioerosion". Specifically, as the biodegradable matrix undergoes gradual hydrolysis, the nanostructure of PANI loses its supporting framework and breaks down into smaller fragments. Although PANI cannot undergo molecular-level degradation, these nanoscale fragments can be cleared by phagocytic cells (e.g., macrophages) and eventually excreted through renal or biliary pathways. Consequently, despite its intrinsic non-degradability, PANI demonstrates favorable biocompatibility and safety, supporting its broad application in biosensing, neural engineering, and tissue engineering[4,5]. The primary degradation product of bis-5HT is serotonin (5-hydroxytryptamine), an endogenous compound in the body, and is therefore considered bioresorbable[6,7].

In summary, these particles and scaffolds are either bioresorbable or amenable to clearance.

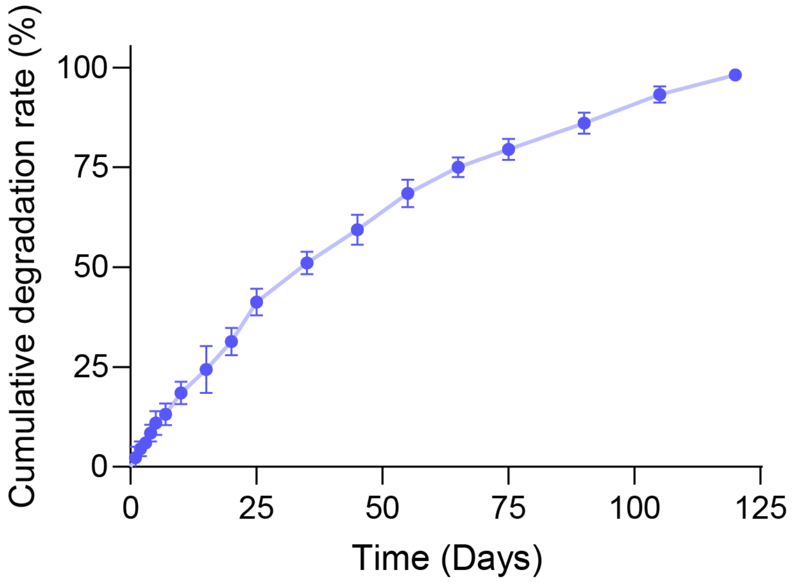

Supplementary Fig. 15 The degradation curve of NeuroConnect Blocks scaffolds (n = 3). Data represent mean ± SD of three independent replicates.

2. Can these particles aggregate with other enzymes including cytochrome C? It is possible

Responses: We thank you very much for the kind advice. Indeed, as you noted, nanoparticles may also undergo aggregation catalyzed by other enzymes including cytochrome C[8]. However, MPO exhibits catalytic activity far exceeding that of cytochrome C and related enzymes, thereby playing a dominant role in nanoparticle aggregation[9-11]. Based on your suggestion, we have included a comprehensive discussion in the relevant sections of the revised manuscript (***Location of Changes:*** *Line 1049-1056 in page 48; Line 1057-1060 in page 49*).

The specific discussion is presented as follows:

"Notably, NeuroConnect Blocks may also undergo aggregation catalyzed by other enzymes with peroxidase activity. For example, under inflammatory conditions, cells may release cytochrome C into the extracellular space, where, in the presence of H_2O_2, cytochrome C can catalyze the oxidative crosslinking of biogenic amines[8]. However, the intrinsic peroxidase activity of cytochrome C is exceedingly low, requiring millimolar (mM) concentrations of H_2O_2, which are far above the pathological levels (nM–μM) observed following TBI[12-14]. Myeloperoxidase (MPO), which is actively

secreted by activated neutrophils and macrophages at sites of inflammation to eliminate damaged tissue, is the most catalytically potent member of the peroxidase family[15,16]. MPO reacts instantaneously with substrates such as 5-HT, exhibiting catalytic activity far exceeding that of cytochrome C and related enzymes, and therefore plays a dominant role in NeuroConnect Blocks aggregation[9-11]."

3. Page33: Lines730-745, I fail to understand how this experiment shows that PANI facilitates propagation of neuronal signaling. The fact that the authors stop neurons from firing is not directly linked with PANI transmitting the signal. Moreover, genetic modification can alter their plasticity due to non-firing. The NeuroConnect blocks are not directly coupled with an external electronic source to mediate signaling, and PANI acts as a normal non-conducting biopolymer in the ion flow. To my opinion this claim is not properly supported and the authors should address this claim or give a justification why they used PANI or modify their claim. A control experiment should show that addition LMK-235@Bis-5HT Janus NPs does not improve the axonal growth and plasticity.

Responses: Thank you very much for your insightful comments, which significantly improve both the quality and clarity of our manuscript. We apologize for not having clearly conveyed our point. In fact, we have already included LMK-235@Bis-5HT Janus NPs as a control group in both *in vitro* and *in vivo* assessments of neuronal regeneration. These controls were applied in experiments evaluating the ability of NeuroConnect Blocks to activate and guide axonal growth (Fig. 5), as well as in assessments of their efficacy in promoting long-term neurological functional recovery (Fig. 9). The attenuated effects on axonal growth, synaptic reconstruction and neurological functional recovery observed with LMK-235@Bis-5HT Janus NPs, relative to the NeuroConnect Blocks, serve to demonstrate the critical role of PANI. Furthermore, the overexpression of the Kir2.1 potassium channel in M2 neurons, which suppresses their electrical activity, was specifically employed to further validate that the neurorepair efficacy mediated by NeuroConnect Blocks partially relies on neuronal electrical signal transmission facilitated by the conductive PANI layer (Supplementary

Figs. 57, 58; Fig. 10j-o). Based on your feedback, we have also revised the relevant descriptions in the manuscript to strengthen the rigor of our presentation (***Location of Changes:*** *Line 770-773 in page 35*).

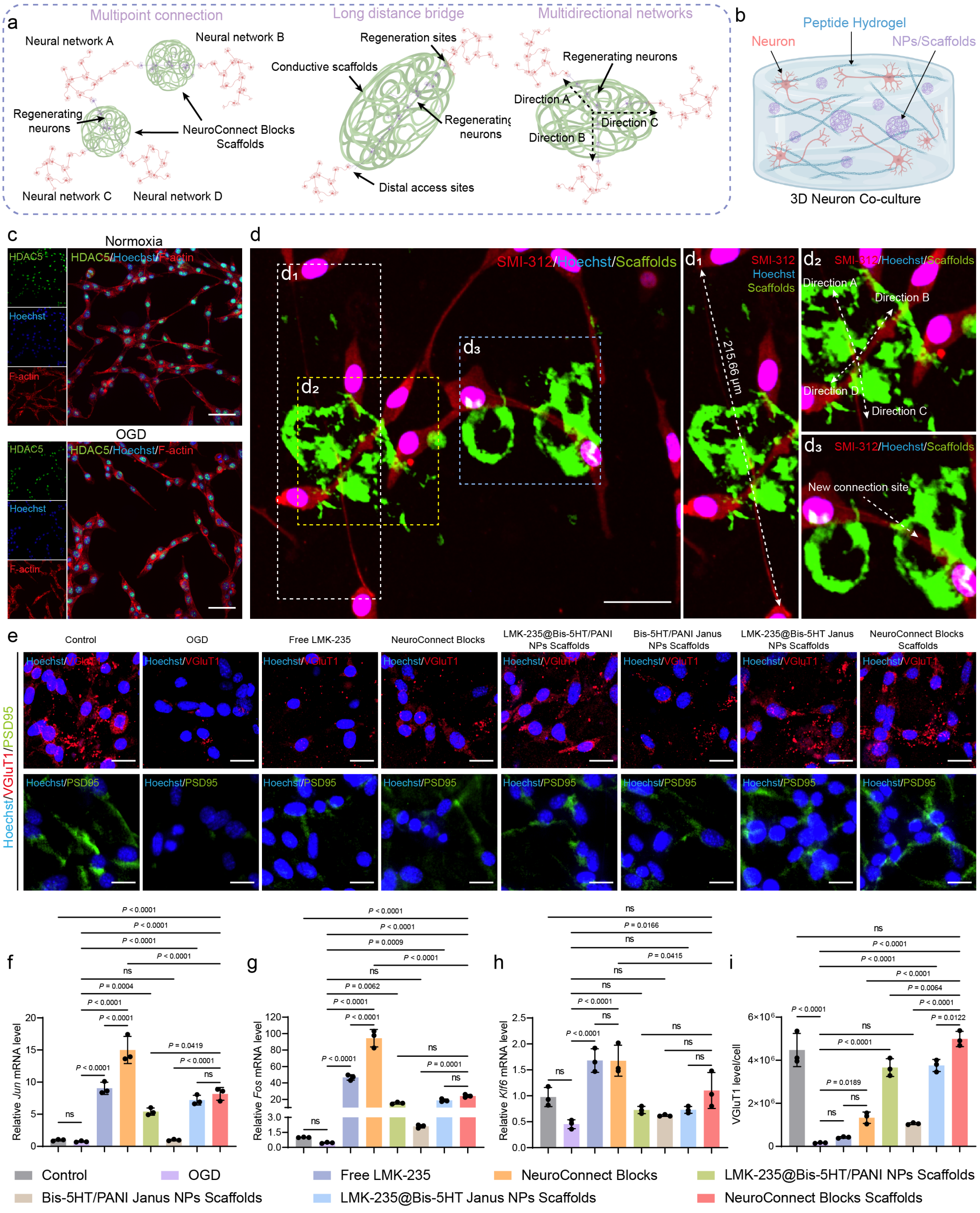

Fig. 5 | NeuroConnect Blocks scaffolds activate and guide axonal growth in vitro. a A schematic representation illustrating the multi-site connections, long-distance bridging, and multi-directional guidance capabilities of the NeuroConnect Blocks scaffolds. Figure created with BioRender.com. **b** Schematic diagram for the 3D co-culture of neurons. Figure created with BioRender.com. **c** Representative images showing the subcellular localization of HDAC5 in HT22 cells before and

after injury. Scale, 50 μm. **d** Representative images illustrating the regenerative guidance effects of NeuroConnect Blocks scaffolds, including (**d1**) long-distance bridging, (**d2**) multi-directional guidance and (**d3**) multi-site connections. Scale, 500 μm. **e** Representative images demonstrating the promotive effects of the scaffolds on the formation of synaptic connections (VGluT1 and PSD95) in injured HT22 cells. Scale, 25 μm. **f-h** qPCR analysis of mRNA levels of (**f**) *Jun*, (**g**) *Fos*, and (**h**) *Klf6* in injured HT22 cells ($n = 3$). **i** Semi-quantitative analysis of VGluT1 in injured HT22 cells as shown in (**e**) ($n = 3$). Data represent mean ± SD of three independent biological replicates. Statistical analyses are performed using one-way ANOVA with Tukey's post hoc test.

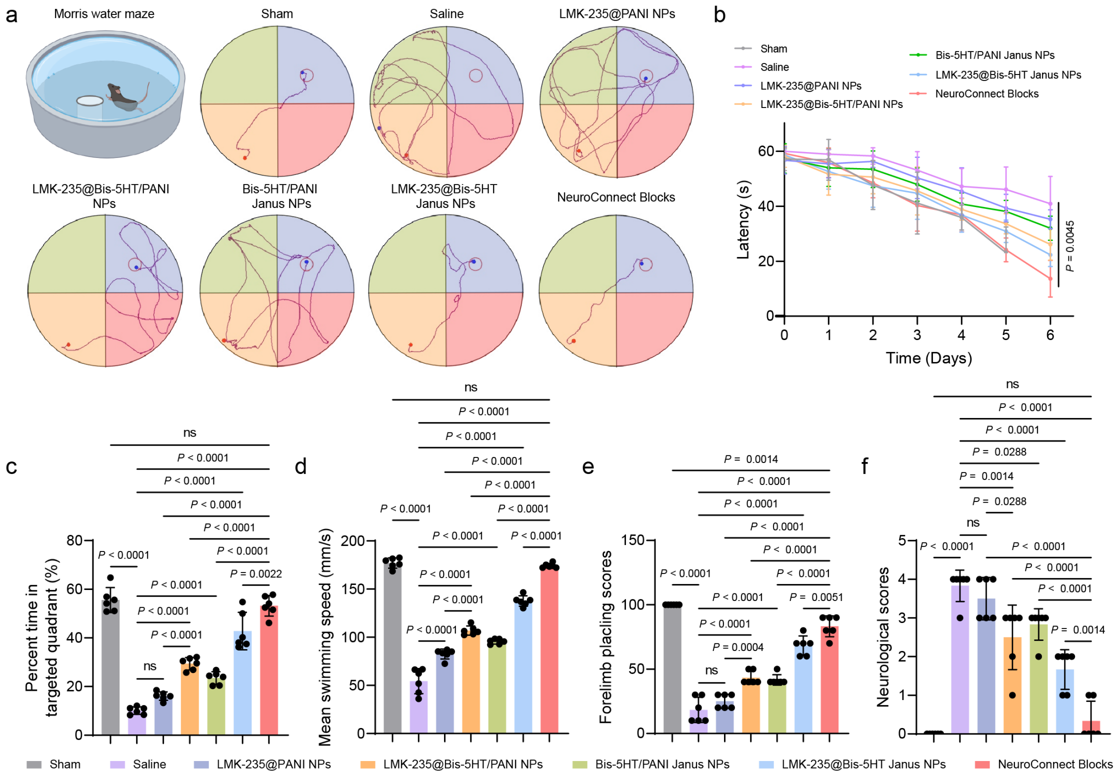

Fig. 9 | NeuroConnect Blocks facilitate the long-term recovery of neurological function. a Representative swimming track of the Morris water maze assays on TBI mice at 28 days post-injury. Schematic diagram of the Morris water maze created with BioRender.com. **b-d** Quantitative analysis from the Morris water maze assays at 28 days post-injury, including (**b**) escape latency over trial days, (**c**) percentage of time spent in the target quadrant, and (**d**) swimming speed ($n = 6$). **e** Forelimb placing scores assessed at 28 days post-injury ($n = 6$). **f** Neurological scores assessed at 28 days post-injury ($n = 6$). Data represent mean ± SD of six independent biological replicates. Statistical analyses are performed using two-way ANOVA for escape latency (**b**) and one-way ANOVA (for **c-f**), followed by Tukey's post hoc test.

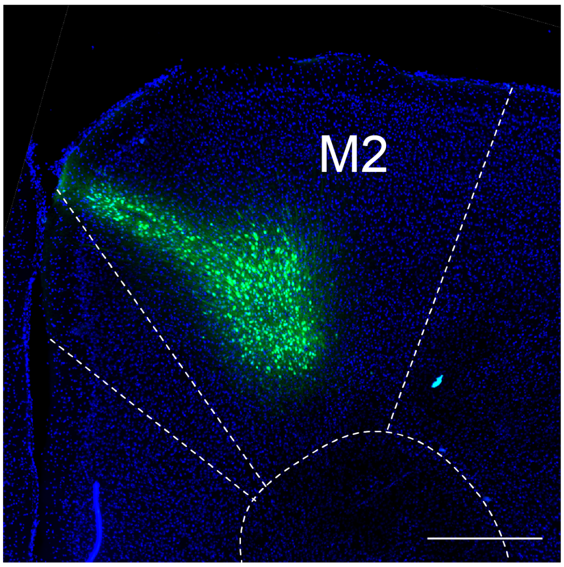

Supplementary Fig. 57 Representative images of the rAVV carrying the Kir2.1 gene transduced the neurons in the M2 region of the motor cortex. Scale, 500 μm.

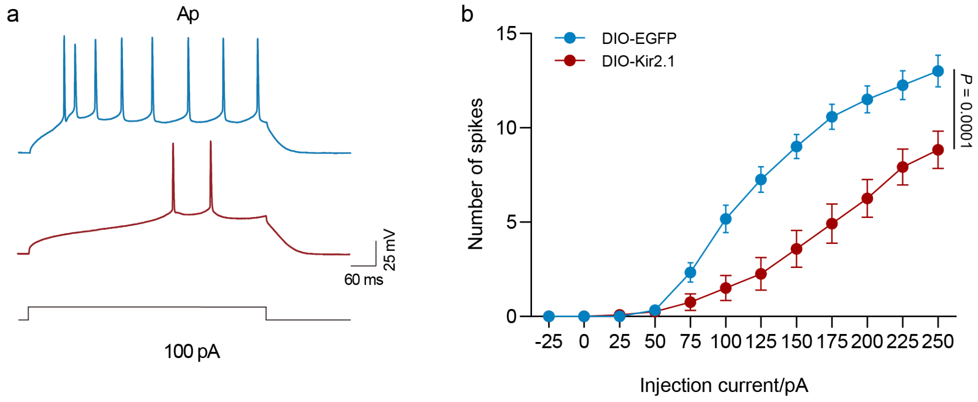

Supplementary Fig. 58 a, b The rAAV-EF1a-Kir2.1-EGFP injection site exhibited differences in (**a**) neuronal action potentials and (**b**) spike counts compared to the control. Data represent mean ± SD of twelve independent biological replicates. Statistical analyses are performed using two-way ANOVA with Tukey's post hoc test.

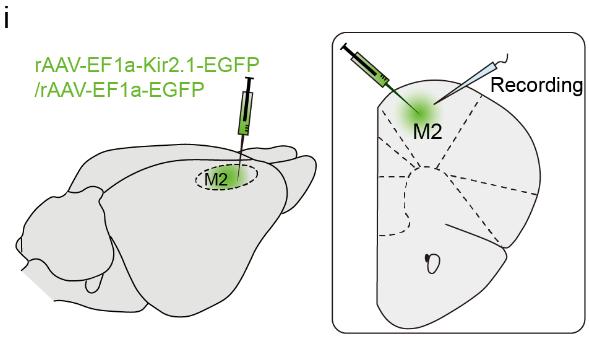

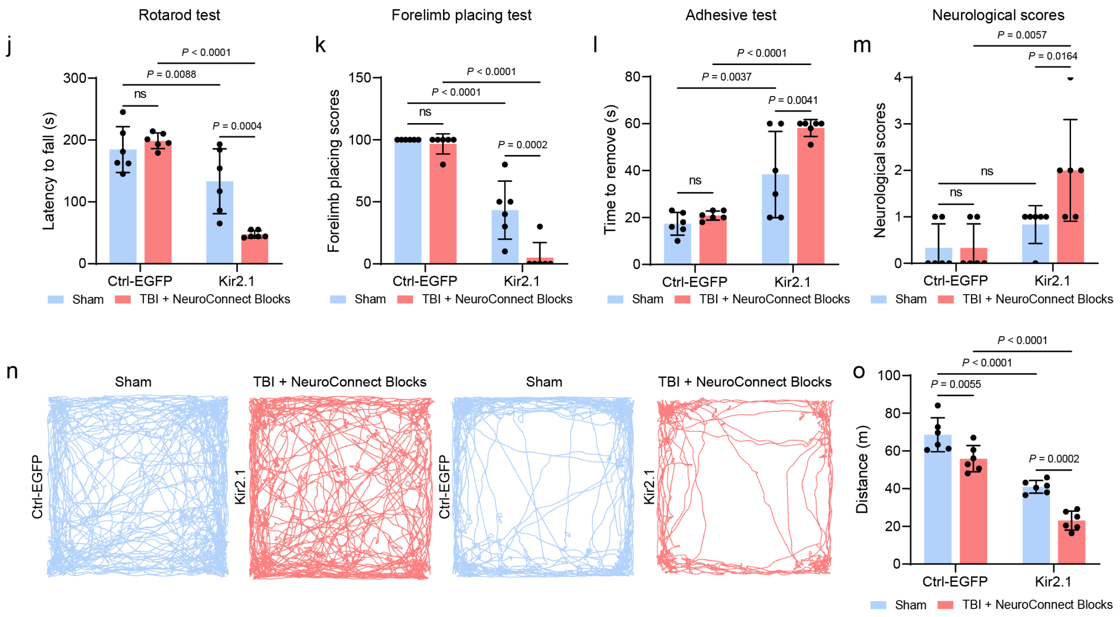

Fig. 10 | Mechanisms by which components of NeuroConnect Blocks activate and guide synaptic reconnection. i Schematic representation of the rAAV-EF1α-Kir2.1-EGFP or rAAV-EF1α-EGFP injection. **j-m** Assessments of (**j**) latency on the rotarod, (**k**) forelimb placing scores, (**l**) time to remove adhesive stickers, (**m**) neurological scores of M2$^{Kir2.1}$ mice and control mice after NeuroConnect Blocks treatment (n = 6). **n** Representative movement track from the open field test. **o** Quantitative analysis of the total distance in open field test (n = 6). Data represent mean ± SD of three or six independent biological replicates. Statistical analyses are performed using one-way ANOVA (for **c-f**) and two-way ANOVA (for **j-m, o**) with Tukey's post hoc test.

Reviewer #2 (Remarks to the Author):

This original research highlights an interesting multifaceted platform namely "NeuroConnect Blocks" as a therapy approach for CNS regeneration. This platform is designed to be conductive and be delivered intravenously and encapsulate a selective HDAC5 inhibitor (LMK-235). This NeuroConnect Blocks can pass the BBB and form scaffold with large pores that serve multiple functions.

While this study presents a promising strategy, critical data appear to be missing to fully support the authors' claims. The following comments outline key areas that should be addressed.

Major comments:

Methods

1. It would help strengthen the study if some rationale is given on why the injection was performed 6h post injury? It is well known that in the span of 1 week-10 days post injury the inflammatory cells are in their most reactive state. How relevant is this *in vivo* model for studying the regeneration capacity of the proposed material in the higher intensity of inflammation?

Responses: Thank you for the professional questions and constructive suggestions. Indeed, although the activation of inflammatory cells peaks approximately 7–10 days after TBI, the administration of NeuroConnect Blocks in this study was scheduled at 6 hours post-injury. This decision was primarily based on four considerations: the time window of BBB disruption, the temporal profile of MPO release, the long-circulating capacity of NeuroConnect Blocks, and the sustained inflammation-modulating effect of the NeuroConnect Blocks scaffold. Accordingly, we have incorporated relevant explanations in the response section of the manuscript (***Location of Changes:*** *Line 292-302 in page 13*). Of course, from a long-term translational perspective, we speculate that administration at other time points may also be effective, which warrants comprehensive evaluation and investigation in future studies.

A detailed explanation is provided as follow:

"We determined the administration time based on the following four considerations: *First*, the BBB undergoes marked disruption within the first week after injury,

facilitating nanoparticle accumulation[17]. *Second*, NeuroConnect Blocks aggregation relies on MPO released by infiltrating neutrophils, which occurs predominantly between 6 and 48 hours post-injury[18-20]. *Third*, following intravenous injection, NeuroConnect Blocks can circulate in the bloodstream for up to 48 hours while continuously accumulating into the brain. *Fourth*, early administration of NeuroConnect Blocks after injury maximizes the attenuation of the inflammatory cascade, while the scaffold formed through NeuroConnect Blocks aggregation serves as a drug reservoir, enabling the sustained release of LMK-235 for prolonged modulation of inflammation[21]."

Results

1. From figure 4, the significant higher accumulation of NeuroConnect Blocks in brain as well as main organs is observed. What is the justification for that? What is the off-target side effects? Perhaps to evaluate any potential damage of inflammation within the resected tissues, organ morphology and histopathology assessment is required.

Responses: Thank you for your insightful comments. The observed increased accumulation of NeuroConnect Blocks across all organs is likely due to their enhanced circulation time in the bloodstream, as demonstrated by our pharmacokinetic evaluation (Supplementary Fig. 16). Accordingly, we have supplemented the study with evaluations of immune cell levels in routine blood tests, biochemical analyses of peripheral organ function, and H&E staining of major organ sections, demonstrating that NeuroConnect Blocks accumulation in peripheral organs does not induce significant inflammation or tissue damage (Supplementary Figs. 46, 47) (***Location of Changes:*** *Line 662-671 in page 31*).

Specifically, to evaluate the potential toxicity of various therapies *in vivo*, we applied the same administration schedule as described above to healthy C57BL/6 mice. After 28 days of NeuroConnect Blocks treatment, no significant changes were observed in white blood cell (WBC) counts, granulocyte counts, lymphocytes counts and monocytes counts between the control and treatment groups (Supplementary Fig. 46a-d). Serum levels of alanine aminotransferase (ALT), aspartate aminotransferase (AST),

blood urea nitrogen (BUN), and creatinine (CREA) showed no appreciable differences (Supplementary Fig. 46e-h). In addition, no discernible structural changes were observed in the hearts, livers, spleens, lungs, or kidneys, confirming the safety of NeuroConnect Blocks (Supplementary Fig. 47).

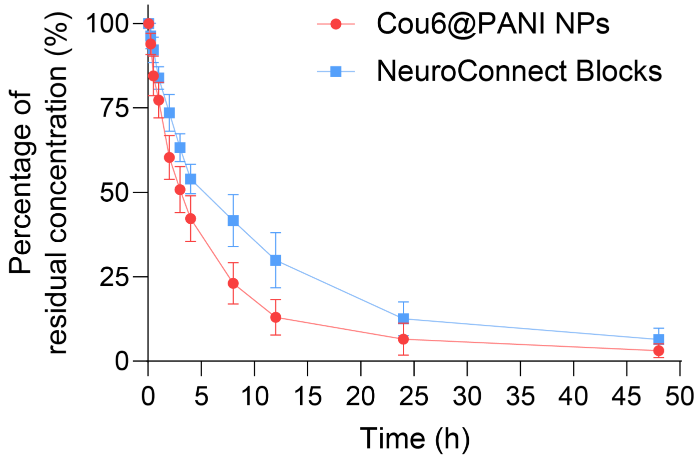

Supplementary Fig. 16 The pharmacokinetic properties of NeuroConnect Blocks scaffolds (n = 3). Data represent mean ± SD of three independent biological replicates.

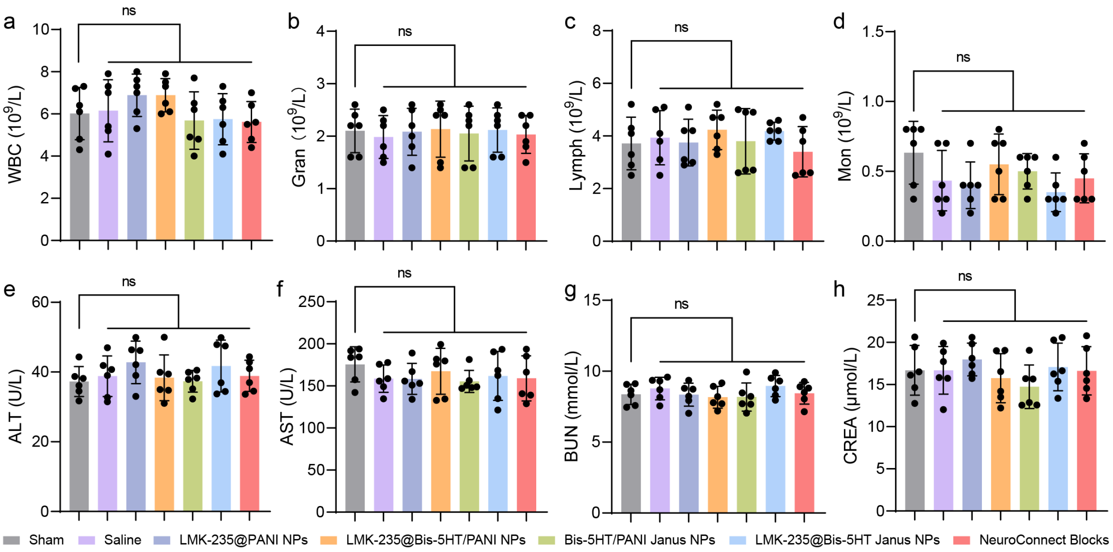

Supplementary Fig. 46 Hematological and biochemical analyses. a-d Evaluations of immune cell levels, including (**a**) WBC counts, (**b**) granulocyte counts, (**c**) lymphocytes counts and (**d**) monocytes counts. **e-h** Assessments of liver and kidney function, including the serum levels of (**e**) ALT, (**f**) AST, (**g**) BUN, and (**h**) CREA. Data represent mean ± SD of six independent biological replicates. Statistical analyses are performed using one-way ANOVA with Tukey's post hoc test.

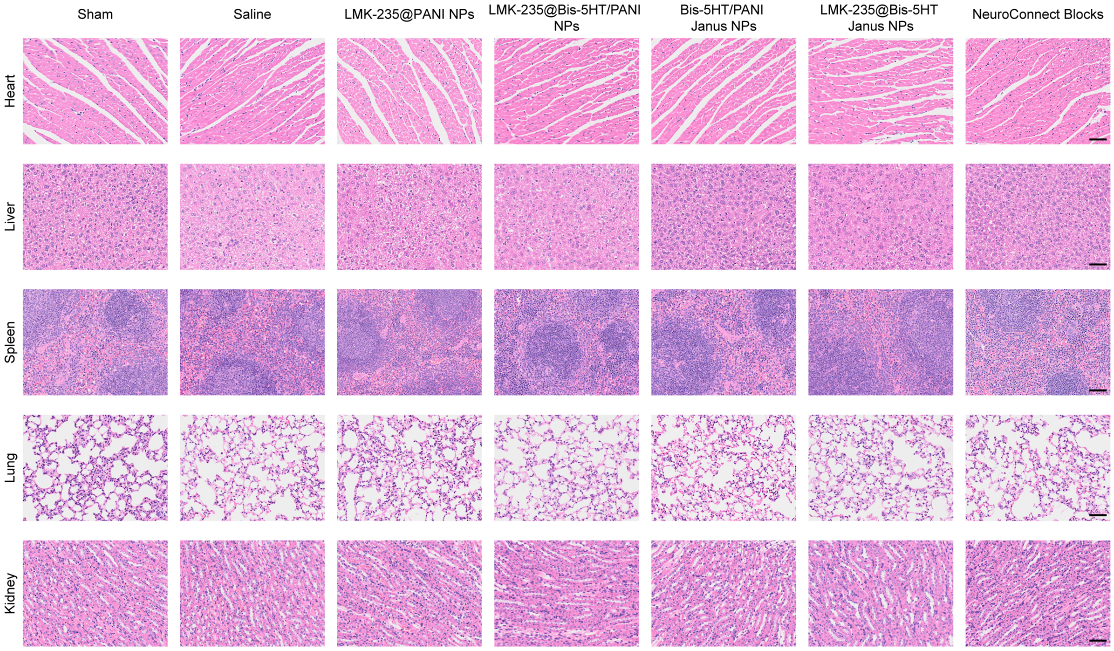

Supplementary Fig. 47 H&E staining to assess the toxicity of various formulations on major organs. Scale, 100 μm. The experiments were repeated six times independently.

2. Is the release rate of LMK-235 *in vivo* comparable to *in vitro* conditions? The authors should consider the microenvironmental differences between *in vitro* and *in vivo* conditions.

Responses: Thank you for your valuable suggestions. Indeed, as you noted, the release rate of LMK-235 *in vitro* is likely to differ from that in the *in vivo* microenvironment. Accordingly, we have incorporated 10% FBS into release media of different pH values to mimic the effect of *in vivo* protein adsorption on drug release, thereby enhancing the relevance of the *in vitro* results to *in vivo* conditions (Supplementary Fig. 9) (***Location of Changes:*** *Line 229-239 in page 10*). Furthermore, to enhance the rigor of this work, we have, following your suggestion and based on relevant authoritative literature, incorporated a discussion in the revised manuscript[22,23](***Location of Changes:*** *Line 1083-1085 in page 49; Line 1086-1087 in page 50*).

The relevant discussion is as follows:

"Due to the complexity of the *in vivo* environment (e.g., pH gradients, abundant proteins, and enzymatic activity), nanoparticle stability and drug release behavior are substantially influenced by factors that are not fully captured in the current *in vitro* PBS

buffer release model. Therefore, the release kinetics of LMK-235 *in vitro* cannot be considered directly equivalent to those *in vivo*. Nevertheless, the existing data provide preliminary evidence for the sustained-release behavior of NeuroConnect Blocks[22,23]. To further elucidate the release characteristics of NeuroConnect Blocks, it will be necessary to incorporate serum proteins into the release media to mimic protein adsorption, as well as to perform animal studies to measure actual plasma drug concentrations and tissue distribution, thereby enabling correlation with the *in vitro* data."

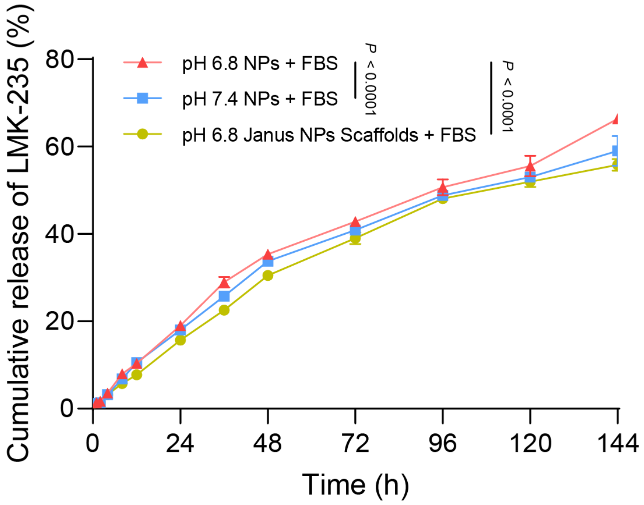

Supplementary Fig. 9 The release profile of LMK-235 from NeuroConnect Blocks before and after scaffolds formation in PBS (pH 6.8 and pH 7.4) at 37 °C with 10% FBS (*n* = 3). Data represent mean ± SD of three independent biological replicates. Statistical analyses are performed using two-way ANOVA with Tukey's post hoc test.

3. Does the extent of NeuroConnect Block assembly to scaffold affect the LMK-235 release rate?

Responses: We appreciate your insightful and kind comments. As you rightly pointed out, the degree of NeuroConnect Block assembly may to some extent influence the release efficiency of LMK-235. Following your suggestion, we examined the release profile of LMK-235 from scaffolds with different degrees of assembly in media of varying pH (Supplementary Fig. 10) (***Location of Changes:*** *Line 231-239 in page 10*).

Specifically, the results showed that scaffolds formed by NeuroConnect Block after

30 minutes of responsive *in vitro* assembly exhibited a slightly slower release rate compared with NeuroConnect Block, whereas further extension of the assembly time had little additional impact on the release profile. Therefore, although the degree of NeuroConnect Block assembly does influence its release behavior, the rapid and sufficient assembly of NeuroConnect Block ensures relatively stable release characteristics (Supplementary Fig. 10).

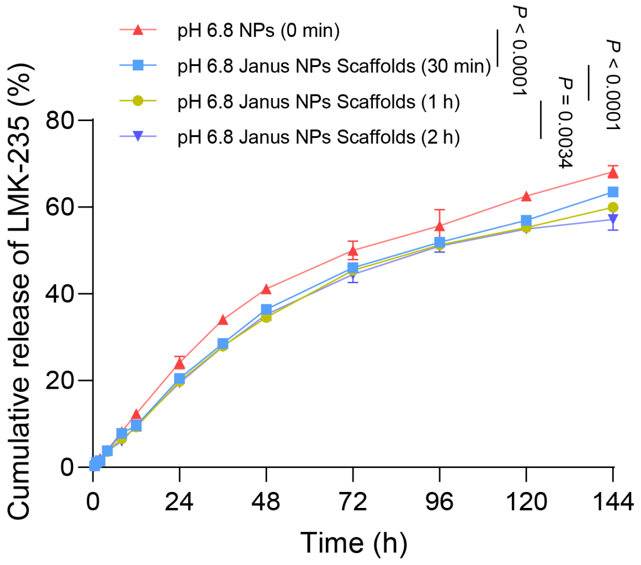

Supplementary Fig. 10 The release profile of LMK-235 from scaffolds with different degrees of assembly in PBS (pH 6.8 and pH 7.4) at 37 °C (n = 3). Data represent mean ± SD of three independent biological replicates. Statistical analyses are performed using two-way ANOVA with Tukey's post hoc test.

4. Figure 7 g. The authors stated, "Confocal imaging revealed that NeuroConnect Blocks scaffolds maintained their structural integrity within the injury site and displayed extensive infiltration by regenerating neuronal axons (Fig. 7f-i)." To show the infiltration of cells and confirm the statement the 3D confocal image is required.

Responses: We sincerely appreciate your professional comments. Following your suggestion, we have incorporated 3D confocal images in the manuscript to demonstrate the infiltration of regenerating neuronal axons (Supplementary Fig. 29) (***Location of Changes:*** *Line 526-528 in page 23*).

The details are as follows:

Conventionally, tissue sections used for immunofluorescence staining are no thicker than 50 μm, while cortical neuronal axons in mice are typically long and extend substantially along the Z-axis. This makes it highly challenging to characterize the 3D spatial relationship between axons and biomaterials using routine tissue sections. Nevertheless, we greatly appreciated your recommendation. Accordingly, we intravenously injected Rhodamine B-labeled NeuroConnect Block and, after 4 weeks, harvested the brains and prepared 300 μm coronal sections. Cortical neurons within the scaffolds were filled with the fluorescent dye Biocytin via patch-clamp recording to visualize their axons, and fluorescence images were acquired using laser confocal microscopy. Subsequently, 3D reconstruction techniques were applied to reveal the spatial relationship between axons and the scaffolds (Supplementary Fig. 29 and Supplementary Video 1). It is important to note that the 3D reconstruction images are computational renderings derived from fluorescence data, primarily reflecting spatial organization rather than representing actual size or dimensions.

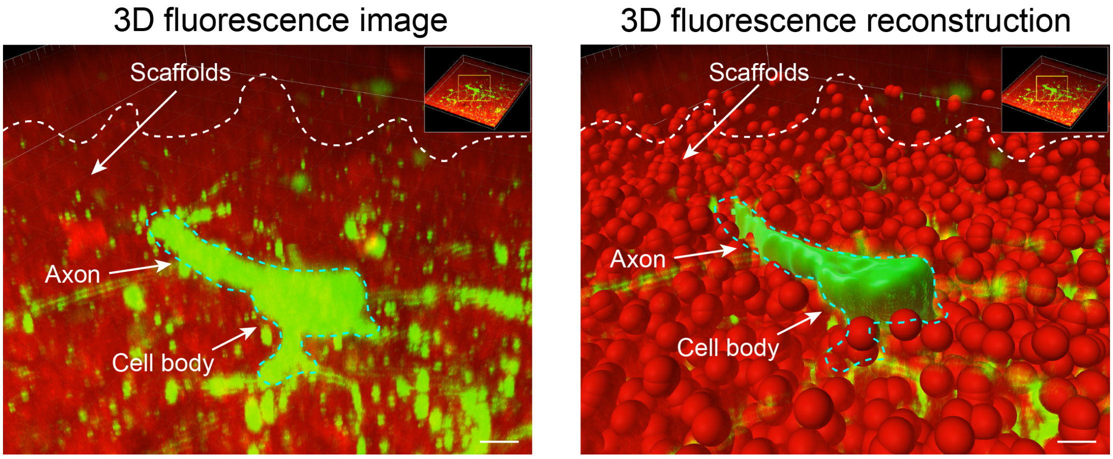

Supplementary Fig. 29 3D fluorescence image and 3D reconstructed image showing the infiltration of axons (Biocytin-labeled) into the NeuroConnect Block scaffold (Rhodamine B-labeled) at the brain injury site. The 3D reconstructed image was rendered using Imaris software. The fluorescence signals of the scaffolds were reconstructed based on a spherical model, which does not represent its actual shape or size. Scale, 10 μm.

5. Figure 8: A) It seems different location of brain is being analysed across different groups. Where in the brain is this stained section from in relation to the site of injection?

B) The intensity of the images taken looks different. We expect to see different intensity in Iba-1 and GFAP between different groups however, the DAPI should be the same in images with same intensity and microscopic adjustment. So, please make sure all the images are taken with the same adjustment as the results and quantifications are based on intensity. (for example, in NeuroConnect Block group in 28 days). C) Higher magnification images are required to better visualise the GFAP and Iba-1 cells.

Responses: We appreciate your professional and valuable suggestions.

A) Our selection of analysis sites for each group was based on two considerations: ***i.*** The analysis regions were chosen near the M2 area of the injured hemisphere, as this is the region most severely affected by secondary injury following TBI and also the primary site of NeuroConnect Block transvascular accumulation after injection. ***ii.*** We preferentially selected areas with relatively high levels of inflammation in each group, in order to more rigorously demonstrate the therapeutic efficacy of NeuroConnect Block. Therefore, although the specific analysis sites differed among groups, our selection was scientific and justified rather than subjective.

B) We ensured that the fluorescence signals of GFAP, Iba-1, and DAPI in each group were processed using a uniform threshold adjustment. The apparent differences in DAPI fluorescence intensity across images arise from the use of an anti-fade mounting medium containing DAPI (#P0131, Beyotime) for nuclear staining. During the mounting process, variations in the amount of mounting medium overflow, or repeated addition of medium due to bubble formation and subsequent remounting, may have introduced slight differences in DAPI staining among tissue sections. However, as DAPI is primarily used for tissue and cellular localization rather than for semi-quantitative fluorescence analysis of markers, these differences do not affect the validity of the conclusions—an understanding that is widely recognized and accepted[24,25]. In contrast, GFAP and Iba-1 were stained using antibody-based immunofluorescence, a method that does not introduce intergroup experimental bias; therefore, under identical threshold adjustments, their semi-quantitative fluorescence intensity results are entirely reliable.

C) In fact, our original high-magnification images already allowed clear

observation of astrocyte and microglial morphology. However, due to the 30 MB file size limit of the Nature Communications submission system, we compressed the original files into PDF format for upload, which resulted in reduced image clarity. Following your suggestion, we have reinserted the high-resolution original images into the manuscript and included enlarged high-resolution originals in the supplementary information, ensuring that the morphology of astrocytes and microglia can be clearly visualized (Fig. 8, Supplementary Figs. 30, 31, 36, 37).

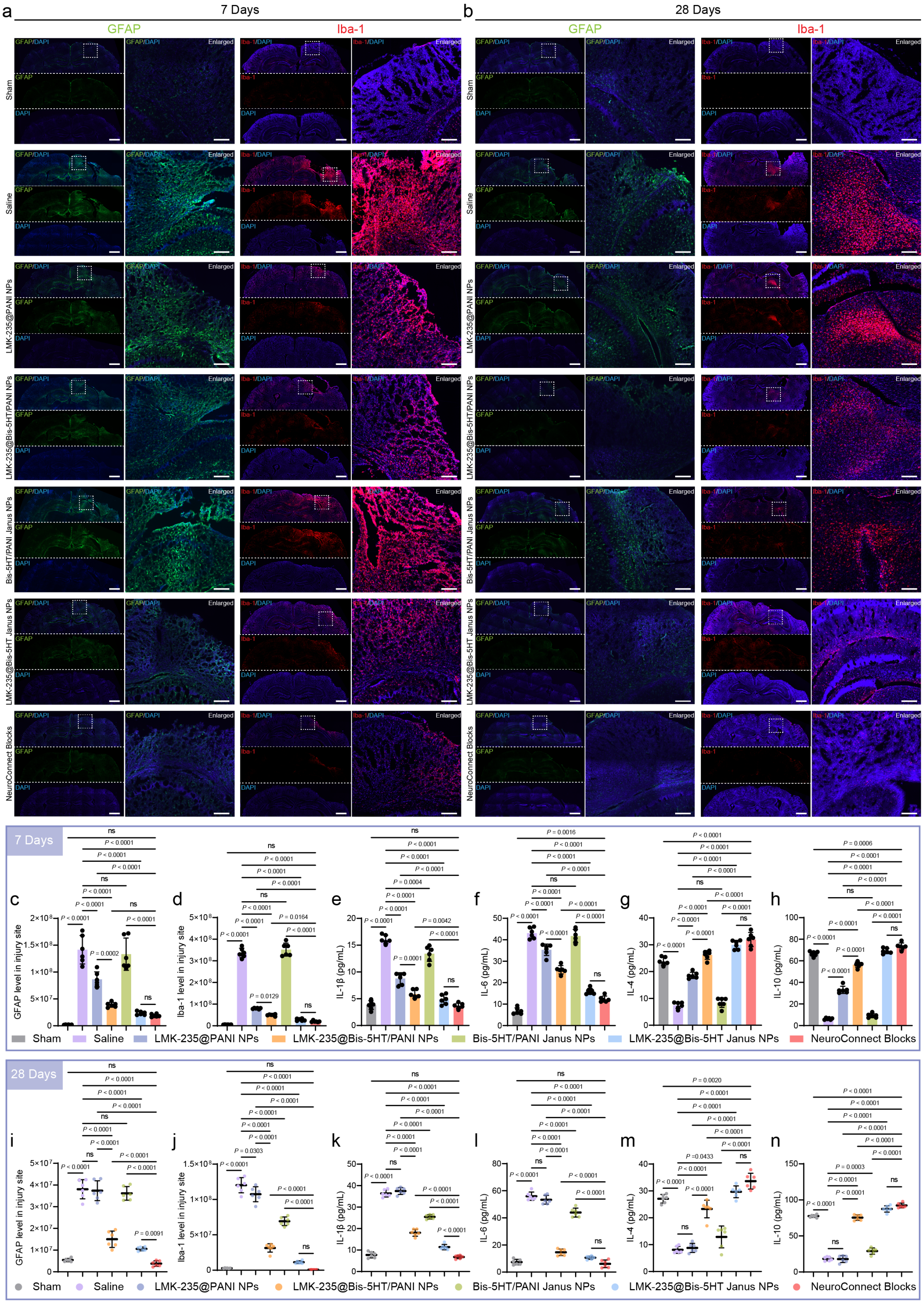

Fig. 8 | NeuroConnect Blocks consistently regulate the injured microenvironment to ensure the smooth reconstruction of neural circuits. a, b Representative images of glial scars (GFAP) and microglia (Iba-1) in injured sites of various groups at 7 (**a**) and 28 days (**b**) post-injury. Scale, 1 mm (scan); 200 μm (enlarged image). **c, d** Quantitative analysis showing the fluorescence intensity

of (**c**) GFAP and (**d**) Iba-1 corresponding to (**a**) ($n = 6$). **e-h** Detection of pro-inflammatory cytokines in the homogenates of injured brains from various groups at 7 days post-injury, including (**e**) IL-1β, (**f**) IL-6, (**g**) IL-4, and (**h**) IL-10 ($n = 6$). **i, j** Quantitative analysis showing the fluorescence intensity of (**i**) GFAP and (**j**) Iba-1 corresponding to (**b**) ($n = 6$). **k-n** Detection of pro-inflammatory cytokines in the homogenates of the injured brain from various groups at 28 days post-injury, including (**k**) IL-1β, (**l**) IL-6, (**m**) IL-4, and (**n**) IL-10 ($n = 6$). Data represent mean ± SD of six independent biological replicates. Statistical analyses are performed using one-way ANOVA with Tukey's post hoc test.

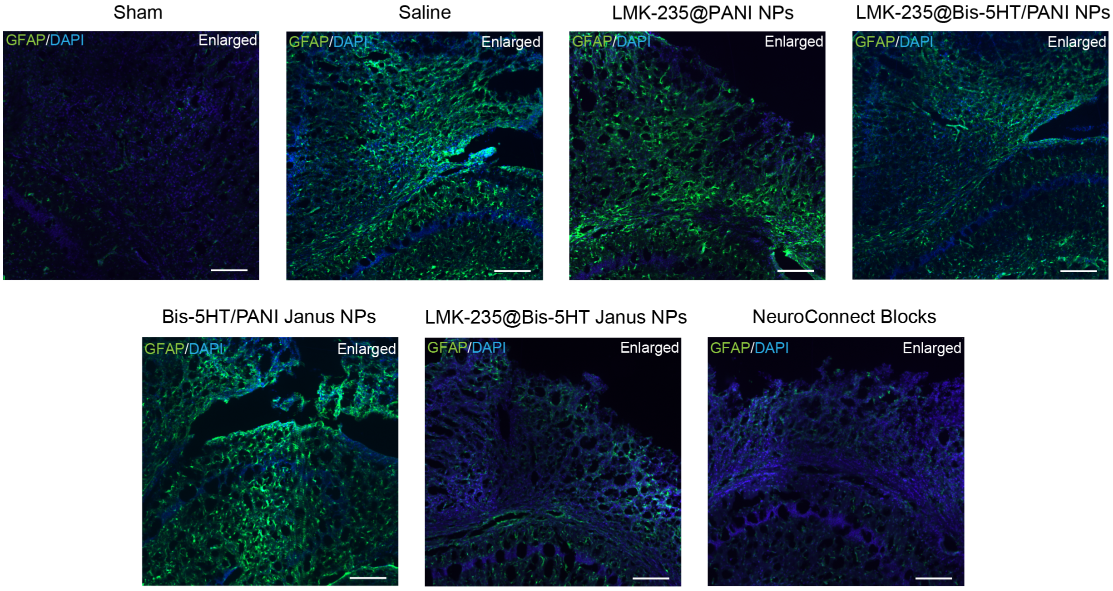

Supplementary Fig. 30 Representative images of glial scars (GFAP) in injured sites of various groups at 7 days post-injury. Scale, 200 μm.

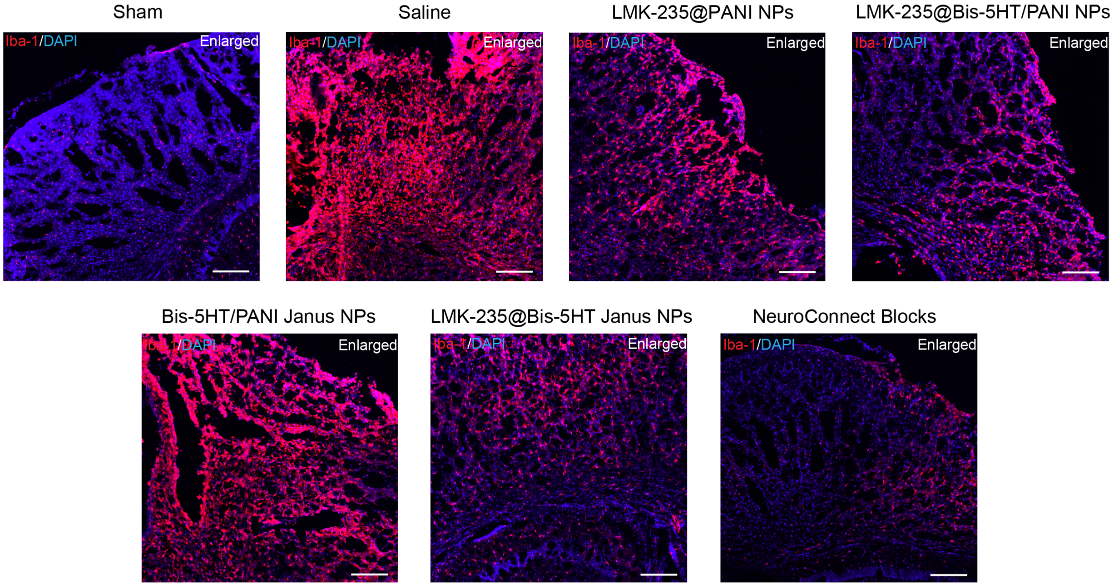

Supplementary Fig. 31 Representative images of microglia (Iba-1) in injured sites of various groups at 7 days post-injury. Scale, 200 μm.

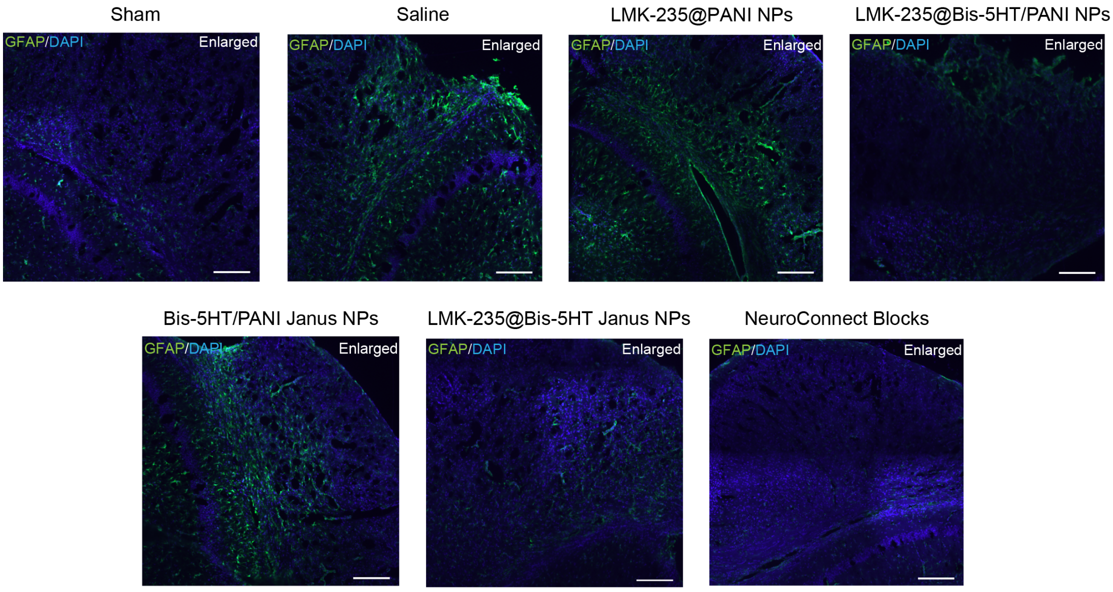

Supplementary Fig. 36 Representative images of glial scars (GFAP) in injured sites of various groups at 28 days post-injury. Scale, 200 μm.

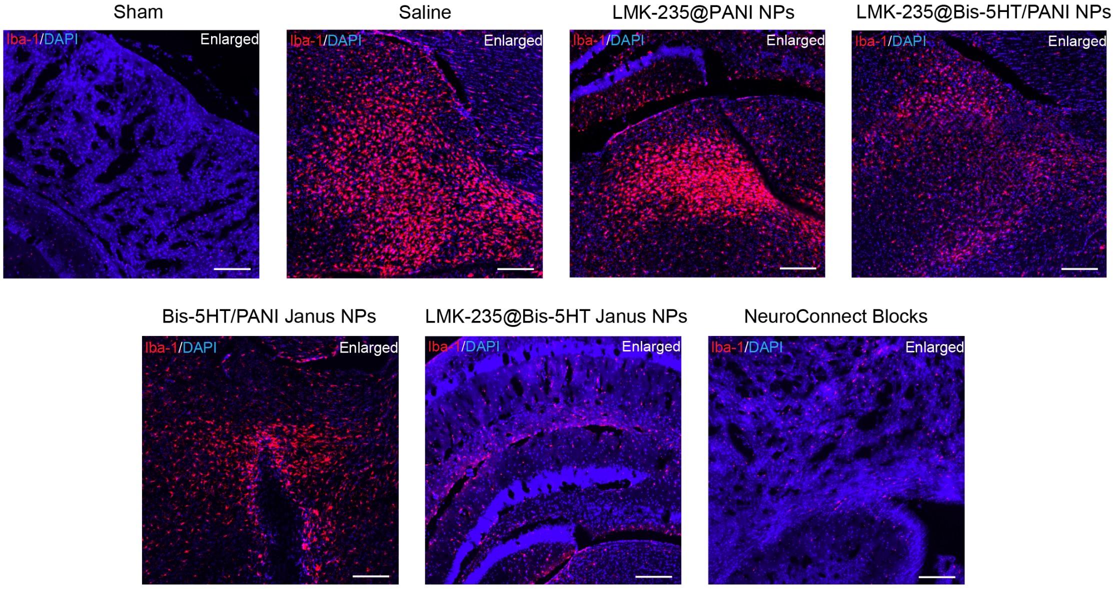

Supplementary Fig. 37 Representative images of microglia (Iba-1) in injured sites of various groups at 28 days post-injury. Scale, 200 μm.

6. What is the degradation profile of the NeuroConnect Blocks scaffold and by what mechanism does it degrade in the body (i.e. enzymatic)?

Responses: We appreciate your rigorous questions. <u>We have already included an</u>

evaluation of the degradation profile of NeuroConnect Blocks scaffolds *in vitro*, showing that they degrade slowly under simulated physiological conditions, with approximately 98.15% degradation observed within 120 days (Supplementary Fig. 15). In addition, following your suggestion, we have added a discussion in the manuscript regarding the potential mechanisms underlying the *in vivo* degradation of NeuroConnect Blocks scaffolds (***Location of Changes:*** *Line 1031-1048 in page 48*).

The relevant discussion is as follows:

"The *in vivo* degradation mechanisms of PLGA and bis-5HT within the NeuroConnect Blocks scaffolds primarily involve chemical hydrolysis, accompanied by enzymatic reactions mediated by esterases that accelerate ester bond cleavage. In addition, immune cells such as macrophages can phagocytose the degradation fragments and further process them through intracellular enzymatic systems[26,27]."

"Polyaniline (PANI) itself exhibits chemical stability under physiological conditions, owing to its conjugated backbone and the absence of hydrolyzable bonds, rendering it poorly degradable and absorbable *in vivo*. However, when PANI is incorporated with biodegradable materials—for instance, in this study, through the in situ polymerization of aniline monomers on the nanoparticle surface to form a core–shell biodegradable polymer—PANI can be eliminated from the body via "bioerosion". Specifically, as the biodegradable matrix undergoes gradual hydrolysis, the nanostructure of PANI loses its supporting framework and breaks down into smaller fragments. Although PANI cannot undergo molecular-level degradation, these nanoscale fragments can be cleared by phagocytic cells (e.g., macrophages) and eventually excreted through renal or biliary pathways. Consequently, despite its intrinsic non-degradability, PANI demonstrates favorable biocompatibility and safety, supporting its broad application in biosensing, neural engineering, and tissue engineering[4,5]."

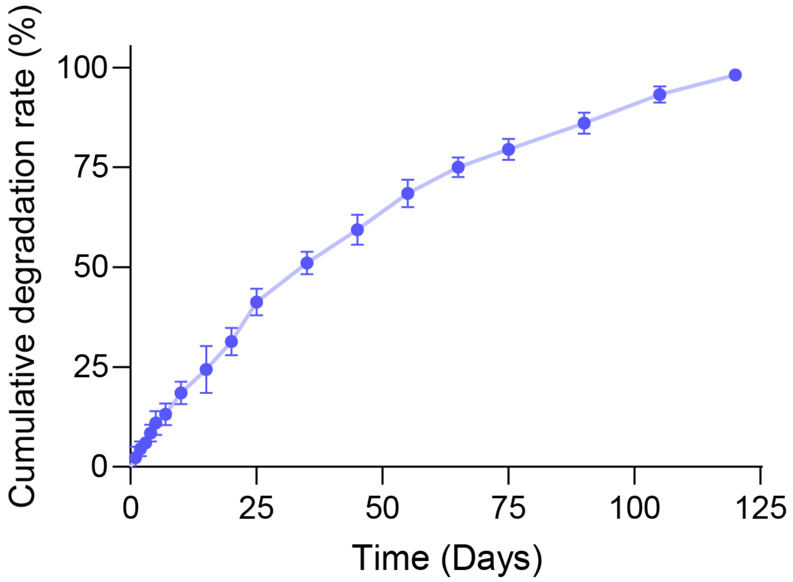

Supplementary Fig. 15 The degradation curve of NeuroConnect Blocks scaffolds (*n* = 3). Data represent mean ± SD of three independent replicates.

7. The image quality and resolution in Figures are suboptimal specially for *in vivo*, making it difficult to observe marker staining.

Responses: We appreciate your expert feedback regarding the image quality and resolution in Figures. Previously, due to the 30 MB file size limit of the Nature Communications submission system, we compressed the original files into PDFs, which resulted in substantial compression of the embedded images. We have now regenerated the PDF using Adobe Acrobat, ensuring that file size reduction does not compromise the embedded images, thereby preserving the original quality and resolution of all figures in the manuscript.

Reviewer #3 (Remarks to the Author):

I co-reviewed this manuscript with one of the reviewers who provided the listed reports. This is part of the Nature Communications initiative to facilitate training in peer review and to provide appropriate recognition for Early Career Researchers who co-review manuscripts.

Responses: We greatly appreciate the reviewer for the professional comments and advice. We have addressed all of your comments and made the corresponding revisions.

Reviewer #4 (Remarks to the Author):

Tong et al present in their manuscript a new approach using targeting nanoparticles for drug delivery to restore neural functions after traumatic brain injury (TBI). The authors support the studies of this new drug delivery system with substantial experiments both *in vitro* and *in vivo*. The main contribution to the field of this manuscript is the elegant use of elevated Myeloperoxidase levels at injury sites to self-assemble the nanoparticles providing enhanced local retention and delivery of the encapsulated drug. This could potentially be of interest to a general public, but many studies have shown the accumulation of nanoscale drug carriers in the CNS after TBI or the central effects of their cargoes. For example, Li et al Sci Adv 2021 BBB pathophysiology–independent delivery of siRNA in traumatic brain injury or the recent review by McLoughlin et al 2024 and ref in there Overcoming the Blood–Brain Barrier: Multifunctional Nanomaterial-Based Strategies for Targeted Drug Delivery in Neurological Disorders - McLoughlin - 2024 - Small Science - Wiley Online Library.

Responses: We greatly appreciate your expert comments and insightful feedback. While many studies have shown the accumulation of nanoscale drug carriers in the CNS after TBI or the central effects of their cargoes, our research introduces several innovative elements, as highlighted by three key aspects:

1. We present the first demonstration of neural guidance scaffold construction at CNS lesion sites through intravenous administration of 'Janus'-functionalized conductive nano-drug delivery systems. These novel structures serve the dual purpose of providing neural support and functioning as drug reservoirs.

2. Our study addresses a fundamental limitation in current CNS axonal regeneration therapies, which have shown limited clinical success. Specifically, we target the intrinsic deficiency in histone deacetylase 5 (HDAC5) nuclear export in central neurons—a key factor limiting their regenerative capacity. Through the application of

depot-formulated HDAC5 inhibitors, we achieve comprehensive activation of the axonal regeneration program in central neurons.

3. We provide compelling evidence that the conductive coating on our scaffolds facilitates neural electrical signal interactions, thereby guiding synaptic reconnection. This demonstrates the crucial role of electrical signal transmission in enhancing repair outcomes.

The manuscript is very extensive and the authors should be more selective and focus on the most relevant results.

Responses: We sincerely appreciate your kind advice. We have reorganized the data according to your suggestions, making the manuscript more selective and focused on the relevant results while preserving its overall integrity. Specifically, we have included the *in vitro* results demonstrating the ability of NeuroConnect Blocks scaffolds to resist rapid cellular uptake and clearance in the Supplementary Information, in order to enhance the focus of the experimental findings presented in the main manuscript (Supplementary Fig. 14).

In addition, the synthesis described of the nanocarriers are complex and included multiple intermediates and there is a lack of details and inadequate characterization of intermediates and the final nanocarrier (more details below) which makes it difficult to validate the *in vitro* and *in vivo* results.

Responses: We appreciate your constructive suggestions. Accordingly, we have supplemented the manuscript with detailed information on the intermediates and the final nanocarrier (Fig 2b, c; Supplementary Tab. 3) (***Location of Changes:*** *Line 1203-1208 in page 54*).

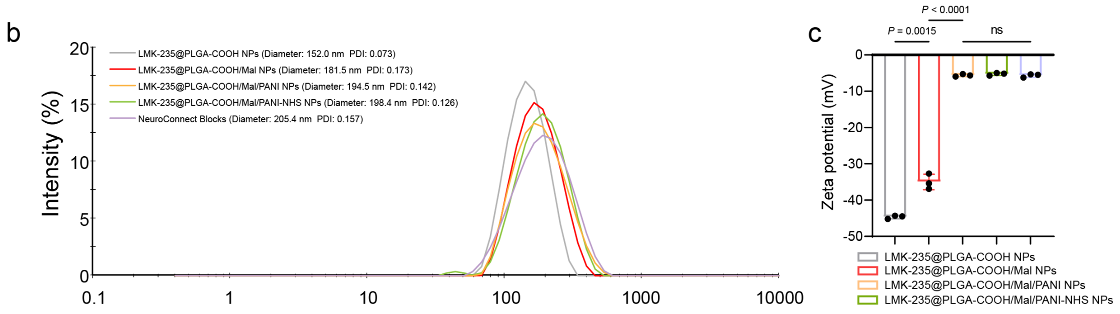

Fig. 2 | Preparation and characterization of NeuroConnect Blocks. b Size distribution of NeuroConnect Blocks detected by DLS. **c** Zeta potential of NeuroConnect Blocks (n = 3). Data represent mean ± SD of three independent biological replicates. Statistical analyses are performed using one-way ANOVA with Tukey's post hoc test.

Supplementary Tab. 3 The detailed information on the intermediates and the final nanocarrier.

Nanoparticles	Size (nm)	PDI	*Zeta* potential (mV)	EE (%)	LE (%)	Yield (%)
**LMK-235@PLGA-COOH NPs**	152.0	0.073	-44.7	78.84	8.26	81.58
**LMK-235@PLGA-COOH/Mal NPs**	181.5	0.173	-35	77.46	8.17	81.03
**LMK-235@PLGA-COOH/Mal/PANI NPs**	194.5	0.142	-5.6	73.75	7.05	34.49
**LMK-235@PLGA-COOH/Mal/PANI-NHS NPs**	198.4	0.126	-5.3	71.39	5.93	38.43
**NeuroConnect Blocks**	205.4	0.157	-5.7	70.56	5.88	38.06

The authors claim that the encapsulated drug LMK-235 is a selective HDAC 5 inhibitor and conclusions at several places in the manuscript are drawn based on this fact. However, LMK-235 is equipotent on HDAC4. Other isoforms of HDACs although 10-100 fold lower potency might also contribute considering the local application and nanomolar potency. For inhibitor profile of LMK-235 see https://www.medchemexpress.com/LMK-235.html?srsltid=AfmBOopA1cuwzkN6saYlQ4eJhO4ydSzxB7IkxyzIcOhw6r2cisRm2wmI

Either the authors need to demonstrate that it is the HDAC5 inhibitory effect that contribute to the regeneration or rephrase throughout the manuscript.

Responses: We sincerely appreciate your highly professional and important comments. You correctly pointed out that LMK-235 exhibits comparable inhibitory activity against HDAC4 and HDAC5, and that other HDAC isoforms may carry potential off-target risks at locally elevated concentrations. We fully agree with your view. In response to your comment, we have taken the following steps:

1. Comprehensive revision of the manuscript:

We have carefully revised the relevant statements throughout the manuscript, avoiding absolute terms such as "selective HDAC5 inhibitor". Instead, we now employ more precise and rigorous descriptions (***Location of Changes:*** *Line 41 in page 2; Line 105-106 in page 4; Line 359 in page 15; Line 725 in page 34; Line 990 in page 46*). In addition, we have added a dedicated paragraph in the Discussion to specifically address the issue of LMK-235 selectivity, transparently analyzing the correlation between its inhibitory activity on HDAC5 and the observed outcomes in neural regeneration (***Location of Changes:*** *Line 1022-1027 in page 47; Line 1028-1030 in page 48*).

2. Inference regarding the role of HDAC5:

Although LMK-235 demonstrates similar inhibitory activity against HDAC4 and HDAC5, we infer that HDAC5 inhibition plays a more critical role in our model based on the following considerations:

A) *Evidence from the literature:* Multiple independent studies have directly linked the loss or inhibition of HDAC5 with enhanced axonal regeneration and neuronal survival, supporting HDAC5 as a more specific regulatory target for neural regeneration[28-30].

B) *Specificity within our model*: Our experimental data show that LMK-235 treatment induces distinct phenotypes—such as enhanced axonal regeneration and upregulation of HDAC5-associated transcription factors including *Jun*, *Fos*, and *Klf6*. These phenotypes closely align with those reported for HDAC5 loss-of-function, rather than HDAC4[28].

C) *Concentration considerations*: The dosing regimen we employed was carefully

designed to achieve effective drug concentrations in the target tissue within the low nanomolar range. At this concentration, the inhibitory effects of LMK-235 on other isoforms are expected to be negligible. Therefore, the main off-target concern lies in the inability to clearly distinguish between HDAC4 and HDAC5.

On this basis, we have incorporated additional discussion in the manuscript to strengthen the rigor of the study (***Location of Changes:*** *Line 1022-1027 in page 47; Line 1028-1030 in page 48*).

Once again, we are deeply grateful for this constructive feedback, which has greatly helped us improve the scientific robustness of our work.

The nanocarrier is reported to be approximately 200 nm. Previous studies have found that nanoparticles around 30 nm are optimal for TBI application and 80 nm remains in the blood vessel. Could the authors comment on this. See Size-Selective Transfer of Lipid Nanoparticle-Based Drug Carriers Across the Blood Brain Barrier Via Vascular Occlusions Following Traumatic Brain Injury

Responses: Thank you for your valuable comments. The findings reported in the reference you provided were obtained using a moderate TBI model (impact velocity: 6 m/s, impact depth: 0.5 mm, contact time: 150 ms), whereas our study employed a severe TBI model (impact velocity: 3.5 m/s, impact depth: 1 mm, contact time: 400 ms). The degree of BBB permeability following TBI is largely determined by the extent of both primary and secondary injury[31]. Therefore, given the more severe injury model used in our study, it is entirely plausible that NeuroConnect Blocks particles of approximately 200 nm in size were able to penetrate the BBB and accumulate within the brain. Moreover, dedicated studies have demonstrated that PLGA nanoparticles ranging from 100 nm to 800 nm are capable of accumulating in the brain after TBI[32].

Specific comments

The method section for the synthesis and characterizations of the intermediates and nanocarrier is not complete and lacks important information that enables reproducibility. The details for synthesis of control nanoparticles lacking critical features are inadequate

Responses: We appreciate your constructive suggestions. We have added the details for synthesis of control nanoparticles lacking critical features (***Location of Changes:*** *Line 1203-1208 in page 54*).

No/limited references to methods used in the synthesis of the nanocarrier

Responses: Thank you very much for the kind advice. Accordingly, we have added the relevant references to methods used in the synthesis of the nanocarrier (***Location of Changes:*** *Line 1176 in page 53; Line 1180-1185 in page 53; Line 1198 in page 53*).

There is no reported yield for the first, PANI step and the final nanocarrier despite isolating the particles.

Responses: Thank you for the suggestions. We have supplemented the manuscript with the yields for each step of NeuroConnect Blocks preparation (Supplementary Tab. 3).

Supplementary Tab. 3 The detailed information on the intermediates and the final nanocarrier.

**Nanoparticles**	**Size (nm)**	**PDI**	***Zeta* potential (mV)**	**EE (%)**	**LE (%)**	**Yield (%)**
**LMK-235@PLGA-COOH NPs**	152.0	0.073	-44.7	78.84	8.26	81.58
**LMK-235@PLGA-COOH/Mal NPs**	181.5	0.173	-35	77.46	8.17	81.03
**LMK-235@PLGA-COOH/Mal/PANI NPs**	194.5	0.142	-5.6	73.75	7.05	34.49
**LMK-235@PLGA-COOH/Mal/PANI-NHS NPs**	198.4	0.126	-5.3	71.39	5.93	38.43
**NeuroConnect Blocks**	205.4	0.157	-5.7	70.56	5.88	38.06

What is hydrogen peroxide enzyme in the PANI step?

Responses: We appreciate your feedback. In our study, hydrogen peroxide enzyme was employed to catalyze the in situ polymerization of aniline. Under the presence of H_2O_2, the heme cofactor (Fe^{3+}) of catalase is first oxidized by H_2O_2 to generate a highly reactive intermediate. This intermediate can abstract an electron from an aniline molecule, forming an aniline radical cation, which in turn efficiently promotes the in

situ polymerization of aniline to yield PANI[33]. Accordingly, we have incorporated the relevant explanation and references into the manuscript (***Location of Changes:*** *Line 1180-1185 in page 53*).

The authors report conductivity of the nanoparticles in figure 2D but does not describe the procedure neither do they show that the conductivity of the PANI layer is crucial for the system considering that the title include "electrically guide..".

Responses: Thank you very much for the kind suggestion. We have incorporated the procedural details of the conductivity testing (***Location of Changes:*** *Line 1223-1229 in page 54*).

Furthermore, in our design, the conductivity of the PANI layer is essential to the system. Mechanistically, the scaffolds formed by NeuroConnect Blocks promote neural circuit repair primarily through two steps: (1) attracting central neurons to grow directionally along the scaffold via the concentration gradient of LMK-235, and (2) facilitating synaptic reconnection of the recruited central neurons within the scaffold channels through neural signal transmission mediated by the conductive PANI layer. Experimentally, we established control groups in both *in vitro* or *in vivo* neuronal synaptic reconnection assays and behavioral tests to demonstrate the critical role of the PANI conductive layer in the system (Fig. 5, 7, 9, 11; Supplementary Figs. 22, 23, 27-29, 43-45). In addition, by overexpressing Kir2.1 in neurons to suppress their electrical activity, we directly confirmed that the scaffold's synaptic reconstruction effect is partly dependent on electrical signal conduction mediated by the PANI conductive layer (Fig. 10i-o; Supplementary Figs. 57, 58).

And the authors claim that the nanocarrier contain a conductive polyaniline layer proven by a change in zeta potential and the analysis of conductivity described in figure 2D. The reported conductivity (50-60 mS/cm) is only slightly above CSF (15-20) and blood (5-10) therefore it is hard to understand how this limited conductivity contribute.

Responses: We greatly appreciate your professional comments. The point you raised is indeed critical. Admittedly, in terms of absolute values, the nanocarriers increase

conductivity only from 5–20 mS/cm (in body fluids) to 50–60 mS/cm, which may appear to be a modest improvement. However, the core function of the NeuroConnect Blocks design in this study is not merely to elevate bulk conductivity, but rather to direct neural regeneration through the construction of an anisotropic, macroscale conductive architecture. To enhance the reader's understanding, we have incorporated the relevant elaboration into the manuscript (***Location of Changes:*** *Line 178-180 in page 7*).

A detailed explanation is provided as follow:

1. Providing oriented pathways: The interconnected conductive network assembled from nanoparticles establishes directional routes for neural signal transmission—something that isotropic cerebrospinal fluid (CSF) alone cannot achieve.

2. Optimizing interfacial transmission: Compared with insulating scaffolds, the conductive PANI layer markedly reduces the impedance at the scaffold–neural tissue interface, thereby greatly improving the efficiency of bioelectrical signal transfer and minimizing signal loss.

3. Creating an electroactive microenvironment: The conductive scaffold network, together with the CSF within the pores, forms a "conductor–electrolyte" interface. This allows current to be effectively transmitted from the scaffold to the CSF and subsequently to the cells, and vice versa, thereby enhancing neural guidance.

Moreover, the conductivity of polyaniline (50–60 mS/cm) is far higher than that of insulating polymers (<0.1 mS/cm), yet avoids the problems associated with metals (>10^6 mS/cm), such as electrical "short-circuiting," excessive localized heating, or harmful electrolytic reactions. This balance explains why PANI is widely used in neural tissue engineering research[4,5].

In summary, the value of the PANI layer in this study lies in endowing the scaffold with essential electrophysiological functionality, rather than simply maximizing its absolute conductivity.

But one important aspect is that the negative zeta potential is reduced approaching 0 and it is well known that this will increase the likelihood of aggregation and most likely increase tissue adherence. Both could potentially be important for retention at the site

of injury and neuron adherence.

Responses: Thank you very much for the professional comments and kind advice. We fully agree that, under typical circumstances, a near-neutral *Zeta* potential markedly reduces colloidal electrostatic stability, thereby leading to aggregation. However, in the nanoparticle design of this study, we circumvented this issue through a steric stabilization mechanism:

As described in the manuscript, the nanoparticle surfaces were densely functionalized with long, hydrophilic PEG_{2000} and PEG_{5000} chains. These PEG chains extend fully in aqueous environments to form a hydrated layer, which generates substantial steric repulsion between nanoparticles and thereby effectively prevents aggregation. This strategy is widely employed in nanomedicine to ensure colloidal stability under physiological conditions[34-39]. Moreover, the uniform particle size distribution shown in Fig. 2b directly demonstrates that the nanoparticles remained well-dispersed after preparation, without evidence of aggregation (Fig. 2b).

b

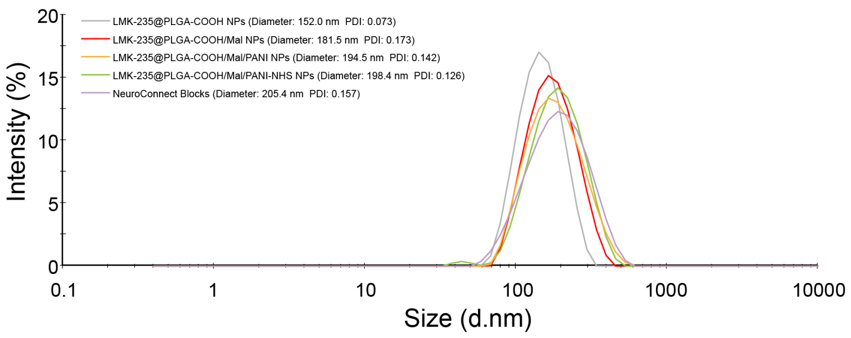

Fig. 2 | Preparation and characterization of NeuroConnect Blocks. b Size distribution of NeuroConnect Blocks detected by DLS.

Bis-5HT-Glu-NH_2 is not properly characterized only with MS (supp info). LC/1H/13C NMR should be added to verify purity.

Responses: Thank you for your valuable feedback and suggestion. We have added the HPLC and ^{1}H NMR purity assessment results to the manuscript, which demonstrate that the purity of Bis-5HT-Glu-NH_2 exceeds 96.26% (Supplementary Figs. 4, 5).

<Chromatogram>

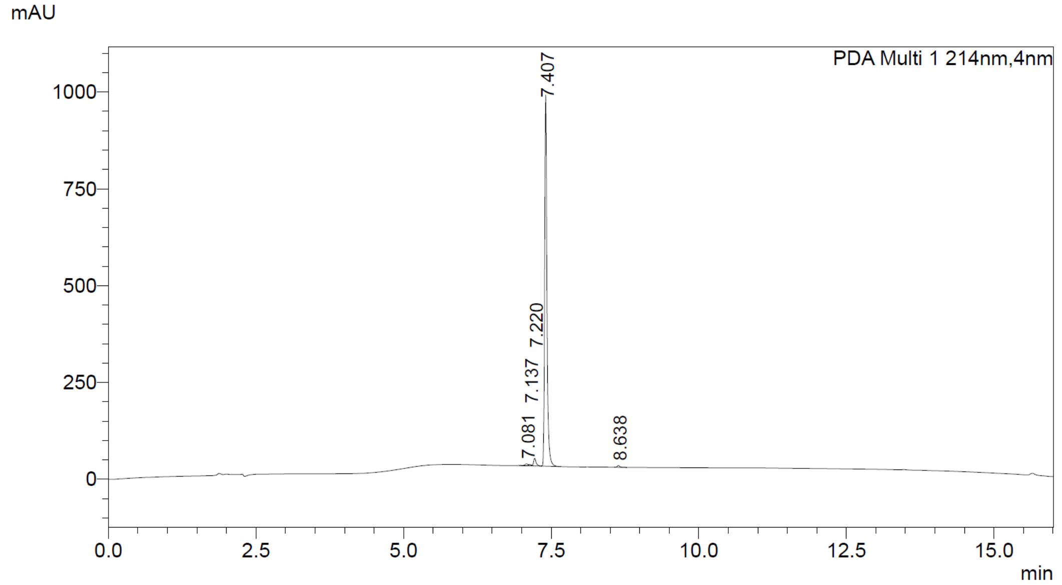

<Peak Table>

PDA Ch1 214nm

Peak#	Ret. Time	USP Width	Height	Area	S/N	Area%
1	7.081	0.128	4791	17428	5.46	0.658
2	7.137	0.192	3520	10224	4.01	0.386
3	7.220	0.087	19699	58662	22.46	2.213
4	7.407	0.076	958499	2551130	1092.73	96.257
5	8.638	0.091	4103	12876	4.68	0.486
Total				2650320		100.000

Supplementary Fig. 4 HPLC purity analysis of bis-5HT-Glu-NH_2.

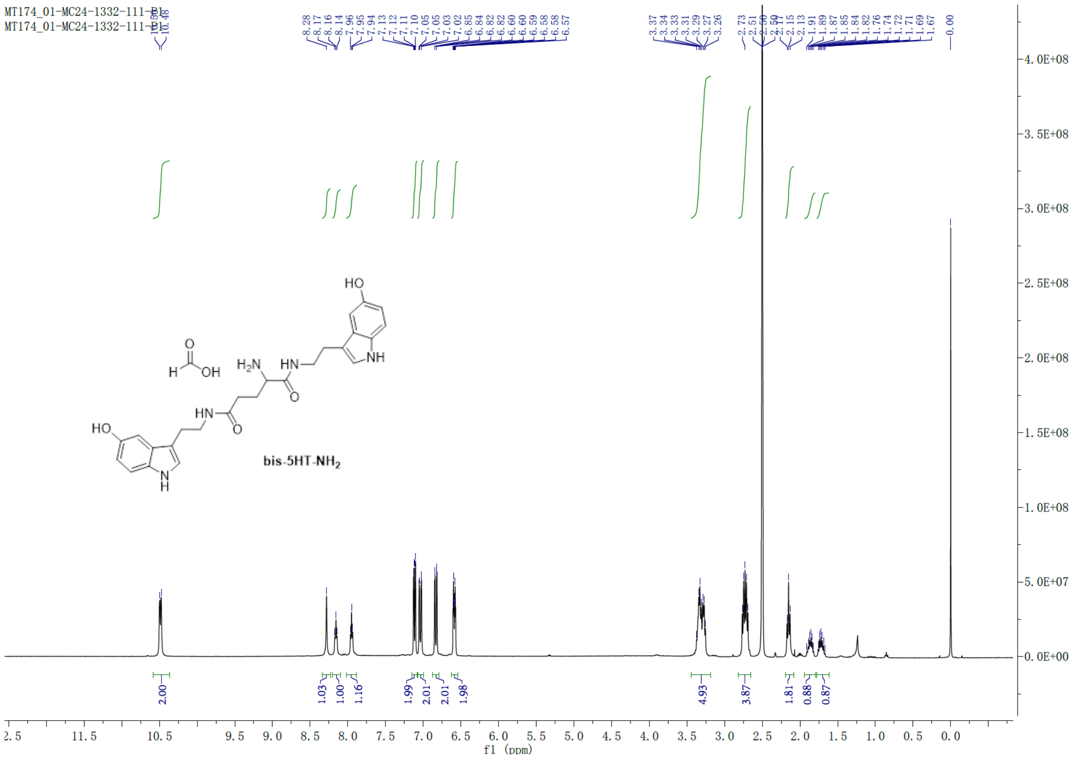

Supplementary Fig. 5 ^{1}H NMR purity analysis of bis-5HT-Glu-NH_2.

The authors covalently adds gold nanoparticles using NHS chemistry. TEM images shows asymmetric distribution on selected particles, but a composition analysis based on elemental analysis with ICP or surface plasmon analysis to quantify % gold is necessary to confirm partial distribution for all particles.

Responses: We sincerely appreciate your professional comments. In line with your suggestion, we have performed quantitative determination of the gold content using ICP-OES and ICP-MS elemental analysis to verify the partial distribution of all particles. Specifically, the gold content was 0.0004% for the control nanoparticles without surface-conjugated gold nanoparticles, 0.2094% for conventional nanoparticles with symmetrically conjugated surface gold nanoparticles, and 0.1283% for NeuroConnect Blocks with asymmetrically conjugated surface gold nanoparticles (Supplementary Fig. 7).

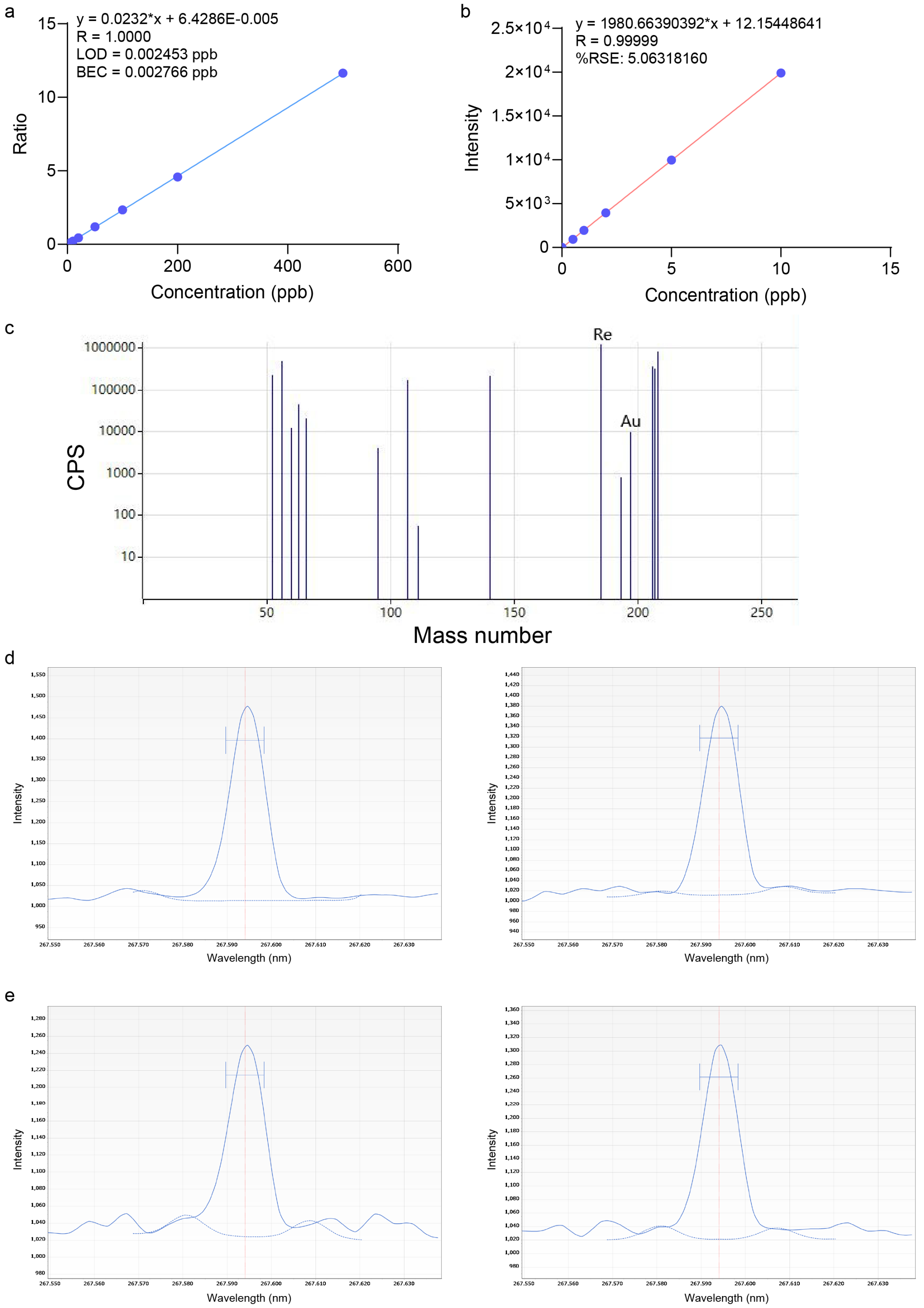

Supplementary Fig. 7 Quantitative determination of the gold content using ICP-OES/MS elemental analysis. a, b Standard calibration curve for gold element determination by (**a**) ICP-MS and (**b**) ICP-OES. **c** Mass spectrum of gold element in control nanoparticles determined by ICP-MS. **d, e** Peak profile of gold element in (**d**) symmetrical nanoparticles and (**e**) NeuroConnect Blocks

determined by ICP-OES.

In addition to TEM (selected particles) and DLS (size), it would be beneficial with composition analysis of the final nanocarrier and the control nanocarrier described in table 2 supp.

Responses: We sincerely thank you for your kind advice. Your suggestion regarding the need for further analysis of the composition of the final nanocarrier and its control counterpart is indeed very important. We fully agree with your point, and have addressed this in the revised manuscript with additional analyses and clarifications (Supplementary Tab. 4).

The details are as follows:

Given that LMK-235 itself constitutes a core component of both the final nanocarrier and the control nanocarrier, we have supplemented information on its encapsulation efficiency and drug loading, as determined by HPLC, to directly demonstrate the successful incorporation of the drug molecules into the nanocarrier as part of its final composition (Supplementary Tab. 4).

In addition, for components of the final and control nanocarriers that are difficult to distinguish solely by elemental analysis due to their similarity, we focused on the systematic changes in their physicochemical properties as indirect evidence of composition. For example, the incorporation of LMK-235, polyaniline, bis-5HT, and other polymeric materials led to predictable and consistent alterations in *Zeta* potential and hydrodynamic size. These changes align precisely with the expected outcomes of each step of chemical modification, thereby providing strong evidence that all functional components were successfully integrated into the nanocarrier (Supplementary Tab. 4).

Supplementary Tab. 4 The detailed information on the control and the final nanocarrier.

Nanoparticles	Size (nm)	PDI	*Zeta* potential (mV)	EE (%)	LE (%)
**LMK-235@PANI NPs**	160.9	0.115	-5.5	74.12	7.11
**LMK-235@Bis-5HT/PANI NPs**	208.7	0.126	-6.2	71.18	5.72
**Bis-5HT/PANI Janus NPs**	201.3	0.155	-5.9	0	0
**LMK-235@Bis-5HT Janus NPs**	186.6	0.146	-36.9	75.71	7.09
**NeuroConnect Blocks**	205.4	0.157	-5.7	70.56	5.88

The authors claim substantial aggregation *in vitro* using MPO/hydrogen peroxide in figure 3a/b DLS and ocular analysis. However, the DLS technique does not scale with size thus it is not possible to compare peak sizes. Scattered light intensity scales with the sixth power of particle diameter, meaning larger particles dominate the signal, even if they're rare. This leads to overrepresentation of large particles in the size distribution. Thus, further analysis to quantify the aggregation is necessary.

Responses: We greatly appreciate your suggestion. We fully agree with your perspective. Accordingly, to accurately characterize the particle size distribution, we have included nanoparticle tracking analysis (NTA) in the manuscript to further quantify the aggregation (Supplementary Fig. 8).

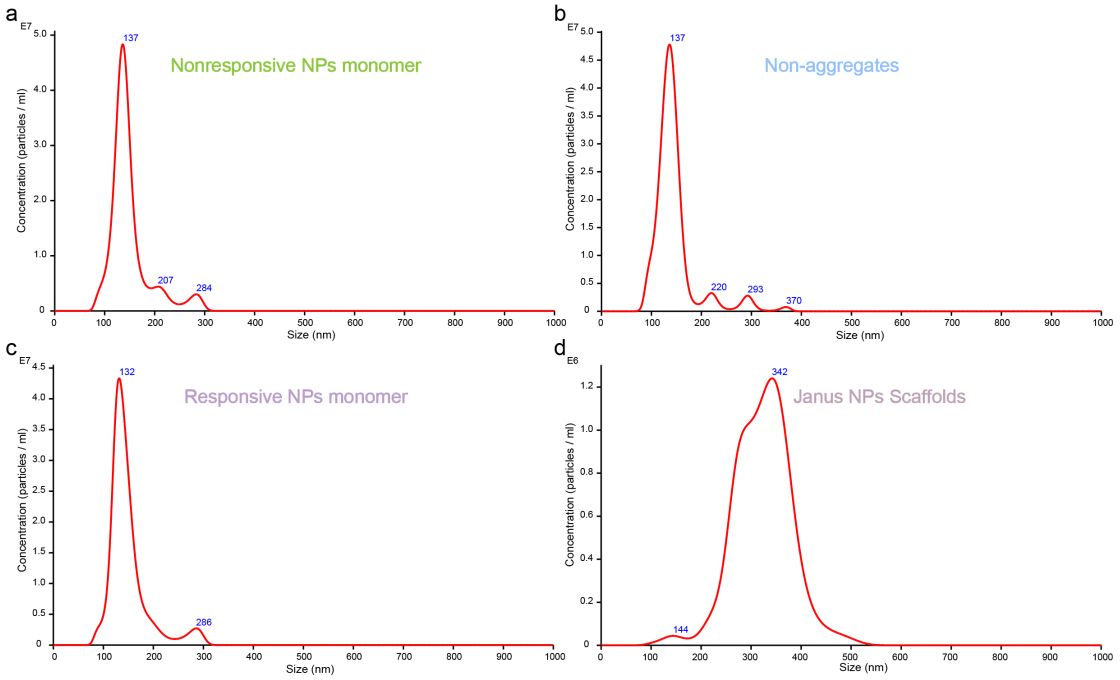

Supplementary Fig. 8 Quantitative determination of the gold content using ICP-OES/MS elemental analysis. a, b NTA analysis of non-responsive nanoparticles in (**a**) PBS or in (**b**) medium containing MPO and H_2O_2. **c, d** NTA analysis of responsive nanoparticles in (**c**) PBS or in (**d**) medium containing MPO and H_2O_2.

In figure 4 c there is an increase of the signal in the head of the mouse but nowhere else. In 4e most of the material is in other organs foremost the liver? Could the authors comment on this. It would be relevant to see the distribution in main organs at earlier time points (0-48 h).

Responses: We sincerely appreciate your insightful questions and constructive feedback. The exclusive enhancement of fluorescence in the mouse head is attributable to the greater ability of near-infrared signals to penetrate and reach brain tissue compared with other organs, thereby generating detectable fluorescence. The observed increased accumulation of NeuroConnect Blocks across all organs is likely due to their enhanced circulation time in the bloodstream, as demonstrated by our pharmacokinetic evaluation (Supplementary Fig. 16).

A detailed explanation is provided as follow:

1. The reason for the exclusive enhancement of fluorescence in the mouse head:

The enhanced fluorescence observed in the mouse head, but not in other regions, can be attributed to two factors: first, the monitoring was performed with the mouse in a prone position; second, the fur on the head had been shaved for surgical procedures. Under these conditions, near-infrared fluorescence penetrates more readily and reaches the brain compared with other organs. Consequently, at the applied fluorescence threshold, only signals from the head are detectable, primarily reflecting the accumulation of nanoparticles in the brain.

2. The reason for the increased accumulation of NeuroConnect Blocks across all organs: In comparison to the control formulation, NeuroConnect Blocks were modified with a PEG_{2000} segment (it was introduced by SH-PEG_{2000}-NHS), which likely enhanced their circulation, contributing to increased accumulation in various organs. To demonstrate the long-circulation capability of NeuroConnect Blocks, we have conducted a pharmacokinetic evaluation. The results indicated that the plasma half-life of NeuroConnect Blocks was significantly prolonged compared to the control nanoparticles (Supplementary Fig. 16).

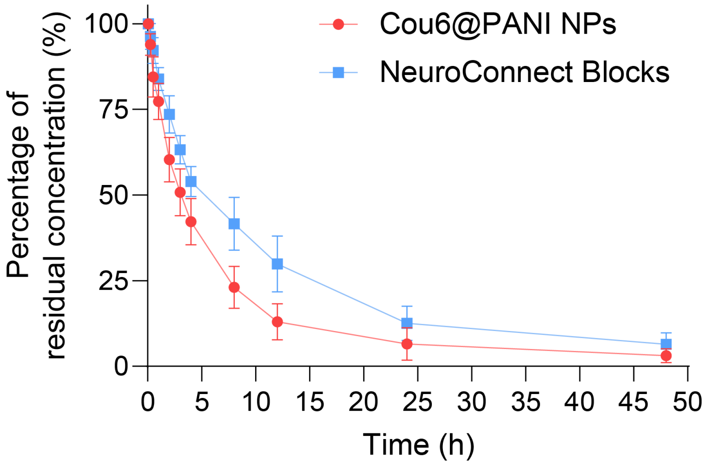

Supplementary Fig. 16 The pharmacokinetic properties of NeuroConnect Blocks scaffolds (n = 3). Data represent mean ± SD of three independent biological replicates.

Since the nanocarriers mainly end up in the liver, would it not be relevant to perform toxicity test on liver cells.

Responses: We greatly appreciate your insightful and kind advice. Accordingly, we have incorporated liver function-related biochemical indices into the revised

manuscript to evaluate the hepatotoxicity of NeuroConnect Blocks (Supplementary Fig. 46e, f) (***Location of Changes:*** *Line 667-669 in page 31*).

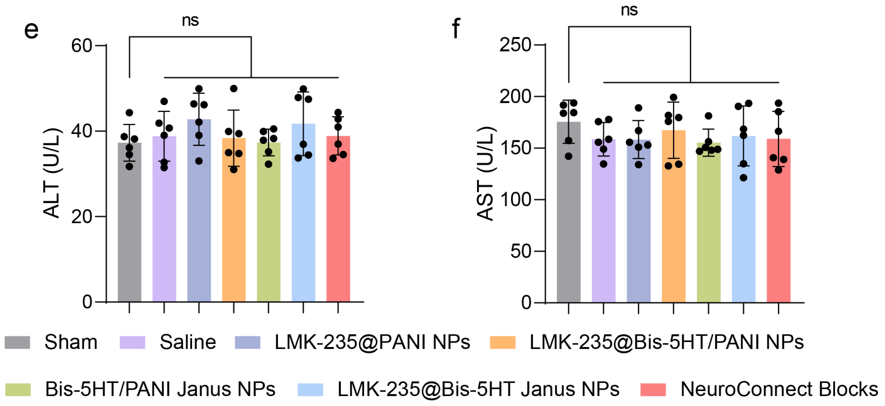

Supplementary Fig. 46 Hematological and biochemical analyses. e, f Assessments of liver and kidney function, including the serum levels of (**e**) ALT and (**f**) AST. Data represent mean ± SD of six independent biological replicates. Statistical analyses are performed using one-way ANOVA with Tukey's post hoc test.

References

1. Buszman, P.P., *et al.* Implantation feasibility and early vascular response of a novel PLLA/PLGA bioresorbable scaffold in porcine coronary in-stent restenosis model. *J. Am. Coll. Cardiol.* **75**, 1351-1351 (2020).
2. Dou, Z., *et al.* A "built-up" composite film with synergistic functionalities on Mg–2Zn–1Mn bioresorbable stents improves corrosion control effects and biocompatibility. *Bioact. Mater.* **25**, 223-238 (2023).
3. Martins, C., Sousa, F., Araújo, F. & Sarmento, B. Functionalizing PLGA and PLGA derivatives for drug delivery and tissue regeneration applications. *Adv. Healthc. Mater.* **7**, 1701035 (2018).
4. Zhang, Y., *et al.* Advances in bioresorbable materials and electronics. *Chem. Rev.* **123**, 11722-11773 (2023).
5. Yu, X., Shou, W., Mahajan, B.K., Huang, X. & Pan, H. Materials, processes, and facile manufacturing for bioresorbable electronics: a review. *Adv. Mater.* **30**, 1707624 (2018).
6. Shankar, R. & Mishra, O. Effect of reserpine on cationic contents of rat brain. *Nature* **251**, 532-532 (1974).

7. Schulz, R., Cartwright, C. & Goldstein, A. Reversibility of morphine tolerance and dependence in guinea pig brain and myenteric plexus. *Nature* **251**, 329-331 (1974).
8. McClelland, L.J., Mou, T.-C., Jeakins-Cooley, M.E., Sprang, S.R. & Bowler, B.E. Structure of a mitochondrial cytochrome c conformer competent for peroxidase activity. *Proc. Natl. Acad. Sci. USA* **111**, 6648-6653 (2014).
9. Chen, J.W., Querol Sans, M., Bogdanov Jr, A. & Weissleder, R. Imaging of myeloperoxidase in mice by using novel amplifiable paramagnetic substrates. *Radiology* **240**, 473-481 (2006).
10. Pulli, B., *et al.* Molecular MR imaging of myeloperoxidase distinguishes steatosis from steatohepatitis in nonalcoholic fatty liver disease. *Radiology* **284**, 390-400 (2017).
11. Wang, C., *et al.* A versatile imaging platform with fluorescence and CT imaging capabilities that detects myeloperoxidase activity and inflammation at different scales. *Theranostics* **9**, 7525 (2019).
12. Gorren, A., Dekker, H. & Wever, R. Kinetic investigations of the reactions of cytochrome c oxidase with hydrogen peroxide. *Biochim. Biophys. Acta* **852**, 81-92 (1986).
13. Prasad, S., Maiti, N.C., Mazumdar, S. & Mitra, S. Reaction of hydrogen peroxide and peroxidase activity in carboxymethylated cytochrome c: spectroscopic and kinetic studies. *Biochim. Biophys. Acta* **1596**, 63-75 (2002).
14. Lei, B., Adachi, N. & Arai, T. Measurement of the extracellular H2O2 in the brain by microdialysis. *Brain Res. Brain Res. Protoc.* **3**, 33-36 (1998).
15. Carlos, T., Clark, R., Franicola-Higgins, D., Schiding, J. & Kochanek, P. Expression of endothelial adhesion molecules and recruitment of neutrophils after traumatic brain injury in rats. *J. Leukoc. Biol.* **61**, 279-285 (1997).
16. Lin, W., Chen, H., Chen, X. & Guo, C. The roles of neutrophil-derived myeloperoxidase (MPO) in diseases: the new progress. *Antioxidants* **13**, 132 (2024).
17. Li, W., *et al.* BBB pathophysiology–independent delivery of siRNA in traumatic brain injury. *Sci. Adv.* **7**, eabd6889 (2021).
18. Liu, Y.-W., Li, S. & Dai, S.-S. Neutrophils in traumatic brain injury (TBI): friend or foe? *J. Neuroinflammation* **15**, 146 (2018).
19. Kenne, E., Erlandsson, A., Lindbom, L., Hillered, L. & Clausen, F. Neutrophil depletion

reduces edema formation and tissue loss following traumatic brain injury in mice. *J. Neuroinflammation* **9**, 17 (2012).

20. Darakchiev, B.J., Itkis, M., Agajanova, T., Itkis, A. & Hariri, R. Changes of MPO activity and brain water accumulation in traumatic brain injury experiments. *Acta Neurochir. Suppl.* **70**, 98-101 (1997).

21. Needham, E.*, et al.* The immunological response to traumatic brain injury. *J. Neuroimmunol.* **332**, 112-125 (2019).

22. Ebeid, K.*, et al.* Synthetically lethal nanoparticles for treatment of endometrial cancer. *Nat. Nanotechnol.* **13**, 72-81 (2018).

23. Mikhaylov, G.*, et al.* Ferri-liposomes as an MRI-visible drug-delivery system for targeting tumours and their microenvironment. *Nat. Nanotechnol.* **6**, 594-602 (2011).

24. Krencik, R., Weick, J.P., Liu, Y., Zhang, Z.-J. & Zhang, S.-C. Specification of transplantable astroglial subtypes from human pluripotent stem cells. *Nat. Biotechnol.* **29**, 528-534 (2011).

25. Nagao, M., Ogata, T., Sawada, Y. & Gotoh, Y. Zbtb20 promotes astrocytogenesis during neocortical development. *Nat. Commun.* **7**, 11102 (2016).

26. Cai, Q., Shi, G., Bei, J. & Wang, S. Enzymatic degradation behavior and mechanism of poly (lactide-co-glycolide) foams by trypsin. *Biomaterials* **24**, 629-638 (2003).

27. Pan, H., Jiang, H. & Chen, W. The biodegradability of electrospun Dextran/PLGA scaffold in a fibroblast/macrophage co-culture. *Biomaterials* **29**, 1583-1592 (2008).

28. Cho, Y., Sloutsky, R., Naegle, K.M. & Cavalli, V. Injury-induced HDAC5 nuclear export is essential for axon regeneration. *Cell* **155**, 894-908 (2013).

29. Park, S.M.*, et al.* Effect of ischemic preconditioning on antioxidant status in the gerbil hippocampal CA1 region after transient forebrain ischemia. *Neural Regen. Res.* **11**, 1081-1089 (2016).

30. Fang, Q., Xu, T., Wu, C., Zhou, S. & Sun, H. Biotargets in neural regeneration. *Biotarget* **1**, 6 (2017).

31. Shlosberg, D., Benifla, M., Kaufer, D. & Friedman, A. Blood–brain barrier breakdown as a therapeutic target in traumatic brain injury. *Nat. Rev. Neurol.* **6**, 393-403 (2010).

32. Cruz, L.J.*, et al.* Effect of PLGA NP size on efficiency to target traumatic brain injury. *J. Control. Release.* **223**, 31-41 (2016).

33. Qin, Y., *et al.* Harnessing oxidative microenvironment for in vivo synthesis of subcellular conductive polymer microesicles enhances nerve reconstruction. *Nano Lett.* **22**, 3825-3831 (2022).

34. Dane, E.L., *et al.* STING agonist delivery by tumour-penetrating PEG-lipid nanodiscs primes robust anticancer immunity. *Nat. Mater.* **21**, 710-720 (2022).

35. Chen, Y., Su, Y.-C. & Roffler, S.R. Polyethylene glycol immunogenicity in nanomedicine. *Nat. Rev. Bioeng*. **3**, 742-760 (2025).

36. Wang, Y., *et al.* Enhanced dispersion stability of gold nanoparticles by the physisorption of cyclic poly (ethylene glycol). *Nat. Commun.* **11**, 6089 (2020).

37. Dewey, D.C., Strulson, C.A., Cacace, D.N., Bevilacqua, P.C. & Keating, C.D. Bioreactor droplets from liposome-stabilized all-aqueous emulsions. *Nat. Commun.* **5**, 4670 (2014).

38. Zhao, Y., *et al.* Polymer-locking fusogenic liposomes for glioblastoma-targeted siRNA delivery and CRISPR–Cas gene editing. *Nat. Nanotechnol.* **19**, 1869-1879 (2024).

39. Yang, Y., *et al.* Self-assembly of size-controlled liposomes on DNA nanotemplates. *Nat. Chem.* **8**, 476-483 (2016).

Point-by-Point Response to Reviewer Comments

Manuscript Number: NCOMMS-25-44797B

Title: Self-assembling scaffolds epigenetically reactivate and electroactively guide neuronal regeneration to restore central neural circuits

Reviewer #1 (Remarks to the Author):

I would like to appreciate that the authors replied and modified the manuscript according to the comments. I have seen their responses and modifications. However, I still recommend that the authors modify their statements in which they mention that "conductive" PANI is crucial for neuronal conduction in this case, because in that case a reader will assume that the property that PANI is mixed conductor is necessary for the neuronal signal to propagate. If the authors claim this, to my understanding this is wrong, or not proved in this manuscript. In the response they mention that in Fig 5 the Janus NPs show decreased neuronal growth compared to Neuroconnect blocks, but the difference is non significant (ns) as they mention. Moreover, figure 9 shows the behavioral tests in which there is some significance but still both control with Janus and Neuroconnects are showing good results, and it can't be extrapolated from behavioral studies facilitation of neuronal activity by a conducting PANI layer. Conduction may be facilitated but the claim "facilitated by a conductive PANI layer" can be misleading. A simple fix to the claim is to remove the "conductive" term from their claim and mention that Neuroconnect blocks seem to not prevent neuronal signaling, possibly due to incorporation of PANI.

Responses: We sincerely thank the reviewer for this thoughtful and technically important comments. We agree that our previous wording could be read as implying that bulk electronic conduction through polyaniline (PANI) is required for action potential propagation or for synaptic transmission to occur—an interpretation that is not directly tested in our study and is not the mechanistic claim we intend to make. In the CNS, neuronal signaling is fundamentally supported by ionic currents across neuronal membranes, and our data do not demonstrate that long-range signal propagation is mediated through the scaffold material. We therefore revised the manuscript to (i) remove potentially misleading "conductive PANI enables neuronal conduction" language, (ii) align all statements with what is experimentally supported, and (iii) explicitly delimit what remains unresolved.

Modifications made in the revised manuscript:

1. Title modification (*Location of Changes:* *Line 1-2 in page 1*):

We have revised the title from: "Self-assembling scaffolds epigenetically reactivate and electrically guide neuronal regeneration to restore central neural circuits" to: "Self-assembling scaffolds epigenetically reactivate and electroactively guide neuronal regeneration to restore central neural circuits". This change avoids the implication that bulk electrical conduction is the primary mechanism and instead emphasizes the broader electroactive properties of the PANI layer (***Location of Changes:*** *Line 1-2 in page 1*).

2. Text revisions to remove the implication that bulk conductivity is required for neuronal signal propagation (*Location of Changes:* *Line 52-54 in page 2; Line 107-109 in page 4; Line 127-128 in page 6; Line 390-396 in page 16; Line 571-578 in page 22; Line 579-585 in page 23*)**:** Across the manuscript, we have replaced wording that could be interpreted as "neuronal signaling is facilitated by a conductive PANI layer" with language consistent with what is directly supported by our data. Specifically, we have described PANI as an electroactive, electrically permissive interface, and we have avoided attributing the observed functional recovery to bulk electronic conduction through the scaffold (***Location of Changes:*** *Line 52-54 in page 2; Line 107-109 in page 4; Line 127-128 in page 6*). Furthermore, we have added/retained an explicit clarification (in the Results mechanistic section (***Location of Changes:*** *Line 390-396 in page 16*) and mirrored in the Discussion (***Location of Changes:*** *Line 571-578 in page 22; Line 579-585 in page 23*)) that PANI alters both electroactive/electrical interfacial properties and surface physicochemistry, and therefore our current data cannot disentangle electrical permissiveness from charge-/chemistry-mediated cell–material interactions. Alternatively, we have interpreted the PANI layer as an electroactive interface that does not appear to impede neuronal activity-dependent repair, rather than claiming it "facilitates neuronal conduction". This directly addresses the reviewer's concern that readers might assume PANI's mixed-conductor property is required for neuronal signal propagation—an inference we agree is not demonstrated here.

3. Janus control comparisons no longer used to support a "conductivity is crucial" claim: We agree with the reviewer's point that the non-significant (ns) comparisons (e.g., certain readouts comparing Janus controls vs NeuroConnect Blocks) should not be used to support strong mechanistic claims about conductivity-dependent neuronal signaling. Accordingly, we removed or softened statements that relied on these comparisons to imply that "conducting PANI is crucial for neuronal conduction." Where differences are modest or ns, we now present them as compatibility/permissiveness observations rather than causal evidence.

4. Behavioral interpretation revised to avoid mechanistic over-attribution (*Location of Changes:* ***Line 390-396 in page 16; Line 572-578 in page 22; Line 579-581 in page 23***):** We have revised relevant text to clarify that behavioral recovery demonstrates functional improvement and that Kir2.1 experiments support a requirement for neuronal activity for full recovery, but we explicitly stated that these data do not establish that recovery is mediated by action potential propagation through (or because of) a "conductive" PANI layer (***Location of Changes:*** *Line 390-396 in page 16; Line 572-578 in page 22; Line 579-581 in page 23*).

5. Limitations strengthened to explicitly acknowledge what is not resolved and what controls are needed (*Location of Changes:* *Line 571-578 in page 22; Line 579-585 in page 23***):** We have strengthened the limitations/future work statements to note that dissecting the relative contributions of electronic/ionic transport, interfacial capacitance, charge-mediated adhesion, and protein-conditioned surface interactions is an important next step, and that our current work supports an electroactive, electrically permissive interface rather than a definitive conduction-based mechanism (***Location of Changes:*** *Line 571-578 in page 22; Line 579-585 in page 23*).

In line with the reviewer's proposed "simple fix," we now avoid framing the effect as "facilitated by a conductive PANI layer" and instead state that NeuroConnect Blocks do not appear to impede neuronal activity-dependent repair, plausibly due to incorporation of a PANI-based electroactive interface—while emphasizing that the precise physical basis (conductivity vs surface chemistry vs both) remains to be resolved.

We hope these revisions address the reviewer's central concern by removing potentially misleading phrasing, preventing over-interpretation of behavioral outcomes, and aligning mechanistic statements with the level of evidence provided in this manuscript.

Reviewer #2 (Remarks to the Author):

All changes have been appropriately addressed and significantly approved the manuscript.

Responses: We sincerely thank the reviewer for the kind assistance and professional suggestions.

Reviewer #3 (Remarks to the Author):

I co-reviewed this manuscript with one of the reviewers who provided the listed reports. This is part of the Nature Communications initiative to facilitate training in peer review and to provide appropriate

recognition for Early Career Researchers who co-review manuscripts.

Responses: Thank you very much for your insightful comments, which significantly improve the quality of our manuscript.

Reviewer #4 (Remarks to the Author):

1. The manuscript is very extensive and the authors should be more selective and focus on the most relevant results.

Responses: We sincerely appreciate your kind advice. We have reorganized the data according to your suggestions, making the manuscript more selective and focused on the relevant results while preserving its overall integrity. Specifically, we have included the in vitro results demonstrating the ability of NeuroConnect Blocks scaffolds to resist rapid cellular uptake and clearance in the Supplementary Information, in order to enhance the focus of the experimental findings presented in the main manuscript (Supplementary Fig. 14).

Comment: The authors have not reduced the manuscript to a comprehensible format. In fact, only limited changes to the original manuscript. The manuscript is still 49 pages and contain 12 figures. They have not adhered to the comment. This needs to be substantially reduced and figures moved to SI section. *The above content comprises the reviewers' comments from the first two rounds of peer review, while the following section presents our responses to the comments from the previous round.*

Responses: We thank the reviewer for emphasizing the need for improved selectivity and readability. In this revision, we undertook a substantial restructuring and condensation of the manuscript to focus the main text on the most essential findings and to move supporting/confirmatory datasets to the Supplementary Information. Concretely, we implemented the following changes:

1) Substantial reduction of manuscript length and complexity. The manuscript was shortened from 49 pages to 22 pages (formatted according to the journal template). The number of main-text figures was reduced from 12 to 8, with redundant panels consolidated and several datasets reallocated to the Supplementary Information.

2) Major streamlining of the Results section. The Results section (including main-figure legends) was reduced from 8,067 words to 4,132 words (≈49% reduction). The number of Results subsections was reduced from 12 to 7 by merging related experiments into integrated, figure-aligned sections and removing repetitive transitional text.

3) Reallocation of non-core and confirmatory datasets to the Supplementary Information. To keep the main narrative focused while preserving rigor, we moved several categories of supportive data to the Supplementary Information, including (where appropriate): expanded characterization/controls, additional validation panels, and experiments that reinforce but are not required to establish the central conclusions (e.g., selected in vitro uptake/clearance datasets and other extended control analyses). This reduces the density of the main text while maintaining transparency and completeness for readers.

4) Improved narrative focus. The revised main text now prioritizes a single, coherent mechanistic arc: injury-responsive scaffold formation → lesion residence and sustained drug exposure → epigenetic reactivation and microenvironmental remodeling → functional synaptic/circuit recovery, with the eight main figures serving as the primary organizational backbone.

2. Either the authors need to demonstrate that it is the HDAC5 inhibitory effect that contribute to the regeneration or rephrase throughout the manuscript.

Responses: We sincerely appreciate your highly professional and important comments. You correctly pointed out that LMK-235 exhibits comparable inhibitory activity against HDAC4 and HDAC5, and that other HDAC isoforms may carry potential off-target risks at locally elevated concentrations. We fully agree with your view. In response to your comment, we have taken the following steps: 1. Comprehensive revision of the manuscript: We have carefully revised the relevant statements throughout the manuscript, avoiding absolute terms such as “selective HDAC5 inhibitor”. Instead, we now employ more precise and rigorous descriptions (Location of Changes: Line 41 in page 2; Line 105-106 in page 4; Line 359 in page 15; Line 725 in page 34; Line 990 in page 46). In addition, we have added a dedicated paragraph in the Discussion to specifically address the issue of LMK-235 selectivity, transparently analyzing the correlation between its inhibitory activity on HDAC5 and the observed outcomes in neural regeneration (Location of Changes: Line 1022-1027 in page 47; Line 1028-1030 in page 48). 2. Inference regarding the role of HDAC5: Although LMK-235 demonstrates similar inhibitory activity against HDAC4 and HDAC5, we infer that HDAC5 inhibition plays a more critical role in our model based on the following considerations: A) Evidence from the literature: Multiple independent studies have directly linked the loss or inhibition of HDAC5 with enhanced axonal regeneration and neuronal survival, supporting HDAC5 as a more specific regulatory target for neural regeneration28-30. B) Specificity within our model: Our experimental data show that LMK-

235 treatment induces distinct phenotypes—such as enhanced axonal regeneration and upregulation of HDAC5-associated transcription factors including Jun, Fos, and Klf6. These phenotypes closely align with those reported for HDAC5 loss-of-function, rather than HDAC428. C) Concentration considerations: The dosing regimen we employed was carefully designed to achieve effective drug concentrations in the target tissue within the low nanomolar range. At this concentration, the inhibitory effects of LMK-235 on other isoforms are expected to be negligible. Therefore, the main off-target concern lies in the inability to clearly distinguish between HDAC4 and HDAC5. On this basis, we have incorporated additional discussion in the manuscript to strengthen the rigor of the study (Location of Changes: Line 1022-1027 in page 47; Line 1028-1030 in page 48). Once again, we are deeply grateful for this constructive feedback, which has greatly helped us improve the scientific robustness of our work.

Comment: The authors' claim that HDAC5 inhibition specifically drives the observed neural regeneration phenotypes is not adequately supported. While the cited literature (Cho & Cavalli, 2013) demonstrates HDAC5 role in peripheral nerve regeneration, these studies did not test whether HDAC4 inhibition would produce similar effects. Given that LMK-235 inhibits HDAC4 and HDAC5 with comparable potency, HDAC4 and HDAC5 both repress MEF2-dependent transcription including immediate early genes, and recent work demonstrates functional redundancy between HDAC4 and HDAC5 in neuronal gene regulation (Zhu et al., 2019), the claim that the observed effects are HDAC5-specific requires direct experimental validation or the use of HDAC4-selective inhibitors such as Tasquinimod.

References for support

Fitzsimons HL, Schwartz S, Given FM, Scott MJ. The histone deacetylase HDAC4 regulates long-term memory in Drosophila. PLoS One. 2013 Dec 9;8(12):e83903. doi: 10.1371/journal.pone.0083903. PMID: 24349558; PMCID: PMC3857321.

Zhu, Y., Huang, M., Bushong, E. et al. Class IIa HDACs regulate learning and memory through dynamic experience-dependent repression of transcription. Nat Commun 10, 3469 (2019). https://doi.org/10.1038/s41467-019-11409-0

Zhu et al demonstrate that HDAC4 and HDAC5 redundantly act in the same pathway that is essential for spatiotemporal control of ERG programs.

Sando R 3rd, Gounko N, Pieraut S, Liao L, Yates J 3rd, Maximov A. HDAC4 governs a transcriptional

program essential for synaptic plasticity and memory. Cell. 2012 Nov 9;151(4):821-834. doi: 10.1016/j.cell.2012.09.037. PMID: 23141539; PMCID: PMC3496186.

And the authors revision from selective HDAC5 inhibitor to HDAC5 inhibitor does not change the interpretation by the reader. HDAC5 is mentioned 36 times and HDAC4 two times in the manuscript, Since the authors argue that the HDAC5 inhibition is the most important pharmacological effect of LMK-235, I suggest experimental validation to confirm this view. *The above content comprises the reviewers' comments from the first two rounds of peer review, while the following section presents our responses to the comments from the previous round.*

Responses: We sincerely thank the reviewer for the professional comments and constructive suggestions. In response to this point, we have treated primary neurons with the HDAC4-selective inhibitor Tasquinimod and compared its effects on axonal regeneration with those of LMK-235, in order to determine whether inhibition of HDAC5 is more likely to underlie the pro-regenerative activity of LMK-235 (Supplementary Fig. 30) (***Location of Changes in Results:*** *Line 373-377 in page 16;* ***Location of Changes in Discussion:*** *Line 505-515 in page 21*).

Specifically, primary cortical neurons subjected to OGD/R injury were co-incubated with 10 nM LMK-235 or Tasquinimod for 24 h, followed by evaluation of axonal regeneration. The results showed that LMK-235 increased the level of the axonal marker SMI-312 by 18.37% in injured neurons, whereas tasquinimod did not exhibit a significant axon regeneration-promoting effect. Although these results do not exclude potential contributions from combined class IIa HDAC inhibition, they indicate that selective HDAC4 inhibition alone is insufficient to elicit a significant pro-regenerative effect in our model and are consistent with a predominant role of HDAC5-related mechanisms during regeneration (Supplementary Fig. 30).

In addition, to improve the rigor and accuracy of the manuscript, we have revised the terminology throughout the text from "HDAC5 inhibitor LMK-235" to "class IIa HDAC4/5-selective inhibitor LMK-235" or "class IIa HDAC inhibition" (***Location of Changes:*** *Line 47-48 in page 2; Line 104-105 in page 4; Line 362 in page 15; Line 365 in page 15; Line 506-507 in page 21*).

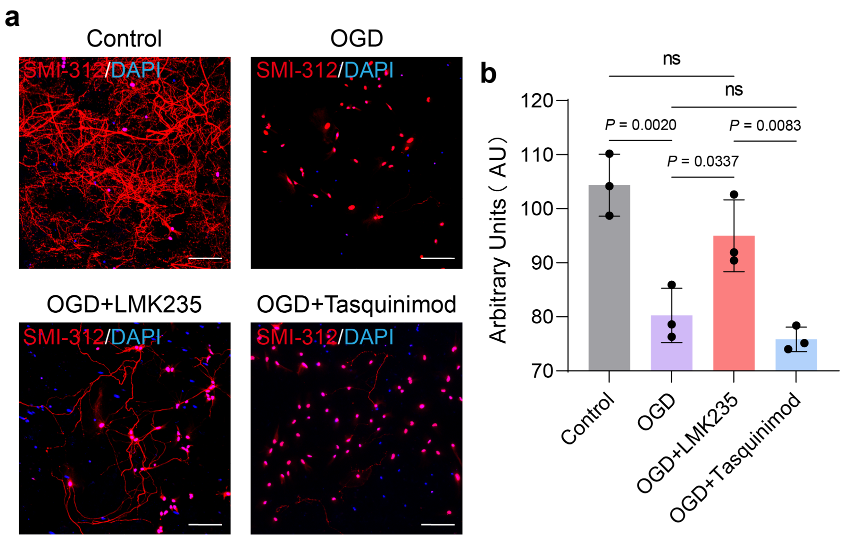

Supplementary Fig. 30 Comparative analysis of pro-axonal regeneration effects of LMK-235 versus tasquinimod. a Representative images of axonal regeneration in injured primary cortical neurons after treatment with 10 nM LMK-235 or tasquinimod. Scale, 100 μm. **b** Quantitative analysis of axonal outgrowth in primary cortical neurons shown in (**a**) (n = 3). Data represent mean ± SD of three independent biological replicates. Statistical analyses are performed using one-way ANOVA with Tukey's post hoc test.

3. The nanocarrier is reported to be approximately 200 nm. Previous studies have found that nanoparticles around 30 nm are optimal for TBI application and 80 nm remains in the blood vessel. Could the authors comment on this. See Size-Selective Transfer of Lipid Nanoparticle-Based Drug Carriers Across the Blood Brain Barrier Via Vascular Occlusions Following Traumatic Brain Injury

Responses: Thank you for your valuable comments. The findings reported in the reference you provided were obtained using a moderate TBI model (impact velocity: 6 m/s, impact depth: 0.5 mm, contact time: 150 ms), whereas our study employed a severe TBI model (impact velocity: 3.5 m/s, impact depth: 1 mm, contact time: 400 ms). The degree of BBB permeability following TBI is largely determined by the extent of both primary and secondary injury31. Therefore, given the more severe injury model used in our study, it is entirely plausible that NeuroConnect Blocks particles of approximately 200 nm in size were able to penetrate the BBB and accumulate within the brain. Moreover, dedicated studies have demonstrated that PLGA nanoparticles ranging from 100 nm to 800 nm are capable of accumulating in the brain after TBI32.

Comment: The authors claim that their model produces a more severe injury, yet the reported impact velocity is lower. It would be helpful to clarify how a reduced velocity leads to increased

severity, given that velocity is typically a major contributor of impact energy. Are there other parameters such as impact depth, dwell time, or tip geometry that could account for the heightened injury? It may also strengthen the argument to confirm the severity using established biomarkers of tissue damage or neuroinflammation. *The above content comprises the reviewers' comments from the first two rounds of peer review, while the following section presents our responses to the comments from the previous round.*

Responses: Thank you for the kind comments and valuable suggestions. Impact depth directly determines the maximum compressive strain and the spatial extent of stress propagation, thereby defining the anatomical depth and range of tissue injury. Dwell time is directly associated with the duration of strain experienced by brain tissue, which exacerbates vasogenic injury and local ischemia, markedly aggravates BBB disruption, and influences both the initiation and magnitude of secondary injury cascades. Therefore, although a lower impact velocity was applied in our study, the impact depth and dwell time were substantially increased, resulting in more severe brain tissue damage and BBB disruption. Furthermore, in accordance with your suggestion, we have directly compared the extent of BBB disruption induced by our modeling protocol (impact velocity: 3.5 m/s, impact depth: 1 mm, contact time: 400 ms) with that reported in the referenced study (impact velocity: 6 m/s, impact depth: 0.5 mm, contact time: 150 ms) using the Evans blue extravasation assay (Supplementary Fig. 12). The results demonstrated that our model produced more pronounced BBB disruption, thereby supporting the feasibility of NeuroConnect Blocks crossing the BBB and accumulating within brain tissue (***Location of Changes in Results:*** *Line 240-242 in page 10;* ***Location of Changes in Discussion:*** *Line 542-548 in page 22*).

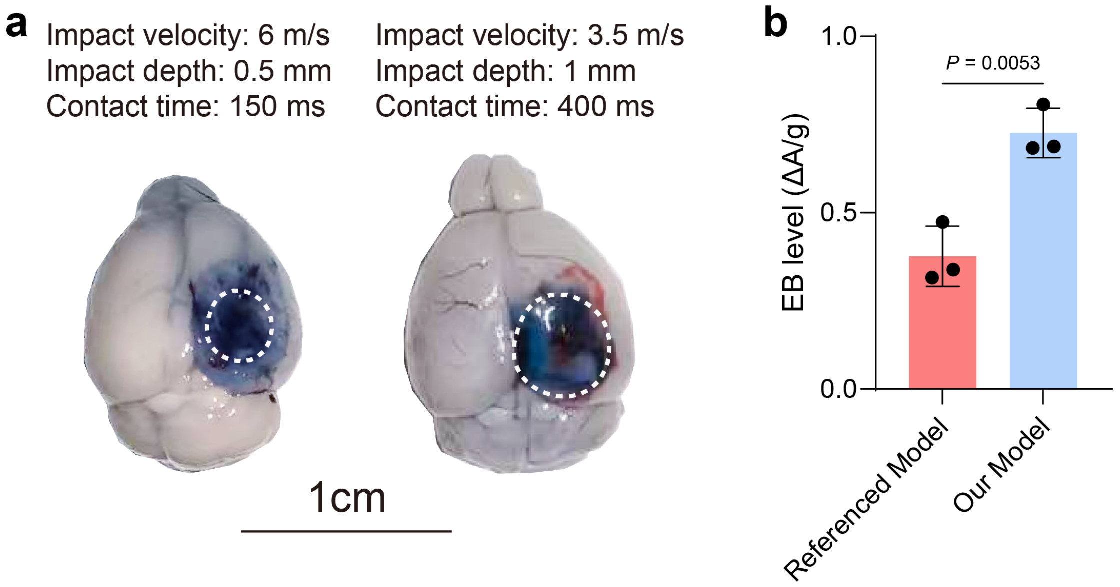

Supplementary Fig. 12 Analysis of Evans blue extravasation in the brain. a, b Representative images (**a**) and quantitative analysis (**b**) of Evans blue leakage in the brain under different CCI parameters (n = 3). Data represent mean ± SD of three independent biological replicates. Statistical analyses are performed using one-way ANOVA with Tukey's post hoc test.

4. What is hydrogen peroxide enzyme in the PANI step?

Responses: We appreciate your feedback. In our study, hydrogen peroxide enzyme was employed to catalyze the in situ polymerization of aniline. Under the presence of H_2O_2, the heme cofactor (Fe3+) of catalase is first oxidized by H_2O_2 to generate a highly reactive intermediate. This intermediate can abstract an electron from an aniline molecule, forming an aniline radical cation, which in turn efficiently promotes the in situ polymerization of aniline to yield PANI33. Accordingly, we have incorporated the relevant explanation and references into the manuscript (Location of Changes: Line 1180-1185 in page 53).

Comment: There are several enzymes that use or degrade H_2O_2 thus the expression hydrogen peroxide enzyme is unspecific. State the enzyme and include the quality+vendor in the materials. For the generation of PANI the most common is HRP and multiple references cover this process. The author claim that they use catalase which is degrading hydrogen peroxide to water which is unusual and with limited support. The mechanism describe in the response by the authors that abstracts electrons from a substrate is more characteristic of peroxidases like HRP, not catalase. *The above content comprises the reviewers' comments from the first two rounds of peer review, while the following*

section presents our responses to the comments from the previous round.

Responses: We appreciate your professional comments. Indeed, in terms of catalytic efficiency for the polymerization of aniline alone, HRP is generally a more suitable catalyst than CAT, as HRP possesses a binding pocket near its active site that accommodates aromatic molecules, facilitating electron transfer from aniline to the oxidized intermediate. In our study, the selection of CAT to catalyze the surface polymerization of polyaniline on nanoparticles was based on the following rational considerations. ***First***, to effectively control the size of the conductive nanoparticles, the polyaniline layer on their surface must be ultrathin and uniform. A slow initiation rate is ideal for forming such a homogeneous thin layer, whereas the rapid polymerization mediated by HRP is difficult to regulate and often leads to nonuniform deposition and localized overgrowth. ***Second***, the negatively charged carboxyl groups within the nanoparticle framework can efficiently adsorb aniline monomers that are preintroduced into the reaction system, creating a locally high concentration of aniline at the nanoparticle surface. This markedly increases the probability that oxidized intermediates of CAT present at the nanoparticle surface capture electrons from aniline monomers, thereby enabling controlled and spatially confined polymerization of aniline on the nanoparticle surface. Moreover, this preparation strategy is supported and corroborated by previous reports in the literature[1].

5. The authors report conductivity of the nanoparticles in figure 2D but does not describe the procedure neither do they show that the conductivity of the PANI layer is crucial for the system considering that the title include “electrically guide..”.

Responses: Thank you very much for the kind suggestion. We have incorporated the procedural details of the conductivity testing (Location of Changes: Line 1223-1229 in page 54). Furthermore, in our design, the conductivity of the PANI layer is essential to the system. Mechanistically, the scaffolds formed by NeuroConnect Blocks promote neural circuit repair primarily through two steps: (1) attracting central neurons to grow directionally along the scaffold via the concentration gradient of LMK-235, and (2) facilitating synaptic reconnection of the recruited central neurons within the scaffold channels through neural signal transmission mediated by the conductive PANI layer. Experimentally, we established control groups in both in vitro or in vivo neuronal synaptic reconnection assays and behavioral tests to demonstrate the critical role of the PANI conductive layer in the system (Fig. 5, 7, 9, 11; Supplementary Figs. 22, 23, 27- 29, 43-45). In addition, by

overexpressing Kir2.1 in neurons to suppress their electrical activity, we directly confirmed that the scaffold's synaptic reconstruction effect is partly dependent on electrical signal conduction mediated by the PANI conductive layer (Fig. 10i-o; Supplementary Figs. 57, 58).

Comments: The authors has still not convincingly demonstrated that it is the conductivity and not the surface properties of PANI that are important. The authors nicely provide analysis of zeta potential which clearly show that the PANI layer provide a neutral/positive surface. The positive surface have been reported to promote neuronal adhesion, neurite outgrowth, and cell spreading. *The above content comprises the reviewers' comments from the first two rounds of peer review, while the following section presents our responses to the comments from the previous round.*

Responses: We thank the reviewer for this important critique and agree with the central point: on our current dataset does not allow us to unambiguously attribute the observed biological effects to the electrical/mixed conductivity, as opposed to PANI-associated surface physicochemical properties (e.g., surface charge, protein adsorption). We therefore revised both the mechanistic interpretation to align strictly with what is supported experimentally.

1) Mechanistic claims were corrected to avoid "conductivity-based causality."

We fully agree that stating that synaptic reconnection is "mediated by conduction through PANI" could be misleading. In the revised manuscript, we therefore removed or softened language implying that bulk conductivity is "crucial" or that neuronal signals propagate through the scaffold because of PANI. We reframed PANI as an electroactive / electrically permissive interface that coincides with improved regeneration and reconnection phenotypes, while explicitly acknowledging that this interface also alters surface charge and interfacial chemistry (***Location of Changes in Results:*** *Line 390-396 in page 16;* ***Location of Changes in Discussion:*** *Line 571-578 in page 22; Line 579-585 in page 23*).

This revision also harmonizes with the updated title/wording changes ("electroactive" rather than "conductive") and prevents readers from inferring that PANI's mixed electronic/ionic conduction is proven to be the causal driver of neuronal signal propagation (***Location of Changes:*** *Line 52-54 in page 2; Line 107-109 in page 4; Line 127-128 in page 6*).

2) Support "an activity-dependent component, not a conduction-only mechanism."

While we cannot isolate conductivity from surface effects, our data provide two lines of evidence supporting an activity-dependent requirement for full functional benefit: 1) NeuroConnect Blocks restored synaptic transmission (patch-clamp *in vitro* and *ex vivo*), consistent with recovery in an

electrically active network. 2) Suppressing neuronal excitability via Kir2.1 attenuated the functional benefit of NeuroConnect Blocks, indicating that neuronal activity is required for full recovery. Importantly, we now interpret these findings as supporting an activity-dependent repair process occurring in the presence of an electroactive scaffold interface, rather than evidence that action potentials are propagated because of scaffold conductivity (***Location of Changes in Results:*** *Line 390-396 in page 16;* ***Location of Changes in Discussion:*** *Line 571-578 in page 22; Line 579-585 in page 23*).

3) Explicitly incorporating the surface-property explanation and strengthening limitations

In line with the reviewer's comment on zeta potential, we explicitly acknowledge in the main text that PANI coating changes both: 1) electrical/electroactive properties (as measured in Fig. 2d), and 2) interfacial physicochemistry (including zeta potential; Fig. 2c), which is known to influence neuronal adhesion/outgrowth. We further strengthened the limitations/future-directions statements to make the mechanistic boundary explicit and to outline what controls would be required to disentangle these contributions (e.g., conductivity-matched but charge-altered controls, charge-matched nonconductive coatings, dedoped PANI, and controlled protein-conditioning studies) (***Location of Changes:*** *Line 571-578 in page 22; Line 579-585 in page 23*).

6. And the authors claim that the nanocarrier contain a conductive polyaniline layer proven by a change in zeta potential and the analysis of conductivity described in figure 2D. The reported conductivity (50-60 mS/cm) is only slightly above CSF (15-20) and blood (5-10) therefore it is hard to understand how this limited conductivity contribute.

Responses: We greatly appreciate your professional comments. The point you raised is indeed critical. Admittedly, in terms of absolute values, the nanocarriers increase conductivity only from 5–20 mS/cm (in body fluids) to 50–60 mS/cm, which may appear to be a modest improvement. However, the core function of the NeuroConnect Blocks design in this study is not merely to elevate bulk conductivity, but rather to direct neural regeneration through the construction of an anisotropic, macroscale conductive architecture. To enhance the reader's understanding, we have incorporated the relevant elaboration into the manuscript (Location of Changes: Line 178-180 in page 7)…more

Comment: The authors change from conductivity matters to anisotropic architecture matters but these are different claims. If the architecture is what is doing the work, then why emphasize

conductivity in the title and throughout the paper? They are essentially conceding the conductivity might not be functionally significant while trying to make it sound like they planned it that way. The numbers problem remains PANI layer 50–60 mS/cm vs CSF 15–20 mS/cm and Blood 5–10 mS/cm. Is it conductivity or surface charge that promote neuronal adhesion, neurite outgrowth, and cell spreading?

The above content comprises the reviewers' comments from the first two rounds of peer review, while the following section presents our responses to the comments from the previous round.

Responses: We thank the reviewer for this critical point and agree our previous framing could be interpreted as shifting the explanation from "conductivity" to "anisotropic architecture" without clearly reconciling the underlying mechanistic claim. We also agree that, given the measured values (≈50–60 mS/cm), our data do not justify attributing neuronal signal propagation or behavioral recovery to bulk electrical conductivity of the PANI layer. In the revised manuscript, we therefore recalibrated both the wording and the interpretation to align strictly with what is supported experimentally.

1) **We removed conductivity-centered causal language and resolved the "conductivity vs architecture" mismatch.**

In the original text, we inadvertently implied that "conductive PANI" is mechanistically required for neuronal signal propagation through the scaffold. We now explicitly avoid this implication. Rather than arguing that "anisotropic conductive architecture" is the dominant mechanism (which indeed represents a different claim), we have revised the manuscript to state that PANI provides an electroactive, electrically permissive interface, and that the observed benefit is best interpreted as activity-dependent repair occurring in the presence of this electroactive interface, without asserting that bulk conductivity is the causal driver. (***Location of Changes:*** *Line 1-2 in page 1; Line 52-54 in page 2; Line 107-109 in page 4; Line 127-128 in page 6; Line 390-396 in page 16; Line 571-578 in page 22; Line 579-585 in page 23*).

2) **We directly addressed the "numbers problem" and clarified what the conductivity measurement does—and does not—support.**

We agree that a conductivity of ~50–60 mS/cm is only modestly higher than CSF and does not, by itself, establish a mechanism in which electronic conduction through the scaffold materially enhances action potential propagation or synaptic transmission. Accordingly, we now treat the conductivity measurement in Fig. 2D as physicochemical characterization confirming that PANI deposition yields an electroactive coating, rather than as proof of functionally dominant "neuronal conduction through

PANI." This point is explicitly stated in the revised Results/Discussion text to prevent over-interpretation (***Location of Changes in Results:*** *Line 390-396 in page 16;* ***Location of Changes in Discussion:*** *Line 571-578 in page 22; Line 579-585 in page 23*).

3) **We explicitly incorporated the alternative explanation raised by the reviewer—surface physicochemistry—and set appropriate boundaries on inference.**

As the reviewer correctly notes, PANI coating changes zeta potential (Fig. 2C) and likely influences additional interfacial properties (e.g., protein conditioning, adhesion, capacitance), all of which can promote neuronal adhesion, neurite extension, and cell spreading independent of bulk conductivity. We therefore explicitly state that our current data cannot disentangle (i) electrical permissiveness/electroactive interfacial effects from (ii) charge-/chemistry-mediated cell–material interactions. This limitation is now integrated in both the mechanistic Results narrative and the Discussion, rather than being implicit (***Location of Changes:*** *Line 571-578 in page 22; Line 579-585 in page 23*).

4) **We strengthened the mechanistic logic to reflect what our data do support: an activity-dependent component, not a conduction-through-PANI mechanism.**

Importantly, our Kir2.1 experiments show that neuronal activity is required for the full functional benefit of NeuroConnect Blocks. This supports an activity-dependent component of repair *in vivo*, but does not prove that PANI's bulk conductivity is the mechanism. We now explicitly interpret this as evidence that the scaffold does not impede, and may provide an interface compatible with, activity-dependent reconnection—while avoiding any claim that the scaffold "conducts" neuronal signals in a way that substitutes for neural tissue (***Location of Changes in Results:*** *Line 390-396 in page 16;* ***Location of Changes in Discussion:*** *Line 571-578 in page 22; Line 579-585 in page 23*).

Overall, we believe these revisions directly address the reviewer's concern by: (i) eliminating potentially misleading "conductive PANI → neuronal signal propagation" statements; (ii) removing the unsupported "anisotropic conductive architecture" rationale; (iii) acknowledging the plausible and well-supported role of surface charge/adhesion; and (iv) aligning the manuscript with a conservative, evidence-based interpretation of PANI as an electroactive interface compatible with activity-dependent repair.

7.But one important aspect is that the negative zeta potential is reduced approaching 0 and it is well

known that this will increase the likelihood of aggregation and most likely increase tissue adherence. Both could potentially be important for retention at the site of injury and neuron adherence.

Responses: Thank you very much for the professional comments and kind advice. We fully agree that, under typical circumstances, a near-neutral Zeta potential markedly reduces colloidal electrostatic stability, thereby leading to aggregation. However, in the nanoparticle design of this study, we circumvented this issue through a steric stabilization mechanism: As described in the manuscript, the nanoparticle surfaces were densely functionalized with long, hydrophilic PEG2000 and PEG5000 chains. These PEG chains extend fully in aqueous environments to form a hydrated layer, which generates substantial steric repulsion between nanoparticles and thereby effectively prevents aggregation. This strategy is widely employed in nanomedicine to ensure colloidal stability under physiological conditions34-39. Moreover, the uniform particle size distribution shown in Fig. 2b directly demonstrates that the nanoparticles remained well-dispersed after preparation, without evidence of aggregation (Fig. 2b).

Comments: If their PEGylation is so effective at preventing all aggregation and tissue interaction, then how do the nanoparticles stay at the injury site? How do neurons adhere to the scaffold? What is the actual mechanism of interaction with neural tissue? *The above content comprises the reviewers' comments from the first two rounds of peer review, while the following section presents our responses to the comments from the previous round.*

Responses: We appreciate the reviewer's insightful comment. In the present design, PEGylation does not serve as a fully inert shielding layer that abolishes all interactions with biological tissues. Instead, dense PEG chains (PEG2000 and PEG5000) primarily provide steric stabilization at the nanoparticle-nanoparticle interface, preventing aggregation even when the *Zeta* potential approaches neutrality. Importantly, the aggregation- or tissue-interactive moieties (bis-5HT) are positioned on the outer side of the PEG layer or at the PEG termini, such that they are not buried within the PEG brush and therefore remain accessible for biological interactions. The outer bis-5HT mediates oligomeric crosslinking reactions between nanoparticles or between nanoparticles and neural tissues[2,3].

Moreover, PEG chains are highly flexible and dynamic in aqueous environments. Upon local aggregation or anchoring within the tissue, the PEG chains may undergo compression and conformational rearrangement, particularly in the mechanically and biochemically complex injury microenvironment. Such PEG chain compression can reduce the effective thickness of the hydrated

PEG layer[4-6]. Under these conditions, the underlying polyaniline-based conductive layer may become partially exposed. Neuronal adhesion in this context is therefore likely mediated, at least in part, by direct interactions between neurons and the exposed electroactively polyaniline surface, which has been widely reported to support neuronal attachment and neurite outgrowth[7,8].

8. In figure 4 c there is an increase of the signal in the head of the mouse but nowhere else. In 4e most of the material is in other organs foremost the liver? Could the authors comment on this. It would be relevant to see the distribution in main organs at earlier time points (0-48 h).

Responses: We sincerely appreciate your insightful questions and constructive feedback. The exclusive enhancement of fluorescence in the mouse head is attributable to the greater ability of near-infrared signals to penetrate and reach brain tissue compared with other organs, thereby generating detectable fluorescence. The observed increased accumulation of NeuroConnect Blocks across all organs is likely due to their enhanced circulation time in the bloodstream, as demonstrated by our pharmacokinetic evaluation (Supplementary Fig. 16). A detailed explanation is provided as follow: 1. The reason for the exclusive enhancement of fluorescence in the mouse head: The enhanced fluorescence observed in the mouse head, but not in other regions, can be attributed to two factors: first, the monitoring was performed with the mouse in a prone position; second, the fur on the head had been shaved for surgical procedures. Under these conditions, near-infrared fluorescence penetrates more readily and reaches the brain compared with other organs. Consequently, at the applied fluorescence threshold, only signals from the head are detectable, primarily reflecting the accumulation of nanoparticles in the brain. 2. The reason for the increased accumulation of NeuroConnect Blocks across all organs: In comparison to the control formulation, NeuroConnect Blocks were modified with a PEG2000 segment (it was introduced by SH-PEG2000-NHS), which likely enhanced their circulation, contributing to increased accumulation in various organs. To demonstrate the long-circulation capability of NeuroConnect Blocks, we have conducted a pharmacokinetic evaluation. The results indicated that the plasma half-life of NeuroConnect Blocks was significantly prolonged compared to the control nanoparticles (Supplementary Fig. 16). Supplementary Fig. 16 The pharmacokinetic properties of NeuroConnect Blocks scaffolds (n = 3). Data represent mean ± SD of three independent biological replicates.

Comment: If NIR penetration was the issue, you would still expect some signal from other

superficial tissues. More importantly, if you can not detect signal from other organs in the live imaging, how can you claim brain-specific targeting? The head signal could be skin, skull, or vasculature, not necessarily brain parenchyma

And early time point data for organ distribution has not been provided. So what fraction actually reaches the brain? What is the brain-to-liver ratio? Is the brain accumulation therapeutically meaningful or just background? If most material goes to liver and other organs, what evidence supports that enough reaches the injured spinal cord/brain to have the claimed therapeutic effects? *The above content comprises the reviewers' comments from the first two rounds of peer review, while the following section presents our responses to the comments from the previous round.*

Responses: Thank you for the careful and constructive reminder. In response to this comment, we have reanalyzed and rearticulated our existing data to confirm the distribution of NeuroConnect Blocks within the brain parenchyma (Fig. 4b) (***Location of Changes:*** *Line 259-268 in page 10*). In addition, we have supplemented *ex vivo* organ imaging at multiple earlier time points and calculated the brain-to-liver ratio to demonstrate improved relative delivery to the brain (Supplementary Fig. 14) (***Location of Changes:*** *Line 253-258 in page 10*). Based on this ratio, together with support from authoritative literature, we consider it reasonable to define the NeuroConnect Blocks as a "brain-targeting" delivery system and to infer that the amount of drug delivered to the brain is sufficient to reach therapeutically relevant concentrations[9-12](***Location of Changes:*** *Line 532-533 in page 21; Line 534-541 in page 22*).

Specifically, to directly test whether NeuroConnect blocks within injured brain parenchyma and assemble in situ, we administered Cou6-labeled NeuroConnect Blocks or Cou6-labeled control nanoparticles and performed confocal imaging on coronal bran sections at 48 h (Fig. 4b). NeuroConnect Blocks formed discrete, multicellular scale aggregates concentrated in the lesion core and peri lesional region, whereas control nanoparticles remained largely diffuse and displayed limited accumulation (Fig. 4b). Notably, the NeuroConnect Blocks signal appeared as compact deposits embedded within the injured tissue architecture rather than as continuous, vessel like trajectories, supporting extravasation followed by assembly in the injured parenchyma.

Furthermore, *ex vivo* imaging performed at multiple time points within 48 h post-injection showed that, compared with the control formulation, NeuroConnect Blocks exhibited markedly enhanced accumulation and retention in the brain (Supplementary Fig. 14a, b). Correspondingly, the brain-to-liver ratio was significantly increased, reaching 15.1515 ± 2.4021% at 48 h (Supplementary Fig. 14c).

Extensive literature reports that the brain delivery efficiency of free drugs is typically below 1%, and even for currently recognized "brain-targeted" delivery systems, the efficiency typically does not exceed 5%[9-12]. Therefore, NeuroConnect Blocks fully meet the prevailing criteria for brain-targeted delivery systems.

Notably, the effective concentration of LMK-235 *in vitro* is 10 nM, whereas the *in vivo* dose of LMK-235 used in this study was 1.5 mg/kg. Taking into account the brain-to-liver ratio, drug release efficiency from the scaffolds, and brain tissue volume, we can reasonably infer that, at this dosing level, the concentration of LMK-235 achieved in the brain is therapeutically meaningful.

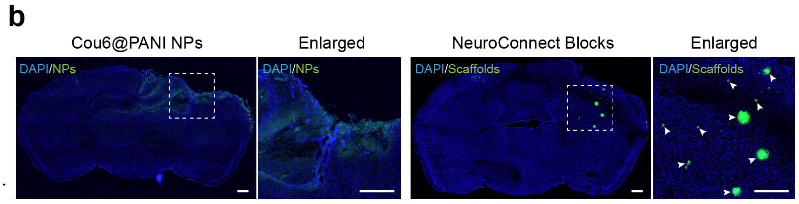

Fig. 4 | NeuroConnect Blocks efficiently target injured brain tissue and assemble into scaffolds *in situ*. b Brain distribution and aggregation of the Cou6-labeled NeuroConnect Blocks. Scale, 500 μm.

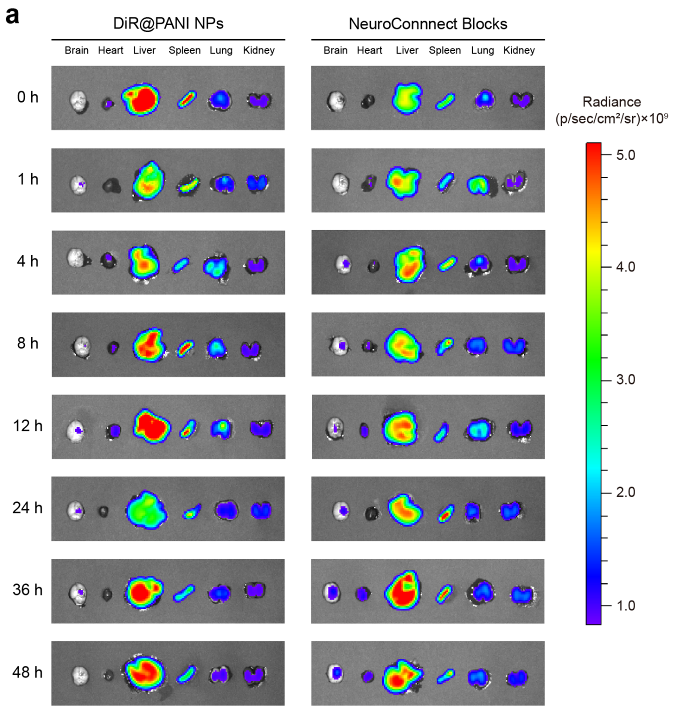

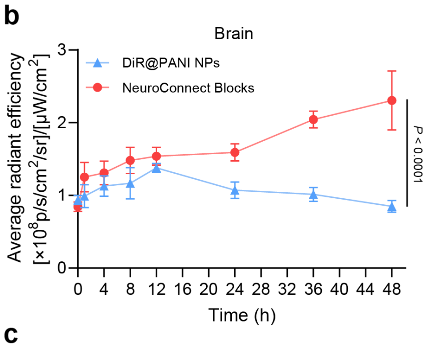

c

Brain-to-Liver Ratio (%)

	DiR@PANI NPs	NeuroConnect Blocks
0 h	4.3761±0.3539	8.9253±0.8959
1 h	6.9874±1.0212	8.2756±1.0832
4 h	7.4126±1.6382	8.2641±0.9945
8 h	6.4807±0.6995	9.2133±1.2845
12 h	6.0592±0.8673	9.9242±1.9607
24 h	8.1954±0.8493	12.8151±1.8600
36 h	4.8078±1.2157	9.8384±0.6862
48 h	3.9349±0.4935	15.1515±2.4021

Supplementary Fig. 14 *Ex vivo* fluorescence analysis at multiple time points. a *Ex vivo* DiR fluorescence imaging of main organs obtained from CCI mice at multiple time points after injection. **b** Semi-quantitative analysis of the *ex vivo* brain radiant efficiency shown in (**a**) (n = 3). **c** Brain-to-liver fluorescence signal ratio shown in (**a**). Data represent mean ± SD of three independent biological

replicates. Statistical analyses are performed using two-way ANOVA with Tukey's post hoc test.

9- Other comments concerning method section and characterization have been addressed.

Responses: Thank you for the valuable comments and constructive suggestions.

References

1. Qin, Y., et al. Harnessing oxidative microenvironment for in vivo synthesis of subcellular conductive polymer microesicles enhances nerve reconstruction. *Nano Lett.* **22**, 3825-3831 (2022).
2. Tang, L., et al. Targeting neutrophils for enhanced cancer theranostics. *Adv. Mater.* **32**, 2002739 (2020).
3. Yao, K., et al. Hesperetin nanoparticle targeting neutrophils for enhanced TBI therapy. *Adv. Funct. Mater.* **32**, 2205787 (2022).
4. Di Fonzo, S., Bellich, B., Gamini, A., Quadri, N. & Cesàro, A. PEG hydration and conformation in aqueous solution: Hints to macromolecular crowding. *Polymer* **175**, 57-64 (2019).
5. Al-Nasassrah, M.A., Podczeck, F. & Newton, J.M. The effect of an increase in chain length on the mechanical properties of polyethylene glycols. *Eur. J. Pharm. Biopharm.* **46**, 31-38 (1998).
6. Drobek, T., Spencer, N.D. & Heuberger, M. Compressing PEG brushes. *Macromolecules* **38**, 5254-5259 (2005).
7. Borah, R., Das, J.M. & Upadhyay, J. Surface functionalized polyaniline nanofibers: chitosan nanocomposite for promoting neuronal-like differentiation of primary adipose derived mesenchymal stem cells and urease activity. *ACS Appl. Bio. Mater.* **5**, 3193-3211 (2022).
8. Eftekhari, B.S., Eskandari, M., Janmey, P.A., Samadikuchaksaraei, A. & Gholipourmalekabadi, M. Conductive chitosan/polyaniline hydrogel with cell-imprinted topography as a potential substrate for neural priming of adipose derived stem cells. *RSC Adv.* 11, 15795-15807 (2021).
9. Nance, E., Pun, S.H., Saigal, R. & Sellers, D.L. Drug delivery to the central nervous system. *Nat. Rev. Mater.* **7**, 314-331 (2022).
10. Pires, P.C. & Santos, A.O. Nanosystems in nose-to-brain drug delivery: A review of non-clinical brain targeting studies. *J. Control. Release.* **270**, 89-100 (2018).
11. Yang, S.C., et al. Body distribution in mice of intravenously injected camptothecin solid lipid nanoparticles and targeting effect on brain. *J. Control. Release.* **59**, 299-307 (1999).
12. Wei, M., et al. Single-particle imaging of nanomedicine entering the brain. *Proc. Natl. Acad. Sci. USA.* **121**, e2309811121 (2024).
